# Loss of mitochondrial DNA helicase in retinal macroglia drives neovascular retinopathy

**DOI:** 10.1038/s44321-026-00438-0

**Published:** 2026-05-08

**Authors:** Sofiia Olander, Sinem Karaman, Fumi Suomi, Kevin Aguilar, Aleksandra Zhaivoron, Maiken Nedergaard, Lina Smeds, Jussi Tiihonen, Albert Quintana, Juan Hidalgo, Kari Alitalo, Petri Ala-Laurila, Gulayse Ince-Dunn, Anu Suomalainen

**Affiliations:** 1 Stem Cells and Metabolism, Research Programs Unit, Faculty of Medicine, University of Helsinki, Helsinki, Finland; 2 Wihuri Research Institute and Translational Cancer Medicine Program, Faculty of Medicine, University of Helsinki, Helsinki, Finland; 3 Institute of Neurosciences, Universitat Autònoma de Barcelona, Bellaterra, Spain; 4 Department of Cellular Biology, Physiology and Immunology, Animal Physiology Unit, Faculty of Biosciences, Universitat Autònoma de Barcelona, Bellaterra, Spain; 5 Center for Translational Neuromedicine, University of Copenhagen, Copenhagen, Denmark; 6 Department of Neurosurgery, Center for Translational Neuromedicine, University of Rochester Medical Center, Rochester, NY USA; 7 Molecular and Integrative Biosciences Research Programme, University of Helsinki, Helsinki, Finland; 8 Department of Neuroscience and Biomedical Engineering, Aalto University, Espoo, Finland; 9 Translational Cancer Medicine Program, Faculty of Medicine, University of Helsinki, Helsinki, Finland; 10 HUS Diagnostic Center, Helsinki University Hospital, Helsinki, Finland; 11 HiLife, University of Helsinki, Helsinki, Finland

**Keywords:** Metabolism, Neuroscience

## Abstract

Retinopathy is a common symptom in mitochondrial diseases, and a leading cause of blindness in working-age individuals, often arising as a consequence of diabetes. Here, we demonstrate that postnatal loss of the replicative helicase of mitochondrial DNA in the astrocytes and Müller glia induces neovascular retinopathy. In these retinas, the macroglia show pathological reactivation, leading to hallmark features of neovascularization with blood-retina-barrier leakage, secondary microgliosis, and complement cascade activation. Similar reactivation of astrocytes in the cerebral cortex does not compromise vascular integrity, indicating tissue-specific roles of mitochondrial metabolism in macroglia for vascular homeostasis. Three secreted angiogenic factors—Fgf2, Pgf, and Lcn2—known to contribute to diabetic retinopathy, were induced. Spike recordings of the most sensitive retinal ganglion cells revealed normal rod function and intact retinal coding. These findings highlight the critical role of glial mitochondrial metabolism in neovascular retinopathy, with important implications for therapy development for mitochondrial and common forms of vision loss.

## Synopsis

Loss of mitochondrial Twinkle helicase in retinal astrocytes causes pathological glial reactivation, secondary neovascularization, blood vessel leakage and inflammation, consistent with neovascular retinopathy. The data indicate the importance of astrocytic mitochondria in retinal homeostasis.

- Twinkle and mtDNA loss in retina causes reactive astrogliosis, neovascularization and vascular leakage.
- Retinal glial reactivation upregulates angiogenic factors, neuroinflammatory cytokines and complement cascade activation.
- Macroglial mitochondria play a key role in neovascular retinopathy pathogenesis.

## Abstract (web summary)

Loss of mitochondrial Twinkle helicase in retinal astrocytes causes pathological glial reactivation, secondary neovascularization, blood vessel leakage and inflammation, consistent with neovascular retinopathy. The data indicate the importance of astrocytic mitochondria in retinal homeostasis.

## Graphical abstract

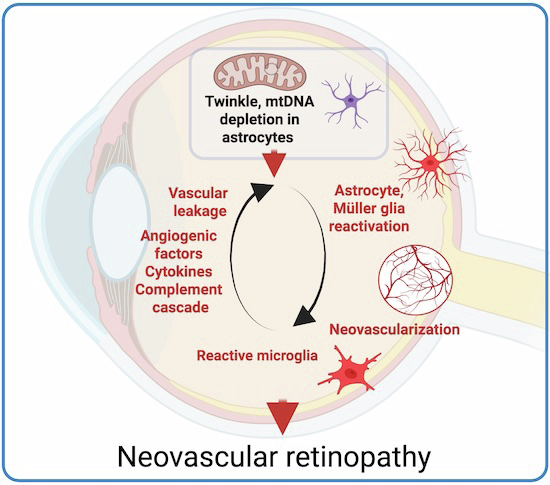

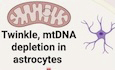

The paper explained **Problem**

Retinopathy is a leading cause of blindness in working-age adults, commonly associated with metabolic disorders such as diabetes and rare genetic defects in mitochondrial proteins. Typically, photoreceptors and retinal ganglion cells are the primary cell types affected, often accompanied by inflammation, as well as abnormal growth and hemorrhaging of retinal blood vessels. Research has focused predominantly on degenerating neuronal subtypes, leaving the metabolic and pathological contributions of astrocytes poorly characterized. As key regulators of the blood-retina barrier, neurovascular coupling, and metabolic homeostasis, astrocytes are uniquely positioned to either mitigate or exacerbate retinal degeneration. Therefore, a deeper investigation into astrocyte-driven mechanisms in retinopathy is critical for understanding disease mechanisms and developing targeted therapeutic strategies.

**Results**

We demonstrate that the loss of the replicative helicase of mitochondrial DNA, Twinkle, in the retinal astrocytes and Müller glia induces neovascular retinopathy. The retina shows remarkable reactivation of astrocytes, retinal neovascularization accompanied by blood-retina-barrier leakage, secondary microgliosis, and complement cascade activation. Intriguingly, Twinkle loss and similar reactivation of astrocytes in the cerebral cortex do not disrupt vascular integrity, indicating that mitochondrial metabolism in macroglia contributes to vascular homeostasis in a tissue-specific manner. We identified three secreted factors with known angiogenic properties and involvement in diabetic retinopathy: Fgf2, Pgf, and Lcn2.

**Impact**

Our findings identify macroglial mitochondria as regulators of retinal vascular homeostasis and suggest their involvement in neovascular retinopathy, sharing key features with diabetic retinopathy. This work highlights macroglial cells as potential therapeutic targets in metabolic retinopathies associated with gliosis, impaired blood–brain barrier integrity, and neuroinflammation.

## Introduction

Retinopathies are common causes of blindness, most often manifesting as a complication of common metabolic diseases such as diabetes, as well as rare inherited metabolic disorders, such as primary mitochondrial diseases (Patel et al, 2019; Wong et al, 2016; Yu-Wai-Man et al, 2011). The retina is one of the most metabolically active tissues in the body (Wong-Riley, 2010). Therefore, bioenergetic defects affecting the cellular centers of metabolism, the mitochondria, challenge its homeostasis. However, not all mitochondrial diseases cause retinal degeneration, indicating that the pathogenesis is not explained solely by energy supply. The degenerating cell types in retinal degeneration are primarily the photoreceptors and retinal ganglion cells (RGCs), and the pathogenesis is frequently accompanied by inflammation as well as pathological growth and hemorrhaging of the new retinal blood vessels (Ferrington et al, 2020). To date, studies of retinal metabolic diseases have mainly focused on the degenerating neuronal subtypes, while the roles of the glial subtypes in regulating metabolic homeostasis and diseases of the retina are poorly understood.

Two types of retinal glial cells, the astrocytes and Müller glia, are located near the blood vessels, where the retinal nutrient and oxygen demand meet the blood supply (Joyal et al, 2018). Retinal astrocytes are a little-studied glia type in the retina. In the mouse retina, retinal astrocytes together with Müller glia make up 3–5% of all retina cells, and of those, Müller glia are the major subtype, forming 90% of macroglia (Vecino et al, 2016). Therefore, macroglia, especially astrocytes, are often absent in single-cell RNA sequencing datasets. Astrocytes are strictly localized to the nerve fiber layer located in the inner retina. They interact closely with RGC cell bodies and axons, and the blood vessels that feed this layer. Apart from the outer photoreceptor segments, Müller glia span the rest of the retinal layers, interacting with all cell types. End-feet projections of both the astrocytes and the Müller glia wrap almost completely around blood vessels and communicate via specific signaling molecules with endothelial cells (Mathiisen et al, 2010). Upon injury or disease, Müller glia and astrocytes transition into a “reactive” state defined by profound changes in cell morphology, gene expression, and metabolism (Coorey et al, 2012). Reactive gliosis can be toxic or protective depending on the nature, magnitude, and duration of the metabolic insult (Graca et al, 2018). Perhaps surprisingly, the contribution of highly metabolic macroglia in retinal diseases is understudied, as past studies of mitochondrial disease have overwhelmingly focused on the degenerating RGC population.

Defects in mitochondrial DNA (mtDNA) expression and maintenance, caused by mutations in nuclear or mtDNA-encoded mitochondrial proteins, are often associated with retinal pathology. Retinitis pigmentosa is the most common type of mitochondrial retinopathy resulting from defects in ATP synthase or mtDNA replication (Schrier and Falk, 2011). In the mtDNA replication fork, the helicase Twinkle initiates the replication and is the main regulator of replication frequency (Spelbrink et al, 2001; Tyynismaa et al, 2004). Previous reports have shown that deletion of the Twinkle and subsequent loss of mtDNA in forebrain astrocytes (TwKO^Astro^ mice) leads to pervasive reactive astrogliosis, spongiotic degeneration, reactive microgliosis, and myelin disorganization, closely resembling human spongiotic encephalopathies resulting from mtDNA depletion (Ignatenko et al, 2018; Kollberg et al, 2006; Palin et al, 2012). Surprisingly, similar mtDNA loss in cortical neurons was well tolerated until seven months of age, after which the mice manifested with an acute onset of rapidly progressing neurodegenerative disease (Ignatenko et al, 2018). The results highlighted that mtDNA loss and consequent energy metabolic deficiency in astrocytes can be a primary driver of disease. Considering that the retina is the most metabolically active tissue in the body, the retinal responses to mitochondrial insult in astrocytes are of high interest (Ames et al, 1992; Wong-Riley, 2010).

Here, we report that astrocytic/Müller glia depletion in TwKO^Astro^ has brain region-specific effects in the nervous system. The cerebral cortex and retina both show remarkable reactive gliosis, but the consequences are different: neovascularization and vascular leakage in the retina, while the cerebral cortex of TwKO^Astro^ mice has intact vasculature and spongiosis. Our data show that reactivation of retinal glia induces a combined response of angiogenic factors, neuroinflammatory cytokines, and complement cascade activation. Our evidence indicates an intriguing role of retinal macroglia reactivation in the pathogenesis of neovascular retinopathy, introducing a new model for the disease and revealing potential targets for treatment.

## Results

### Knockout of Twinkle in retinal astrocytes and Müller glia induces reactive gliosis, neovascularization, and vascular leakage

**Figure 1:**
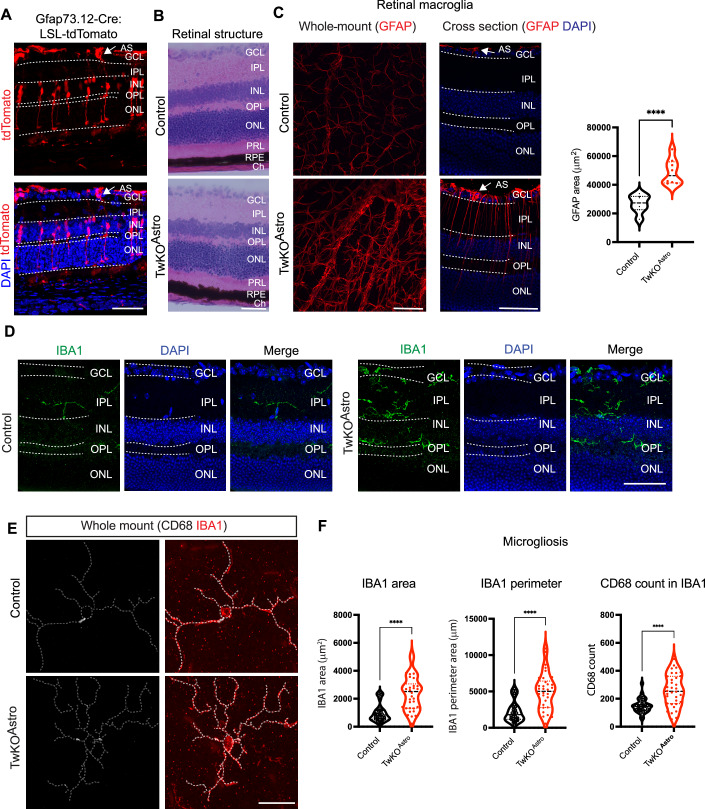
Reactive gliosis in the retina of mice. (**A**) Cre-transgene expression in astrocytes and Müller glia. Cre-activity marked by Gfap73.12-Cre:LSL-tdTomato expression (red). The white dotted line delineates the boundary between retinal layers. (**B**) Retinal structure, hematoxylin and eosin staining. (**C**) Astrocytes and Müller glia, marked by glial fibrillary acidic protein GFAP (red), immunofluorescence analysis. Retinal whole-mount preparation (maximum intensity projection of z-stack) and cross-section. The white dotted line delineates the boundary between retinal layers. (**D**) Microglia marked by IBA1 (ionized calcium-binding adapter molecule 1) immunofluorescence staining, retinal cross-section. The white dotted line delineates the boundary between retinal layers. (**E**) Microglia, immunofluorescent staining of the whole-mount retina against microglial marker IBA1 and the activated microglial marker CD68. An IBA1-positive microglial cell is outlined with white dotted lines. (**F**) Quantification of microglial cell area, perimeter, and activated microglia amount (whole-mount IBA1/CD68 staining). Seven to eight locations (field of view) in each whole-mount retina were imaged with ×40 objective, analysis by Cell Profiler. Each dot represents the number of microglia per field of view. Control *n* = 4, TwKO^Astro^ *n* = 4. Data represent mean ± SEM. *P* values were calculated using unpaired two-tailed parametric *t* test. Statistical significance: IBA1 area; ****P = 0.0000003, IBA1 perimeter; ****P = 0.000001, CD68 count in IBA1; ****P = 0.000008. GCL ganglion cell layer, IPL inner plexiform layer, INL inner nuclear layer, OPL outer plexiform layer, ONL outer nuclear layer, PRL photoreceptor layer, RPE retinal pigment epithelium, Ch choroid. Scale bars: 50 μm (**A**), 100 μm (**B**), 100 μm, and 50  μm (**C**), 50 μm, (**E**) 50 μm. Source data are available online for this figure.

**Figure EV1:**
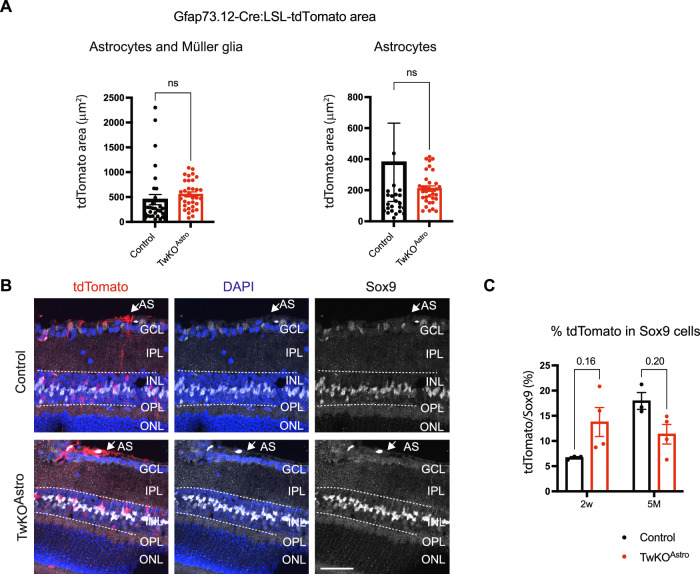
Validation of Twinkle inactivation in retinal macroglia of TwKO^Astro^ mice. (**A**) Quantification of cells expressing tdTomato under GFAP-promoter in 5-month-old TwKO^Astro^ and control mice (Gfap73.12-Cre:LSL-tdTomato). Left panel: quantification of retina section of 205.8 × 205.8 μm^2^ image area including AS, GCL, IPL, INL, OPL, and ONL. Right panel: quantification of cropped astrocyte layer 100 × 100 μm^2^. Six to nine locations in each retina section were imaged. Each dot represents an image. No difference in recombination activity between genotypes (*P* = 0.2301 for the left panel and *P* = 0.1692 for the right panel). Data represent mean ± SEM. *P* values were calculated using two-tailed unpaired *t* test. Statistical significance: *P*  <  0.05. Control mice, *n* = 3; TwKO^Astro^ mice, *n* = 3. (**B**) Müller glia, representative image. immunofluorescence with Sox9 antibody, retinal cross-section from 5-month-old mice. The white dotted line indicates the inner nuclear layer (INL) with Sox9-positive Müller glia cells and tdTomato-positive cells. Scale bar: 50 µm. (**C**) Quantification of GFAP-promoter activity in Müller glia; tdTomato/Sox9 ratio per image (2 weeks and 5 M old control *n* = 3, TwKO^Astro^ *n* = 4). Eight to 14 different locations in each retina section were imaged; ×60 objective. Data represent mean ± SEM. *P* values were calculated using two-way ANOVA multiple comparisons. Statistical significance: *P*  <  0.05. AS astrocytes, GCL ganglion cell layer, IPL inner plexiform layer, INL inner nuclear layer, OPL outer plexiform layer, ONL outer nuclear layer. Source data are available online for this figure.

**Figure EV2:**
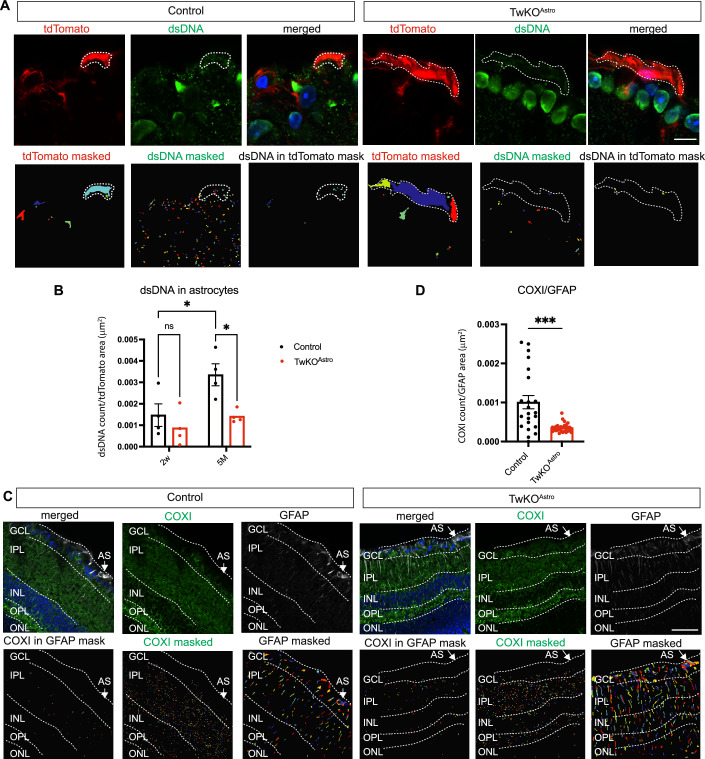
Quantification of mtDNA and its translation product cytochrome c oxidase subunit I (COX-I) in retina. (**A**) mtDNA in TwKO^Astro^. Upper panel: Double-stranded DNA (dsDNA) immunofluorescence (green) in retinal cross-section with tdTomato (red). Lower panel: Cell Profiler masked images. Outlined with a white dotted line, examples of tdTomato-positive astrocytes in the vicinity of the ganglion cell layer (green nuclei). Scale bar: 10 µm. (**B**) Quantification of dsDNA signal in tdTomato-positive astrocytes (count/µm^2^). Control *n* = 4, TwKO^Astro^ *n* = 4. Six to nine locations in each retina section were imaged using a ×60 objective. The Cell Profiler pipeline was designed to quantify dsDNA within the tdTomato-positive cells in each image. Overlapping dsDNA signals within the nuclear (DAPI) signal were omitted. dsDNA count is normalized to the area of tdTomato positive signal area. Each dot in the graph represents an animal. Data represent mean ± SEM. *P* values were calculated using two-way ANOVA multiple comparisons. Statistical significance: Control 2 M vs 5 M; *P = 0.041. 5 M Control vs TwKO^Astro^; *P = 0.034. (**C**) Mitochondrial DNA encoded protein expression: cytochrome c oxidase subunit I (COX-I). Upper panel: COX-I and GFAP immunofluorescence; retina cross-section; Lower panel: Cell Profiler masked images. Control *n* = 3, TwKO^Astro^ *n* = 4. Six to nineteen locations in each retina section were imaged using a ×60 objective. The white dotted line delineates the boundary between retinal layers. Scale bar: 50 µm. (**D**) Quantification of COX-I in GFAP-positive astrocytes. Control *n* = 4, TwKO^Astro^ *n* = 4. Six to nine locations in each retina section were imaged using a ×60 objective. Each dot in the graph represents an image from which all positive COXI/GFAP-positive signals were calculated. Data represent mean ± SEM. *P* values were calculated using two-tailed unpaired *t* test. Statistical significance: ***P  =  0.0001. AS astrocytes (pointed with a white arrow), GCL ganglion cell layer, IPL inner plexiform layer, INL inner nuclear layer, OPL outer plexiform layer, ONL outer nuclear layer. Source data are available online for this figure.

**Figure EV3:**
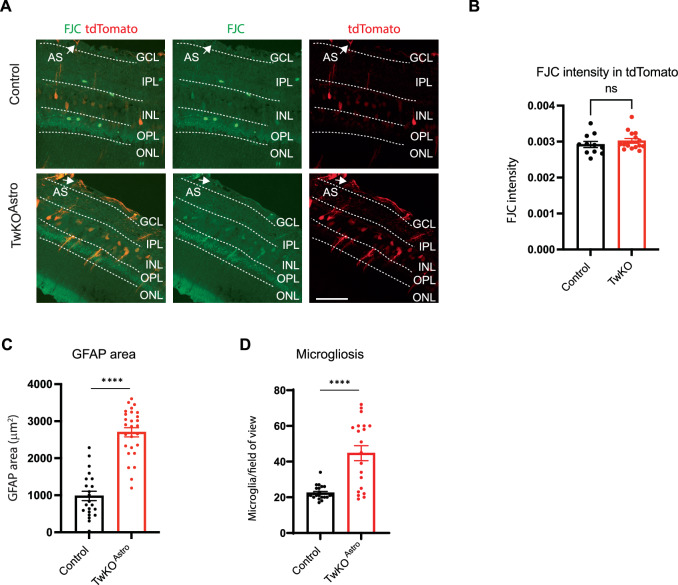
Quantification of microglial and GFAP-expressing cells. (**A**) Cell death in retina of TwKO^Astro^ and control mice, Fluoro-Jade C (FJC) staining (green); tdTomato (red). White dotted line delineates the boundary between retinal layers. Scale bar: 50 µm. AS astrocytes, GCL ganglion cell layer, IPL inner plexiform layer, INL inner nuclear layer, OPL outer plexiform layer, ONL outer nuclear layer. (**B**) Quantification of (**D**); FJC intensity in cells expressing Gfap73.12-Cre:LS-tdTomato. Data represent mean ± SEM. Four to six locations in each retina section were imaged. Each dot represents an image. Control mice, *n* = 3; TwKOAstro mice, *n* = 3. *P* values were calculated using two-tailed unpaired *t* test. Statistical significance: *P  <  0.05. (**C**) Macroglial (GFAP area) quantification on retina sections. Control n = 4, TwKO^Astro^ *n* = 4. Six to nine locations in each retina section were imaged for 10 µm z-stack using a ×60 objective. Cell Profiler pipeline was designed to quantify GFAP area for the maximum intensity projection of z-stack in each image. Data represent mean ± SEM. *P* values were calculated using two-tailed unpaired *t* test. Statistical significance: ****P  = 1.341 × 10^−8^. Each dot in the graph represents each image. (**D**) Microglia in whole-mount retinas; IBA1 staining. Six locations in each retina were imaged using a ×20 objective. The number of cell bodies in each image (“field of view”) was counted, with each dot in the graph representing the cell count per field of view. Control *n* = 4. TwKO^Astro^ *n* = 4. Statistical significance: ****P = 3 × 10^−6^. Source data are available online for this figure.

We previously engineered mice with astrocyte-specific postnatal inactivation of Twinkle helicase, leading to mtDNA depletion, spongiotic encephalopathy with brain parenchyma vacuolization, and extensive astrocyte reactivation (Ignatenko et al, 2018). These TwKO^Astro^ mice (Gfap-Cre73.12;Twnk^LoxP/LoxP^) express CRE also in their retina, in the astrocytes and Müller glia. We confirmed the macroglial expression pattern of CRE by crossing the TwKO^Astro^ line to a ROSA26-CAG-tdTomato reporter line (Figs. 1A and  EV1A). The retinal astrocytes reside in the vitreal lining that in the TwKO^Astro^ showed a tomato-positive, reactivated astrocyte phenotype. The recombination efficiency in Müller cells was less than 20% (Fig. EV1B,C). Next, loss of Twinkle function in retinal astrocytes was confirmed by analyzing downstream consequences of Twinkle deficiency, mtDNA amount, and its encoded cytochrome c oxidase subunit I (COX-I). Both mtDNA and COX-I protein showed progressive depletion in tdTomato-positive astrocytes, when measured at 2-week or 5-month time points (Fig. EV2A–D). No significant increase in cell death or degeneration was present (Fig. EV3A,B).

Unlike the cerebral cortex, the retina of TwKO^Astro^ mice showed no vacuolization, and the overall tissue organization of TwKO^Astro^ retina was control-like in the morphological organization of the tissue layers (Fig. 1B). Next, we immunostained retinal whole-mount explants and cross-sections with an antibody against GFAP, a standard marker upregulated in reactive glia (Graca et al, 2018). Astrocytes and also some Müller glia were remarkably reactivated, showing higher GFAP expression compared to controls, hypertrophy of cell bodies, and thickening of processes (Figs. 1C and EV3C). IBA1 immunostaining indicated also secondary microglia proliferation and activation, similar to the cerebrum of the same TwKO^Astro^ animals (Figs. 1D,F and EV3D). Both IBA1-positive microglial area and perimeter were significantly increased in the TwKO^Astro^ retina, as well as the CD68 count within the IBA1 area (Fig. 1E,F). The morphological changes of microglia were consistent with a hyper-ramified state, representing an intermediate state between resting and reactive microglia.

**Figure 2:**
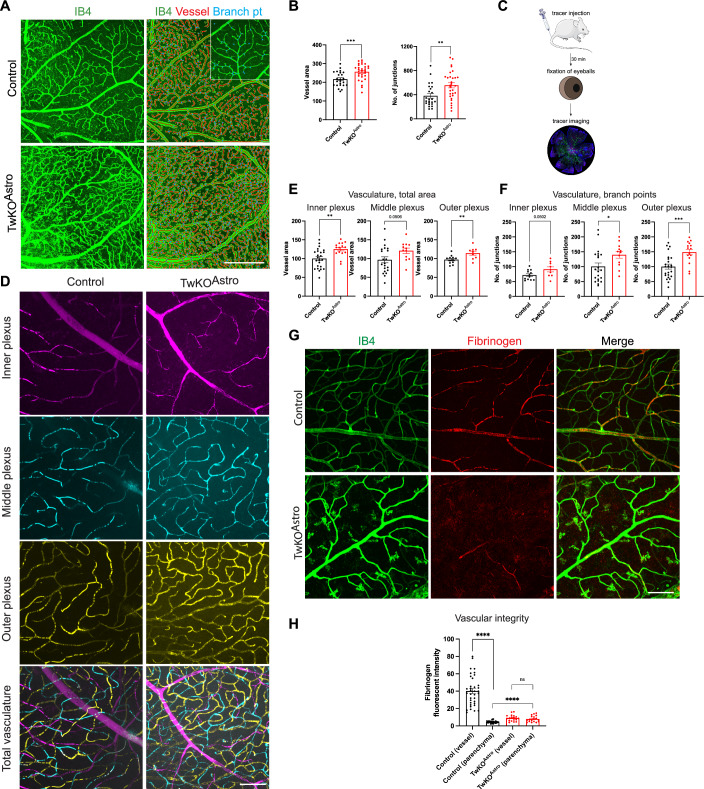
Neovascular retinopathy of TwKO^Astro^ mice. (**A**) Blood vessels in whole-mount retina. Isolectin-IB4 immunofluorescence, analyzed by AngioTool. Inset (upper right corner) indicates the branch points detected by AngioTool. (**B**) Quantification of (**A**): vascular area and branch number of whole-mount retina; AngioTool analysis. Control *n* = 8, TwKO^Astro^ *n* = 10. Statistical significance: Vessel area; ***P = 0.000915. No. of junctions; **P = 0.004561. (**C**) Schematic presentation of the retinal vasculature analysis. Tracer: FITC-dextran. (**D**) The three vascular layers in the whole-mount retina stained by FITC-dextran fluorescent tracer; pseudo-colored. Quantification of (**E**); total blood vessel area and (**F**) branch points; AngioTool analysis. Control *n* = 8, TwKO^Astro^ *n* = 6. (**E**) Statistical significance: Inner plexus; **P = 0.0084, Outer plexus; **P = 0.0026. (**F**) Statistical significance: Middle plexus; *P = 0.0354, Outer plexus; ***P = 0.0006. (**G**) Vascular integrity; whole-mount retina; immunofluorescent analysis of intravascular fibrinogen protein (intact vessels) and extravasated fibrinogen (vessel leakage). IB4 staining for vasculature. (**H**) Quantification of fibrinogen signal in blood vessels and surrounding parenchyma in whole-mount retina. Statistical significance: Control *n* = 7, TwKO^Astro^ *n* = 6. Control vessel vs parenchyma; ****P = 2.01 × 10^−^^14^, Control vs TwKO^Astro^ parenchyma; ****P = 0.000003. Data represent mean ± SEM. Each dot represents an individual measurement. Calculation of *P* values: unpaired two-tailed parametric *t* test. Near-significant comparisons (*P*  <  0.06) are reported with exact *P* values. Scale bars: 200 μm (**A**), 100 μm (**D**, **G**). Source data are available online for this figure.

Since Müller glia- and astrocyte-derived signals can promote vascularization during development, we explored the vascular architecture of the retina in TwKO^Astro^ mice. Isolectin-B4 (IB4)-stained retinal flat-mount preparations from adult TwKO^Astro^ and control mice revealed a significant increase both in coverage of total vascular area and number of vessel junctions (Fig. 2A,B). Imaging of an intravenously injected FITC-dextran fluorescent tracer in whole flat-mount retinas confirmed neovascularization (Fig. 2C,D). The neovascularization was present in all three inner retinal vascular beds (Fig. 2D–F). These findings showed that TwKO^Astro^ in astrocytes and Müller glia led to reactive gliosis, microglia activation, and pathological neovascularization in the retina.

To test whether the abnormal vascularization affected vascular integrity (BRB, blood retina barrier), we analyzed the localization of fibrinogen, a plasma protein, which in intact vessels resides inside the vessel walls. In TwKO^Astro^ mouse whole-mount retinas, a remarkable leakage of fibrinogen into the parenchyma was detected, outside of the vessel lumen. These results indicated that loss of mtDNA helicase activity exclusively in retinal macroglia population leads to vascular disorganization and leakage (Fig. 2G,H).

### Retinal vascular structure is not affected in other mitochondrial disease models

**Figure EV4:**
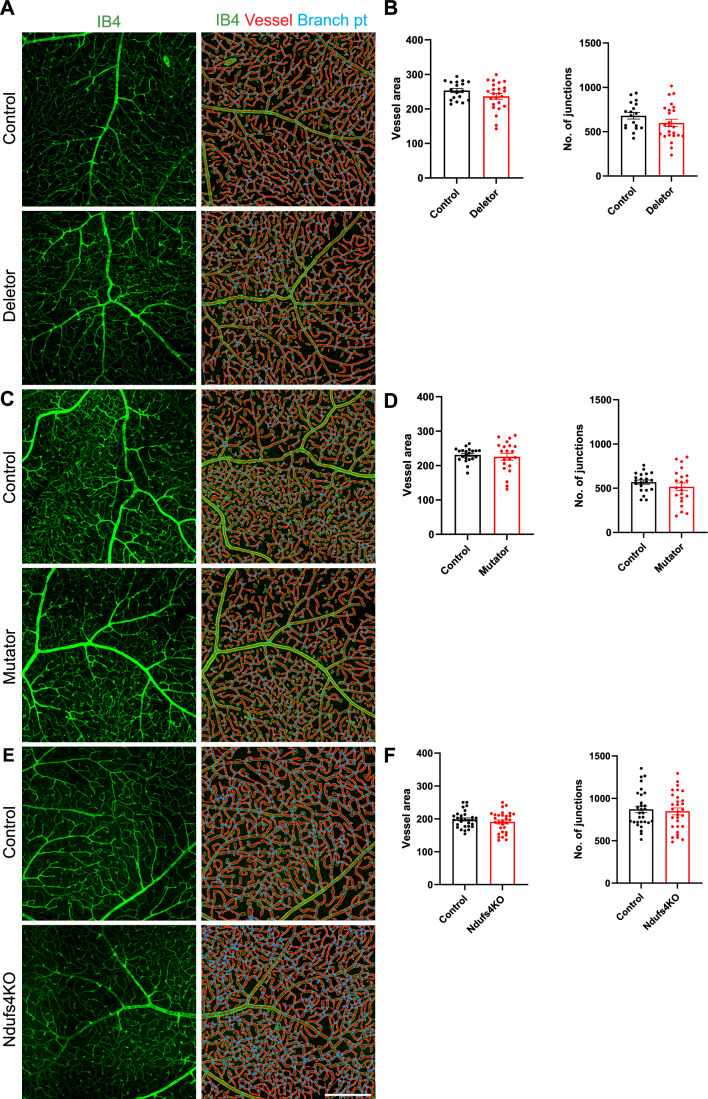
Retinal vasculature in different mitochondrial disease models. IB4 staining of retinal whole mounts and their corresponding AngioTool quantification. (**A**, **B**) Deletor mice (*n* = 8) and littermate controls (*n* = 6). (**C**, **D**) Mutator mice (*n* = 7) and their littermate controls (*n* = 7). (**E**, **F**) Ndufs4KO (*n* = 6) and their littermate controls (*n* = 6). Data represent mean ± SEM. Each dot represents an individual measurement. *P* values were calculated using unpaired two-tailed parametric *t* test. Scale bar, 200 μm. Source data are available online for this figure.

Next, we studied whether retinal astrocyte reactivity and neovascularization were present in other mouse models, similar to TwKO^Astro^. We analyzed Müller glia and astrocyte reactivity and vessel growth parameters in adult retinas of deletor, mutator, and *Ndufs4* knockout mice (*Ndufs4*KO). Briefly, the “mtDNA deletor” mice manifest adult-onset mitochondrial myopathy as a consequence of ubiquitously expressed dominant patient mutation in Twinkle (Tyynismaa et al, 2005), leading to accumulation of multiple large mtDNA deletions in postmitotic cells and gradual development of respiratory chain deficiency. The “mtDNA mutator“ mice carry a knock-in mutation in the exonuclease domain of the catalytic subunit of DNA polymerase gamma, the replicative polymerase of mtDNA. The loss of proof-reading capacity of the polymerase leads to random mutagenesis of mtDNA (Kujoth et al, 2005; Trifunovic et al, 2004) and a respiratory chain deficiency in postmitotic tissues. Also, we included a mouse model for Leigh Syndrome, with constitutive knockout of NDUFS4 subunit of the respiratory chain complex I, with respective decrease of the enzyme activity (Kruse et al, 2008). The mice were tested at ages at which their phenotype was well manifest, with their littermate controls. No reactivation of Müller glia and/or astrocytes was found in these mice, and their vessel architecture was comparable with that of their controls (Fig. EV4A–F). The findings suggested specificity of the vascular findings to the loss of mtDNA helicase activity in macroglia in TwKO^Astro^. Importantly, all the tested models, including TwKO^Astro^, share the downstream consequence of respiratory chain deficiency, as they are either affecting mtDNA replication, or directly a respiratory chain enzyme subunit. Therefore, the evidence suggests that respiratory chain deficiency per se does not induce reactive gliosis and neovascularization in the retina.

### Differential responses of retina and cerebral cortex vasculature to glial mitochondrial stress

**Figure 3:**
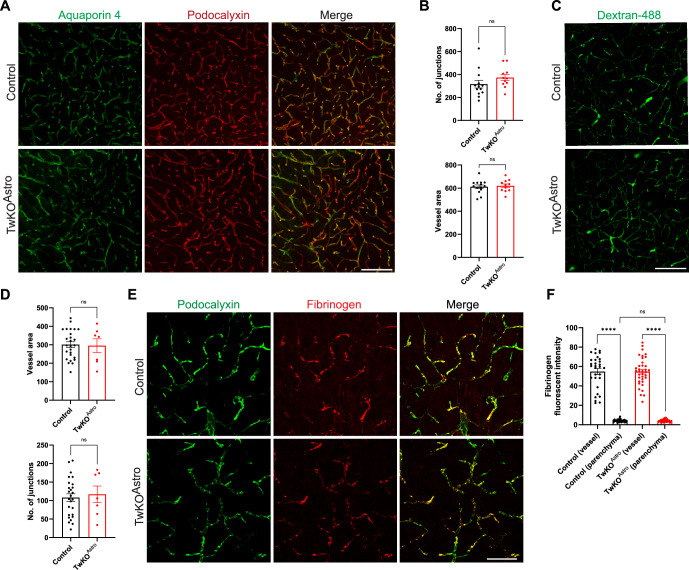
Integrity and structure of cerebral cortex vasculature of TwKO^Astro^ mice. (**A**) Endothelial cells (podocalyxin) and astrocyte end-feet (aquaporin 4); immunofluorescent staining, cerebral cortex. (**B**) Quantification of (**A**), vasculature area and junctions in the cerebral cortex; Angiotool analysis. Control *n* = 4, TwKO^Astro^ *n* = 3. (**C**) Vessel density in the cerebral cortex. Intravascularly injected FITC-dextran fluorescent tracer. (**D**) Vascular parameters were quantified with AngioTool based on FITC-dextran distribution in the cerebral cortex. Control *n* = 10, TwKO^Astro^ *n* = 4. (**E**) Integrity of vasculature; intravascular fibrinogen, immunofluorescent staining; cerebral cortex. (**F**) Quantification of fibrinogen signal in blood vessels and brain parenchyma, cerebral cortex. Control *n* = 7, TwKO^Astro^ *n* = 8. Statistical significance: Control vessel vs parenchyma; ****P = 3.81 × 10^−^¹⁶, TwKO^Asto^ vessels vs parenchyma; ****P = 1.27 × 10^−^²¹. Data represent mean ± SEM. Each dot represents an individual measurement. *P* values were calculated using unpaired two-tailed parametric *t* test. Scale bars: 100 μm (**A**), 50 μm (**C**, **E**). Source data are available online for this figure.

Next, we asked whether the vascular overgrowth and barrier leakage in TwKO^Astro^ retina were present in other parts of the nervous system, as the Gfap-Cre73.12 is also expressed in astrocytes of the forebrain and causes their extensive reactivation (Ignatenko et al, 2018). Vessel coverage and junction (branch point) density in the cortex, assessed by staining cortical tangential sections in TwKO^Astro^ mice for the endothelial cell marker Podocalyxin (PODXL) was comparable to that of control mice (Fig. 3A,B). Intravascularly injected FITC-dextran fluorescent tracer distribution, as a marker of vessel density (Fig. 3C,D), also showed no abnormality. The data indicate that the reactivated astrocytes did not cause vascular abnormalities in the cerebral cortex of TwKO^Astro^ mice.

**Figure 4:**
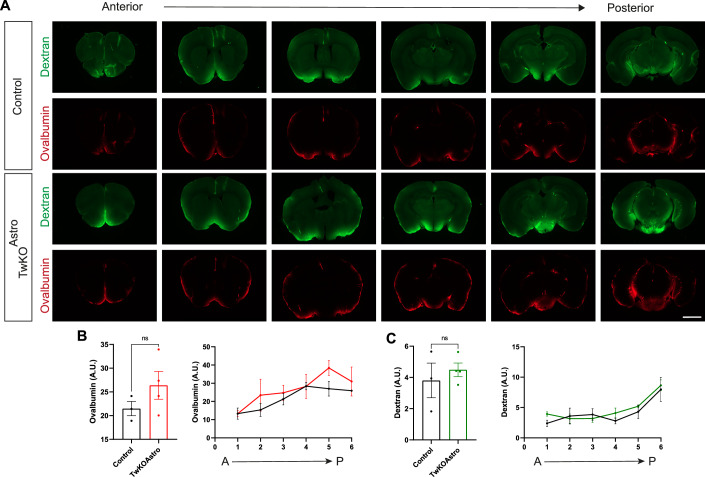
Paravascular fluid flow dynamics in TwKO^Astro^ mice. (**A**) Influx of fluorescent cerebrospinal fluid tracers, dextran-FITC (3 kDa; green) and ovalbumin-conjugated Alexa-647 (45 kDa; red); intracisternal injections into the mouse subarachnoid space; tracing time 30 min. (**B**) Quantification of ovalbumin and (**C**) dextran as a percentage of whole brain slice fluorescence. Data represent mean ± SEM. Control *n* = 3, TwKO^Astro^ *n* = 4. Dots represent an average measurement per mouse or an average per brain slice. *P* values were calculated using unpaired two-tailed parametric *t* test. Scale bar, 2000 μm. Source data are available online for this figure.

To further test the functional capacity of reactivated cortical astrocytes in the maintenance of vascular homeostasis, we explored the integrity of the blood-brain barrier (BBB) and the function of the paravascular clearance system, two processes dependent on astrocyte end feet function (Bohr et al, 2022). First, fibrinogen distribution was localized to vessel lumens in the cortical tissue, and no tissue extravasation was observed in TwKO^Astro^ cortex (Fig. 3E,F). Second, we tested the function of the paravascular fluid flow system, by which cerebrospinal fluid (CSF) circulates through the perivascular space of brain vessels into the brain parenchyma. We injected a mixture of two fluorescent tracers with molecular weights of 3 kDa (Dextran-FITC) and 45 kDa (Ovalbumin-Alexa647) into the CSF through the cisterna magna of mice under anesthesia. After allowing the tracers to enter the brain parenchyma for 30 min, mice were sacrificed, and the brains were collected for sectioning and quantification of tracer intensity. Our quantification of tracer amounts in the brain parenchyma revealed similar amounts in TwKO^Astro^ and control mice (Fig. 4A–C). These results indicated that the lack of Twinkle in cerebral cortex astrocytes did not cause defects in BBB maintenance or paravascular fluid flow and that the vascular findings were retina-specific.

### Innate immune activation and neuroinflammation in TwKO^Astro^ retina

**Figure 5:**
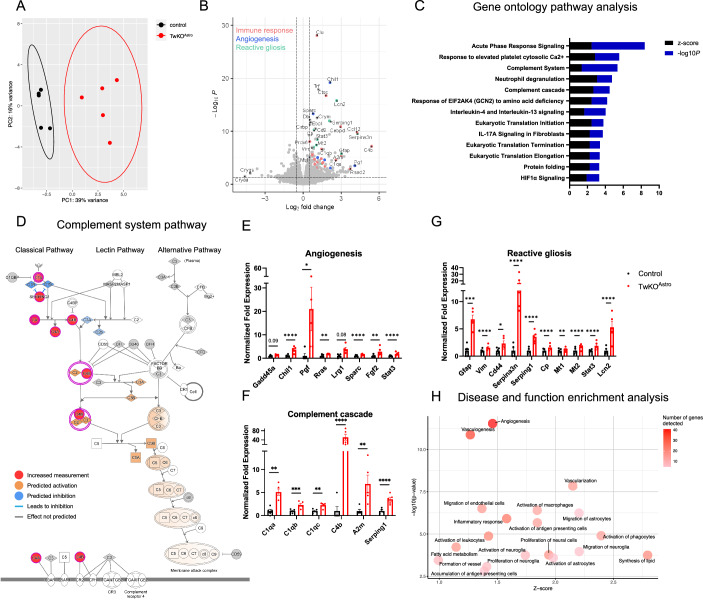
Retinal transcriptomes, pro-angiogenic signals, and neuroinflammation. (**A**) Transcriptome analysis of retina; principal component analysis (PCA) of RNA-sequencing data from control and TwKO^Astro^. (**B**) Transcripts of retina; volcano plot of all transcripts in TwKO^Astro^ retina compared to control retina. Immune response transcripts (red), angiogenesis (blue), and reactive gliosis (green). Dashed line: *P* = 0.05. (**C**) Gene ontology pathway analysis of TwKO^Astro^ retina transcriptome compared to controls (FDR < 0.1). (**D**) Complement system pathway highlighting differentially expressed genes in TwKO^Astro^ retina; Ingenuity Pathway Analysis. (**E**–**G**) Transcripts of (**E**) angiogenesis pathway, (**F**) complement cascade, and (**G**) reactive gliosis markers in TwKO^Astro^ retina. (**E**) ****P = 4.32 × 10^−16^ (*Chil1*), *P = 0.04 (*Pgf*), **P = 0.002 (*Rras*), ****P = 1.13 × 10^−10^ (*Sparc*), **P = 0.005 (*Fgf2*), ****P = 2.49 × 10^−6^ (*Stat3*). (**F**) **P = 0.006 (*C1qa*), **P = 0.0001 (*C1qb*), **P = 0.0037 (*C1qc*), ****P = 3.99 × 10^−5^ (*C4b*), **P = 0.001 (*A2m*), ****P = 1.61 × 10^−6^ (*Serping1*). (**G**) ***P = 0.0006 (*Gfap*), ****P = 5.31 × 10^−5^ (*Vim*), *P = 0.021 (*Cd44*), ****P = 2.44 × 10^−7^ (*Serpina3n*), ****P = 1.61 × 10^−9^ (*Serping1*), ****P = 5.00 × 10^−8^ (*Cp*), **P = 0.002 (*Mt1*), ****P = 2.65 × 10^−5^ (*Mt2*), ****P = 2.49 × 10^−6^ (*Stat3*), ****P = 4.23 × 10^−13^ (*Lcn2*). (**H**) Disease and function enrichment analysis for all transcripts in TwKO^Astro^ retina. Ingenuity Pathway Analysis bubble plot (FDR < 0.1). Data represent mean ± SEM. Control *n* = 4, TwKO^Astro^ *n* = 5. The *P* values calculated by the Wald test underwent multiple testing correction by the Benjamini and Hochberg method to get PDR values. Source data are available online for this figure.

**Figure EV5:**
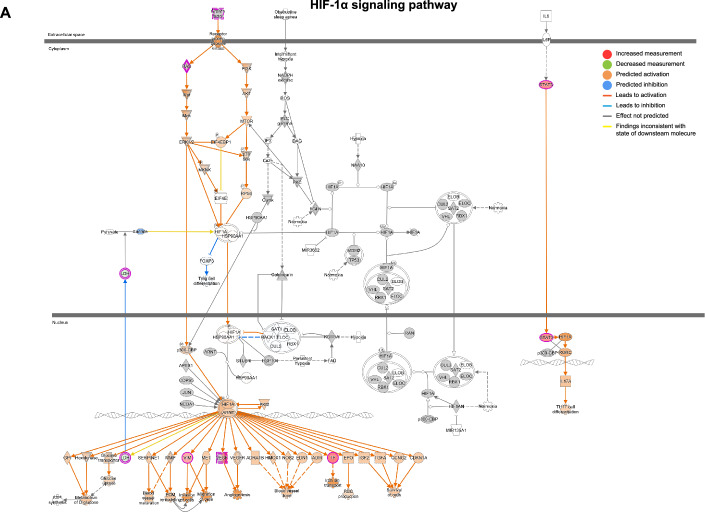
HIF-1α pathway. (**A**) HIF-1α signaling pathway. Highlighting differentially expressed genes in TwKO^Astro^ retina; Ingenuity Pathway Analysis of RNA sequencing data.

To gain insight into the molecular pathways in the TwKO^Astro^ retina, we carried out bulk RNA sequencing from isolated whole retina dissected from 4.5- to 5-month-old TwKO^Astro^ and control mice. Even though Müller glia cells and astrocytes make up 3–5% of all cells in the retina, due to the large cell bodies of Müller glia, they contribute to approximately ~30% of the total retina transcriptome (Pauly et al, 2019). Our differential expression analysis identified 178 differentially expressed genes (122 upregulated, 56 downregulated, with log_2_FC > 0.5, FDR < 0.1), and a principal component analysis demonstrated separate clustering of TwKO^Astro^ and control datasets (Fig. 5A,B). The affected pathways highlighted inflammation (11/20 of most significantly upregulated transcripts), translation, gliosis and angiogenesis (Fig. 5B–F; Table 1. The most activated upstream regulators included multiple cytokines, interferon alpha family, and complement *C1q* (Table EV1). The overlay of gene expression results over the complement system pathway showed that the classical pathway of the complement cascade is activated (Fig. 5D). Further, functional enrichment analysis supported affected angiogenesis and vasculogenesis as well as reactive gliosis (Fig. 5E,G,H). Overlaying the gene expression results onto the HIF1α signaling pathway indicated hypoxia-independent angiogenesis in TwKO^Astro^ retina (Fig. EV5A).

**Table 1:**
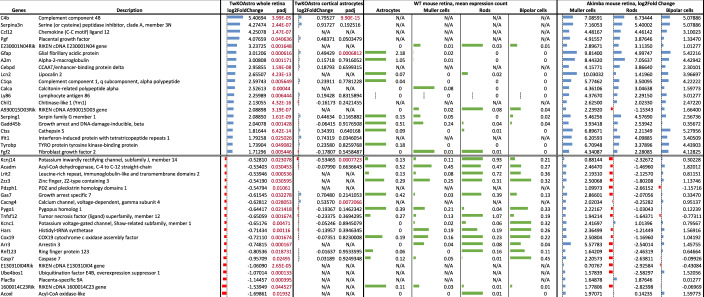
Main differentially expressed transcripts in the retina of mice with macroglial Twinkle-knockout, TwKO^Astro^, compared with the cortex of TwKOAstro, as well as normal mouse retina and the Akimba mouse dataset.

Akimba: hyperglycemic mice with retinopathy (*Ins2* mutant Akita crossed to the Kimba mouse, VEGF + /+).

To better understand the potential molecular mechanisms underlying the retinal pathology phenotype, we next focused on individual differentially expressed genes with known or suggested roles in angiogenesis, immunity, and gliosis. We identified three secreted factors with angiogenic properties and potential contributors to the neovascularization phenotype of TwKO^Astro^ retinas (Fig. 5E); *Fgf2* (fibroblast growth factor 2; log_2_FC = 1.71 FDR = 0.0005), one of the most potent angiogenic factors; *Pgf* (placental growth factor; log_2_FC = 4, FDR = 0.04) that stimulates angiogenesis and recruits critical angiogenesis cells, and *Lcn2* (lipocalin-2, log_2_FC = 2,66, FDR = 4,23E-13); an inducer of retinal microangiopathy (Dong et al, 2019; Nguyen et al, 2018; Zhang et al, 2024). Several genes involved in the activation of the complement cascade, including the C1q complex (*C1qa*, *C1qb* and *C1qc*), *C4b*, *A2m* and *Serping1*, were upregulated (Fig. 5F). In agreement with the morphological assessment of gliosis in TwKO^Astro^ retinas, genes associated with gliosis were induced: *Gfap* (log_2_FC = 3, FDR = 0.00062), *Vim* (log_2_FC = 0.84, FDR = 5.31E-05), *Cd44* (log_2_FC = 1.46, FDR = 0.02), *Serpina3n* (log_2_FC = 4.27, FDR = 2.44E-07), *Serping1* (log_2_FC = 2, FDR = 1.61E-09), *Cp* (log_2_FC = 0.87, FDR = 5.00E-08), as well as potent antioxidants metallothioneins *Mt1* (log_2_FC = 0.53, FDR = 0.001) and *Mt2* (log_2_FC = 1.05, FDR = 2.65E-05), previously shown to be expressed predominantly by astrocytes in response to oxidative stress and neuroinflammation (West et al, 2008) (Fig. 5G).

To get information on the cell types from where the transcriptomic changes in our RNAseq originate, we compared the top-changed transcripts in TwKO^Astro^ retina to a previously published single-cell RNA sequencing dataset from normal mouse retina at postnatal day 14 (accession no. GSE63472; (Macosko et al, 2015)). Out of the 25 largest fold-change transcripts, nine were macroglia specific, despite the fact that macroglia represent a minority (5%) of the retinal cells (Table 1), suggesting that the main part of our RNAseq findings in retina originated from macroglia. We then compared the same list with 12-week-old Akimba mice, a model with hyperglycemia and retinal neovascularization (Rakoczy et al, 2010) (Data ref: accession no. E-MTAB-9061). The top-changed TwKO^Astro^ transcripts were induced in these hyperglycemic mice but showed little difference between different retinal cell types. However, the dataset was small (*n* = 2), *P* values could not be calculated, and remain suggestive (Table 1). Next, we compared an RNAseq dataset of TwKO^Astro^ cortical astrocytes (accession no. GSE174343) to TwKO^Astro^ whole-retina dataset (Ignatenko et al, 2022). Among the 40 most upregulated and downregulated genes in TwKO^Astro^ retina, only four genes exhibited similar changes in cortical astrocytes: *C4b*, *Gfap*, and the ion channels *Kcnj14* and *Cacng4* (Table 1). These data indicated that TwKO^Astro^-related macroglial/astrocytic reactivation in different nervous system sites causes different transcriptomic consequences.

### TwKO^Astro^ mice have normal visual sensitivity to light

**Figure 6:**
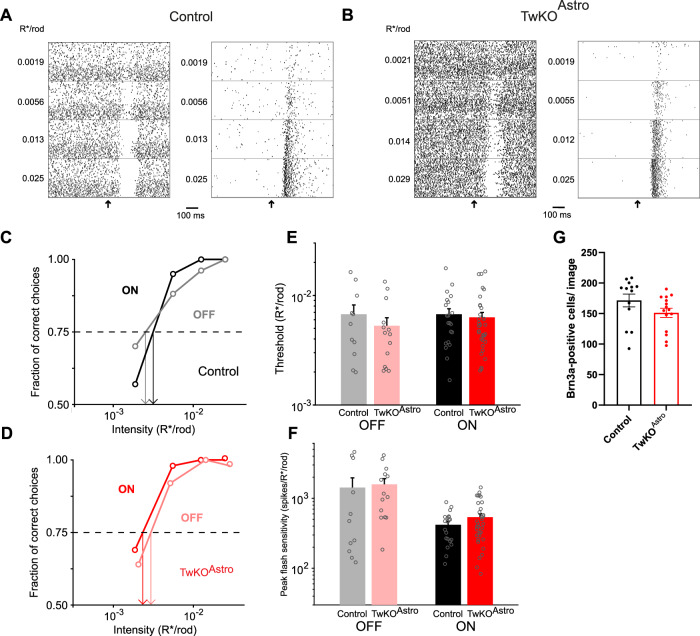
Functional ex vivo testing of retinal ganglion cells (RGCs) of TwKO^Astro^ mice. (**A**) Spike rasters showing responses of control ON sustained alpha retinal ganglion cells (RGC) (right) and OFF sustained alpha RGC (left) to a family of dim flashes (20-ms) delivered at the time of the arrow. Each raster shows 50 trials in response to flashes of a constant intensity indicated in the left (in R*/rod). (**B**) The same as (A) but for TwKO^Astro^ mice. (**C**) A two-alternative forced choice (2AFC) procedure to characterize RGC threshold sensitivity (0.75 fraction of correct choices) shown for the same control ON sustained alpha RGC (black) and OFF sustained alpha RGC (gray) RGC as in (**A**). (**D**) The same as in (**C**), but for TwKO^Astro^ ON sustained alpha RGC (red) and OFF sustained alpha RGC (pink) RGCs. (**E**) Population data on RGC thresholds (mean ± SEM) for control RGCs (OFF, 0.0067 ± 0.0014 R*/rod, *n* = 11 cells; ON, 0.0067 ± 0.0008 R*/rod, *n* = 21) and TwKO^Astro^ RGCs (OFF, 0.0053 ± 0.001 R*/rod, *n* = 14; ON, 0.0063 ± 0.0007 R*/rod, *n* = 33). No significant difference can be observed between control and TwKO^Astro^ strains in either ON (*P* = 0.70) or OFF (*P* = 0.40) alpha RGCs. (**F**) Peak flash sensitivities ( = response per photoisomerization; the highest flash sensitivity) for ON and OFF sustained alpha RGCs were 419 ± 45 spikes/R*/Rod and 1420 ± 540 spikes/R*/Rod, respectively, for control, and 539 ± 63 and 1580 ± 330 for TwKOAstro, respectively. Flash sensitivities were significantly different between ON and OFF RGCs for control (*P* = 0.015) and TwKO^Astro^ strains (*P* < 0.0001) but not between genotypes. Pairwise comparisons; *P* > 0.05, unpaired Welch’s *t* test. Data represent mean ± SEM. (**G**) Ganglion cell number assessment based on Brn3a immunofluorescent staining in whole-mount retina (Images in Fig. EV3; *P* = 0.13). Control *n* = 5, TwKO^Astro^ *n* = 5. Unpaired two-tailed parametric *t* test. Data represent mean ± SEM. Each dot represents an individual measurement. Source data are available online for this figure.

Given the retinal vascular manifestation in TwKO^Astro^ mice, we assessed their retinal output function by recording the spike outputs of the most sensitive retinal ON and OFF ganglion cell types (ON and OFF sustained alpha RGCs) ex vivo in dark-adapted flat-mounted mouse retinas. Figure 6A exemplifies spike rasters of OFF sustained alpha RGC (left) and ON sustained alpha RGC (right) at four increasing flash intensities in control mice. Figure 6B shows similar data on the same RGC types in TwKO^Astro^ mice. These flash intensities (from the lowest to highest) elicit only ~0.002 – 0.03 photoisomerizations per rod per flash. We used two-alternative forced choice (2AFC) ideal observer analysis to define the sensitivity limit of RGCs on these spike responses as previously described (Smeds et al, 2019). The detectability of the stimulus was quantified by computing the correlation between the mean response and each epoch during the interval before and after the flash. Figure 6C exemplifies detection performance for ON and OFF sustained alpha RGCs in control mice and Fig. 6D for TwKO^Astro^ mice. We defined the detection threshold for RGCs as the flash intensity giving 75% fraction of correct choices. Figure 6E shows population data on detection thresholds for ON and OFF sustained alpha RGCs for control mice and TwKO^Astro^ mice. No significant difference was observed between ON and OFF alpha RGCs in either control or TwKO^Astro^ mice. Furthermore, we also quantified the peak flash sensitivities (response strength divided by the light intensity) as another light sensitivity metrics of the RGCs (Fig. 6F). No significant differences were present in flash sensitivity between control and TwKO^Astro^ mice neither for ON nor OFF RGCs. Collectively, despite the major structural changes and leakage of vasculature, TwKO^Astro^ mice have similar retinal output sensitivity to light detection at visual threshold, probing the most sensitive readout of the rod signaling.

**Figure EV6:**
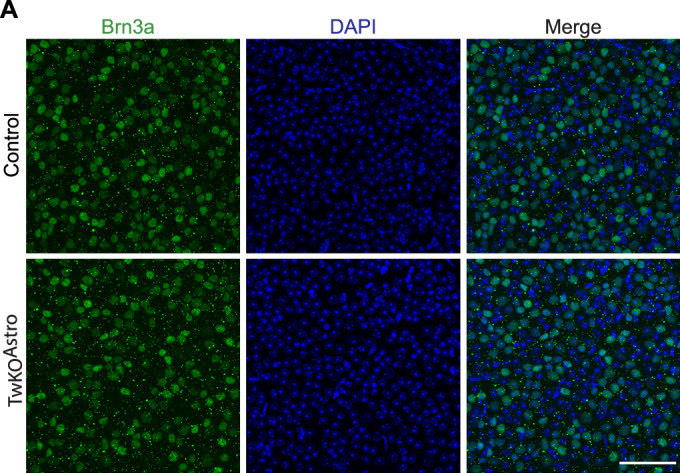
Retinal ganglion cells. (**A**) Brn3a immunofluorescent staining of whole-mount retinas of TwKO^Astro^ and control mice. Scale bar, 50 μm. Source data are available online for this figure.

To test whether retinal ganglion cells were affected in number, we counted them based on Brn3a immunofluorescent staining in whole-mount retinas (Fig. EV6). The cell amount showed a slight decreasing trend in TwKO^Astro^ mice, but this did not reach statistical significance (Fig. 6G).

## Discussion

We demonstrate that knockout of mtDNA helicase Twinkle in retinal astrocytes and Müller glia causes widespread reactive gliosis, pathological neovascularization, and leakage of retinal blood vessels. Our results indicate that Twinkle depletion, leading to progressive mtDNA loss and oxidative phosphorylation deficiency in this retinal cell population, does not kill the macroglia but disrupts vascular homeostasis. The profound vascular pathology was surprising, as the targeted macroglial cell population forms less than 5% of retinal cells, and the knockout was partial. Other mitochondrial disease models that also show respiratory chain deficiency did not have similar vascular changes, raising the possibility that mtDNA replisome-derived signaling in microglia has a specific role for vascular homeostasis in the retina. The upregulated angiogenic factors *Fgf2* (Fibroblast growth factor 2), *Pgf* (Placental growth factor), and retinal microvasculopathy inducer *Lcn2* (lipocalin-2) are candidates as causative factors, along with neuroinflammatory cytokines and components of the complement cascade. Similar pathways were not activated in the cerebral cortex, which despite remarkable astrogliosis showed intact vascular integrity and paravascular fluid flow. Our results highlight region-specific roles of astrocytes and demonstrate an essential role for mitochondrial signaling in macroglia in the maintenance of retinal vascular homeostasis.

The findings in TwKO^Astro^ retina resemble those in diabetic retinopathy (DR). The pathological signs of DR include microaneurysms, retinal hemorrhages, intraretinal microvascular abnormalities (non-proliferative DR), and pathological retinal neovascularization (proliferative DR), accompanied by vascular leakage (Stitt et al, 2016). Retinal astrocytes and Müller glia have been reported to reactivate in DR, along with a chronic inflammatory environment, which is associated with the progression of diabetic complications (Krügel et al, 2011; Pfaller et al, 2024; Yang et al, 2020), but the effect has been considered secondary. Moreover, microglia activation, complement factor response, and inflammation are important pathological mechanisms of proliferative DR (Tang and Kern, 2011). Intriguingly, our data on TwKO^Astro^ mice show that all the signs and symptoms of DR can be caused by reactivation of retinal macroglia. Furthermore, TwKO^Astro^ retina shows robust increases of established molecular factors contributing to DR, such as Fgf2 and Pgf, A2m, B2m, Serping1, Clu, complement factors C1q, C3, C4b and candidate DR biomarkers such as Edn2, Pgf, Lcn2 and Ctss (Binz et al, 2016; Grant et al, 2004; Hill et al, 1997; Kaštelan et al, 2020; Nguyen et al, 2018; Youngblood et al, 2019; Yuan et al, 2023; Zhang et al, 2024). To conclude, Twinkle and mtDNA depletion in retinal macroglia cause a neovascular retinopathy with the morphological, functional, and molecular characteristics of DR. The findings offer a new, previously underappreciated target for neovascular retinopathy interventions: mitochondrial metabolism in retinal macroglia.

RGC loss and optic nerve atrophy occur often in primary mitochondrial diseases as well as in DR (Finsterer et al, 2018; Potilinski et al, 2020). The TwKO^Astro^ mice allow exploration of the vasculopathy separately from RGC loss, pointing to partially different pathogenic signaling. At the time of vascular leakage, the number of retinal ganglion cells of TwKO^Astro^ mice was control-like, and recordings of single ON or OFF RGCs from whole-mount retina showed wild-type-like light responses. These findings suggest that the most sensitive retinal rod pathway functions normally in these mice. Our findings support a model where macroglia-specific mtDNA loss autonomously leads to their reactivation and is followed by non-cell autonomous microgliosis, vascular endothelial cell proliferation, and fragility of vessel walls, while RGC survival and physiology are still intact at this disease stage.

The activation of the HIF1alpha pathway suggests that the loss of Twinkle and/or mtDNA in retinal macroglia causes a pseudohypoxic response. When oxidative phosphorylation is disabled, pseudohypoxic signaling can induce vascular proliferation, which paradoxically can lead to hyperoxic damage. Such mechanism has been observed in the brain stem of a mouse models of Leigh disease, Ndufs4KO, with respiratory chain complex I deficiency (Kruse et al, 2008). Indeed, hyperoxia causes neovascular retinopathy in preterm newborns with bronchopulmonary dysplasia (Lajko et al, 2016). This previous literature together with our data suggest that astrocytic mitochondria participate in hypoxia-sensing or initiate a pseudohypoxic signal, consequently triggering angiogenesis in the retina. In neovascular retinopathy and age-related macular degeneration, the major angiogenic growth factor VEGF-A is activated by hypoxia (Osaadon et al, 2014). Furthermore, partial ablation of Müller glia in mice was reported to cause reactivation of the remaining Müller glia, VEGF-A activation, and consequent neovascularization, blood-retina-barrier breakdown, microglia activation, and photoreceptor apoptosis (Shen et al, 2012). While VEGF-A was not induced in TwKO^Astro^ mice, FGF2 and PGF, two potent drivers of pathological angiogenesis in humans and experimental models (Dong et al, 2019; Nguyen et al, 2018; Rusnati and Presta, 2007; Zubilewicz et al, 2001) were. Anti-VEGF-A therapies are the current standard of care for a majority of neovascular retinal pathologies, including DR. However, many patients are non-responders, supporting involvement of additional pathological processes (Simó and Hernandez, 2008). Combination therapies of FGF2 or PGF with anti-VEGF therapy were reported to be superior at reducing ocular angiogenesis and vascular leakage compared to the single VEGF blockage (Balser et al, 2019; Stahl et al, 2009), which aligns well with our data of FGF2 and PGF contribution in pathogenesis. Taken together, our findings propose that pro-angiogenic factors FGF2 and PGF contribute to retinal neovascularization caused by mitochondrial-derived metabolic signaling by macroglia.

We demonstrate that the key complement components that originate primarily from macroglia (C1s, C4) were upregulated, as were the microglial-originating C1q, while the membrane attack complex components (C5–C9,) primarily released by retinal pigment epithelium were not changed. Complement cascade has previously been shown to be activated in different retinal pathologies associated with inflammation and neovascularization, including retinopathy of prematurity, age-related macular degeneration, and DR (Garcia-Ramirez et al, 2007; Gerl et al, 2002; Natoli et al, 2017; Rathi et al, 2017). Our results indicate that mtDNA depletion in retinal macroglia is sufficient to activate the complement cascade in the whole retina.

In conclusion, our data highlight the role of macroglial mitochondria in regulating retinal vascular homeostasis and their intriguing potential roles in neovascular retinopathy, with intriguing similarity to that of diabetic retinopathy. Our findings have important implications for targeting macroglia in therapy development for rare and common metabolic retinopathies manifesting with gliosis, leaky blood-retina barrier, and inflammation.

### Limitations of the study

As the general well-being of TwKO^Astro^ mice declines after 5 months of age because of a severe progressive neurodegenerative disease, we could not assess how the RGC light responses developed over time. Bulk RNASeq over scRNASeq was preferred for the transcriptomics analysis to achieve better sequencing depth from the low-frequency cell populations of interest, Müller glia and astrocytes (total 5%, astrocytes 0.5%), and also to identify non-autonomous effects of the astrocytic TwKO for the whole retina. This approach does not, however, identify astrocyte-specific changes. Finally, the retina is a highly complex tissue with a large number of cell types interacting to maintain its delicate homeostatic state. Even with a low percentage of macroglia undergoing depletion of Twinkle helicase with the GFAP-Cre line used in this study, we observe vascular pathology of the retina, although in a qualitative manner. Future use of different macroglial-directed CRE strains holds the potential to reveal a quantitative relationship between macroglial mtDNA maintenance defects and retinal pathology. While secondary changes in other cell types, which do not express CRE-recombinase, can contribute to the pathogenesis, the primary origin of the phenotype is the knockout of Twinkle in astrocytes. The other mouse models with mitochondrial respiratory chain defects that lacked vasculopathy were ubiquitously expressing the genetic modification, differing from the current model targeting only astrocytes/macroglia. Therefore, we cannot fully exclude the possibility that the lack of vasculopathy in other models could be caused by systemic signaling factors affecting tissue homeostasis.

## Methods

**Table:** Reagents and tools table

Reagent/resource	Reference or source	Identifier or catalog number
**Experimental models**		
TwKO^Astro^ mouse	Ignatenko et al (2022)	
Deletor mouse	Tyynismaa et al (2005)	
Mutator mouse	Tyynismaa et al (2005)	
Ndufs4KO mouse	Kruse et al (2008)	
Gfap73.12-Cre:LSL-tdTomato mouse	This study	
B6.Cg-Gt(ROSA)26Sortm14(CAG-tdTomato)Hze/J reporter mouse	The Jackson Laboratory	Strain #:007914
**Recombinant DNA**		
**Antibodies**		
Rabbit anti-aquaporin 4	EMD Millipore Corp.	cat#AB3594
Rabbit anti-Brn3a	Synaptic Systems	cat#411003
Rat anti-CD68	Bio-Rad	cat#MCA1957
Mouse (IgG2a) anti-ds DNA	Abcam	cat#ab27156
Rabbit anti-fibrinogen	Agilent Technologies	cat#A008002-2
Rabbit anti-GFAP polyclonal antibodies	Sigma-Aldrich	cat#AB5804
Rabbit anti-IBA1	Synaptic Systems	cat#234003
Rabbit anti-IBA1	Wako Chemicals	cat#019 19741
Primary goat anti-podocalyxin	R&D Systems	cat#AF1556
Rabbit anti-Sox9	Millipore	cat#AB5535
Streptavidin-conjugated Alexa Fluor 488 antibodies	Invitrogen	cat#S-32354
Donkey anti-rabbit Alexa-fluor 594	Invitrogen	cat#A-21207
Goat anti-rat Alexa fluor 633	Invitrogen	cat#A-11007
**Oligonucleotides and other sequence-based reagents**		
**Chemicals, enzymes, and other reagents**		
Biotinylated *Griffonia simplicifolia* Lectin I Isolectin B4	Vector Laboratories	cat#B-1205
Vectashield mounting medium with DAPI	Vector Laboratories	cat#H-1200-10
Hoechst	Thermo Fisher Scientific	cat#62249
Fluorescent CSF tracers FITC-dextran 3000	Invitrogen	cat#D3306
Ovalbumin-conjugated Alexa-647	Invitrogen	cat#O34784
TRIzol reagent	Invitrogen	cat #15596-018
**Software**		
Fiji/ImageJ		https://fiji.sc/
CellProfiler	Lamprecht et al (2007)	
Angiotool	Zudaire et al (2011)	
GraphPad Prism	GraphPad Software, Boston, MA, USA	www.graphpad.com
**Other**		

### Mouse models

The animals were raised and handled at the Laboratory Animal Center of the University of Helsinki, Finland. All mouse experiments were carried out in accordance with the guidelines of the European Community Council Directive 2010/63/EU and the Finnish Act on the Protection of Animals Used for Scientific or Educational Purposes (497/2013). The study protocol was reviewed and approved by the Finnish Animal Experiment Board (ELLA) and authorized by the Regional State Administrative Agency (AVI) under license number ESAVI/37265/2024. All efforts were made to minimize animal suffering, and humane endpoints were applied. The study is reported in accordance with the ARRIVE guidelines. Investigators were blinded to group allocation during the experiments and outcome assessment.

Mice were maintained in a temperature-controlled facility with a 12-h light/dark cycle and ad libitum access to regular mouse chow and drinking water. Animal experiments were conducted according to the guidelines approved by the Finnish Committee of Experimental Animal Research. The following mouse lines were used: the astrocyte-specific Twinkle knockout mouse (TwKO^Astro^), Deletor mice expressing a dominant duplication of amino acids 353–365 in mouse Twinkle protein, Mutator mice carrying the substitution of threonine for alanine at the amino acid 360 of mouse Twinkle protein, Ndufs4KO mice lacking a mitochondrial complex I subunit and Gfap73.12-Cre:LSL-tdTomato mice, which were created by crossing Gfap73.12-Cre (TwKO^Astro^) and B6.Cg-Gt(ROSA)26Sortm14(CAG-tdTomato)Hze/J reporter lines (Ignatenko et al, 2018; Ignatenko et al, 2022; Kruse et al, 2008; Kujoth et al, 2005; Trifunovic et al, 2004; Tyynismaa et al, 2005). The TwKO^Astro^ mice at 2 weeks or 4–5 months of age, Deletor mice at 16–27 months of age, 12-month-old Mutator mice, 50-day-old Ndufs4KOs, 1–2 weeks old Gfap73.12-Cre:LSL-tdTomato. Littermates were used as controls. Only males were used for TwKO^Astro^ and Deletor mouse models, as they have previously shown sensitivity to early mitochondrial phenotype (Deletor). In our previous work on TwKOastro, no gender differences existed for cerebral spongiotic phenotype [16]. Mixed-gender cohorts were used for Mutator, Ndufs4KO and Gfap73.12-Cre:LSL-tdTomato mice. For these mice, no significant gender-specific phenotypes have been reported(Ignatenko et al, 2018).

The mice were either euthanized with CO_2_ or terminally anesthetized by intraperitoneal injection of pentobarbital followed by transcardial perfusion with 4% paraformaldehyde (PFA). Both perfused and non-perfused brains and eyeballs were fixed overnight in 4% PFA in PBS at 4 °C. Then the tissues were stored in PBS with 0.02% sodium azide at 4 °C and immersed into 30% sucrose in PBS for several days prior to freezing. Prior to sectioning, the tissues were embedded in OCT Compound Embedding Medium (Tissue-Tek).

### Immunohistochemistry

The brains and eyeballs were cryosectioned (Leica CM3050 S Research Cryostat; Leica) at 100-, 20-, and 12-μm sections on glass slides (Superfrost plus; Thermo Scientific) and stored at −20 °C. The following primary antibodies were used in this study: primary goat anti-podocalyxin (1:100; RD systems; cat#AF1556), rabbit anti-aquaporin 4 (1:100; EMD Millipore Corp.; cat#AB3594), rabbit anti-GFAP polyclonal antibodies (1:500; Sigma-Aldrich; cat#AB5804), rabbit anti-fibrinogen (1:300; Agilent Technologies; cat#A008002-2), rabbit anti-IBA1 (1:500; Synaptic Systems; cat#234003), rabbit anti-Brn3a (1:500; Synaptic Systems; cat#411003), mouse (IgG2a) anti-ds DNA (1:600; Abcam; cat#ab27156), rabbit anti-Sox9 (1:1000; Millipore; cat#AB5535). Corresponding secondary antibodies with appropriate fluorescent dyes were used at 1:500 dilution (Invitrogen). The tissues were nuclear counterstained with Hoechst and covered with Vectashield mounting medium with DAPI (Vector Laboratories; cat#H-1200-10). Images were acquired with a fluorescence confocal microscope (Zeiss LSM 780 or ANDOR Dragonfly 505 with 60x objective Plan Apo VC; NA 1.2).

For whole-mount staining, the retinas were dissected in cold PBS. For the vascular staining, they were pre-incubated in PBlec (0.1 mM CaCl2, 0.1 mM MgCl_2_, 0.1 mM MnCl_2_, and 1% Triton X-100 in PBS, pH 6.8) for 30 min and in blocking solution (1% BSA 0.5% Triton X-100 in PBS) for 1 h at RT. Then they were incubated with biotinylated *Griffonia simplicifolia* Lectin I Isolectin B4 (IB4; 1:25; Vector Laboratories; cat#B-1205) diluted in PBlec, overnight at 4 °C. IB4 was detected by incubation with streptavidin-conjugated Alexa Fluor 488 antibodies (1:200; Invitrogen; cat#S-32354) overnight at 4 °C. For the other stainings, the retinas were incubated with primary antibodies (rabbit anti-IBA1 (1:500; Wako Chemicals; cat#019 19741), rat anti-CD68 (1:40; Bio-Rad; cat#MCA1957), diluted in blocking solution overnight at 4 °C (RT for Brn3a staining), and then with the corresponding secondary antibodies overnight at 4 °C (1:800 dilution for donkey anti-rabbit Alexa-fluor 594 for IBA1 (Wako), 1:150 dilution for goat anti-rat Alexa fluor 633 for CD68). The retinas were postfixed with 1% PFA. The tissues were nuclear counterstained with Hoechst and covered with Vectashield mounting medium with DAPI (Vector Laboratories; cat#H-1200-10). Images were acquired with a fluorescence confocal microscope (Zeiss LSM 780 or ANDOR Dragonfly 505 with ×40 objective Apo LWD; NA 1.2).

### Image analysis and statistical analysis

**Figure EV7:**
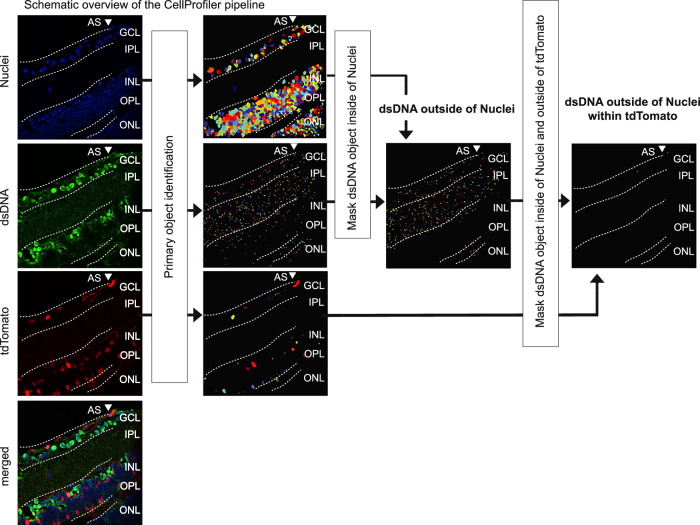
Schematic overview of the CellProfiler pipeline for mtDNA quantification within tdTomato-positive retinal astrocytes. Each stained object was identified and outlined (nuclei, DAPI, blue; dsDNA, green, both in nucleus and cytoplasm (mtDNA); Tomato indicating cells with cre-activity), the dsDNA signal within the nucleus was masked and removed, and the remaining cytoplasmic dsDNA signal within the tdTomato positive region was quantified. The white dotted line delineates the boundary between retinal layers. Source data are available online for this figure.

For the quantification of confocal images, each pixel of the images was classified into GFAP-Cre tdTomato (tdTomato positive), mtDNA (dsDNA positive outside of the nuclei area), Sox9 (Sox9-positive Müller cells), GFAP (GFAP-positive astrocytes), COX-I protein (COXI positive spots). All the pixels were quantified by CellProfiler 4.2.8 software (McQuin et al, 2018). Schematic description of the CellProfiler pipeline is represented in Fig. EV7. Comparisons between two groups were performed by an unpaired two-tailed Student’s *t* test. Multi-comparisons were performed by two-way ANOVA following Tukey’s multiple comparisons test. The *P* value < 0.05 was considered to be statistically significant. Statistical parameters were reported either in individual figures or corresponding figure legends. All statistical analyses were performed using Prism 10.6.0 Software (GraphPad Software).

### Blood vessel tracing

To visualize the retinal vascular network, the mice were anesthetized by intraperitoneal injection of pentobarbital, and 2.5% FITC-dextran (10.000 MW; Invitrogen; cat#D1820) was injected into a tail vein. After 30 min, the mice were sacrificed with CO_2_. The brains and eyeballs were fixed overnight in 4% PFA in PBS at 4 °C.

### Intracisternal tracer injection

Mice were anesthetized via intraperitoneal administration of a mixture of ketamine and xylazine (100 mg/kg and 10 mg/kg, respectively) and fixed in a stereotactic frame; a 30 G needle was inserted into the cisterna magna. For intracisternal injections, 10 µl of fluorescent CSF tracer was injected at a rate of 1 L/min for 10 min with a syringe pump. The fluorescent CSF tracers FITC-dextran 3000 and ovalbumin-conjugated Alexa-647 (Invitrogen) were constituted in artificial CSF at a concentration of 0.5%. Thirty minutes after the injection, the mice were euthanized by decapitation, and the brains were fixed by immersion in 4% PFA. Brains were post-fixed overnight in 4% PFA. In all, 100-μm coronal slices were acquired using a cryotome (Leica CM3050 S Research Cryostat; Leica), mounted with Vectashield mounting medium with DAPI (cat#H-1200-10, Vector Laboratories), and imaged by epifluorescence microscope (Zeiss Axio Imager). To quantify CSF tracer distribution in the brain, six slice images per animal were analyzed in ImageJ software (Iliff et al, 2012). Shortly, a whole-slice region of interest (ROI) was determined for each color channel separately based on the DAPI channel. The background was subtracted, and the mean fluorescence intensity was calculated. The pixel intensity was adjusted to a threshold of 50/255 and 20/255 for FITC and TR channels, respectively. The area above the threshold was expressed as % from overall area of a brain slice.

### AngioTool quantification

Quantitative analysis of vascular networks in the retina and brain was carried out semiautomatically using AngioTool (Zudaire et al, 2011). This software computes several parameters that characterize vessel organization and sprouting activity. A set of three 800 × 800 pixels square images of the retina or four 1200 × 1200 pixels square images of the cortex were randomly selected in Zen Blue on a Z-stacked image of a flat-mounted retina or longitudinal cortex section. To analyze the layers of retinal vasculature, confocal z-stacks were acquired for each layer, maximum intensity projections were created, and further processed in AngioTool. The layers of retinal vasculature were distinguished from one another by their depth and vessel morphology. Vessel area values were divided by 1000 to improve figure readability.

### Histology

The eyeballs were collected and post-fixed in 4% PFA, followed by embedding in paraffin. The hematoxylin-eosin staining was carried out on 8-μm sections.

### RNA isolation

TwKO^Astro^ mice and their age-matched control mice were sacrificed with CO_2_, and the eyes were enucleated. The retinas were dissected and snap-frozen on dry ice and stored at −80 °C until use. Total retina RNA was extracted according to the manufacturer’s protocol following homogenization of retinal tissues in the presence of TRIzol reagent (Invitrogen; cat #15596-018). RNA was analyzed using TapeStation, and only samples with RIN > 7 were used for the sequencing.

### RNA sequencing

The RNA sequencing of the retinas was carried out by the Biomedicum Functional Genomics Unit, University of Helsinki (the Helsinki Institute of Life Science, HiLIFE, and Biocenter Finland). A “Bulkseq” 3’UTR counting gene expression profiling method was utilized to carry out RNA sequencing, as in (Freitag et al, 2020). Shortly, mRNA was primed with an oligo dT primer, which contains both sample code and unique molecular identifier sequence (UMIs). Using the template switch, single-stranded cDNA was converted to double-stranded and subsequently amplified using a set of SMART PCR primers. Next, the samples were pooled together, and the Dropseq P5 and Nextera i7 primers were used to create PCR sequencing pools. RNA sequencing was performed on the NextSeq 500 (NextSeq High Output 75 cycle flow cell). Bcl2fastq2 conversion software was used to produce FASTQ from BCL files and demultiplex the samples. The Trimmomatic program was utilized for adapter sequence trimming and sorting out the reads shorter than 20 nt. PolyA tails equal or longer than 6 were removed, and the reads were aligned to the mouse (mm10) reference genome using STAR aligner. The duplicates were removed based on UMIs.

Differential expression analysis for both RNA sequencing experiments was carried out with the DESeq2 Bioconductor R package(Love et al, 2014). The plots were created in RStudio (version 2023.03.0) using ggplot, PlotPCA, and EnhancedVolcano packages. Gene Ontology (GO) analysis was performed using the PANTHER system(Thomas et al, 2022) and Ingenuity Pathway Analysis Qiagen Ingenuity Pathway Analyses (Qiagen; https://digitalinsights.qiagen.com/IPA). GO terms with a false discovery rate (FDR) < 0.05 were considered significantly altered.

### Reanalysis of previously published single-cell data

Publicly available single-cell RNA-sequencing data were obtained from (Van Hove et al, 2020) via the ArrayExpress repository (accession E-MTAB-9061). The dataset comprises 10x Genomics 3′ Gene Expression v3 libraries generated from retinas of wild-type and Akimba (*Ins2*^Akita × *Vegfa*^+/–) mice (Van Bergen et al, 2017).

Downloaded FASTQ files were processed using Cell Ranger Count v9.0.1 (10x Genomics). Reads were aligned to the *Mus musculus* reference genome (GRCm39, 10x Genomics release 2020-A) with the STAR aligner (v2.7.x) (Dobin et al, 2013) integrated within the Cell Ranger pipeline. The pipeline performed barcode processing, UMI collapsing, cell calling, and generation of filtered gene-by-barcode matrices following default 10x Genomics algorithms.

Subsequent analyses were conducted in R v4.x using Seurat v5.2.1 (Hao et al, 2021). Low-quality barcodes and potential multiplets were excluded by filtering out cells expressing fewer than 200 or more than 8000 genes, fewer than 400 unique molecular identifiers (UMIs), or with >10% mitochondrial RNA content. After filtering, 7327 cells were retained for downstream analysis, including 5582 control and 1745 Akimba cells.

Normalization, scaling, and clustering were performed in Seurat using PCA followed by UMAP for visualization. Clusters were annotated based on canonical retinal marker genes: *Rlbp1*, *Aqp4*, and *Gfap* for Müller glia; *Rho* and *Pde6b* for rod photoreceptors; and *Vsx2* and *Grm6* for bipolar cells, guided by expression patterns described in (Van Hove et al, 2020).

For exploratory comparison of rod photoreceptors between the Akimba and control samples, pseudo-bulk average expression values were calculated by aggregating all identified rod cells per condition. Log₂ fold changes were derived as the difference between mean normalized expression values in Akimba and control rods. Due to the absence of biological replicates, no statistical testing was performed; reported values represent descriptive fold changes only.

### Ganglion cell recordings

Ex vivo retinal preparations were harvested from dark-adapted control mice and in TwKO^Astro^ mice. All ganglion cell recordings were done from flat-mounted preparations as previously described (Koskela et al, 2020; Smeds et al, 2019; Westö et al, 2022). Briefly, dark-adapted mice were sacrificed by cervical dislocation, their eyes were enucleated, and the vitreous was removed. The eye cups were stored in a light-tight container at 32 °C in oxygenated (95% O_2_/5% CO_2_) Ames solution (Sigma, A-1420; osmolality adjusted to 280 ± 2 mOsm/kg). The whole retina was gently isolated from the pigment epithelium and placed on a poly-D-lysine coverslip (12 mm; VWR, Corning) with the photoreceptor side down. The mounted retina was then placed on a recording chamber and transferred to the microscope and perfused with Ames solution (32 ± 1 °C, flow rate: 8 ml/min). These and all subsequent procedures were performed under IR light (> 900 nm) using night vision goggles (PVS-7-1600, B.E. Meyers) and IR pocket scopes (D7200-I-1600, B.E. Meyers) attached to the dissection microscope. The preparations were visualized using IR light (940 nm; turned off during recordings) and a CCD camera (Wat-902HS, Watec) attached to the microscope.

The recordings were made from ON and OFF sustained alpha retinal ganglion cells. The spiking activity of the cells was measured with the cell-attached patch clamp technique in response to a sequence of 20–50 short flashes of various intensities in darkness. The RGC types were identified based on the large soma size and their characteristic light response. In a subset of experiments, the dendritic morphology of cells was verified by filling the cells with a fluorescent dye (HiLyte Fluor 750 hydrazide, AnaSpec, AS-81268) and by imaging the cells (Andor Zyla 4.2 PLUS sCMOS) following fluorescence excitation (peak at 740 nm; width 35 nm, CoolLED pE-4000, CoolLED). The cell morphology was confirmed to be consistent with ON-S and OFF-S alpha RGCs.

For the stimulus detection, calibrated spatially uniform flashes (20-ms in duration, circular spot, 580 mm in diameter) centered on the target cell were used from a blue LED (peak at 470 nm). Stimulus intensities were set using neutral density filters and by controlling the current driving the LEDs. Light intensities were calibrated with an optometer (S450 with the sensor 268 R, UDT Instruments), and the spectrum was measured with a spectrometer (Jaz spectrometer, OceanOptics). Calibrated photon fluxes were converted to photoisomerizations per rod per second (R*/rod/s) based on mouse rhodopsin absorption spectrum (Koskela et al, 2020).

We used two-alternative forced choice (2AFC) ideal observer analysis to define the sensitivity limit of RGCs (Smeds et al, 2019). We computed a correlation of the pre- and post-flash firing rates against an average flash response (discriminant). The average flash response was computed across all other epochs except for the one under examination. The choice was assigned based on the higher correlation value. We defined the intensity giving rise to 75% correct choices in the task as the sensitivity threshold for RGCs.

### Statistical methods

Method-specific statistics are described under relevant headings. Statistical significance was determined in GraphPad Prism software 8. The level of significance was set at *P  <  0.05, **P  <  0.01, ***P  <  0.001. All the graphs, with the exception of Fig. 6, were created using GraphPad Prism software 8. Figure 6 was generated using MATLAB. All values are expressed as mean ± standard error of the mean (SEM).

### Graphics

Synopsis image created using BioRender https://www.biorender.com/.

## Supplementary information

Table EV1

Peer Review File

Source data Fig. 1

Source data Fig. 2

Source data Fig. 3

Source data Fig. 4

Source data Fig. 5

Source data Fig. 6

Figure EV1 Source Data

Figure EV2 Source Data

Figure EV3 Source Data

Figure EV4 Source Data

Figure EV6 Source Data

Figure EV7 Source Data

Expanded View Figures

## Data Availability

The sequencing data generated in this study have been deposited in the European Nucleotide Archive (ENA) and are available under the accession number PRJEB83104. The source data of this paper are collected in the following database record: biostudies:S-SCDT-10_1038-S44321-026-00438-0.
