# Supplementary figures and images for: Loss of mitochondrial DNA helicase in retinal macroglia drives neovascular retinopathy (part 1 of 2)
Source: EMBO Mol Med. 2026 May 8;18(7):2573–98. doi: 10.1038/s44321-026-00438-0 (PMC13365537; doi:10.1038/s44321-026-00438-0)

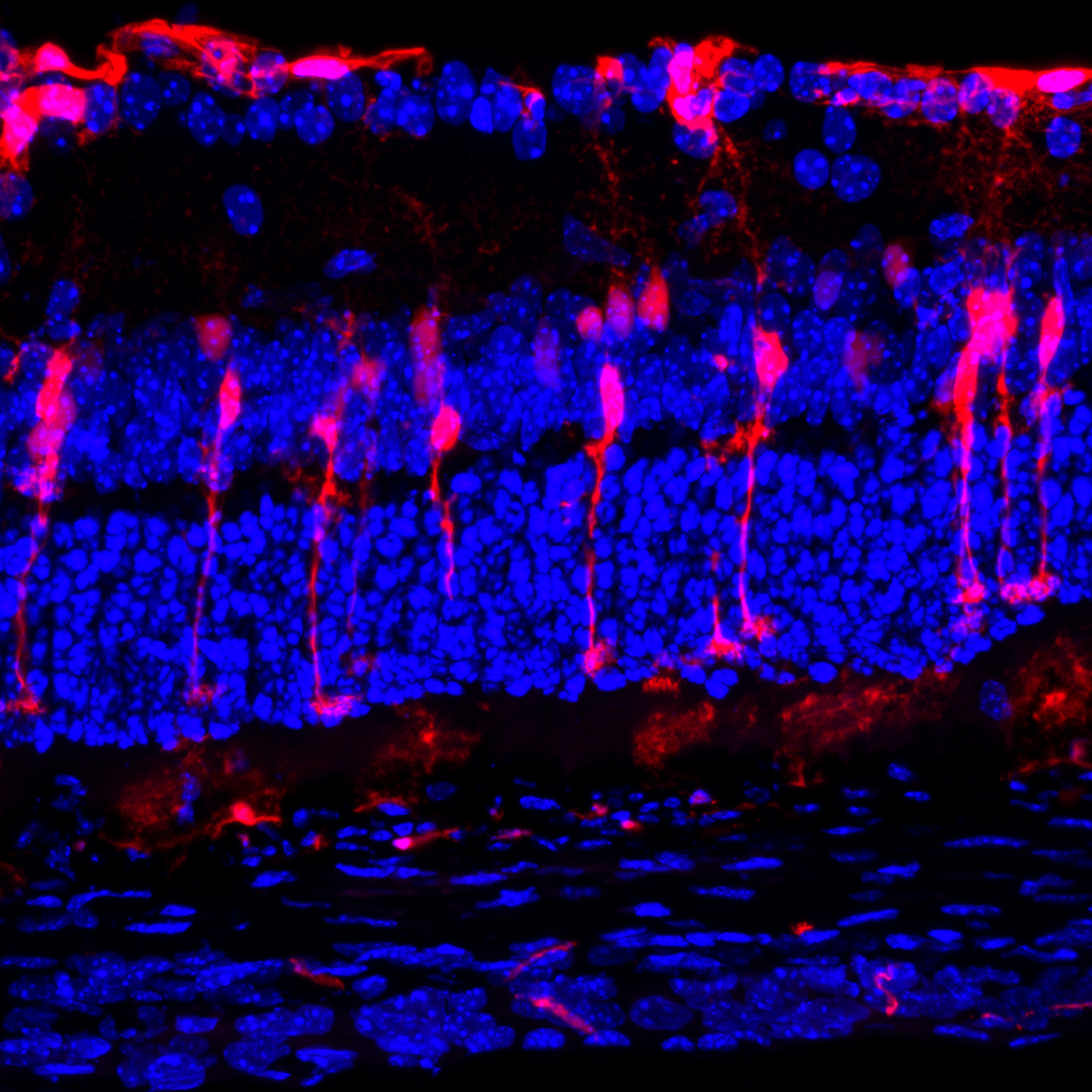

Supplement: Supplementary file 3 — Source data Fig. 1 [file 44321_2026_438_MOESM3_ESM.zip › Figure 1/1A/OIM1636BC-pup3-3_merge_cropped.tif]

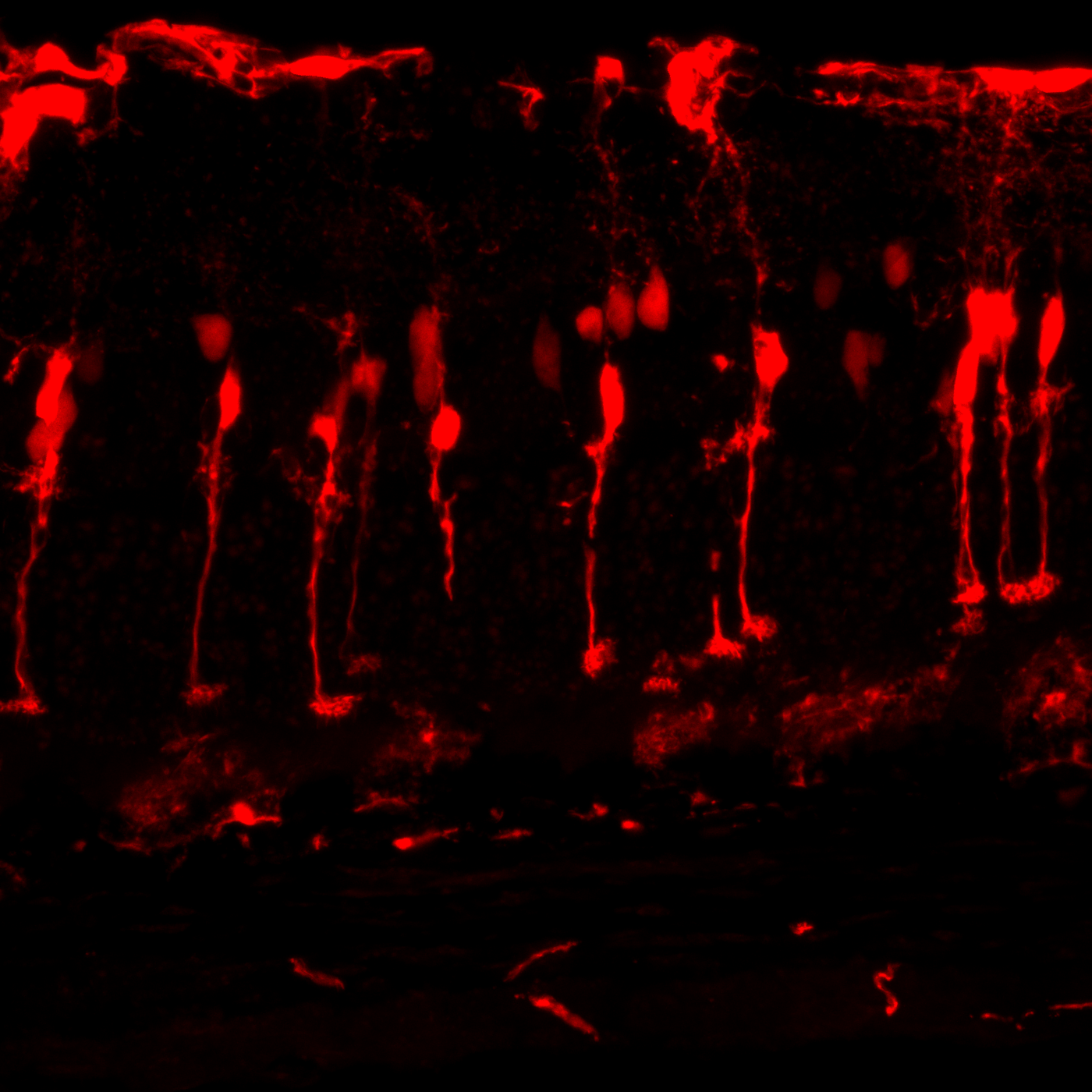

Supplement: Supplementary file 3 — Source data Fig. 1 [file 44321_2026_438_MOESM3_ESM.zip › Figure 1/1A/OIM1636BC-pup3-3_tomato_cropped.tif]

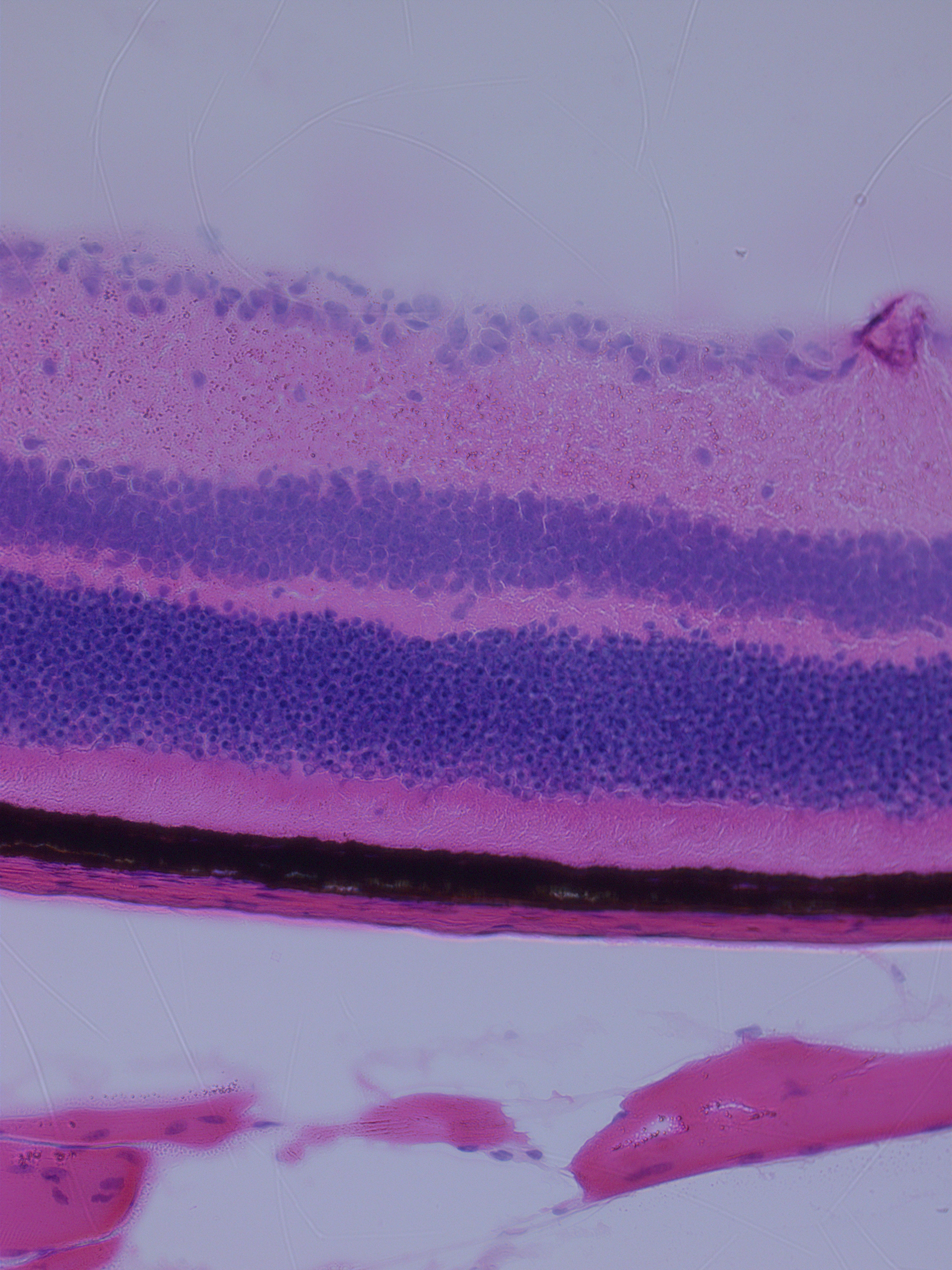

Supplement: Supplementary file 3 — Source data Fig. 1 [file 44321_2026_438_MOESM3_ESM.zip › Figure 1/1B/Ctrl-3 copy.tiff]

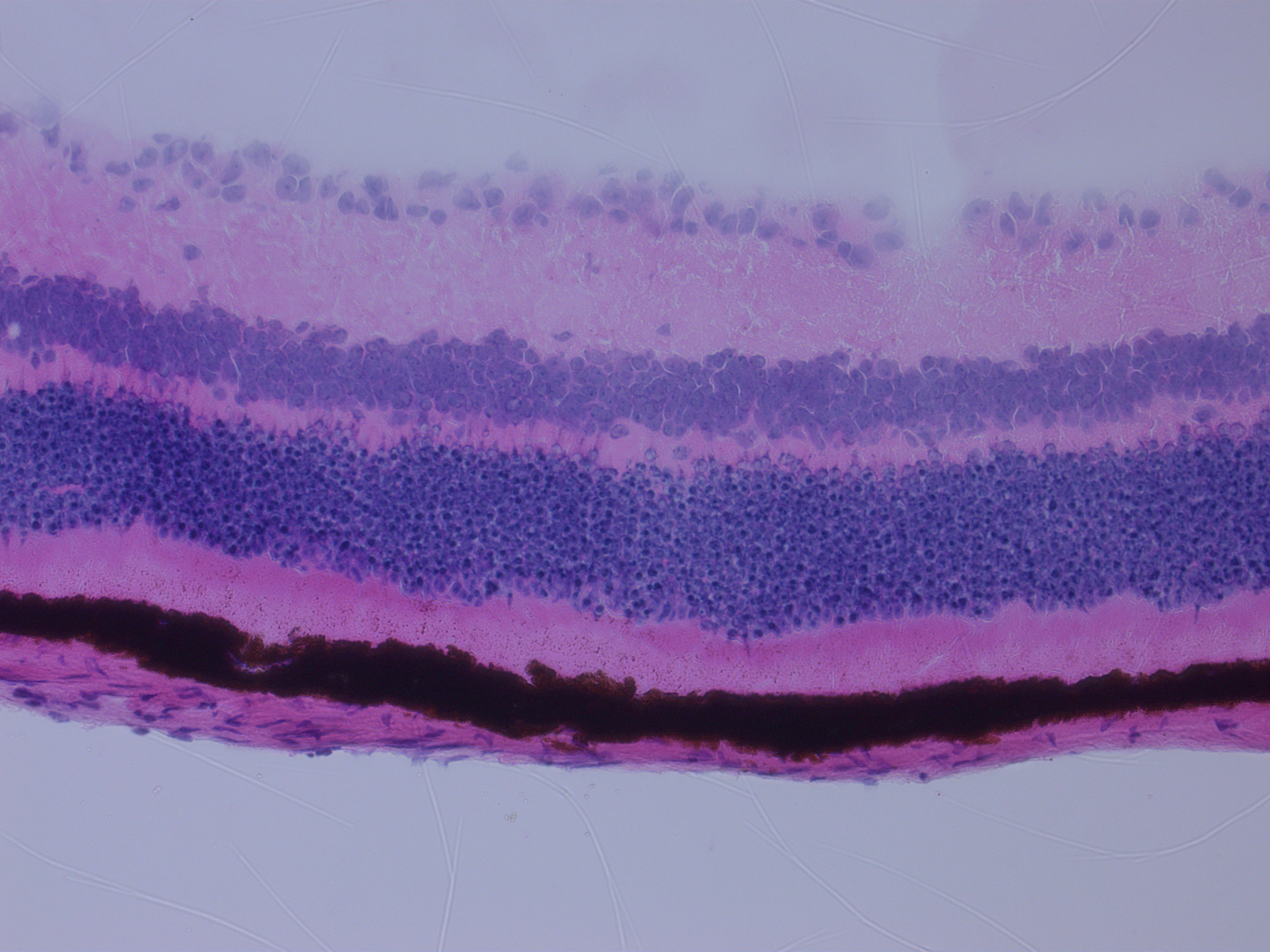

Supplement: Supplementary file 3 — Source data Fig. 1 [file 44321_2026_438_MOESM3_ESM.zip › Figure 1/1B/TwKOAstro-9 copy.tiff]

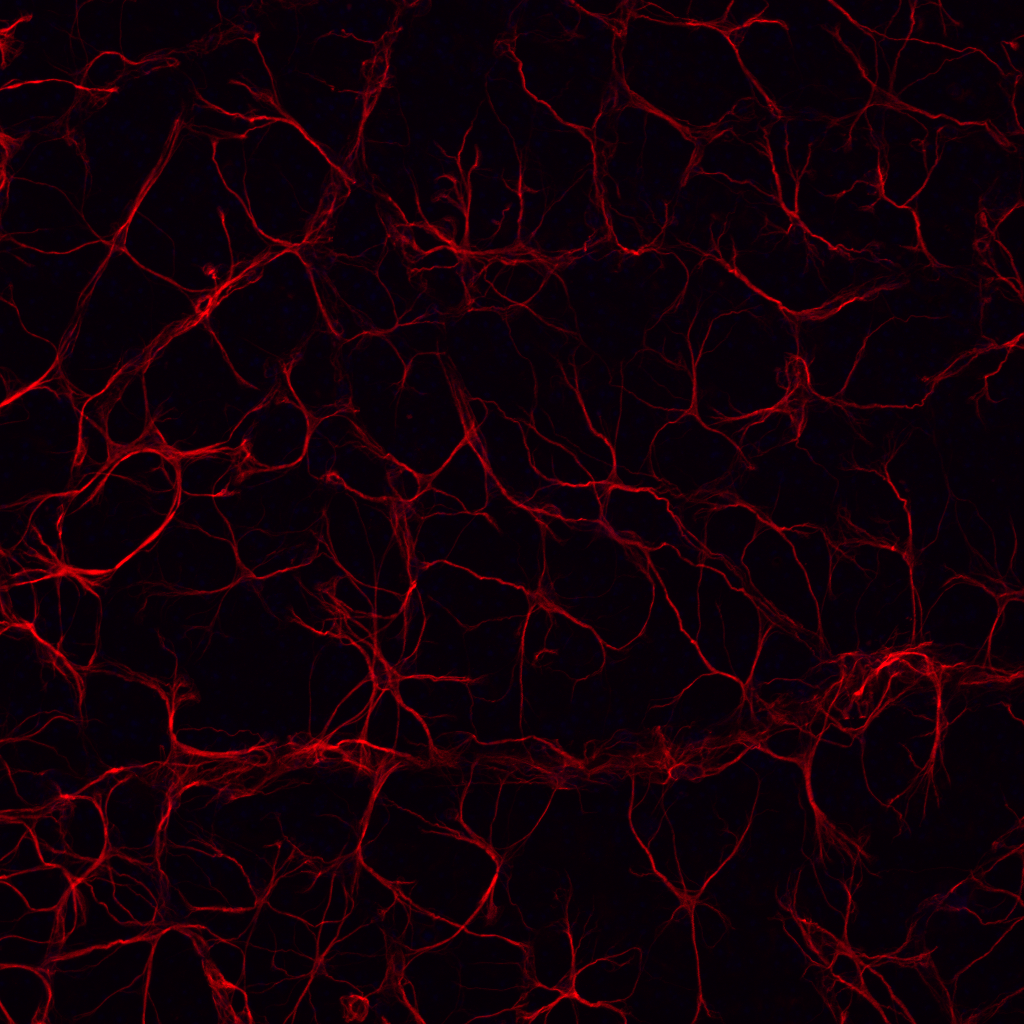

Supplement: Supplementary file 3 — Source data Fig. 1 [file 44321_2026_438_MOESM3_ESM.zip › Figure 1/1C/1211-GFAP-2-20x-zstack_Maximum intensity projection copy.tiff]

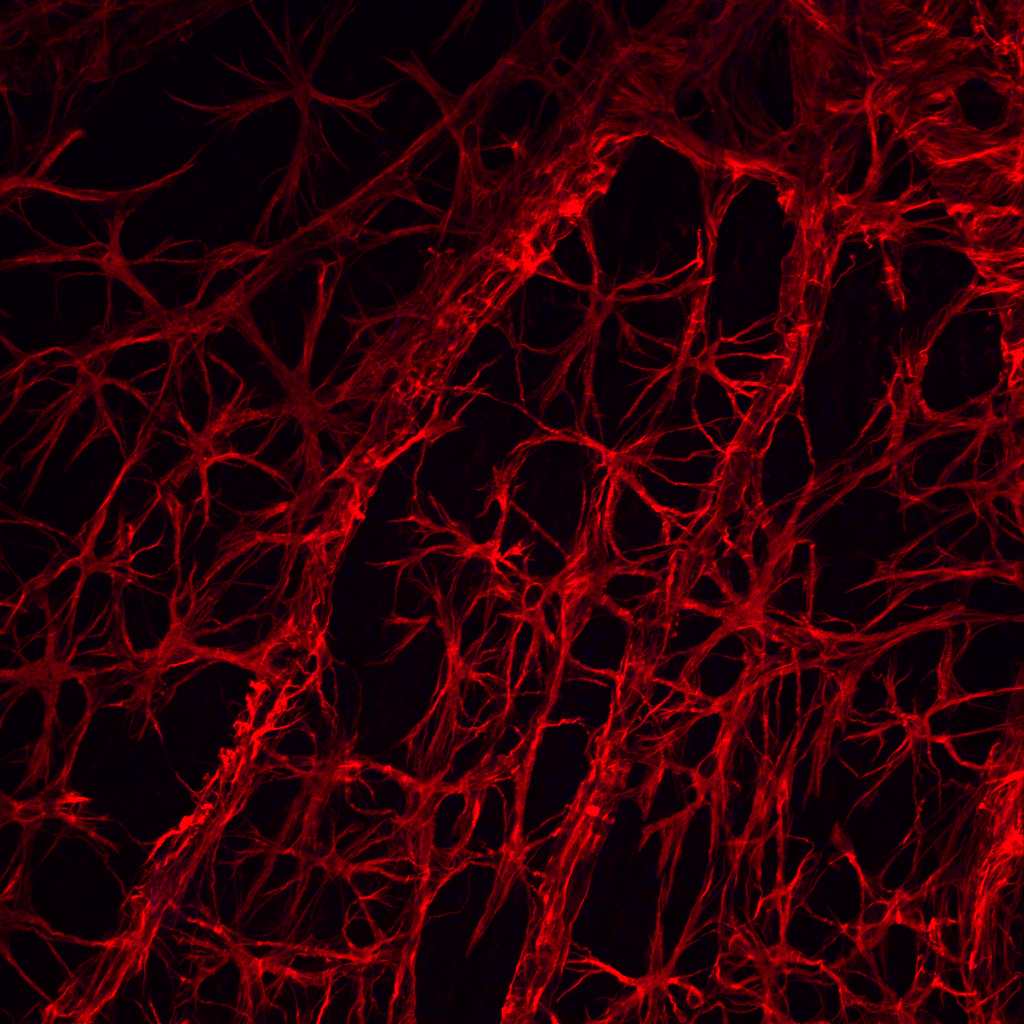

Supplement: Supplementary file 3 — Source data Fig. 1 [file 44321_2026_438_MOESM3_ESM.zip › Figure 1/1C/1213-GFAP-4-20x-zstack_Maximum intensity projection copy.tiff]

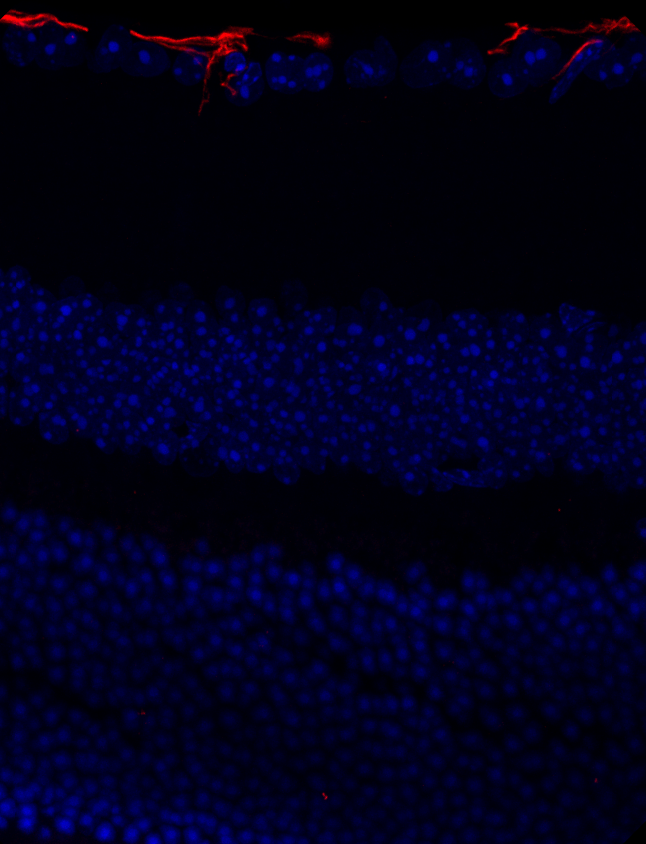

Supplement: Supplementary file 3 — Source data Fig. 1 [file 44321_2026_438_MOESM3_ESM.zip › Figure 1/1C/OIM1487_GFAP_40x_2_rotated_c1-2.tif]

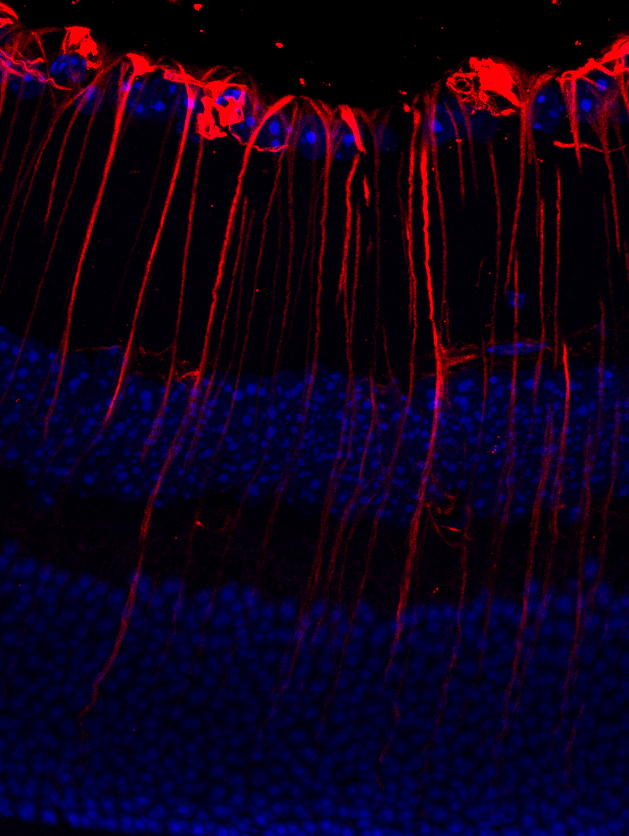

Supplement: Supplementary file 3 — Source data Fig. 1 [file 44321_2026_438_MOESM3_ESM.zip › Figure 1/1C/OIM1492_GFAP_40x_9_rotated_c1-2.tif]

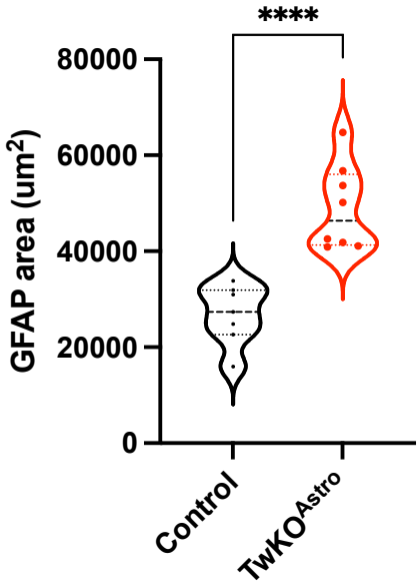

Supplement: Supplementary file 3 — Source data Fig. 1 [file 44321_2026_438_MOESM3_ESM.zip › Figure 1/1C/GFAP area um2_animal.pdf]

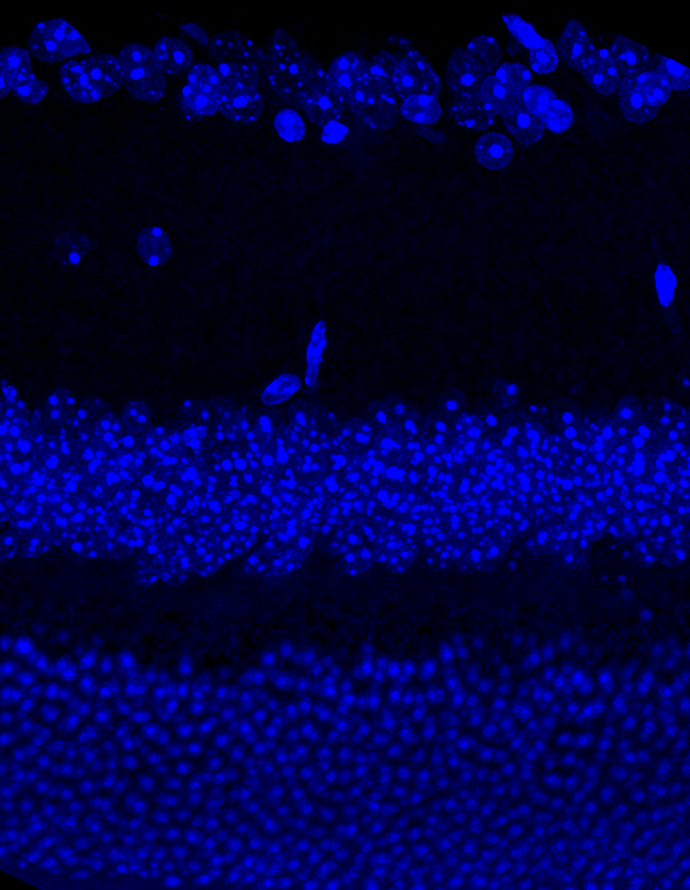

Supplement: Supplementary file 3 — Source data Fig. 1 [file 44321_2026_438_MOESM3_ESM.zip › Figure 1/1D/AW5822_Ctrl_IBA1_20um_40x_2_cropped_blue.tif]

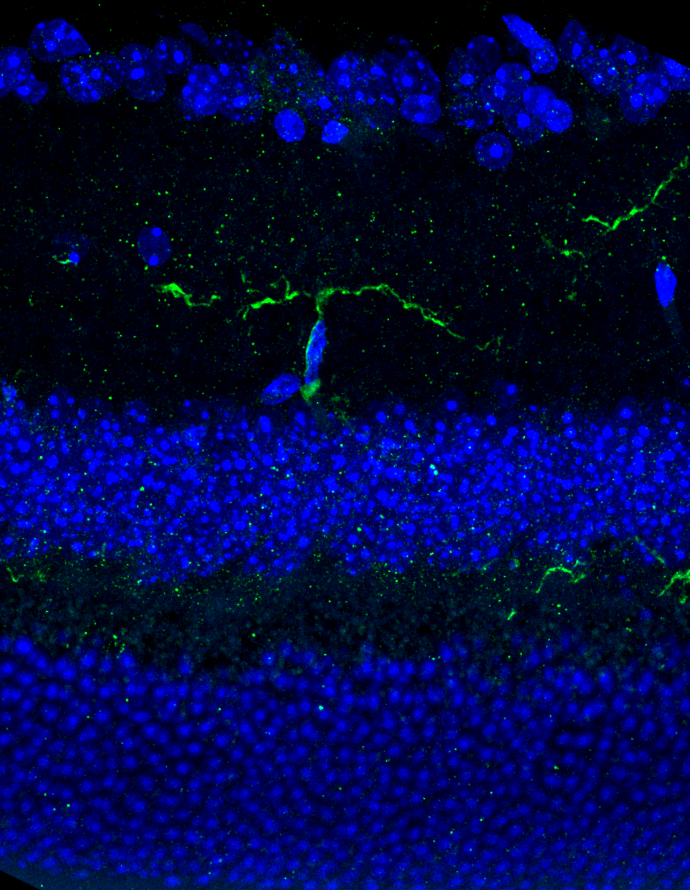

Supplement: Supplementary file 3 — Source data Fig. 1 [file 44321_2026_438_MOESM3_ESM.zip › Figure 1/1D/AW5822_Ctrl_IBA1_20um_40x_2_cropped_c1-2.tif]

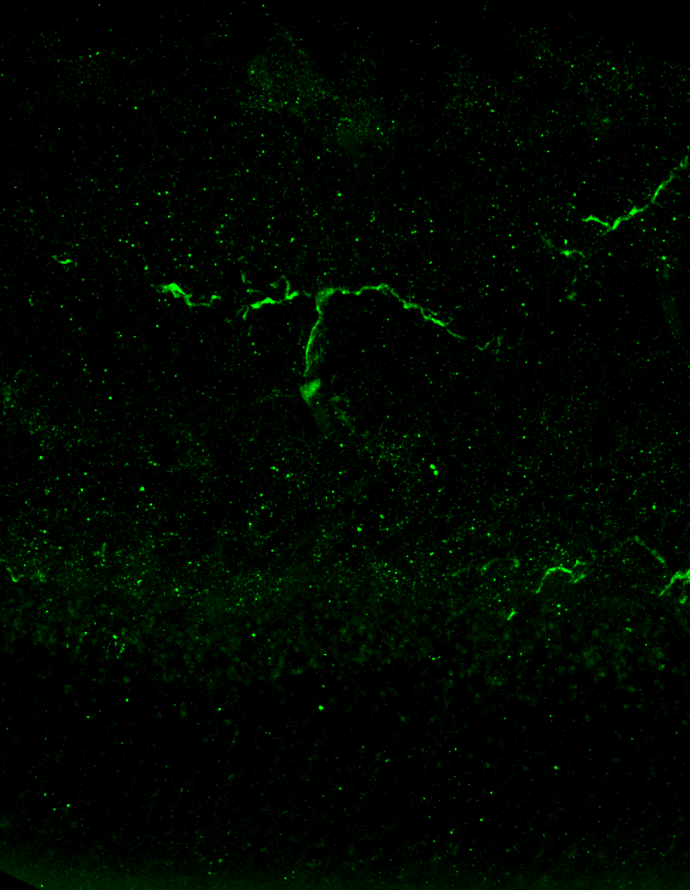

Supplement: Supplementary file 3 — Source data Fig. 1 [file 44321_2026_438_MOESM3_ESM.zip › Figure 1/1D/AW5822_Ctrl_IBA1_20um_40x_2_cropped_green.tif]

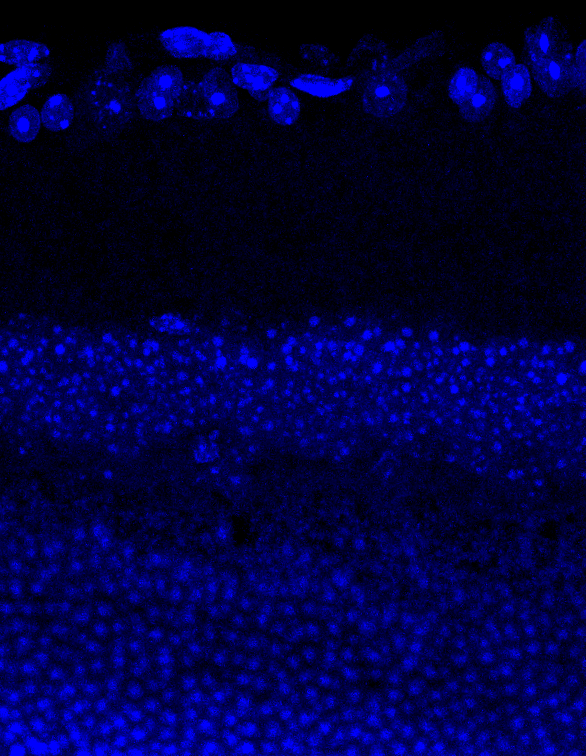

Supplement: Supplementary file 3 — Source data Fig. 1 [file 44321_2026_438_MOESM3_ESM.zip › Figure 1/1D/AW5823_KO_IBA1_20um_40x_4_cropped_blue.tif]

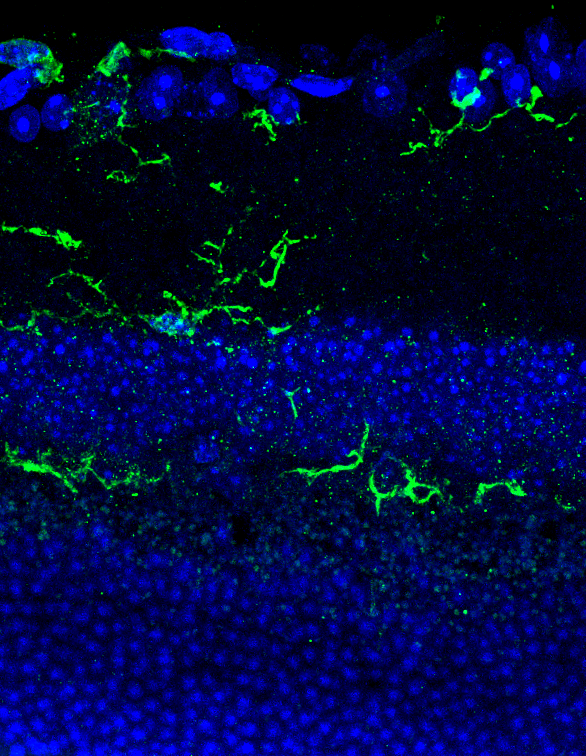

Supplement: Supplementary file 3 — Source data Fig. 1 [file 44321_2026_438_MOESM3_ESM.zip › Figure 1/1D/AW5823_KO_IBA1_20um_40x_4_cropped_c1-2.tif]

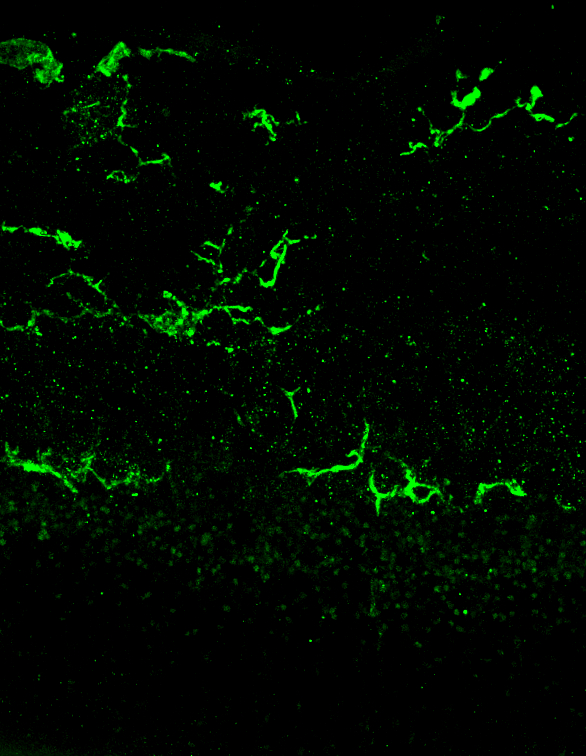

Supplement: Supplementary file 3 — Source data Fig. 1 [file 44321_2026_438_MOESM3_ESM.zip › Figure 1/1D/AW5823_KO_IBA1_20um_40x_4_cropped_green.tif]

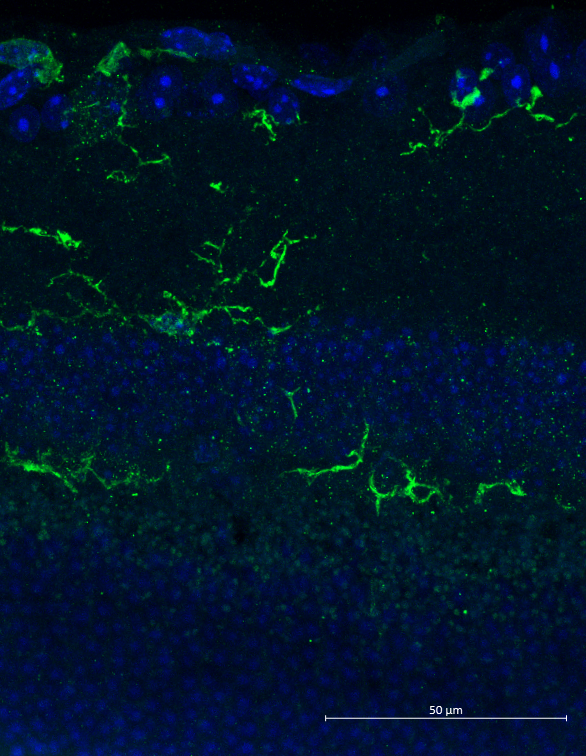

Supplement: Supplementary file 3 — Source data Fig. 1 [file 44321_2026_438_MOESM3_ESM.zip › Figure 1/1D/AW5823_KO_IBA1_20um_40x_4_cropped_scale_c1-2.tif]

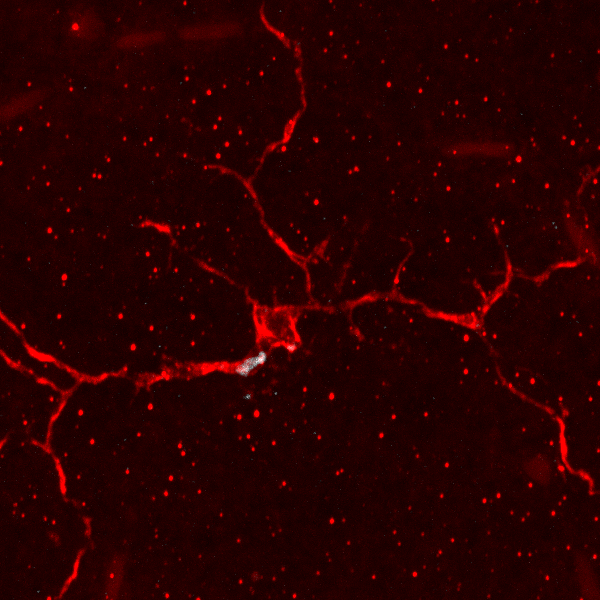

Supplement: Supplementary file 3 — Source data Fig. 1 [file 44321_2026_438_MOESM3_ESM.zip › Figure 1/1E/FS020_AW7111_10um.png]

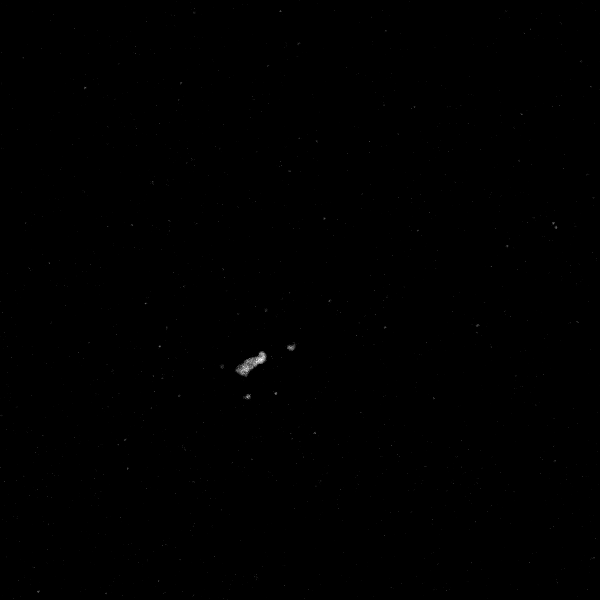

Supplement: Supplementary file 3 — Source data Fig. 1 [file 44321_2026_438_MOESM3_ESM.zip › Figure 1/1E/FS020_AW7111_10um_CD68.png]

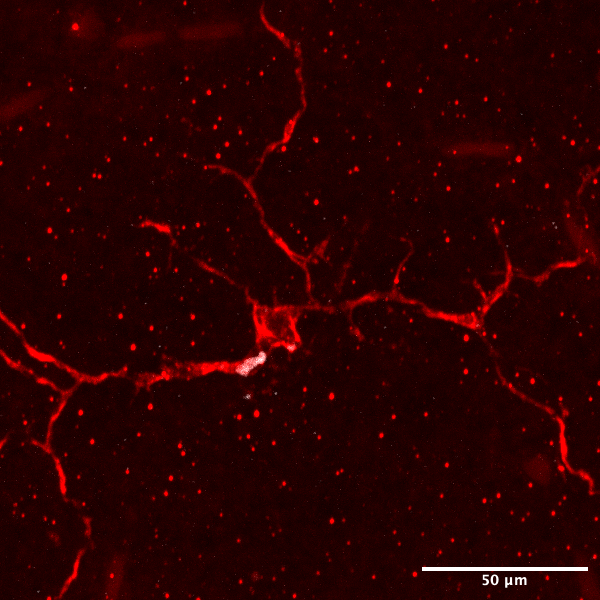

Supplement: Supplementary file 3 — Source data Fig. 1 [file 44321_2026_438_MOESM3_ESM.zip › Figure 1/1E/FS020_AW7111_10um_CropScale.png]

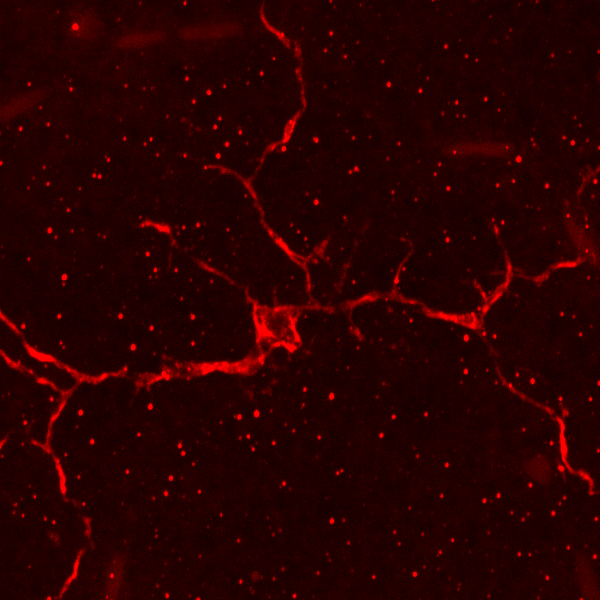

Supplement: Supplementary file 3 — Source data Fig. 1 [file 44321_2026_438_MOESM3_ESM.zip › Figure 1/1E/FS020_AW7111_10um_IBA1.png]

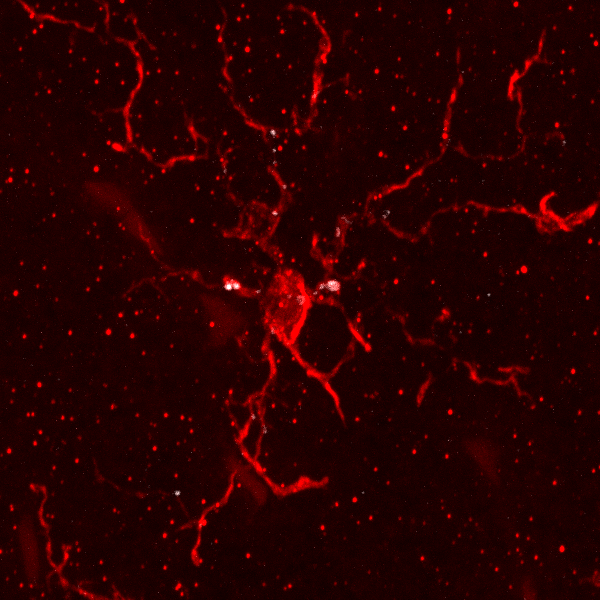

Supplement: Supplementary file 3 — Source data Fig. 1 [file 44321_2026_438_MOESM3_ESM.zip › Figure 1/1E/FS020_AW7114_10um.png]

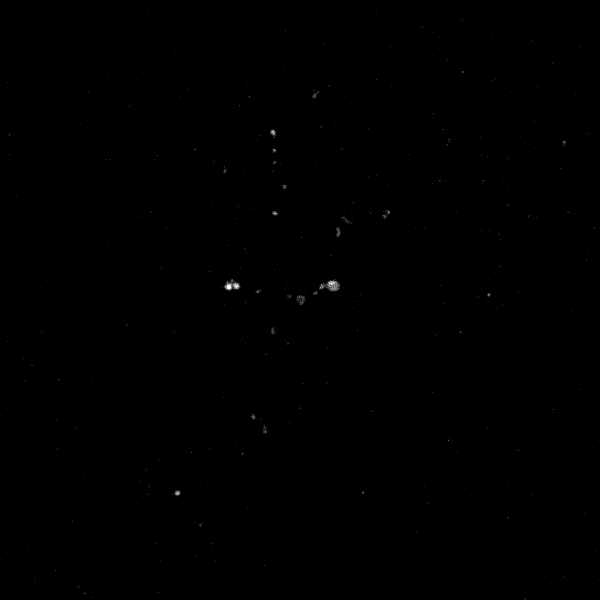

Supplement: Supplementary file 3 — Source data Fig. 1 [file 44321_2026_438_MOESM3_ESM.zip › Figure 1/1E/FS020_AW7114_10um_CD68.png]

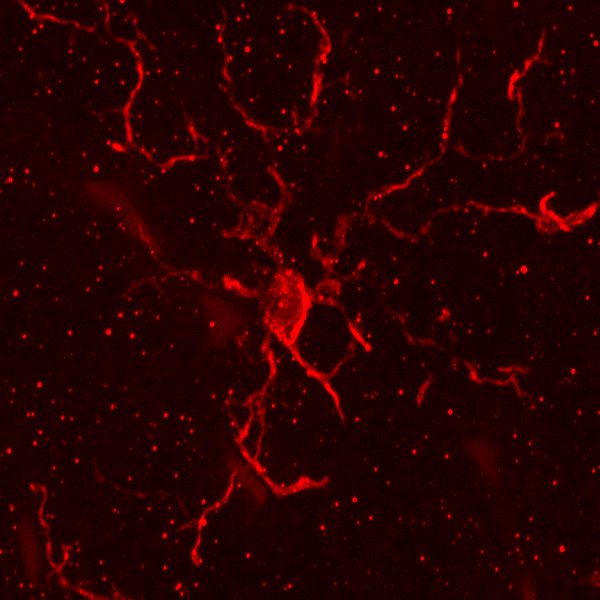

Supplement: Supplementary file 3 — Source data Fig. 1 [file 44321_2026_438_MOESM3_ESM.zip › Figure 1/1E/FS020_AW7114_10um_IBA1.png]

# CD68 count in IBA1

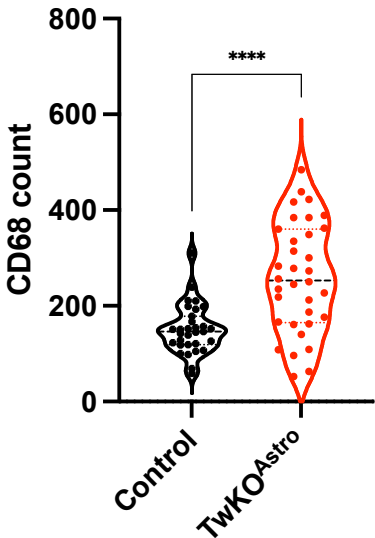

Supplement: Supplementary file 3 — Source data Fig. 1 [file 44321_2026_438_MOESM3_ESM.zip › Figure 1/1F/1F CD68 count in IBA1_image um.pdf]

# IBA1 area

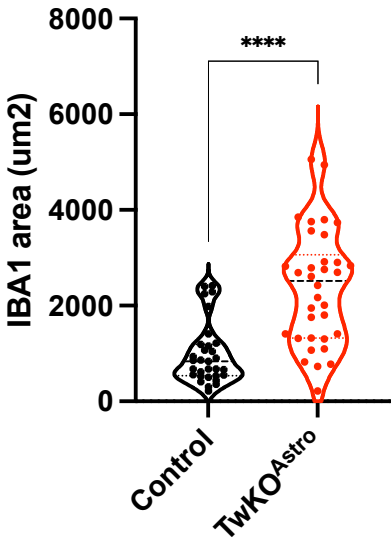

Supplement: Supplementary file 3 — Source data Fig. 1 [file 44321_2026_438_MOESM3_ESM.zip › Figure 1/1F/1F IBA1 area_image um.pdf]

# IBA1 perimeter

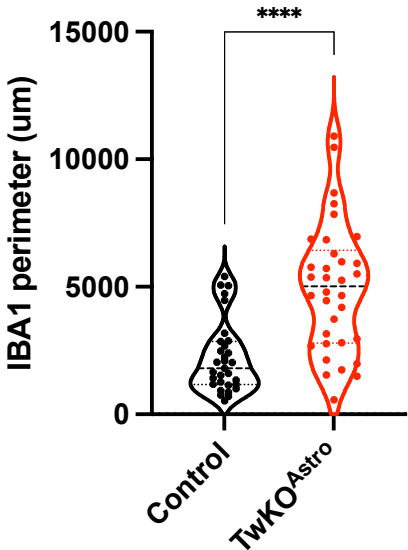

Supplement: Supplementary file 3 — Source data Fig. 1 [file 44321_2026_438_MOESM3_ESM.zip › Figure 1/1F/1F IBA1 perimeter_image um.pdf]

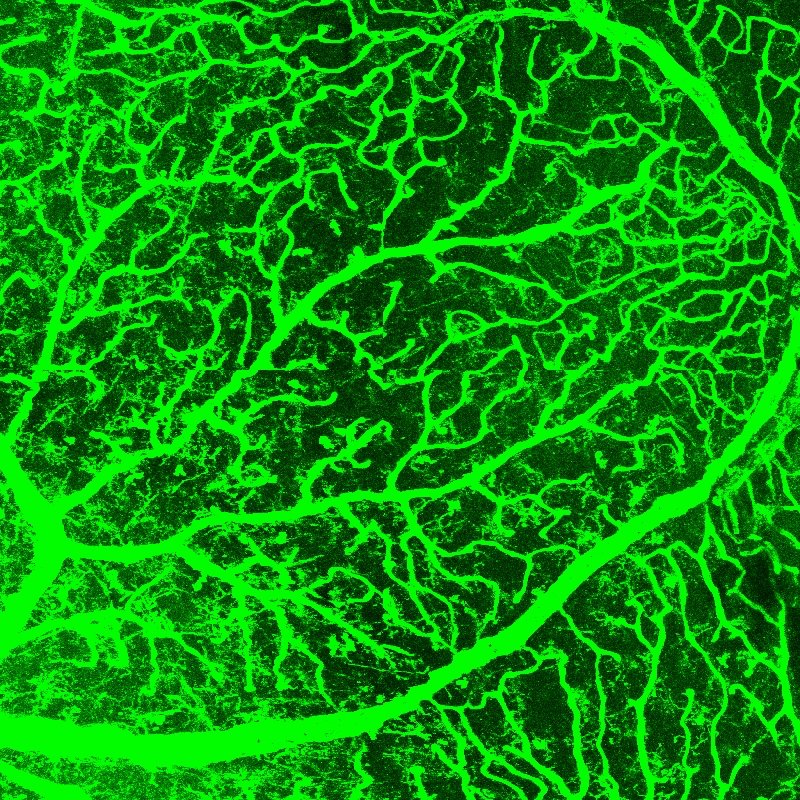

Supplement: Supplementary file 4 — Source data Fig. 2 [file 44321_2026_438_MOESM4_ESM.zip › Figure 2/2A/1207_IB4_Aqua4_10x_Zstack_Tiles_Maximum intensity projection-Create Image Subset-07.jpg]

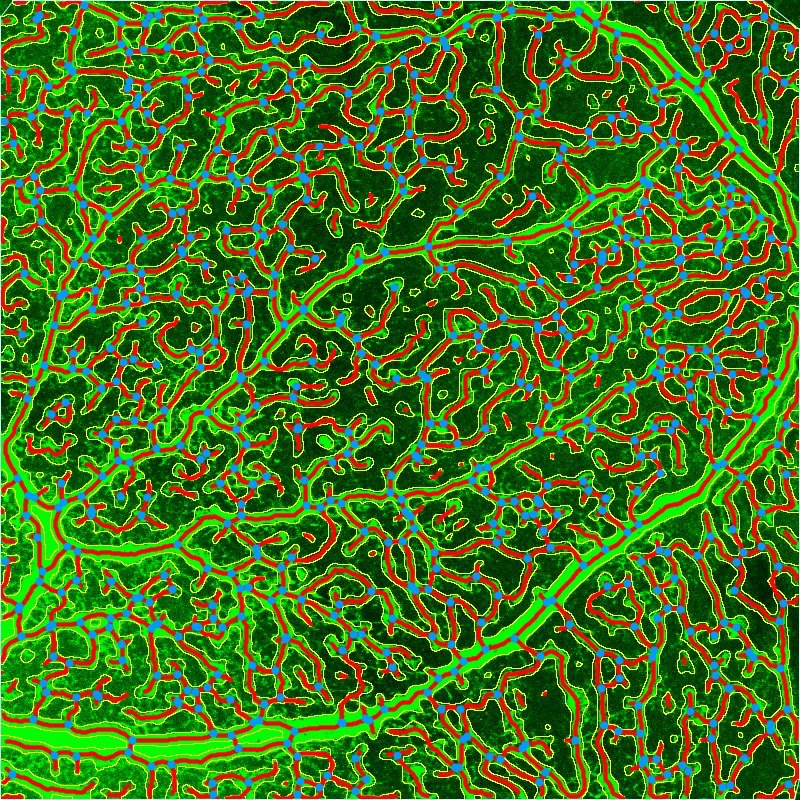

Supplement: Supplementary file 4 — Source data Fig. 2 [file 44321_2026_438_MOESM4_ESM.zip › Figure 2/2A/1207_IB4_Aqua4_10x_Zstack_Tiles_Maximum intensity projection-Create Image Subset-07.jpg result.jpg]

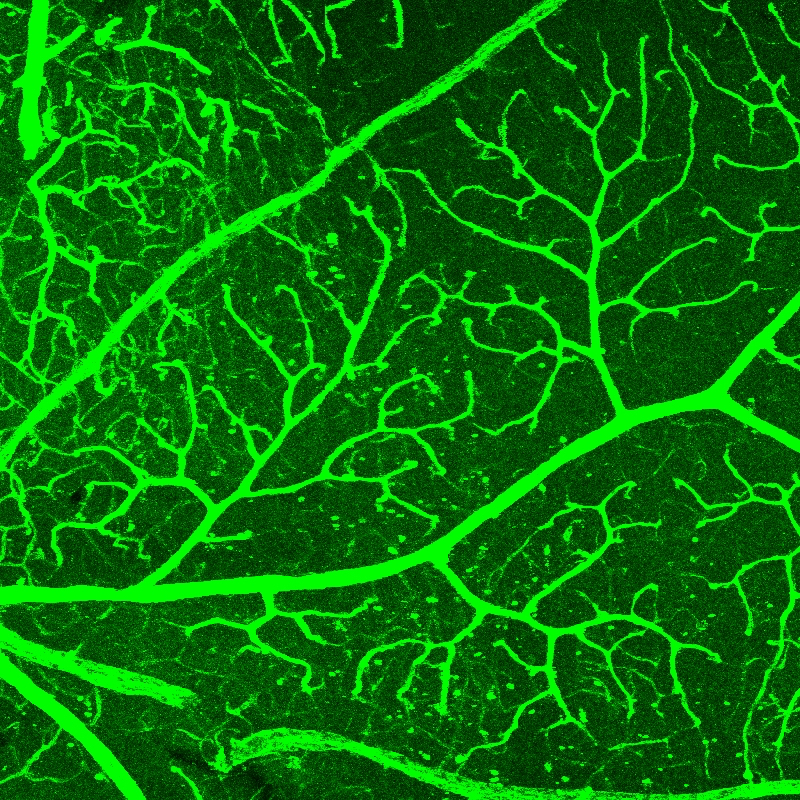

Supplement: Supplementary file 4 — Source data Fig. 2 [file 44321_2026_438_MOESM4_ESM.zip › Figure 2/2A/1240_IB4_Aqua4_10x_Zstack_Tiles_Maximum intensity projection-Create Image Subset-45.jpg]

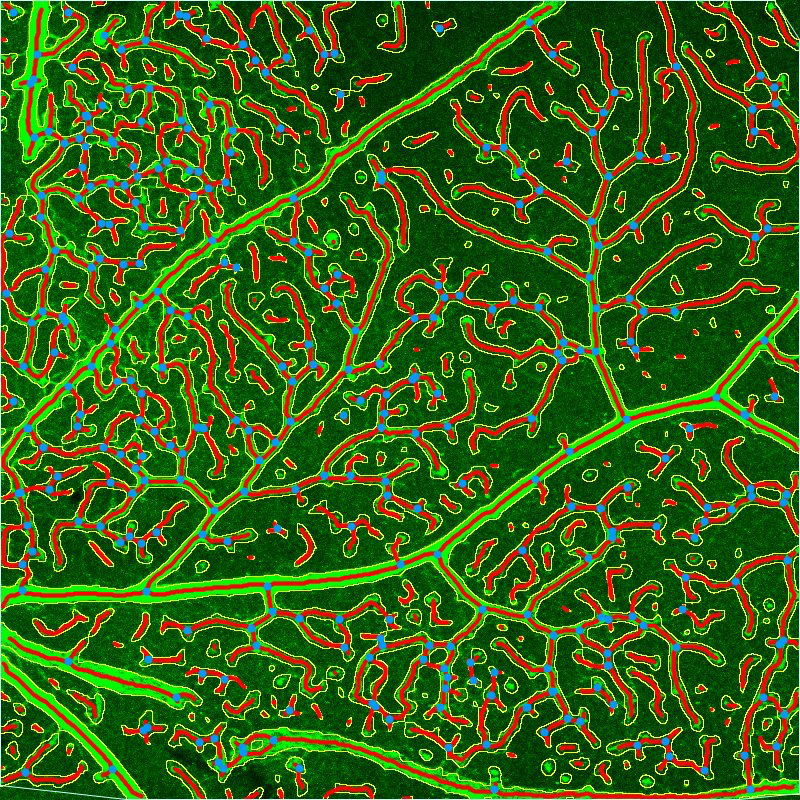

Supplement: Supplementary file 4 — Source data Fig. 2 [file 44321_2026_438_MOESM4_ESM.zip › Figure 2/2A/1240_IB4_Aqua4_10x_Zstack_Tiles_Maximum intensity projection-Create Image Subset-45.jpg result.jpg]

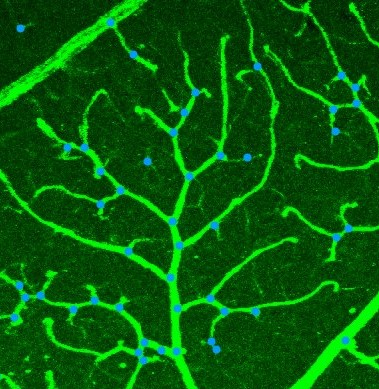

Supplement: Supplementary file 4 — Source data Fig. 2 [file 44321_2026_438_MOESM4_ESM.zip › Figure 2/2A/BP cropped 1240_IB4_Aqua4_10x_Zstack_Tiles_Maximum intensity projection-Create Image Subset-45.jpg result.jpg]

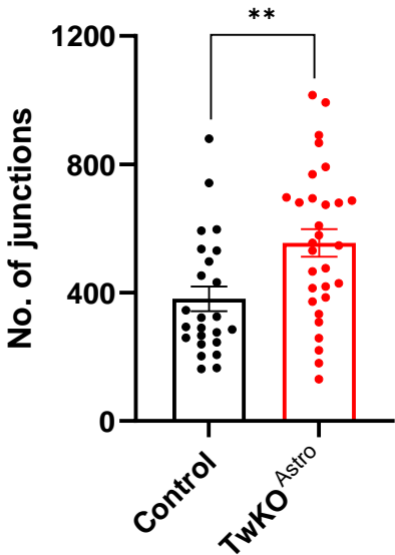

Supplement: Supplementary file 4 — Source data Fig. 2 [file 44321_2026_438_MOESM4_ESM.zip › Figure 2/2B/No of junctions (males, 3 values per mouse) black and red.pdf]

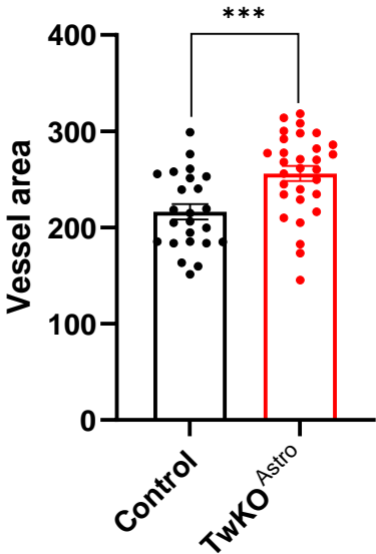

Supplement: Supplementary file 4 — Source data Fig. 2 [file 44321_2026_438_MOESM4_ESM.zip › Figure 2/2B/Vessel area (males, 3 values per mouse) black and red.pdf]

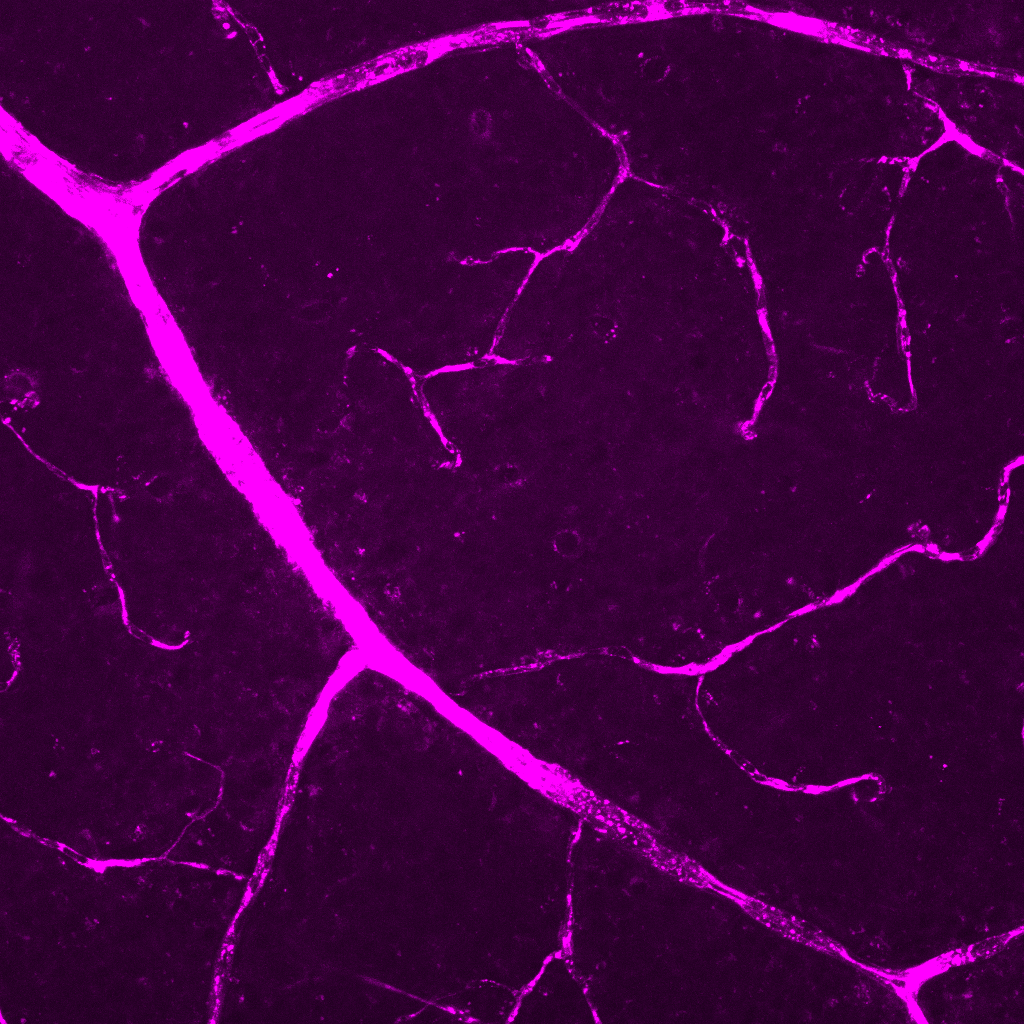

Supplement: Supplementary file 4 — Source data Fig. 2 [file 44321_2026_438_MOESM4_ESM.zip › Figure 2/2D/IDG317B_retina_3_inner_zstack_Dextran_magenta.tif]

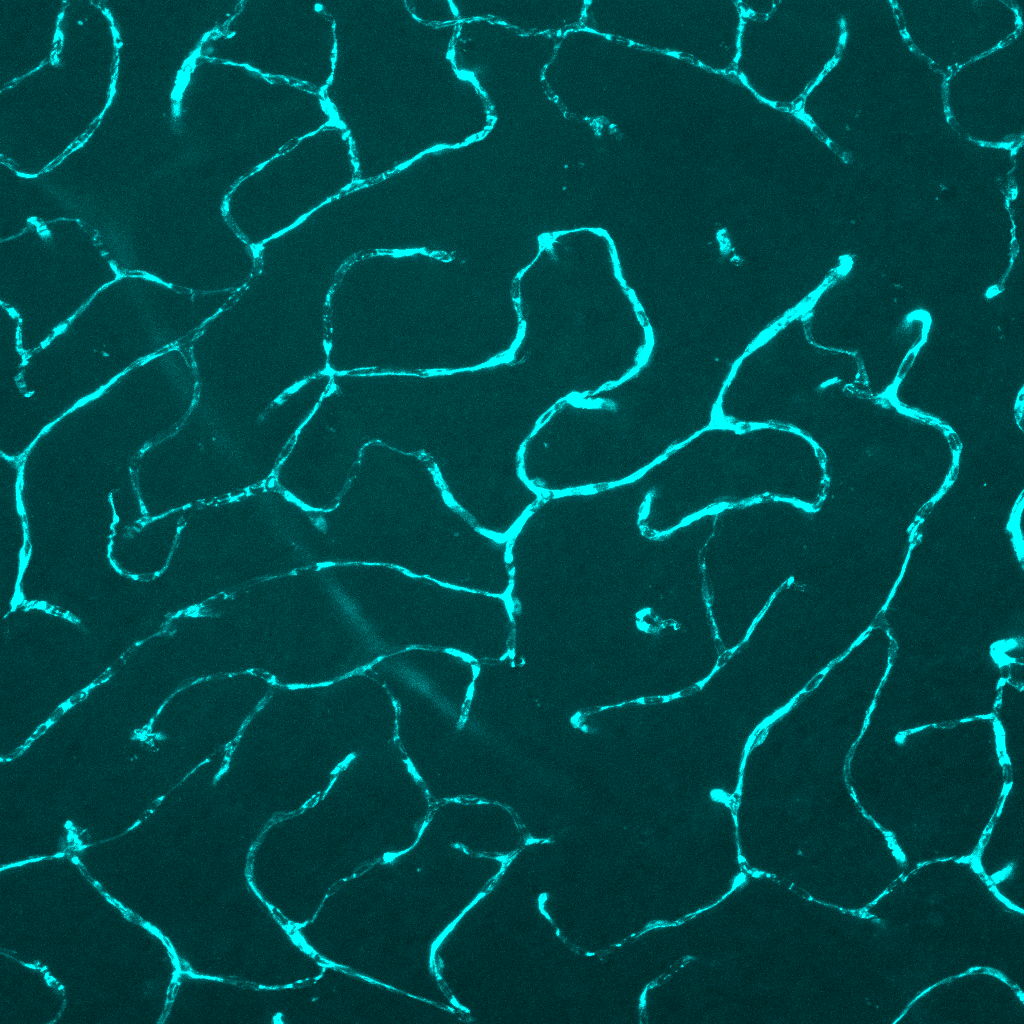

Supplement: Supplementary file 4 — Source data Fig. 2 [file 44321_2026_438_MOESM4_ESM.zip › Figure 2/2D/IDG317B_retina_3_middle_zstack_Dextran_cyan.tif]

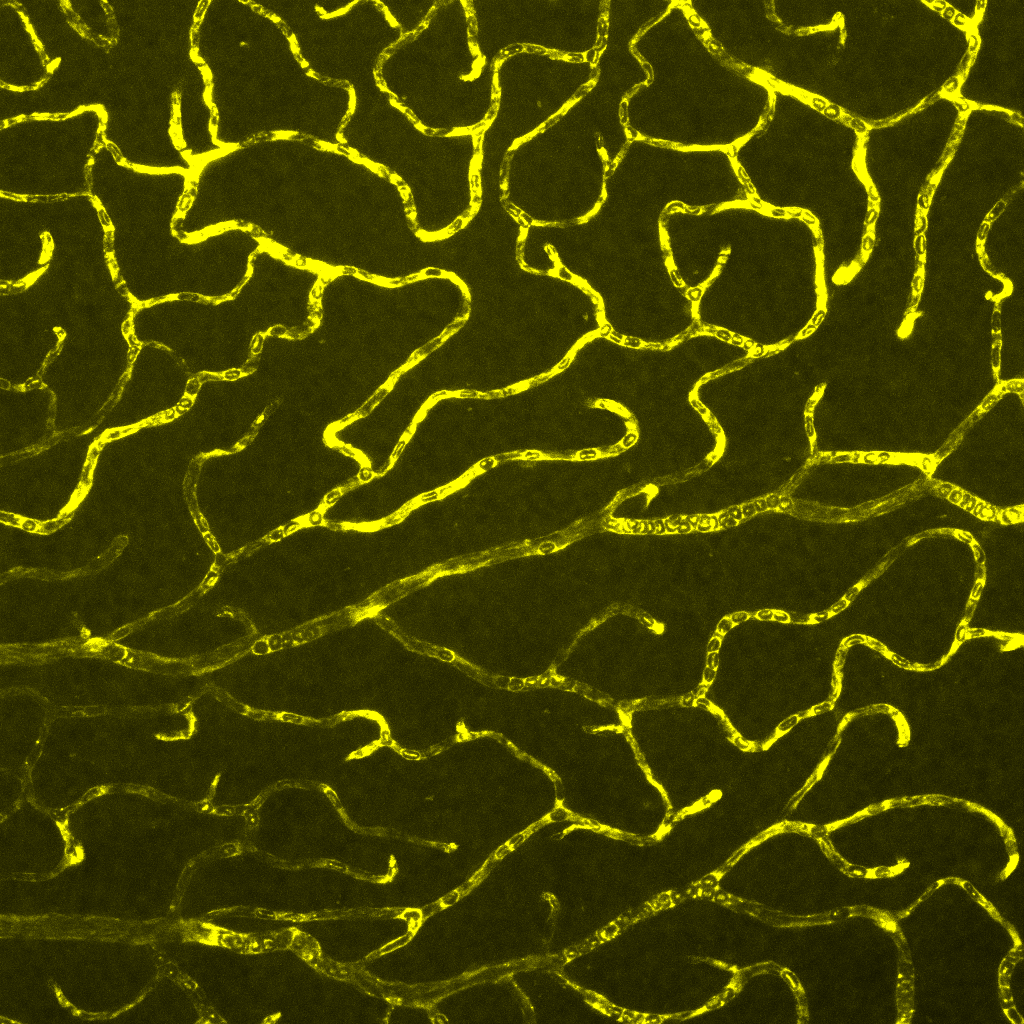

Supplement: Supplementary file 4 — Source data Fig. 2 [file 44321_2026_438_MOESM4_ESM.zip › Figure 2/2D/IDG317B_retina_3_outer_zstack_Dextran_yellow.tif]

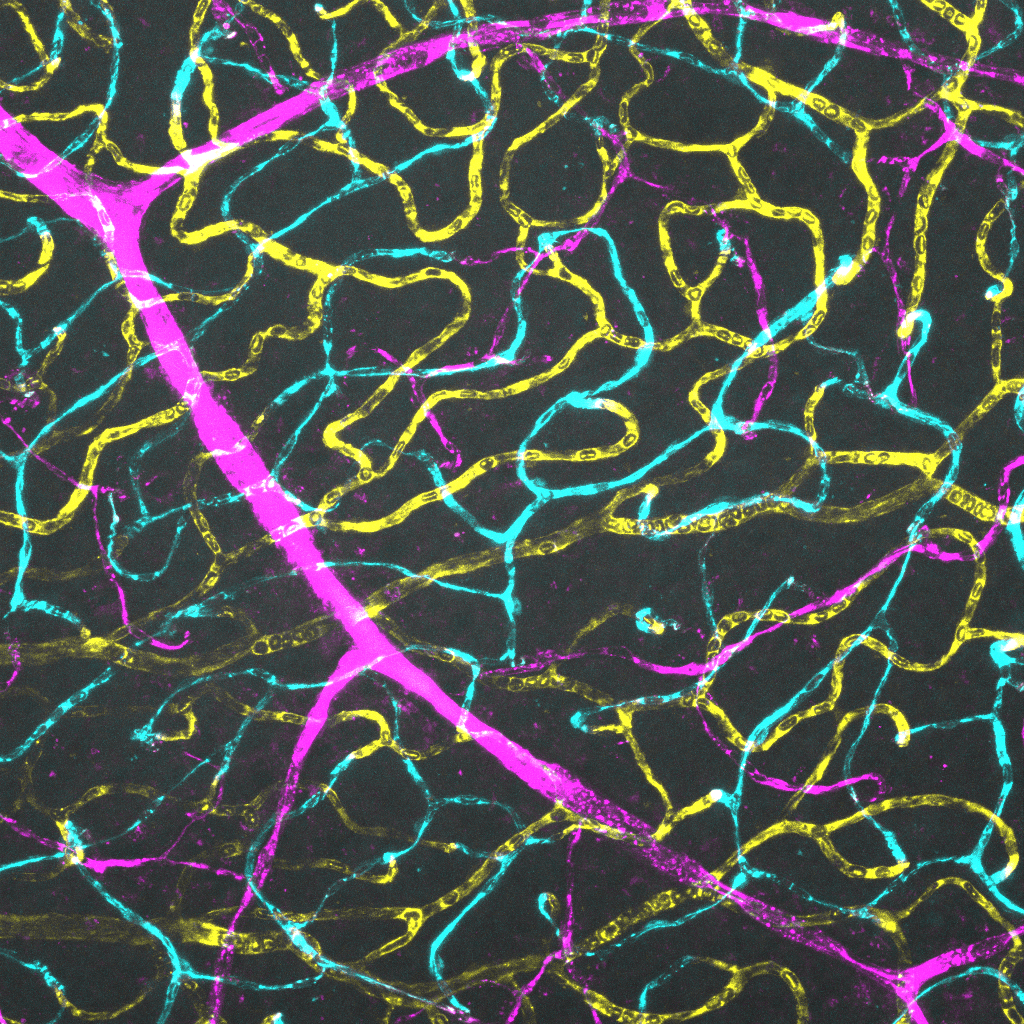

Supplement: Supplementary file 4 — Source data Fig. 2 [file 44321_2026_438_MOESM4_ESM.zip › Figure 2/2D/IDG317B_retina_3_total.png]

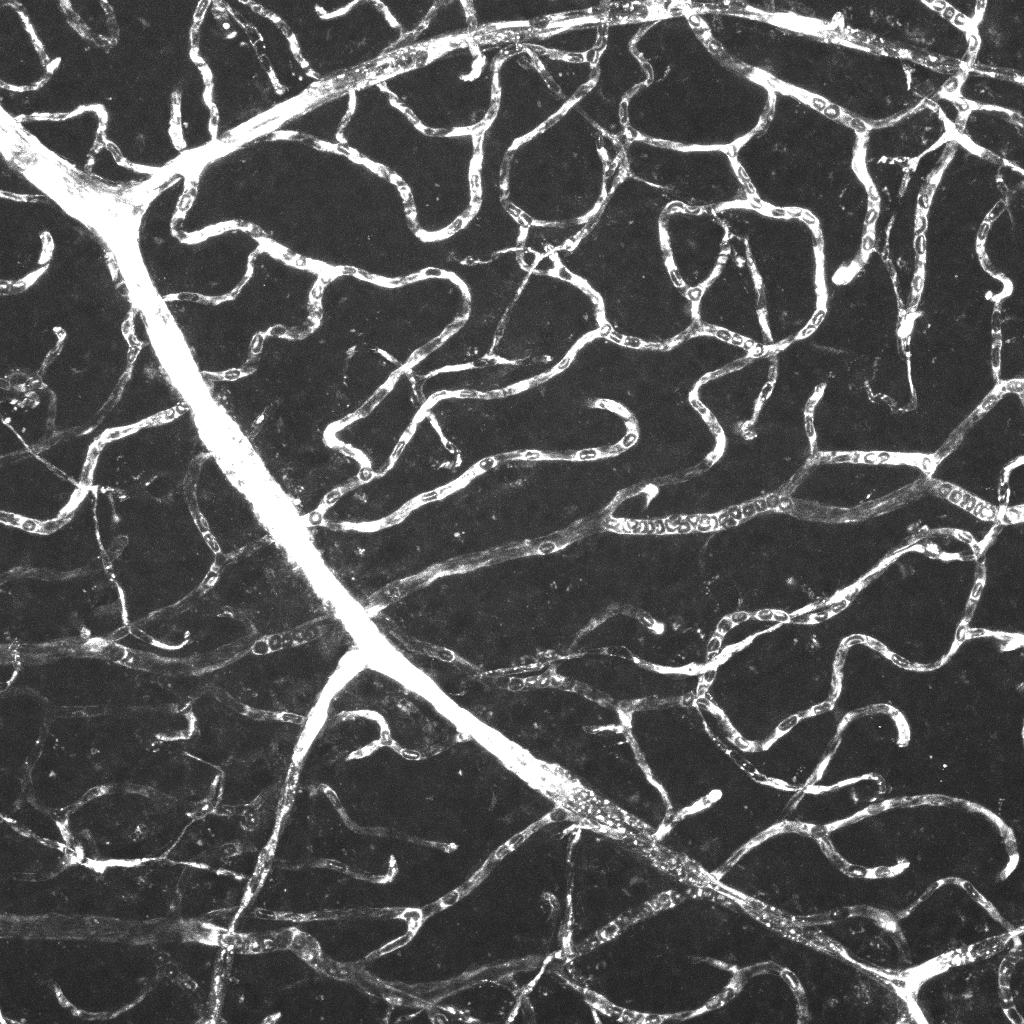

Supplement: Supplementary file 4 — Source data Fig. 2 [file 44321_2026_438_MOESM4_ESM.zip › Figure 2/2D/IDG317B_retina_3_total.tif]

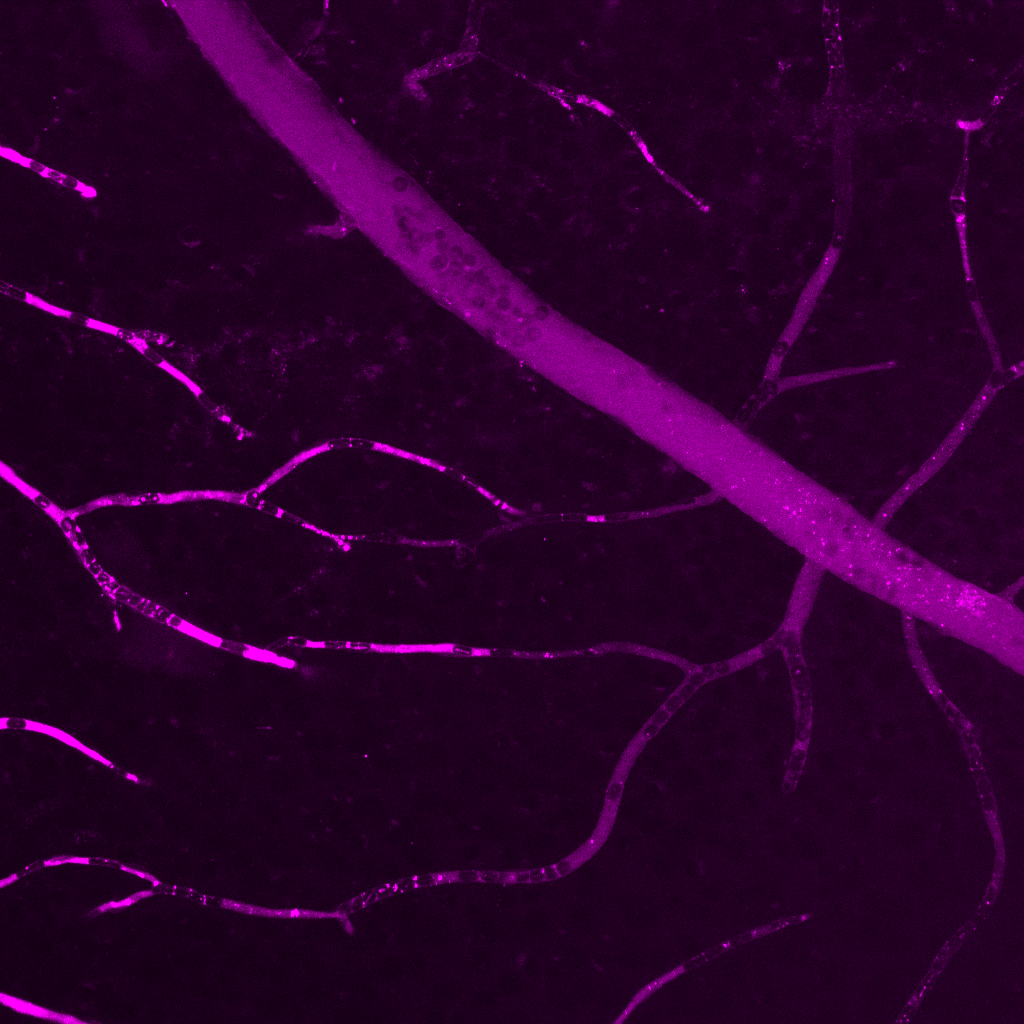

Supplement: Supplementary file 4 — Source data Fig. 2 [file 44321_2026_438_MOESM4_ESM.zip › Figure 2/2D/IDG318B_retina_3_inner_zstack_Dextran_magenta.tif]

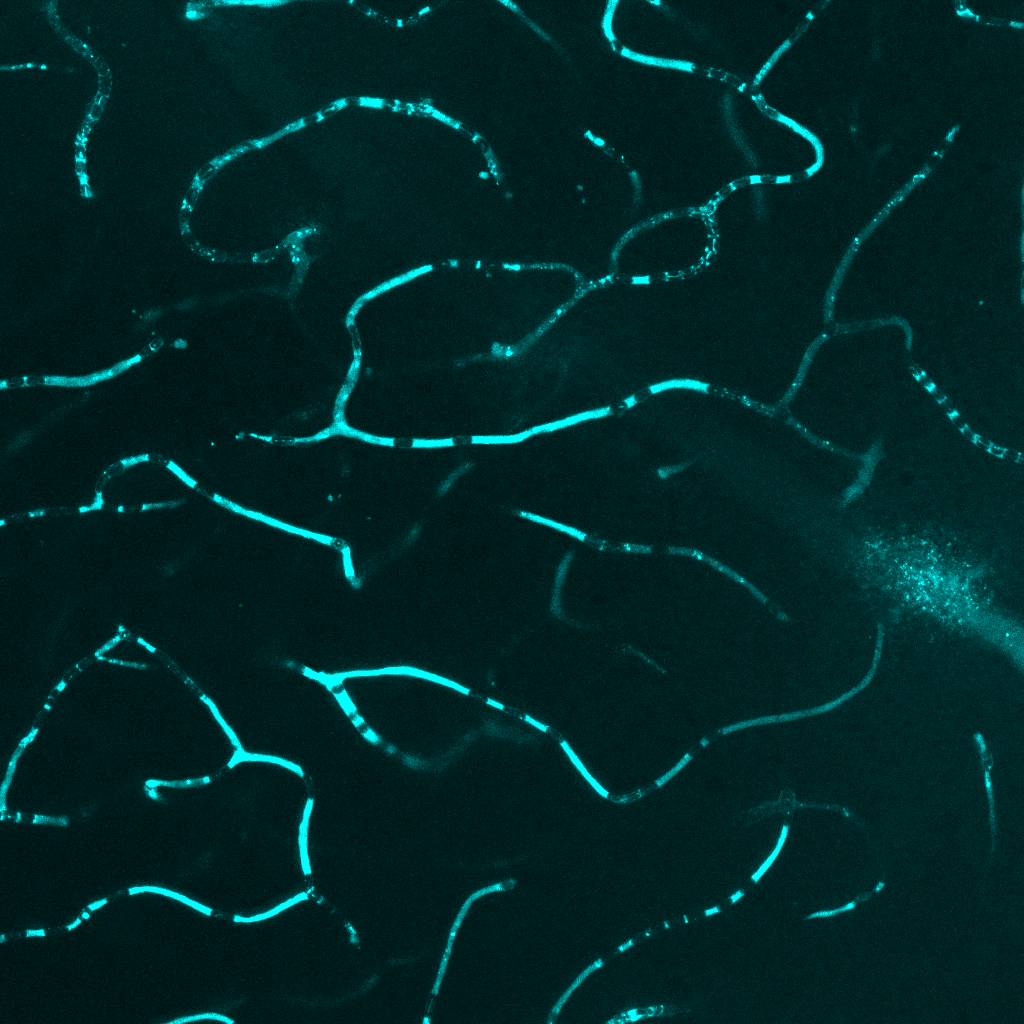

Supplement: Supplementary file 4 — Source data Fig. 2 [file 44321_2026_438_MOESM4_ESM.zip › Figure 2/2D/IDG318B_retina_3_middle_zstack_Dextran_cyan.tif]

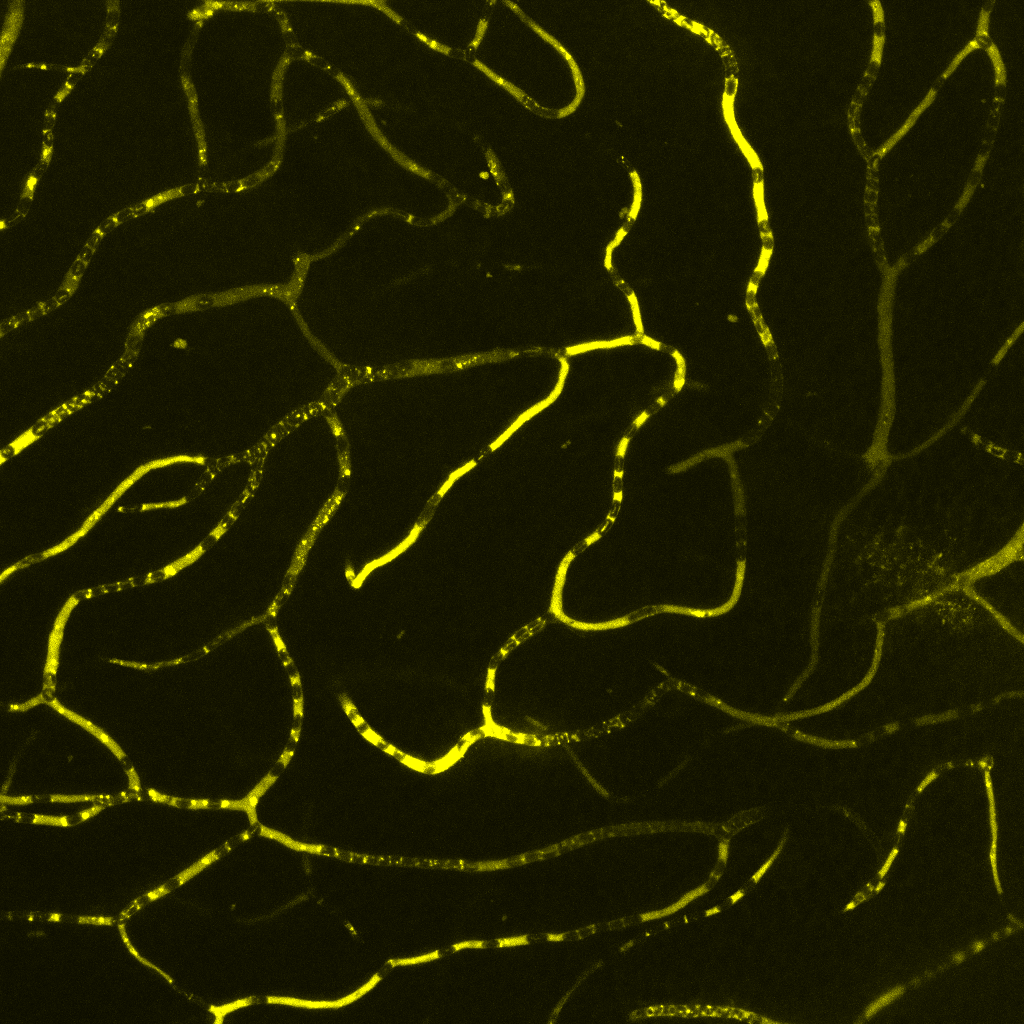

Supplement: Supplementary file 4 — Source data Fig. 2 [file 44321_2026_438_MOESM4_ESM.zip › Figure 2/2D/IDG318B_retina_3_outer_zstack_Dextran_yellow.tif]

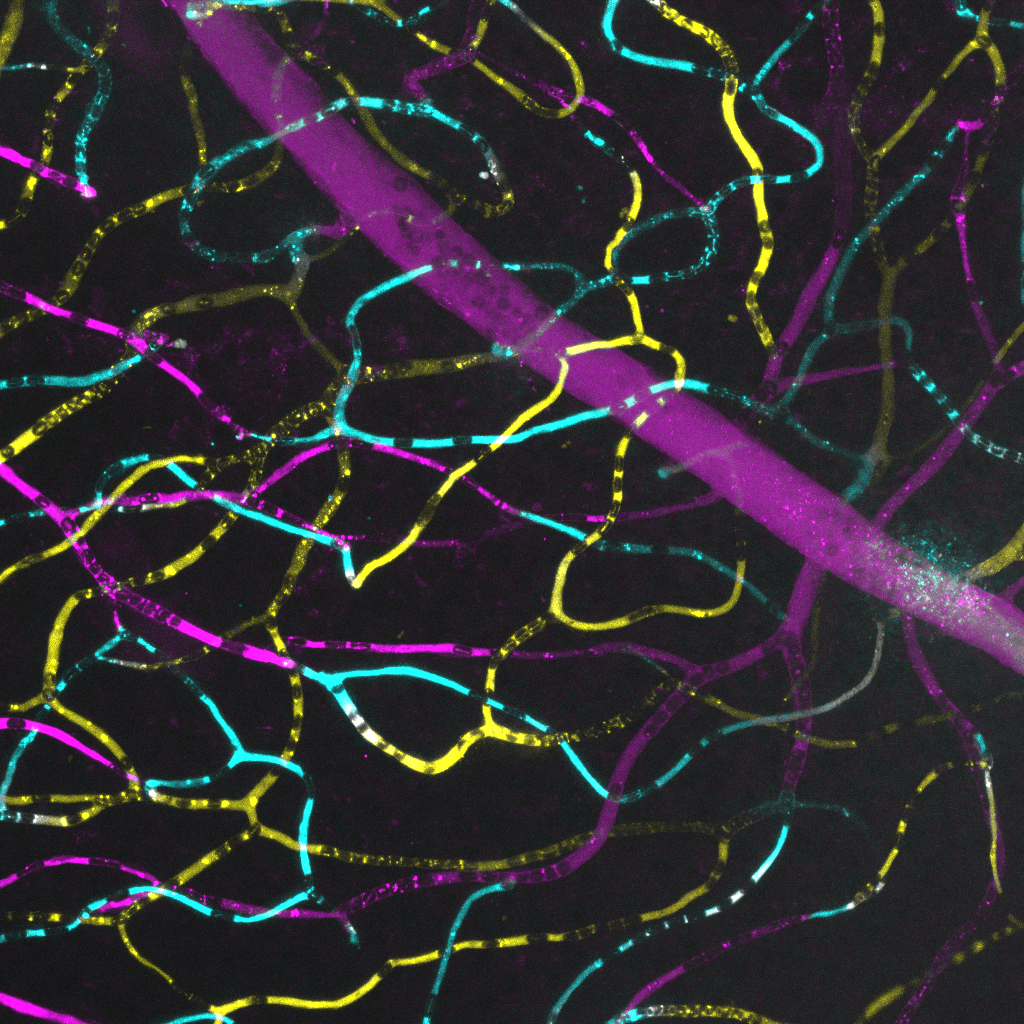

Supplement: Supplementary file 4 — Source data Fig. 2 [file 44321_2026_438_MOESM4_ESM.zip › Figure 2/2D/IDG318B_retina_3_total.png]

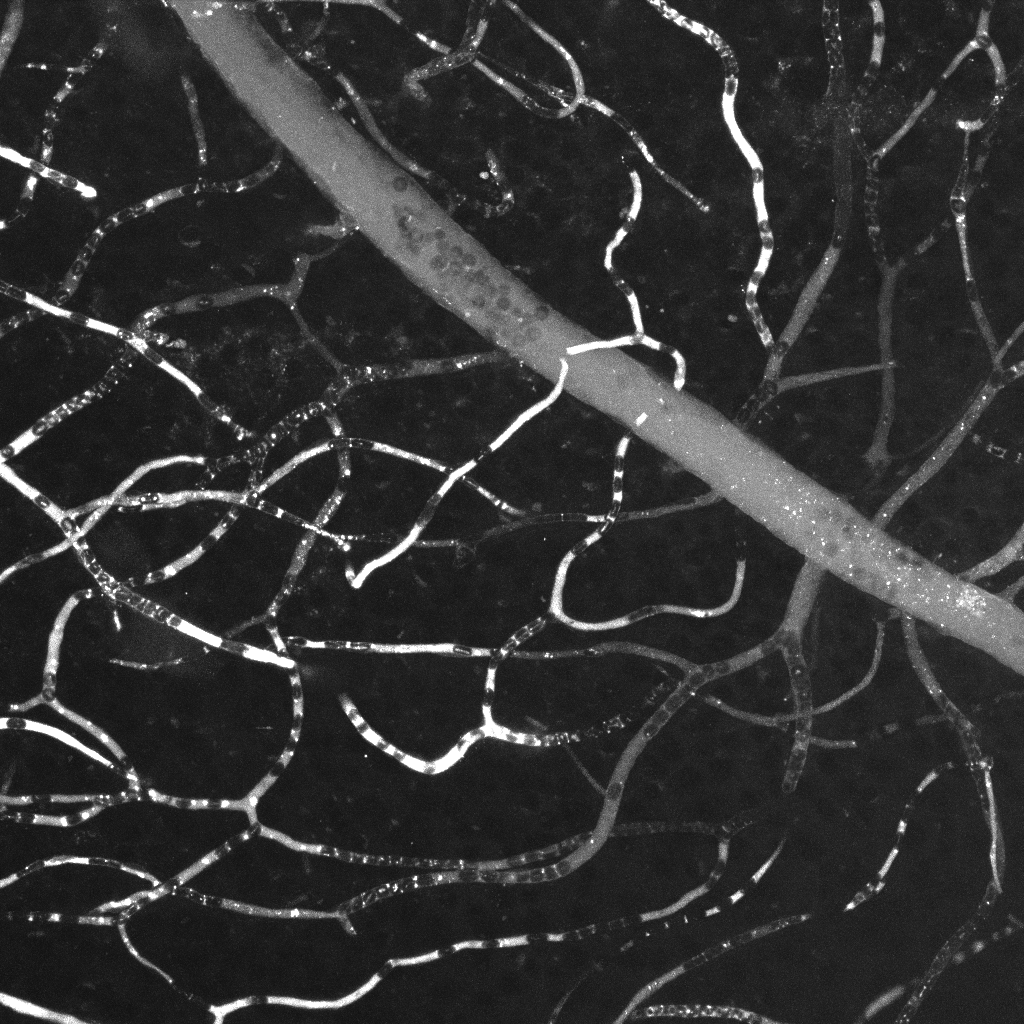

Supplement: Supplementary file 4 — Source data Fig. 2 [file 44321_2026_438_MOESM4_ESM.zip › Figure 2/2D/IDG318B_retina_3_total.tif]

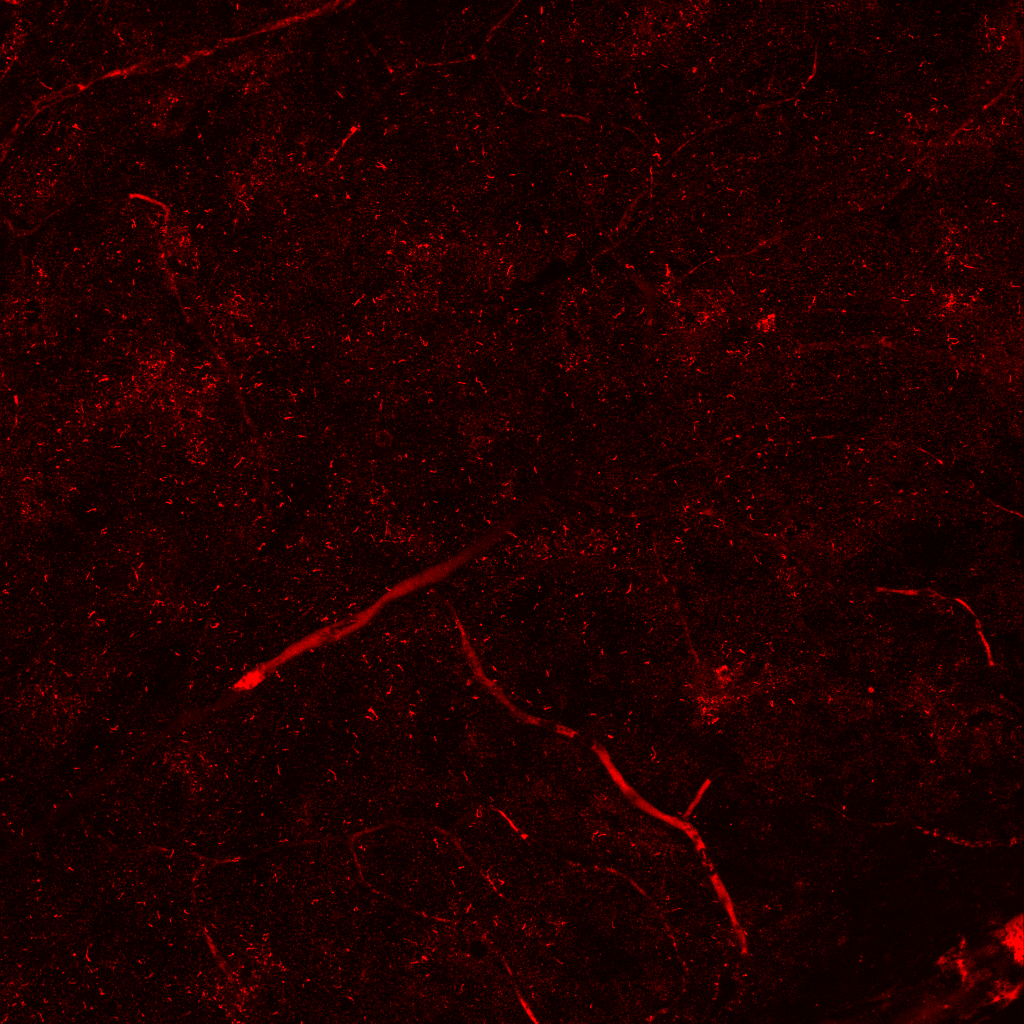

Supplement: Supplementary file 4 — Source data Fig. 2 [file 44321_2026_438_MOESM4_ESM.zip › Figure 2/2G/Astro5559(KO)_fibrinogen_20x-1_Maximum intensity projection.tif]

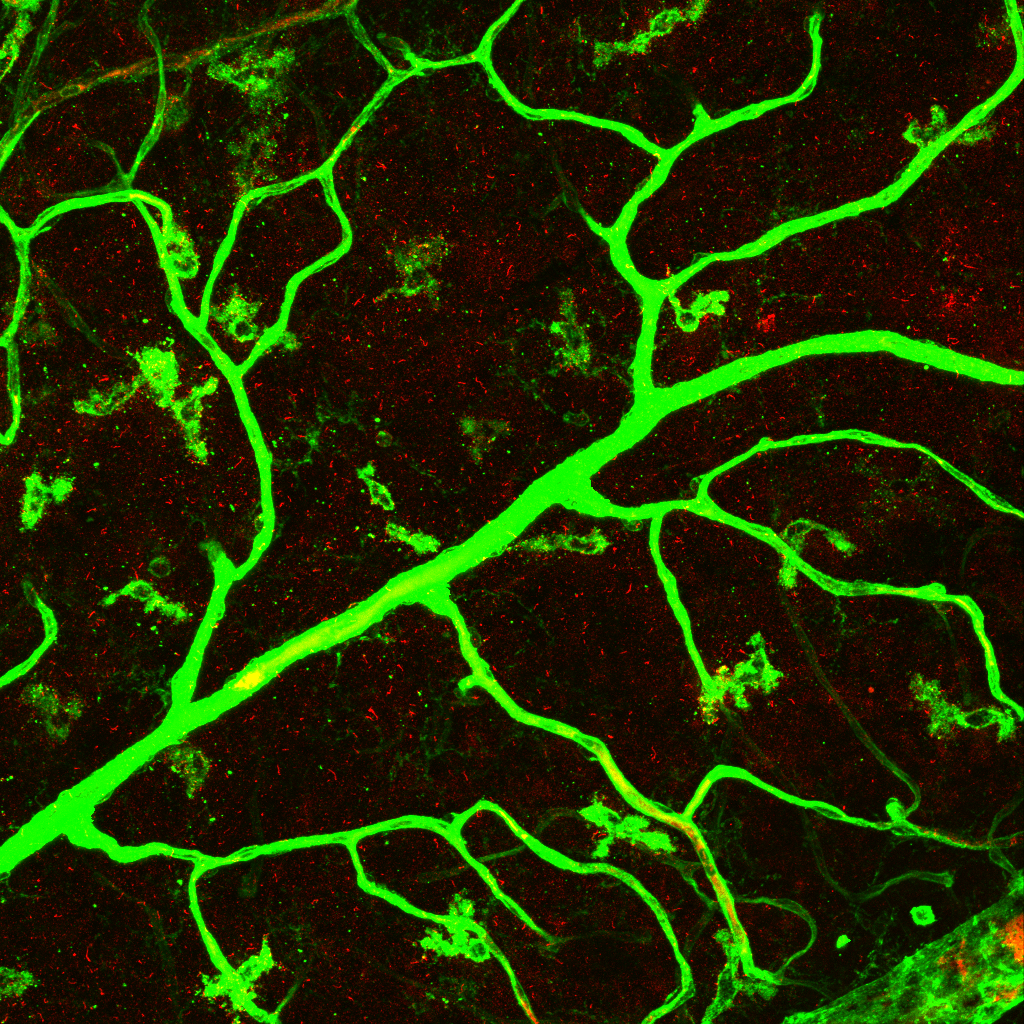

Supplement: Supplementary file 4 — Source data Fig. 2 [file 44321_2026_438_MOESM4_ESM.zip › Figure 2/2G/Astro5559(KO)_IB4_20x-1_Maximum intensity projection.tif]

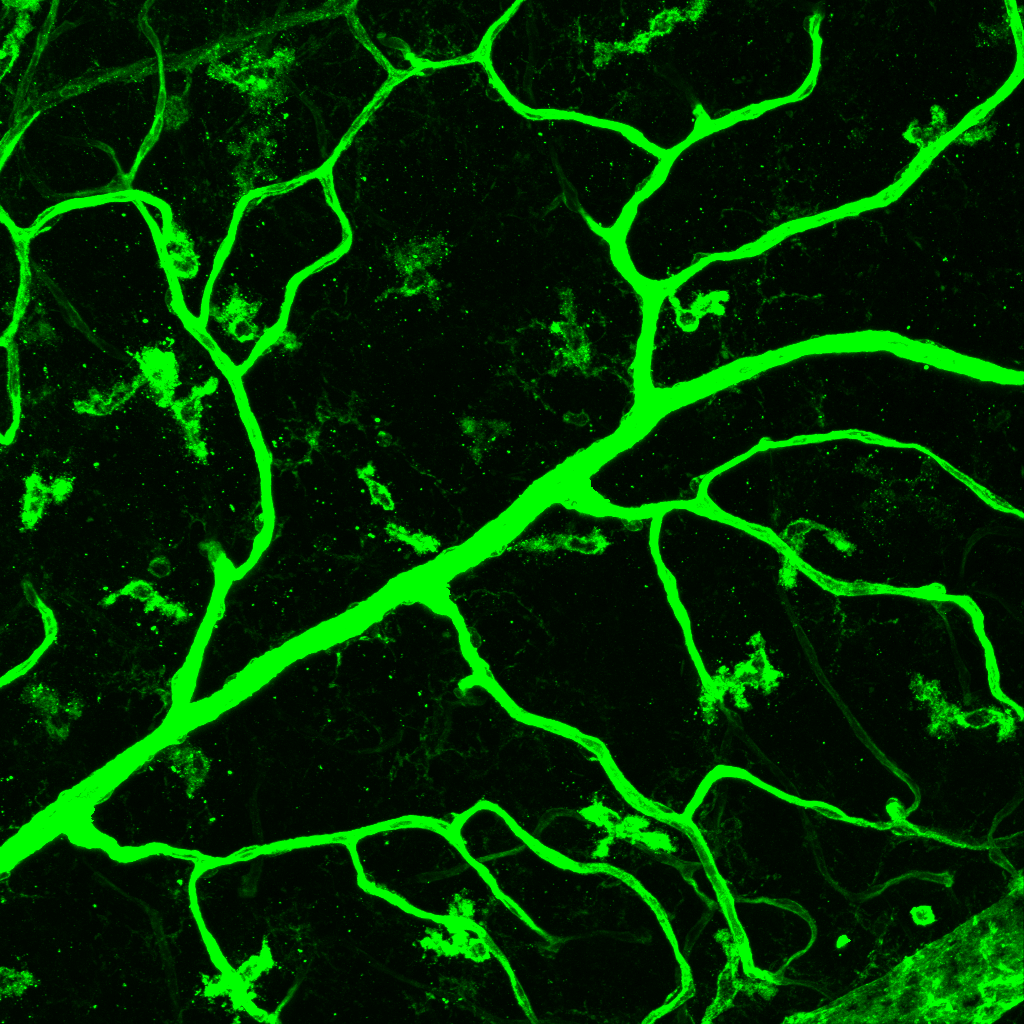

Supplement: Supplementary file 4 — Source data Fig. 2 [file 44321_2026_438_MOESM4_ESM.zip › Figure 2/2G/Astro5559(KO)_merge_Maximum intensity projection.tif]

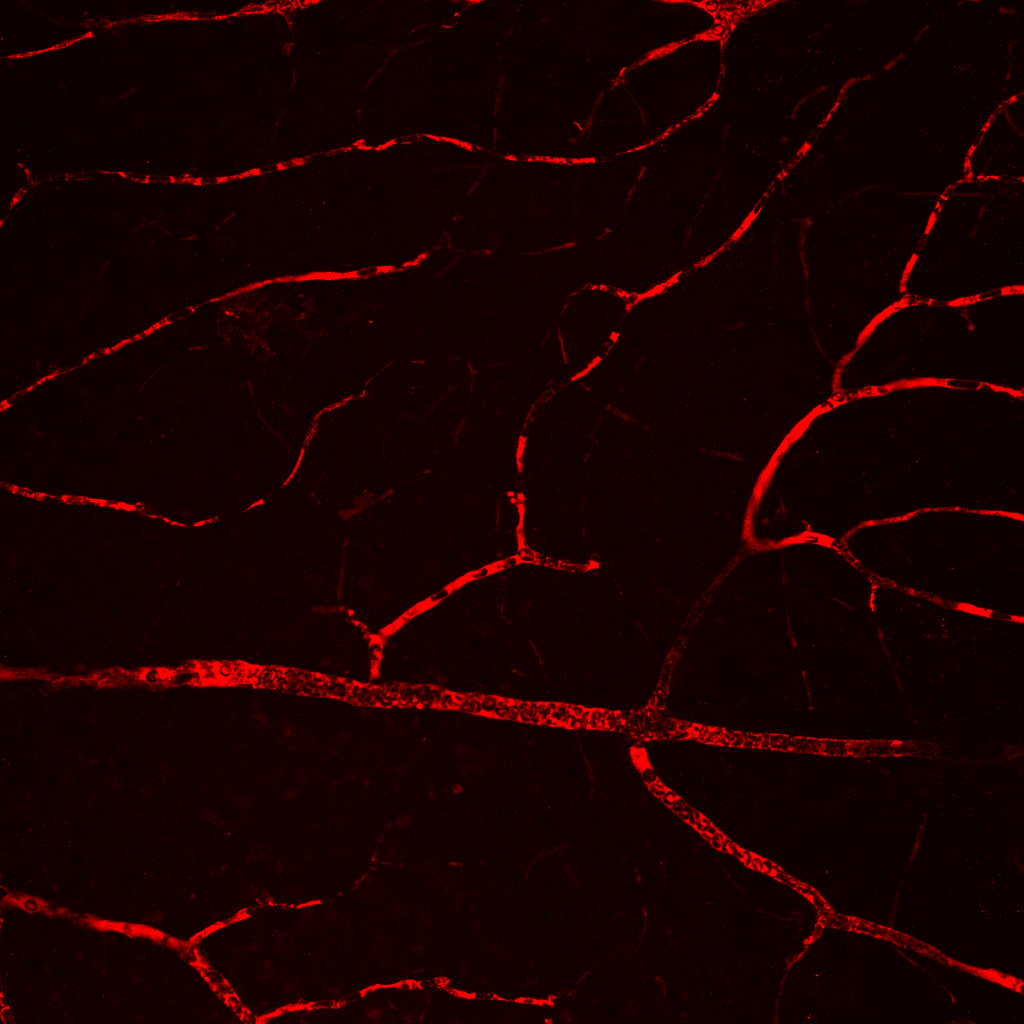

Supplement: Supplementary file 4 — Source data Fig. 2 [file 44321_2026_438_MOESM4_ESM.zip › Figure 2/2G/Astro5560(Ct)_fibrinogen_20x-1_Maximum intensity projection.tif]

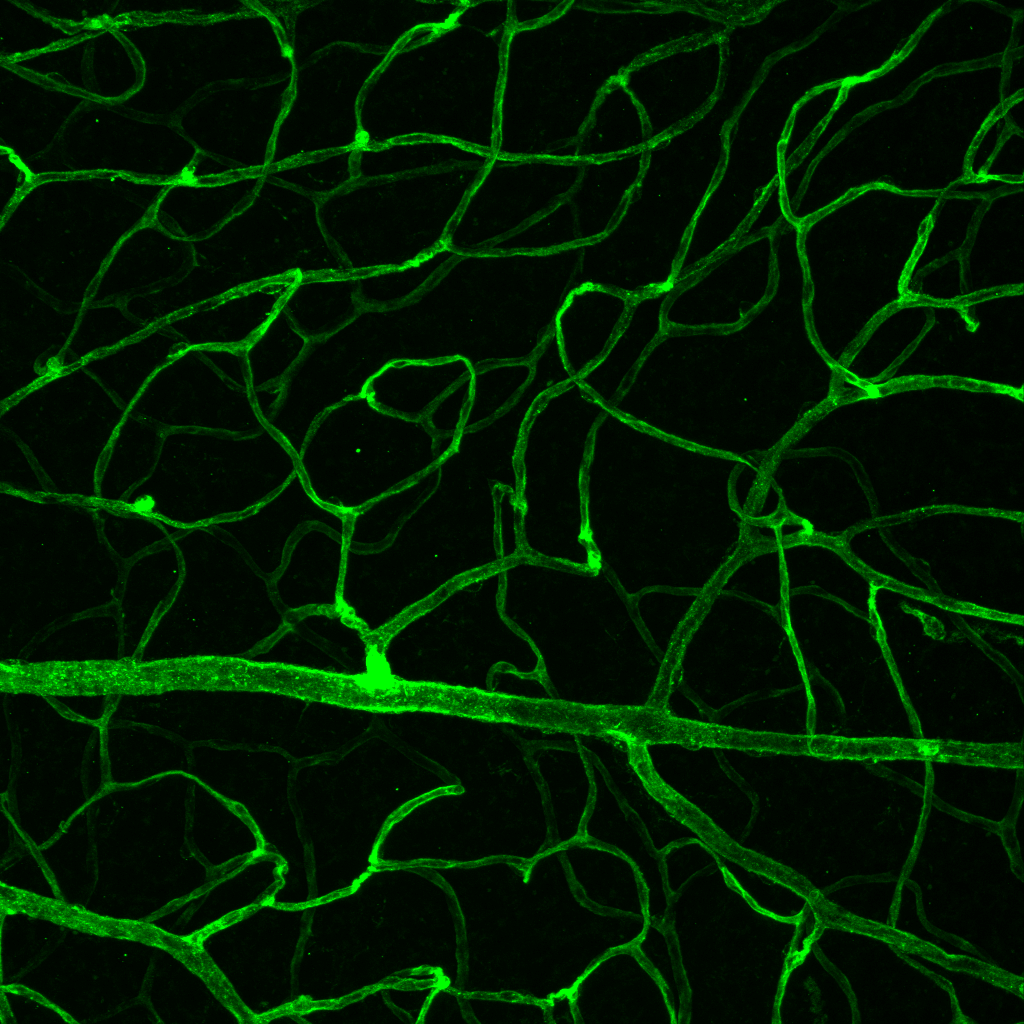

Supplement: Supplementary file 4 — Source data Fig. 2 [file 44321_2026_438_MOESM4_ESM.zip › Figure 2/2G/Astro5560(Ct)_IB4_20x-1_Maximum intensity projection.tif]

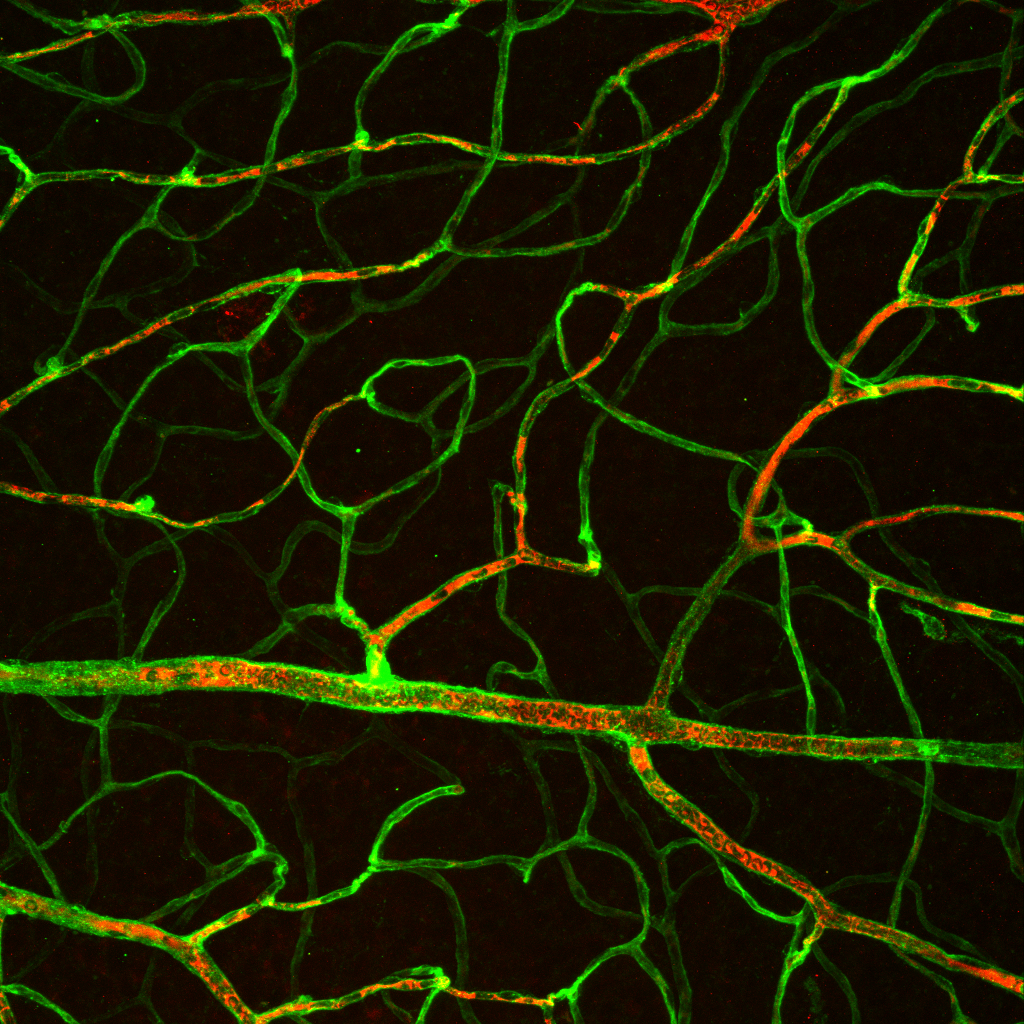

Supplement: Supplementary file 4 — Source data Fig. 2 [file 44321_2026_438_MOESM4_ESM.zip › Figure 2/2G/Astro5560(Ct)_merge_Maximum intensity projection.tif]

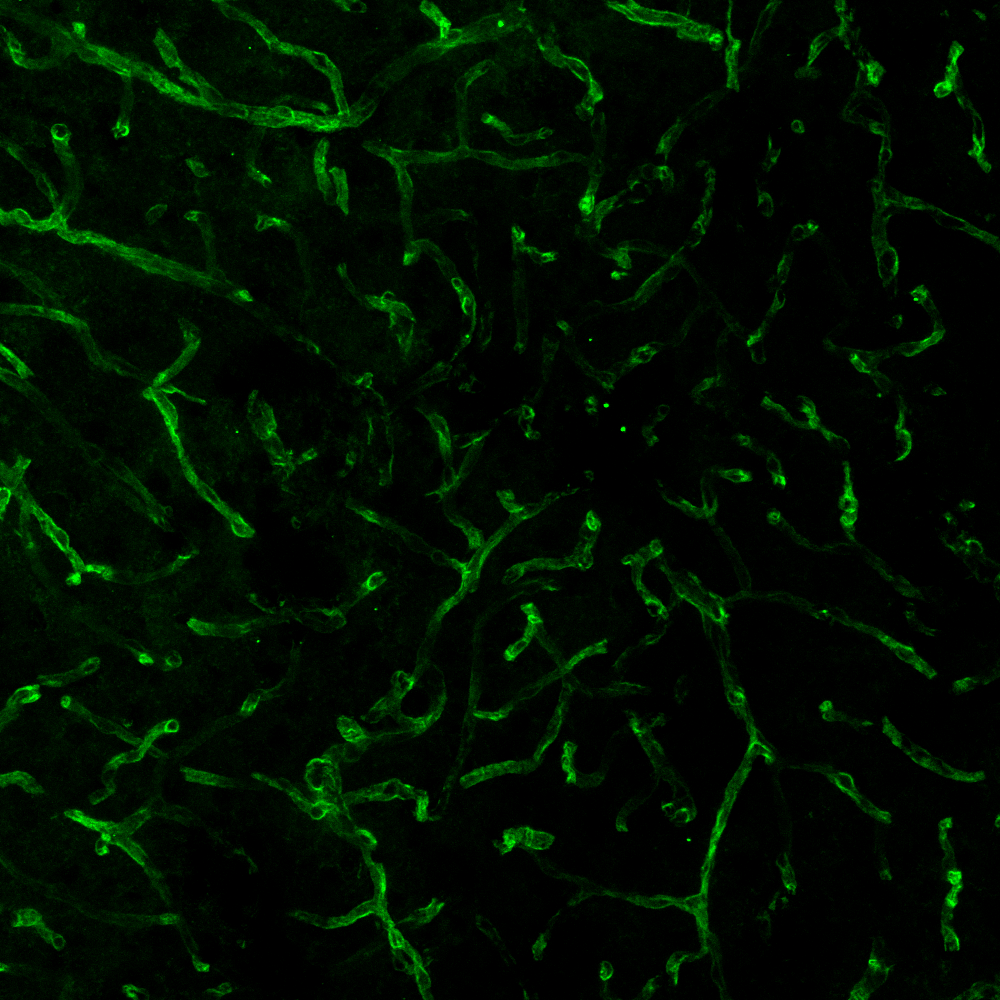

Supplement: Supplementary file 5 — Source data Fig. 3 [file 44321_2026_438_MOESM5_ESM.zip › Figure 3/3A/OIM770(KO)-cropped-aqua4.tif]

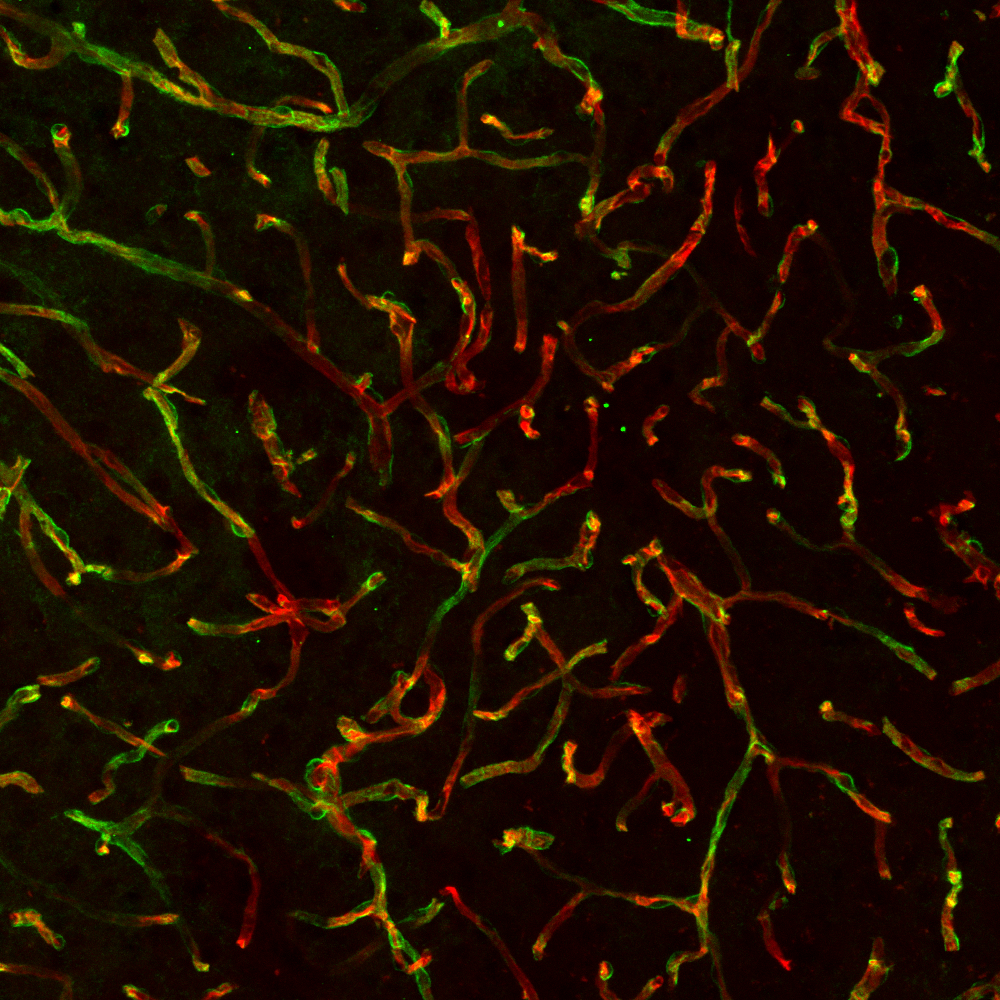

Supplement: Supplementary file 5 — Source data Fig. 3 [file 44321_2026_438_MOESM5_ESM.zip › Figure 3/3A/OIM770(KO)-cropped-merge.tif]

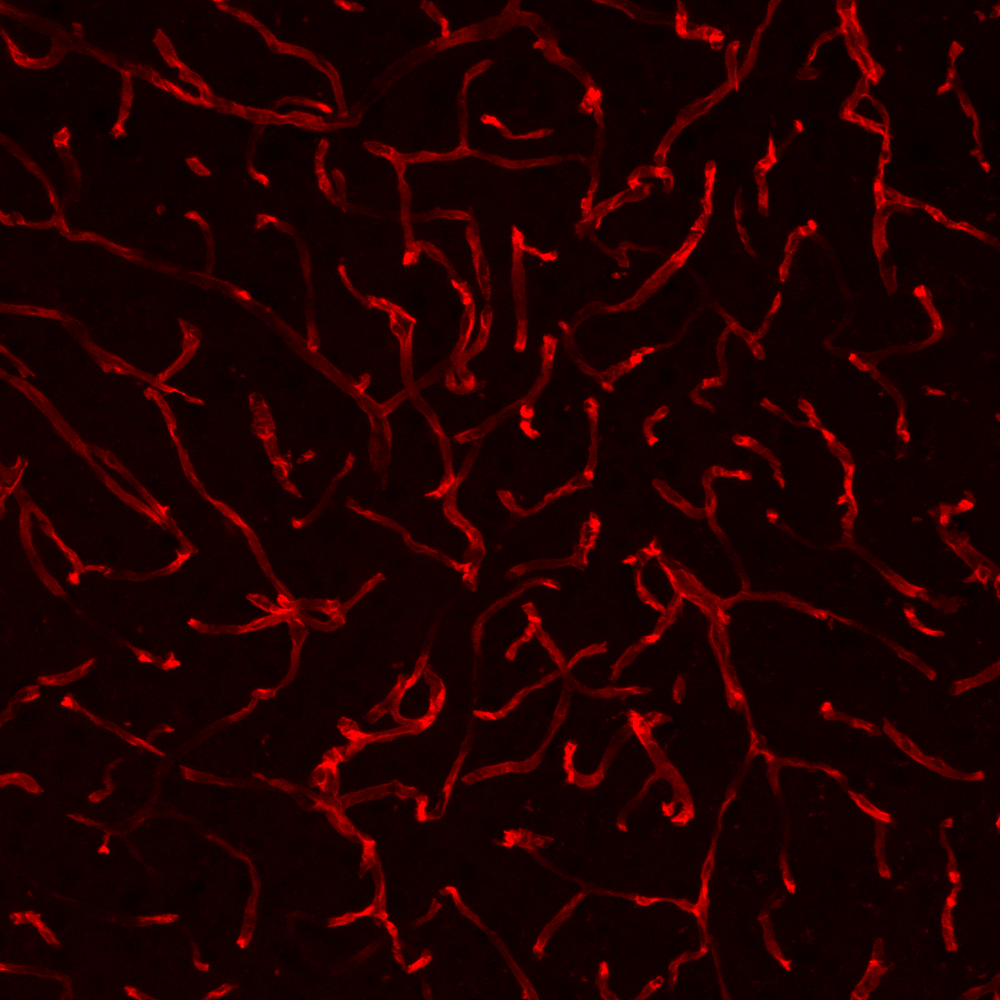

Supplement: Supplementary file 5 — Source data Fig. 3 [file 44321_2026_438_MOESM5_ESM.zip › Figure 3/3A/OIM770(KO)-cropped-podo.tif]

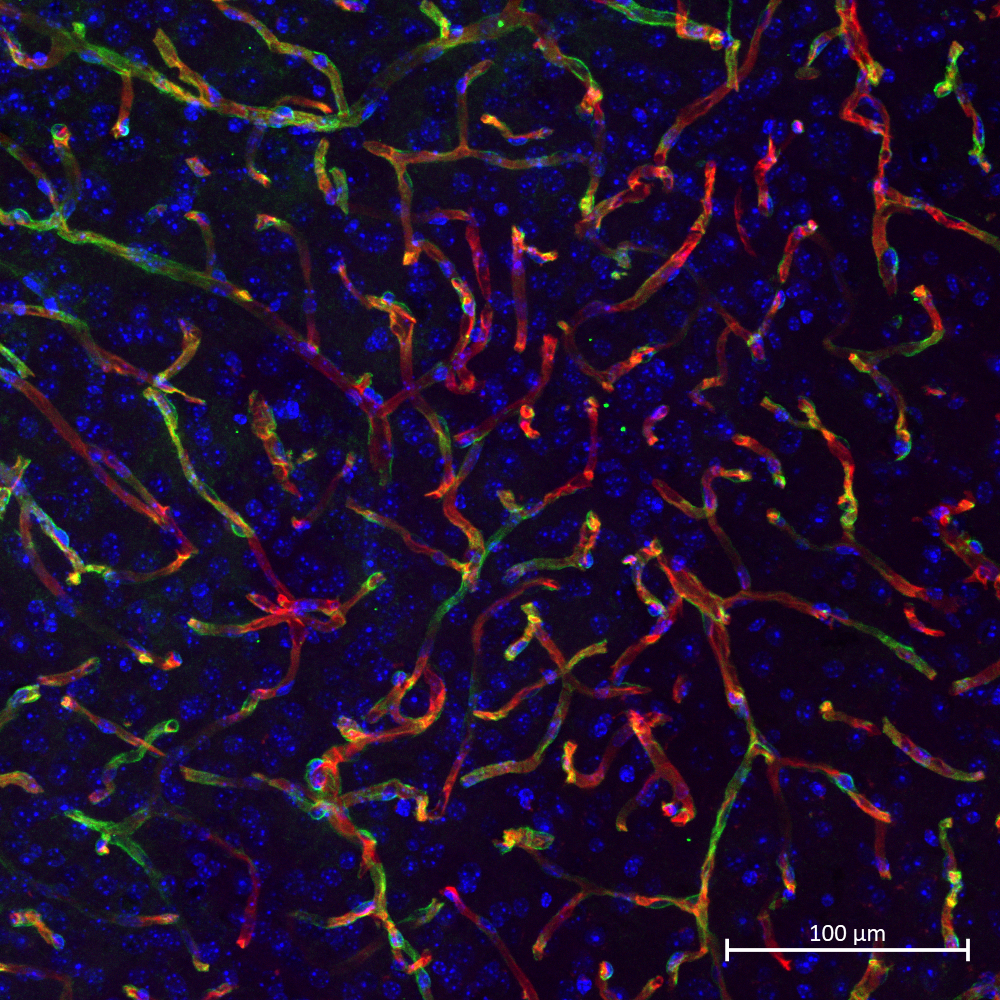

Supplement: Supplementary file 5 — Source data Fig. 3 [file 44321_2026_438_MOESM5_ESM.zip › Figure 3/3A/OIM770(KO)-cropped-scale.tif]

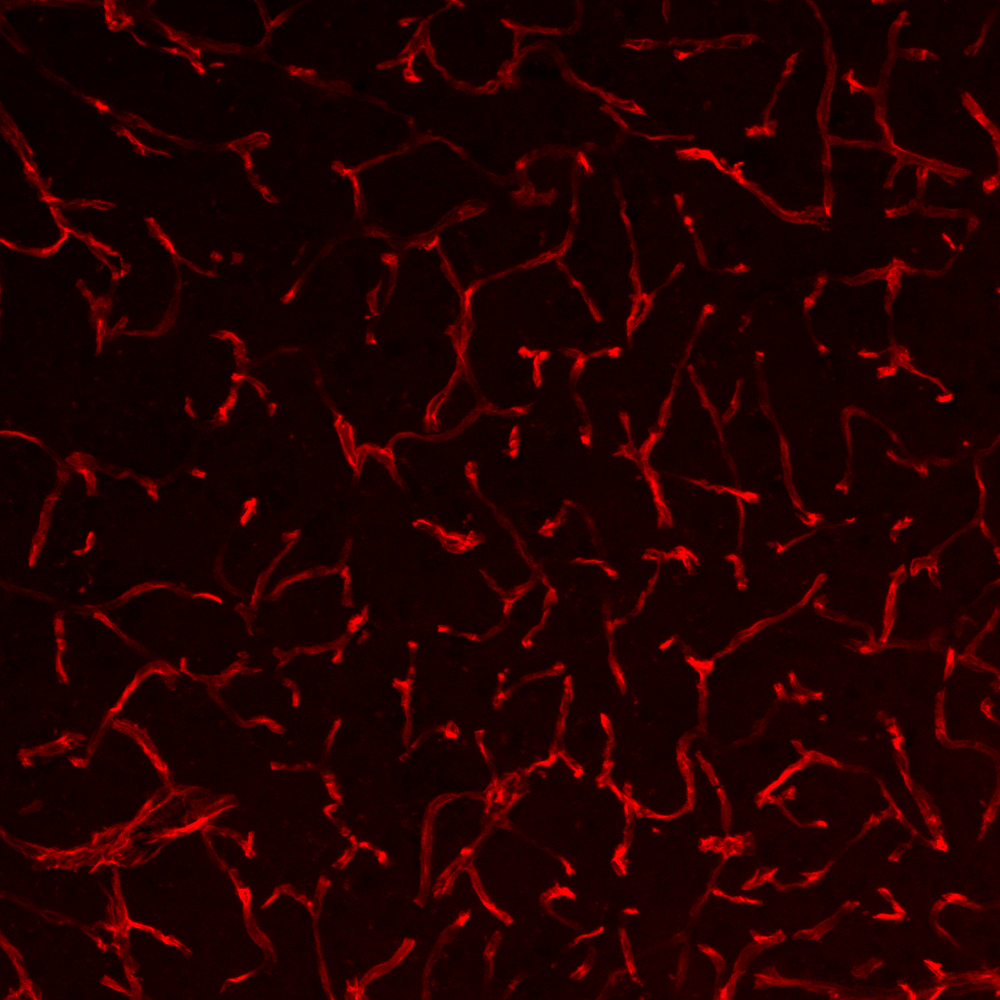

Supplement: Supplementary file 5 — Source data Fig. 3 [file 44321_2026_438_MOESM5_ESM.zip › Figure 3/3A/OIM843(Ct)-cropped--podo.tif]

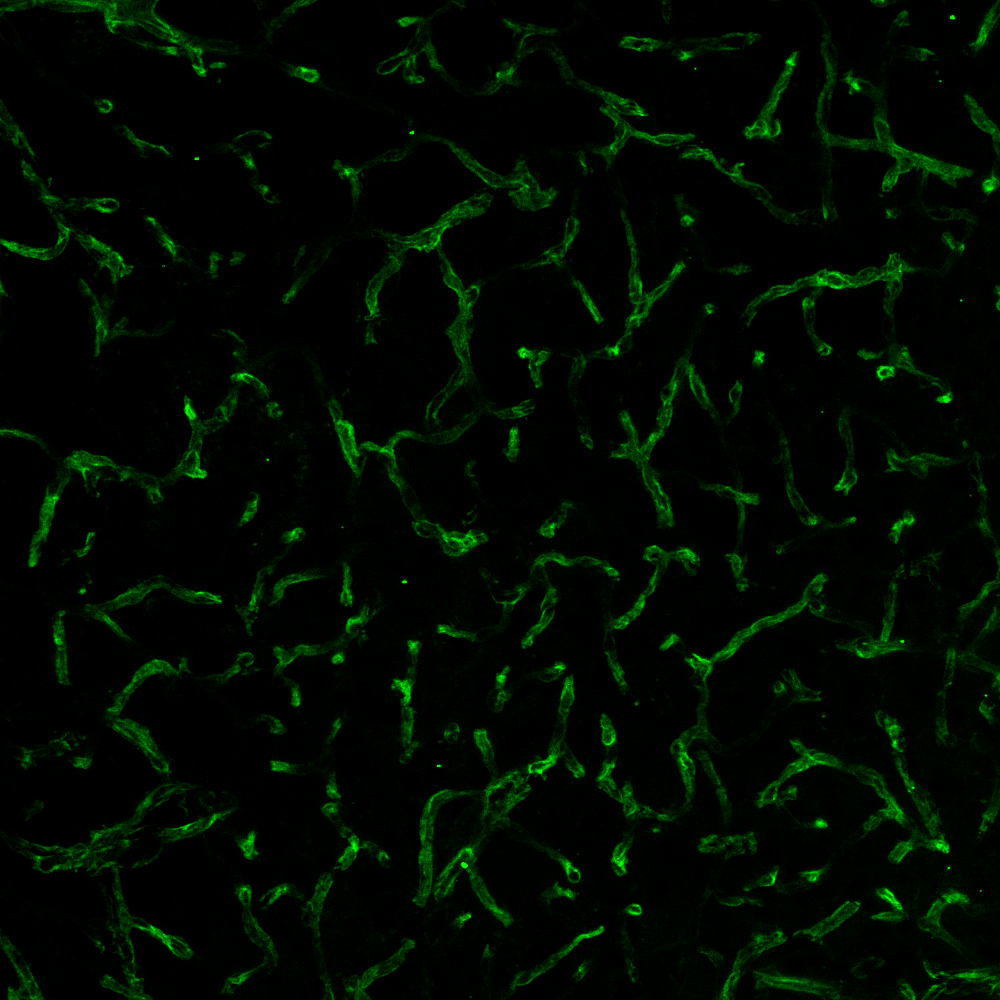

Supplement: Supplementary file 5 — Source data Fig. 3 [file 44321_2026_438_MOESM5_ESM.zip › Figure 3/3A/OIM843(Ct)-cropped-aqua4.tif]

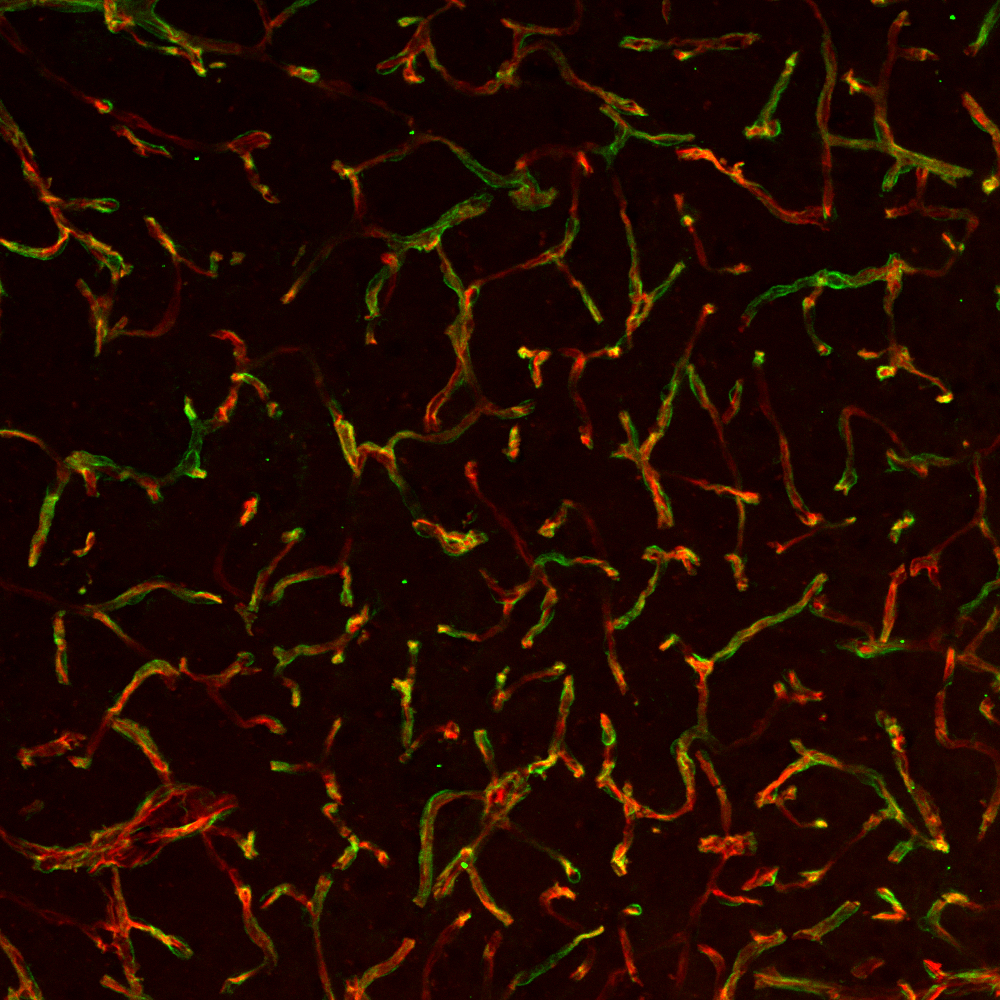

Supplement: Supplementary file 5 — Source data Fig. 3 [file 44321_2026_438_MOESM5_ESM.zip › Figure 3/3A/OIM843(Ct)-cropped-merge.tif]

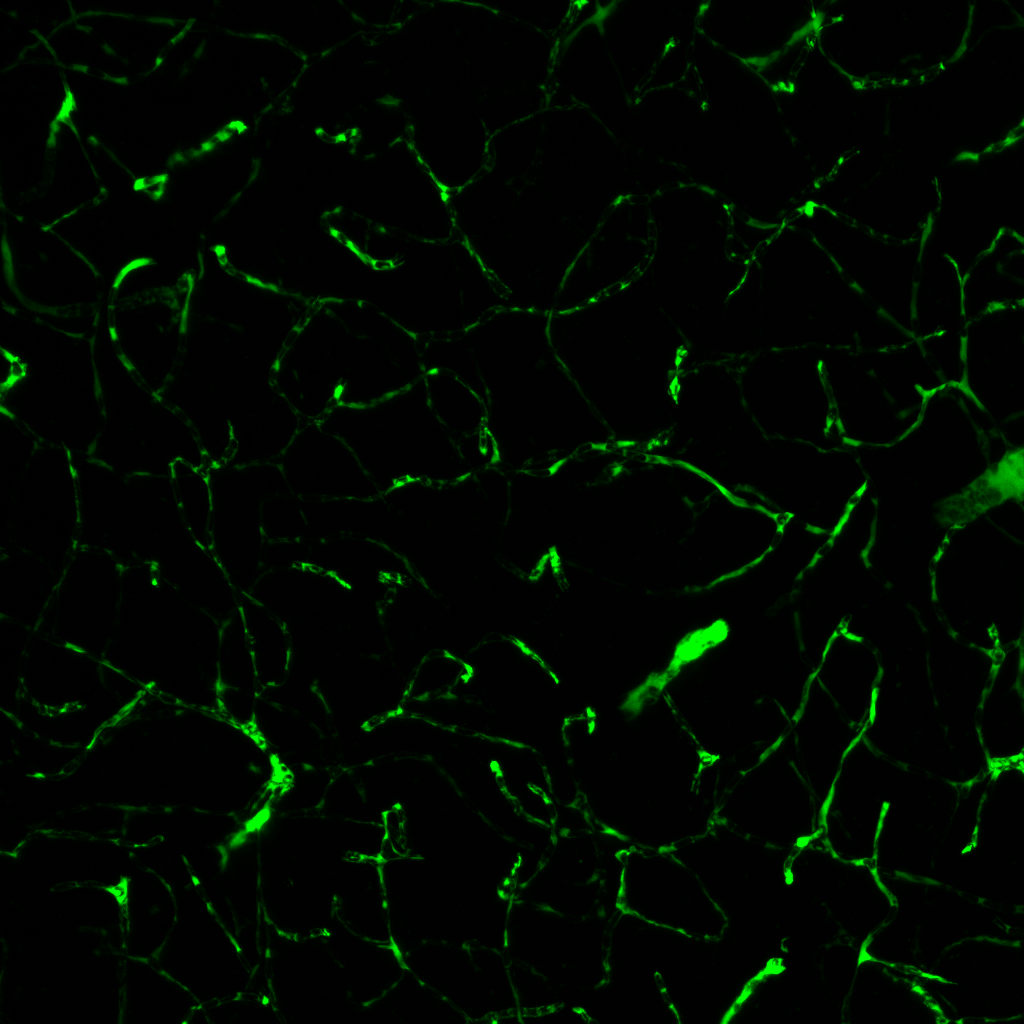

Supplement: Supplementary file 5 — Source data Fig. 3 [file 44321_2026_438_MOESM5_ESM.zip › Figure 3/3C/Astro3190(Ct)_dextran_cortex_20x_1_Maximum intensity projection-.tif]

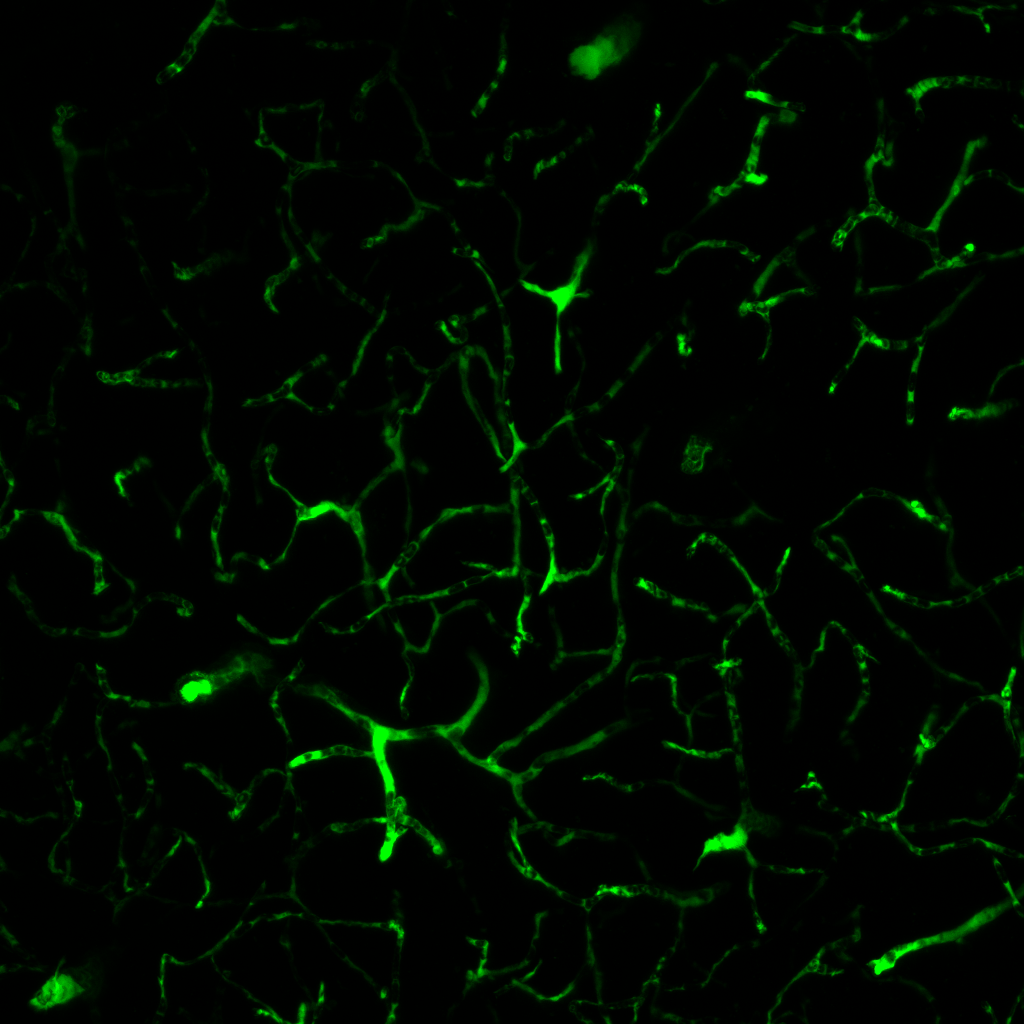

Supplement: Supplementary file 5 — Source data Fig. 3 [file 44321_2026_438_MOESM5_ESM.zip › Figure 3/3C/Astro3193(KO)_dextran_cortex_20x_1_Maximum intensity projection-.tif]

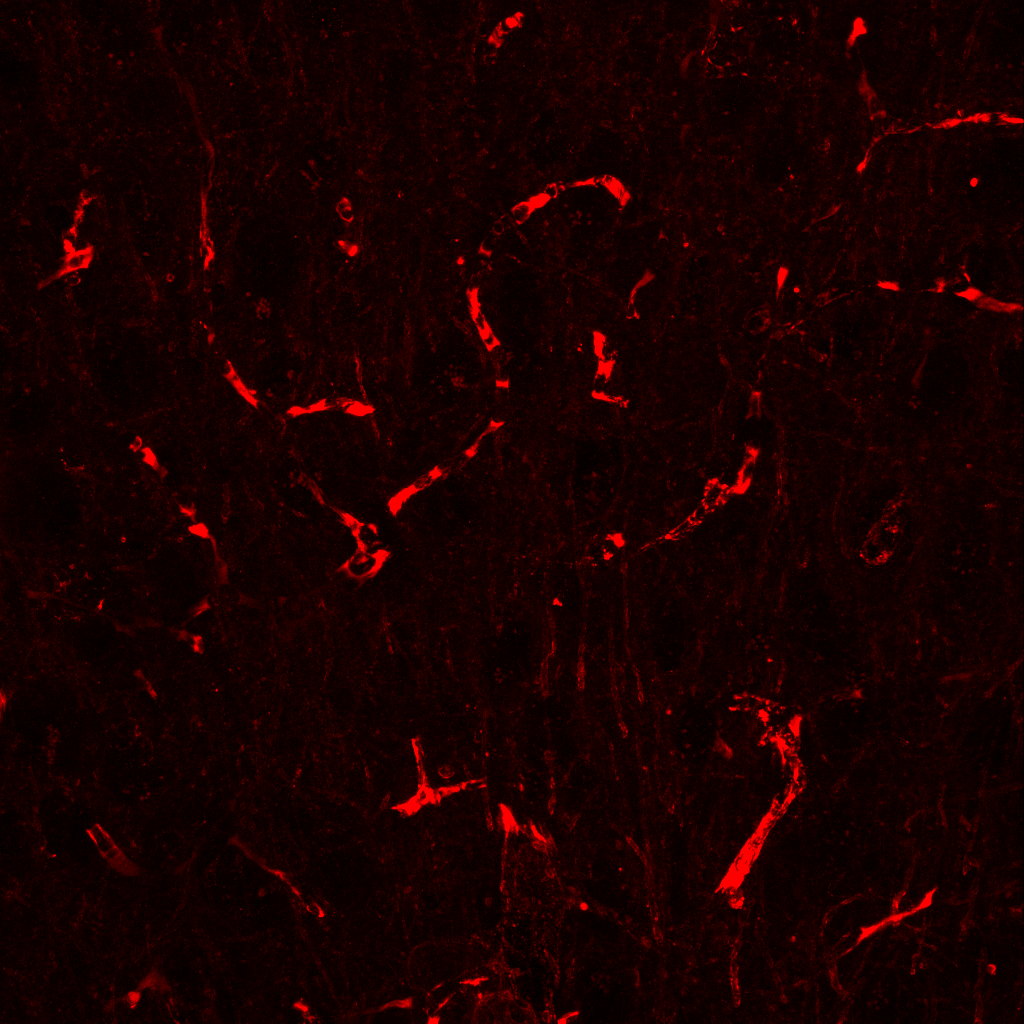

Supplement: Supplementary file 5 — Source data Fig. 3 [file 44321_2026_438_MOESM5_ESM.zip › Figure 3/3E/Astro5822(Ct)_fibrinogen_40x-1_Maximum intensity projection.tif]

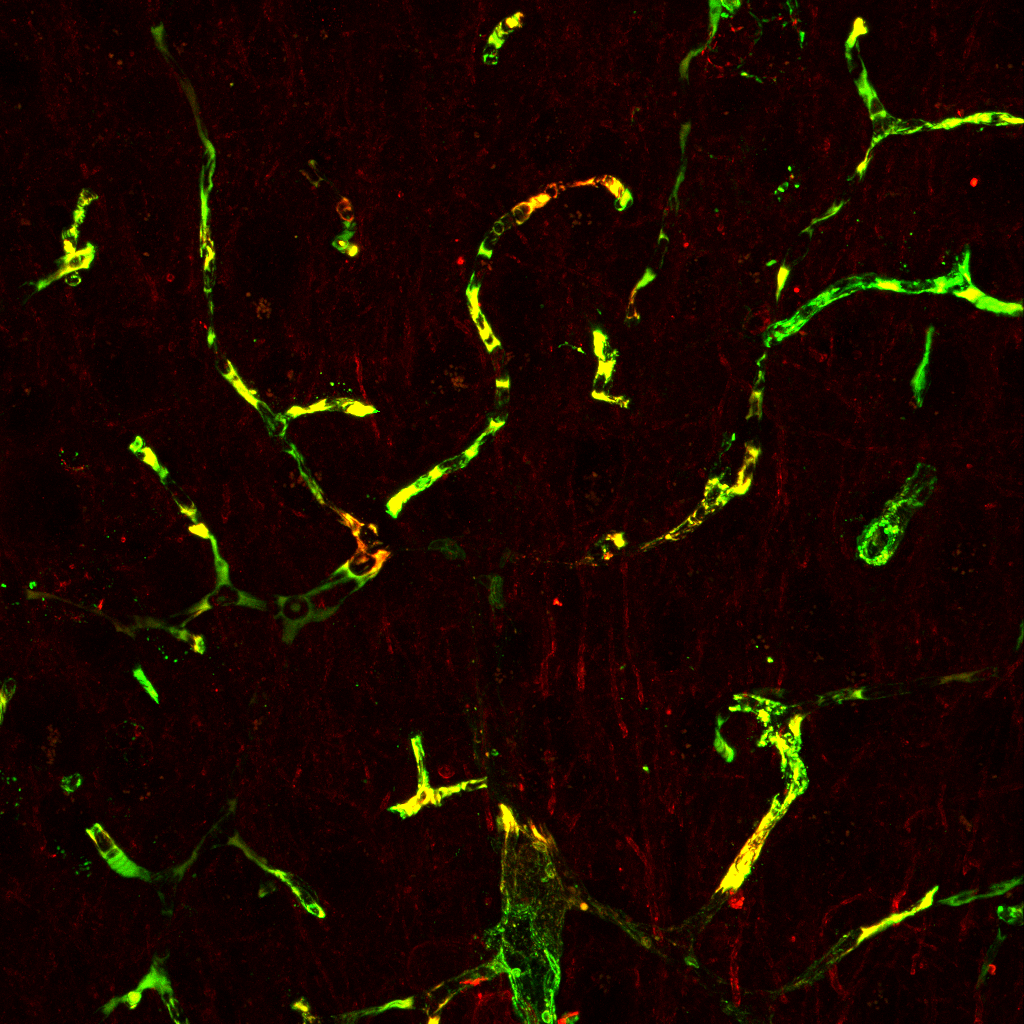

Supplement: Supplementary file 5 — Source data Fig. 3 [file 44321_2026_438_MOESM5_ESM.zip › Figure 3/3E/Astro5822(Ct)_merge_Maximum intensity projection.tif]

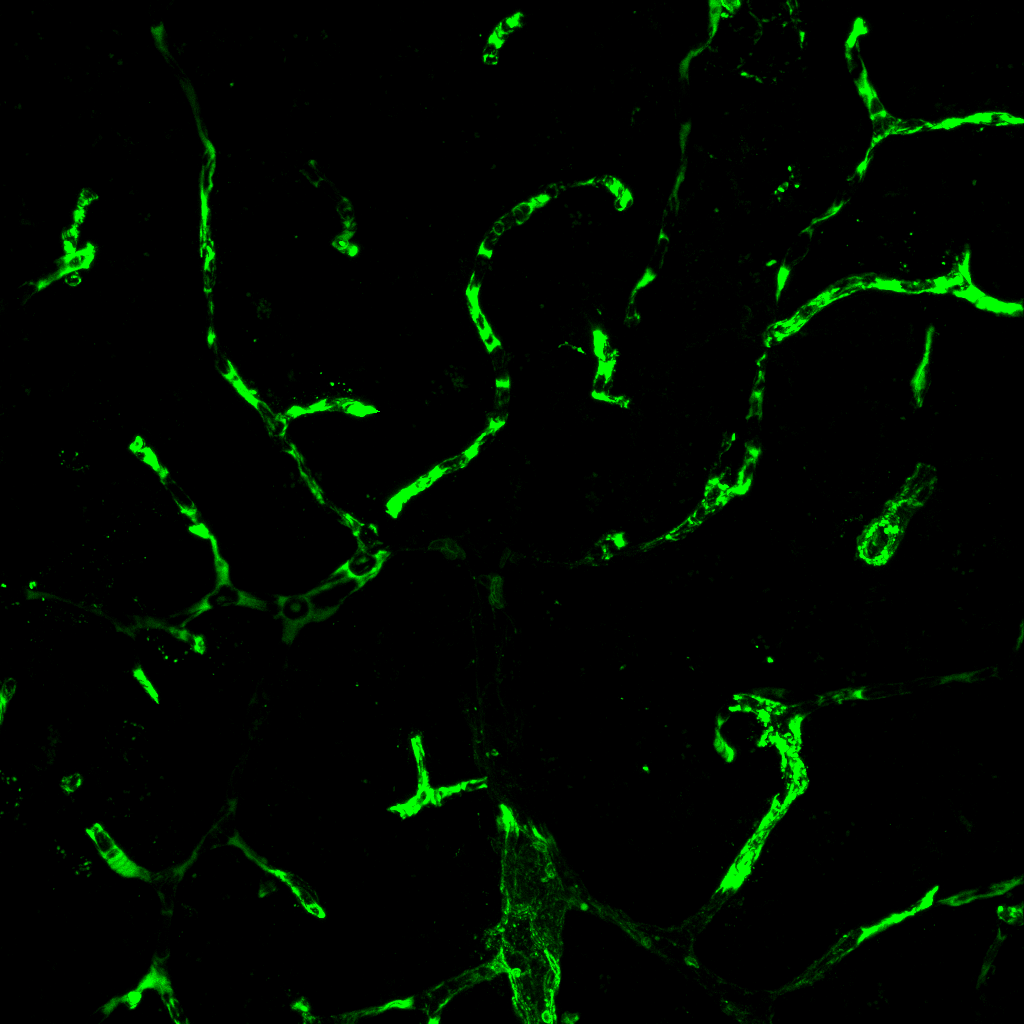

Supplement: Supplementary file 5 — Source data Fig. 3 [file 44321_2026_438_MOESM5_ESM.zip › Figure 3/3E/Astro5822(Ct)_podo_40x-1_Maximum intensity projection.tif]

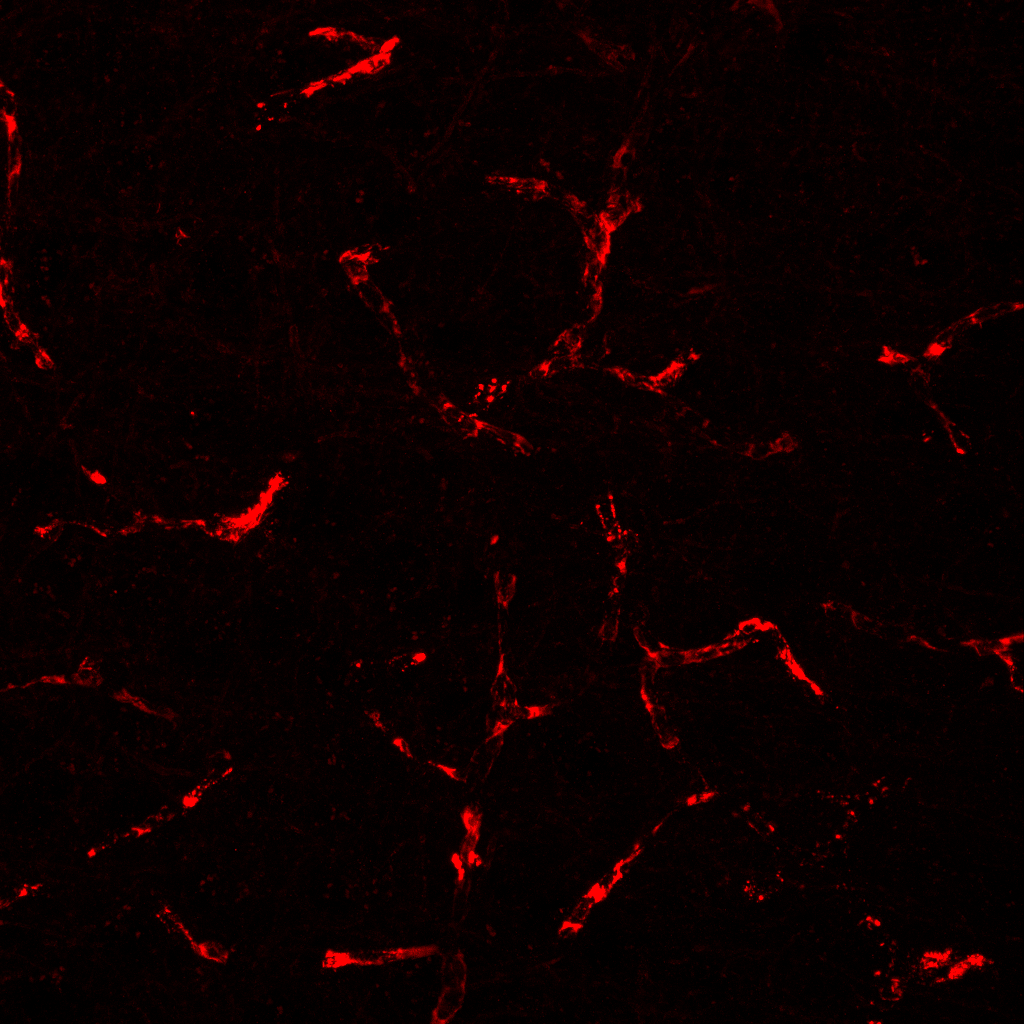

Supplement: Supplementary file 5 — Source data Fig. 3 [file 44321_2026_438_MOESM5_ESM.zip › Figure 3/3E/Astro5865(KO)_fibrinogen_40x-1_Maximum intensity projection.tif]

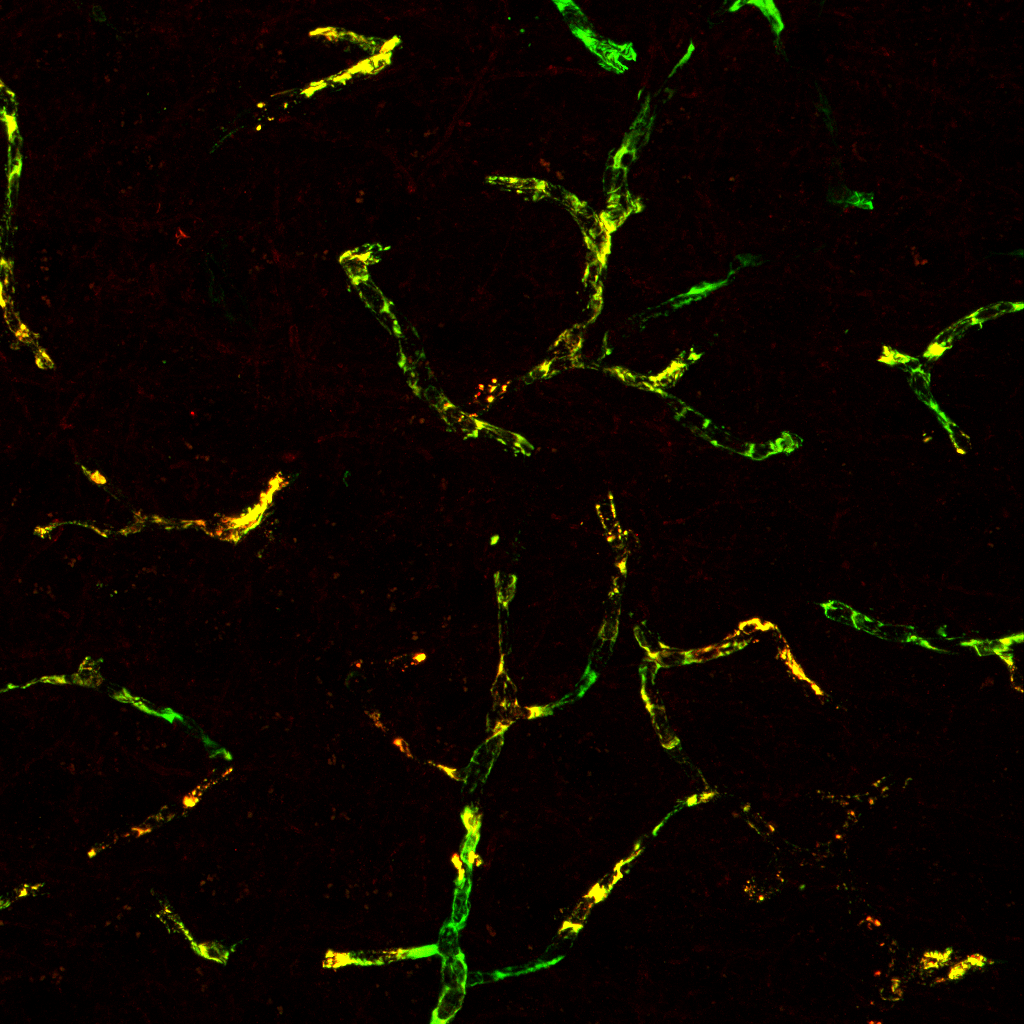

Supplement: Supplementary file 5 — Source data Fig. 3 [file 44321_2026_438_MOESM5_ESM.zip › Figure 3/3E/Astro5865(KO)_merge_Maximum intensity projection.tif]

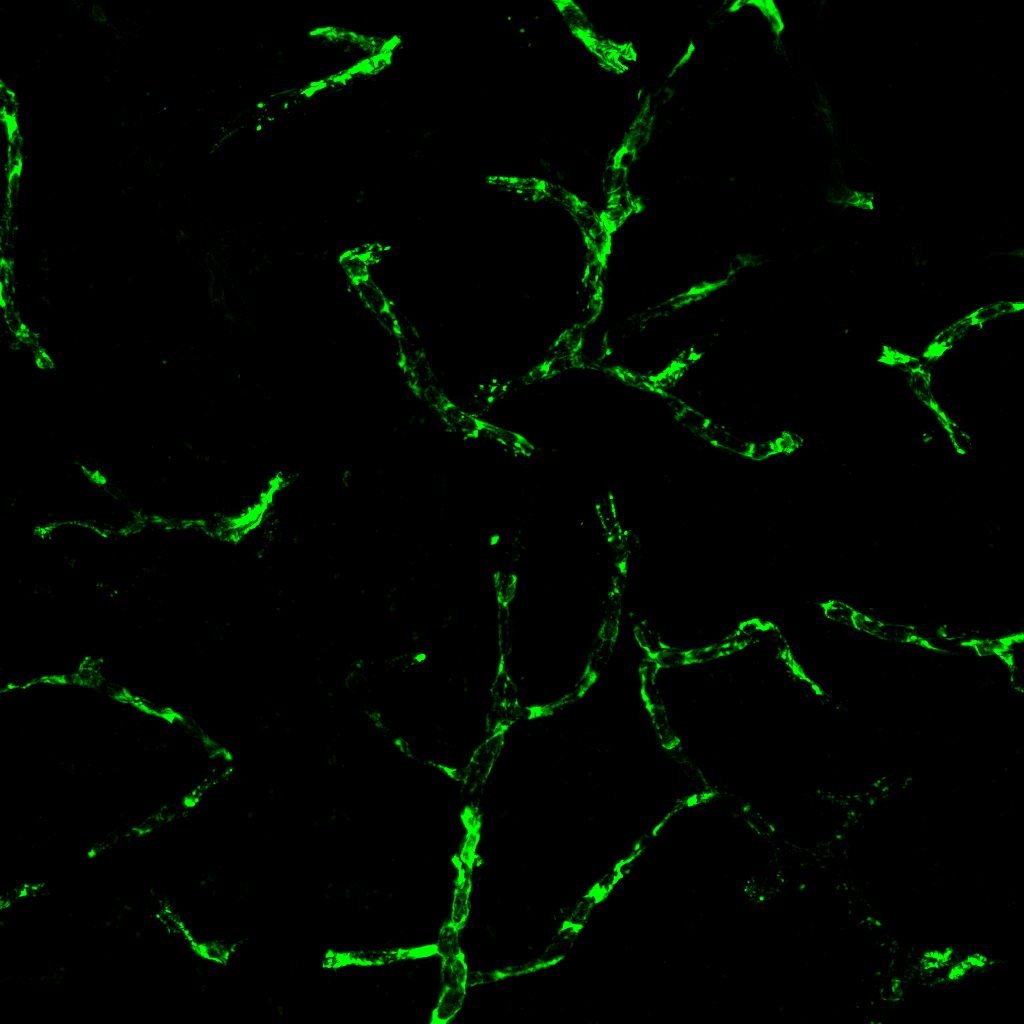

Supplement: Supplementary file 5 — Source data Fig. 3 [file 44321_2026_438_MOESM5_ESM.zip › Figure 3/3E/Astro5865(KO)_podo_40x-1_Maximum intensity projection.tif]

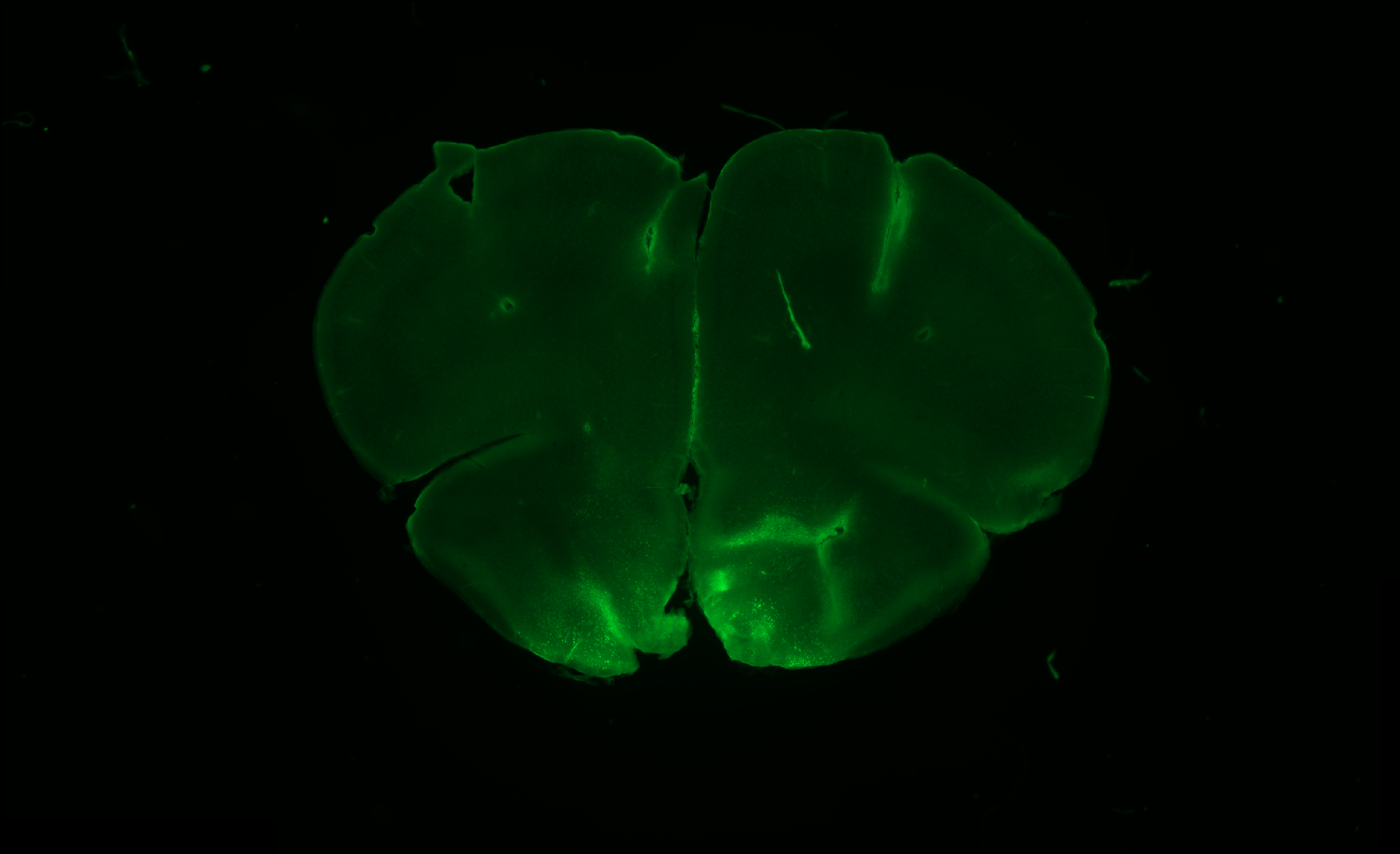

Supplement: Supplementary file 6 — Source data Fig. 4 [file 44321_2026_438_MOESM6_ESM.zip › Figure 4/4A/Enhanced TIF Control/1200_1_Enchanced_Green.tif]

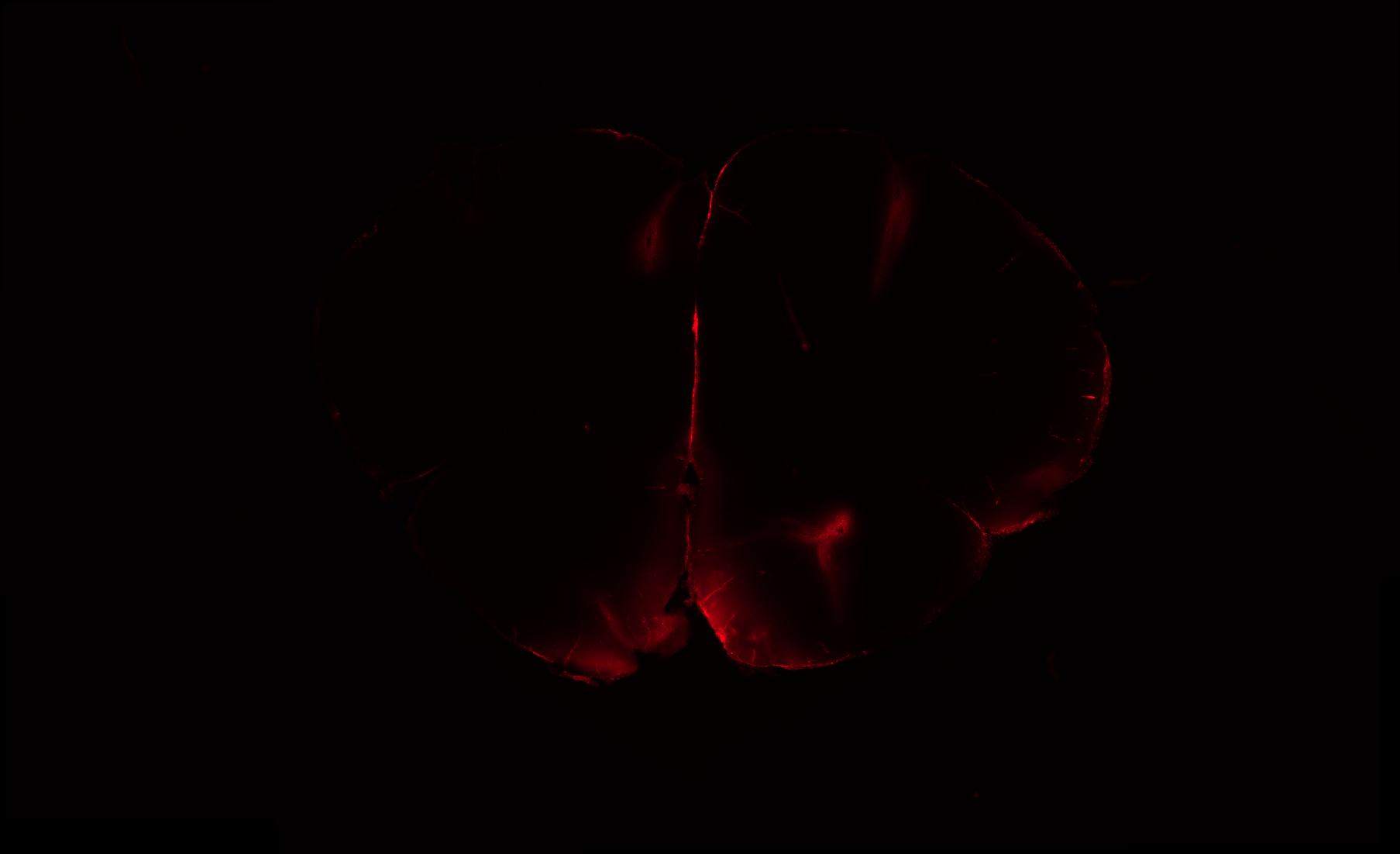

Supplement: Supplementary file 6 — Source data Fig. 4 [file 44321_2026_438_MOESM6_ESM.zip › Figure 4/4A/Enhanced TIF Control/1200_1_Enchanced_Red.tif]

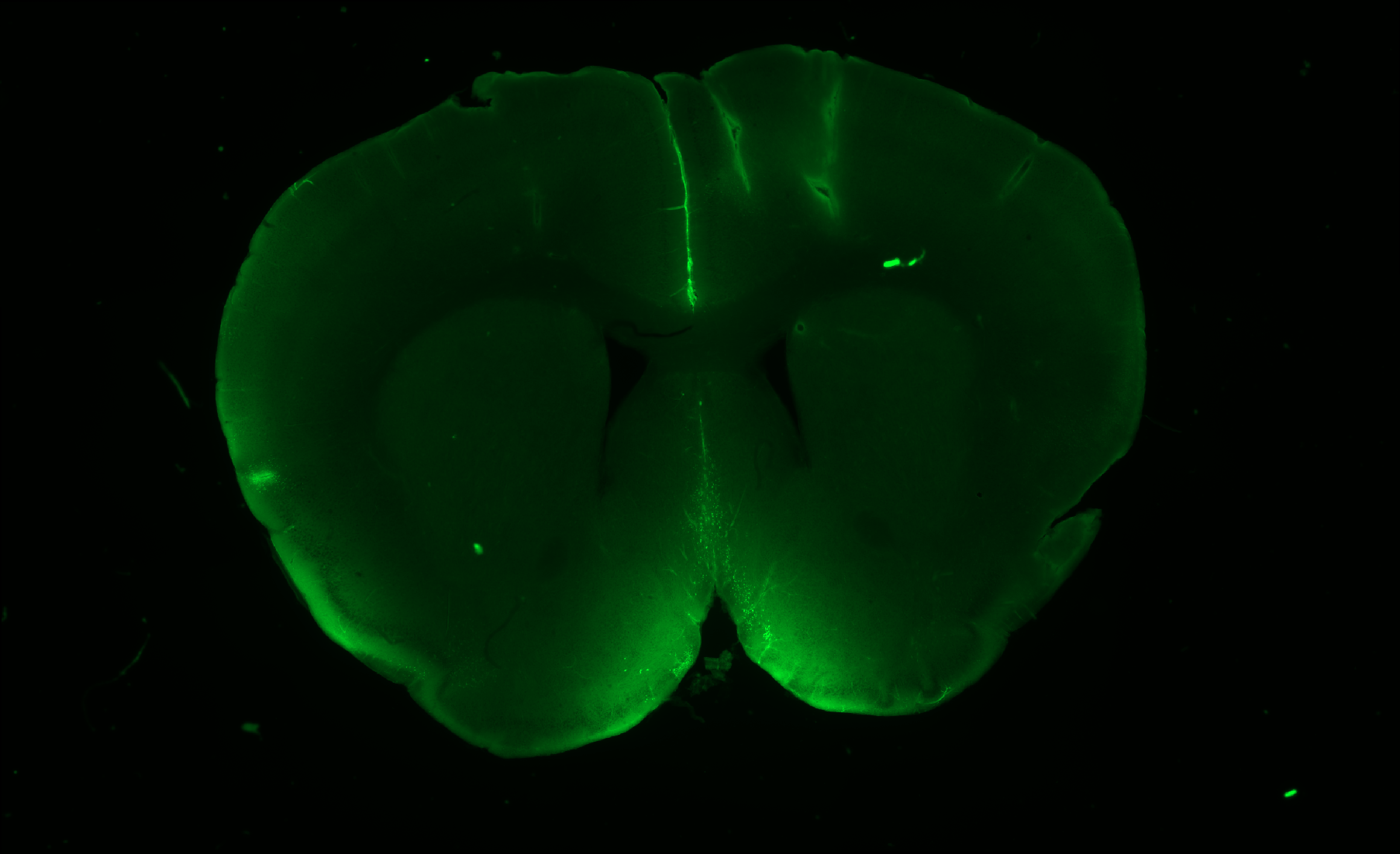

Supplement: Supplementary file 6 — Source data Fig. 4 [file 44321_2026_438_MOESM6_ESM.zip › Figure 4/4A/Enhanced TIF Control/1200_2_Enchanced_Green.tif]

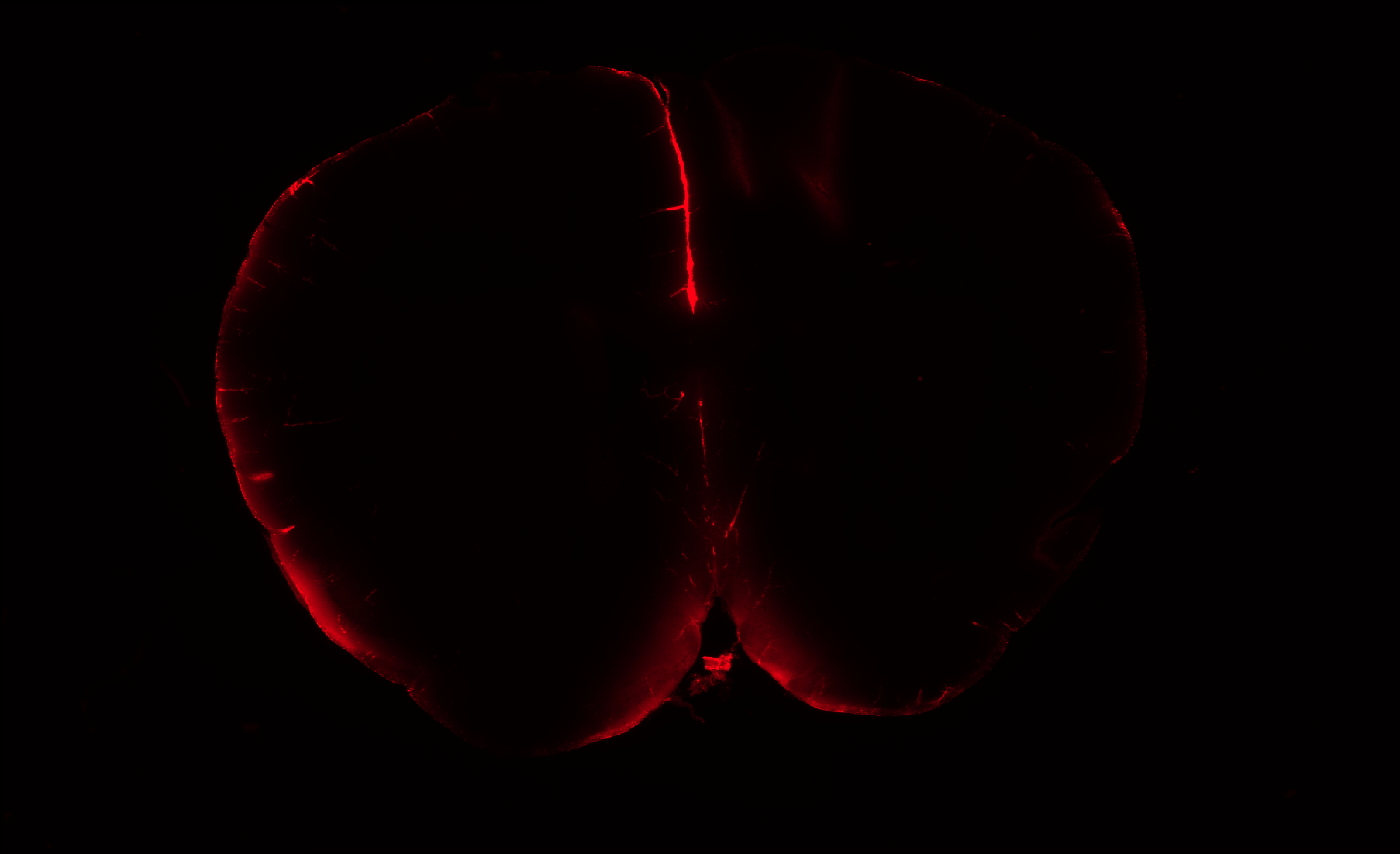

Supplement: Supplementary file 6 — Source data Fig. 4 [file 44321_2026_438_MOESM6_ESM.zip › Figure 4/4A/Enhanced TIF Control/1200_2_Enchanced_Red.tif]

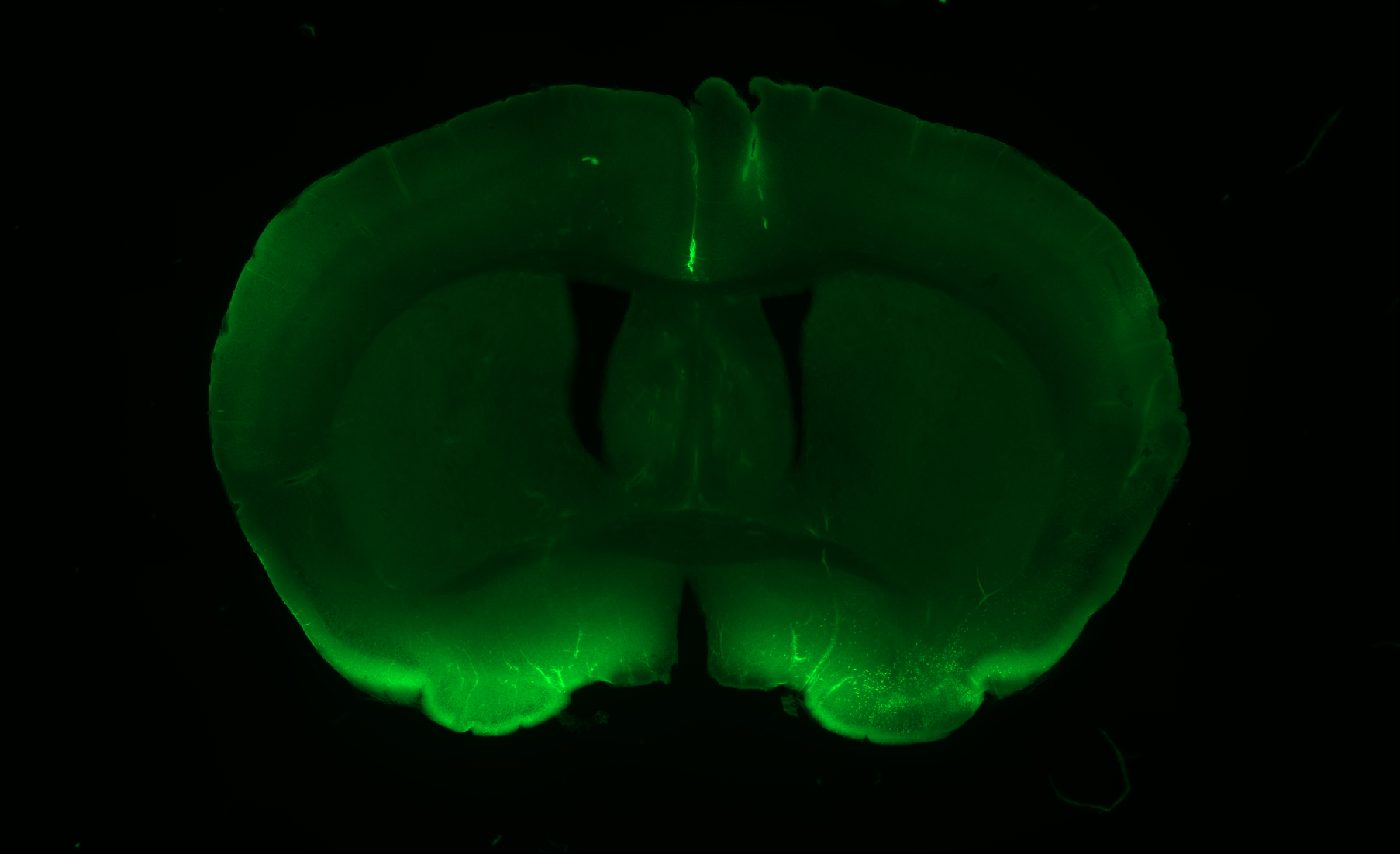

Supplement: Supplementary file 6 — Source data Fig. 4 [file 44321_2026_438_MOESM6_ESM.zip › Figure 4/4A/Enhanced TIF Control/1200_3_Enchanced_Green.tif]

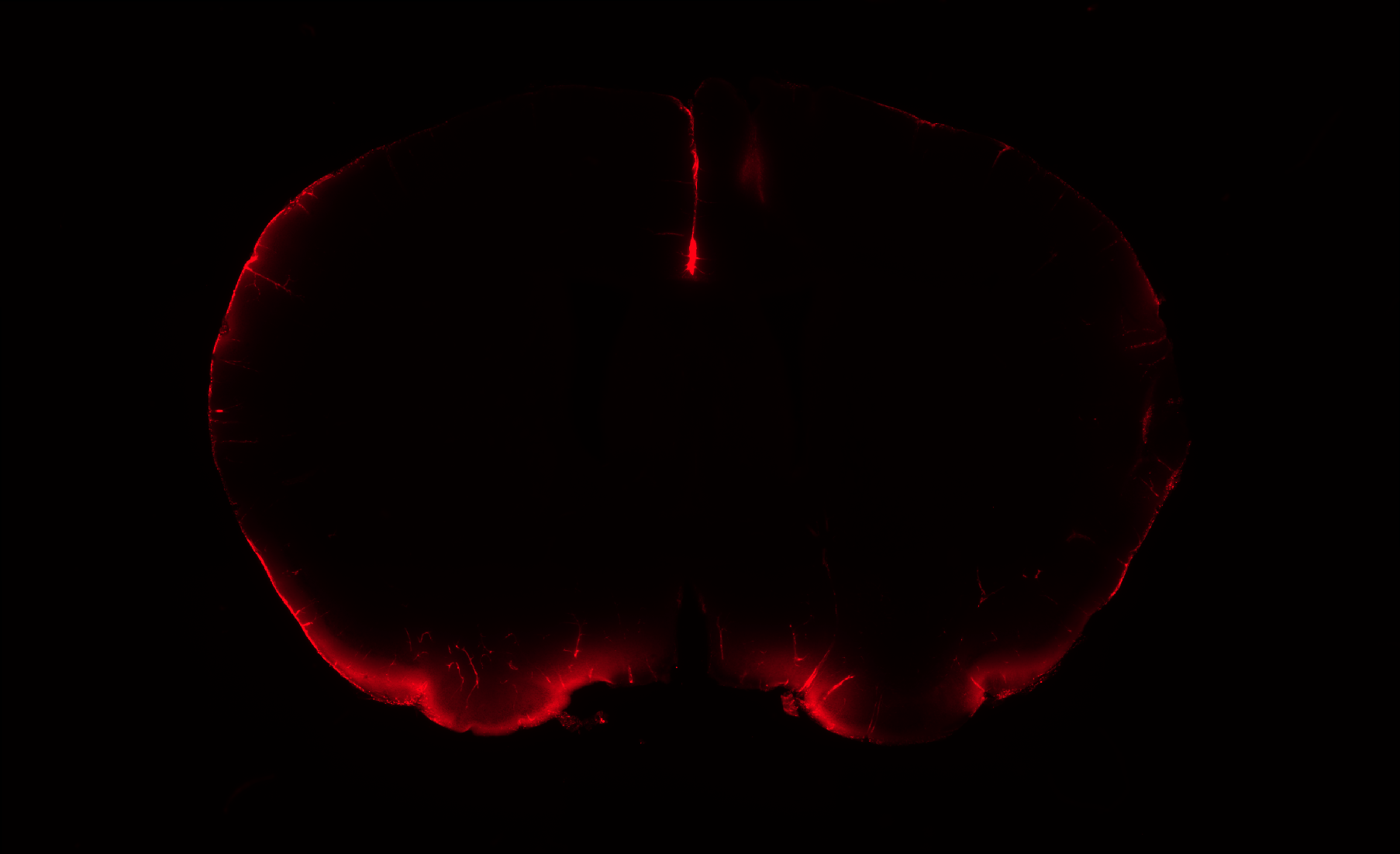

Supplement: Supplementary file 6 — Source data Fig. 4 [file 44321_2026_438_MOESM6_ESM.zip › Figure 4/4A/Enhanced TIF Control/1200_3_Enchanced_Red.tif]

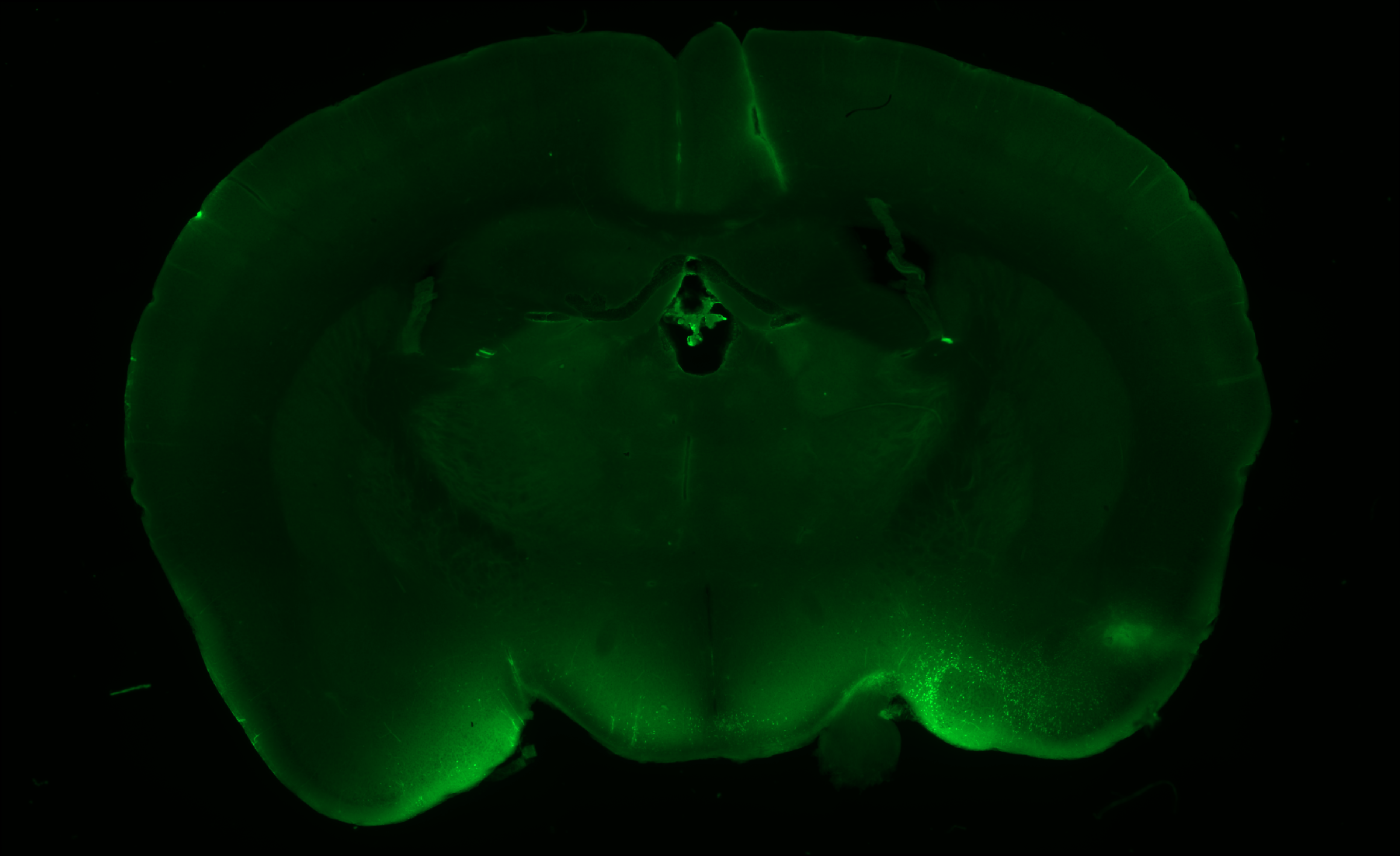

Supplement: Supplementary file 6 — Source data Fig. 4 [file 44321_2026_438_MOESM6_ESM.zip › Figure 4/4A/Enhanced TIF Control/1200_4_Enchanced_Green.tif]

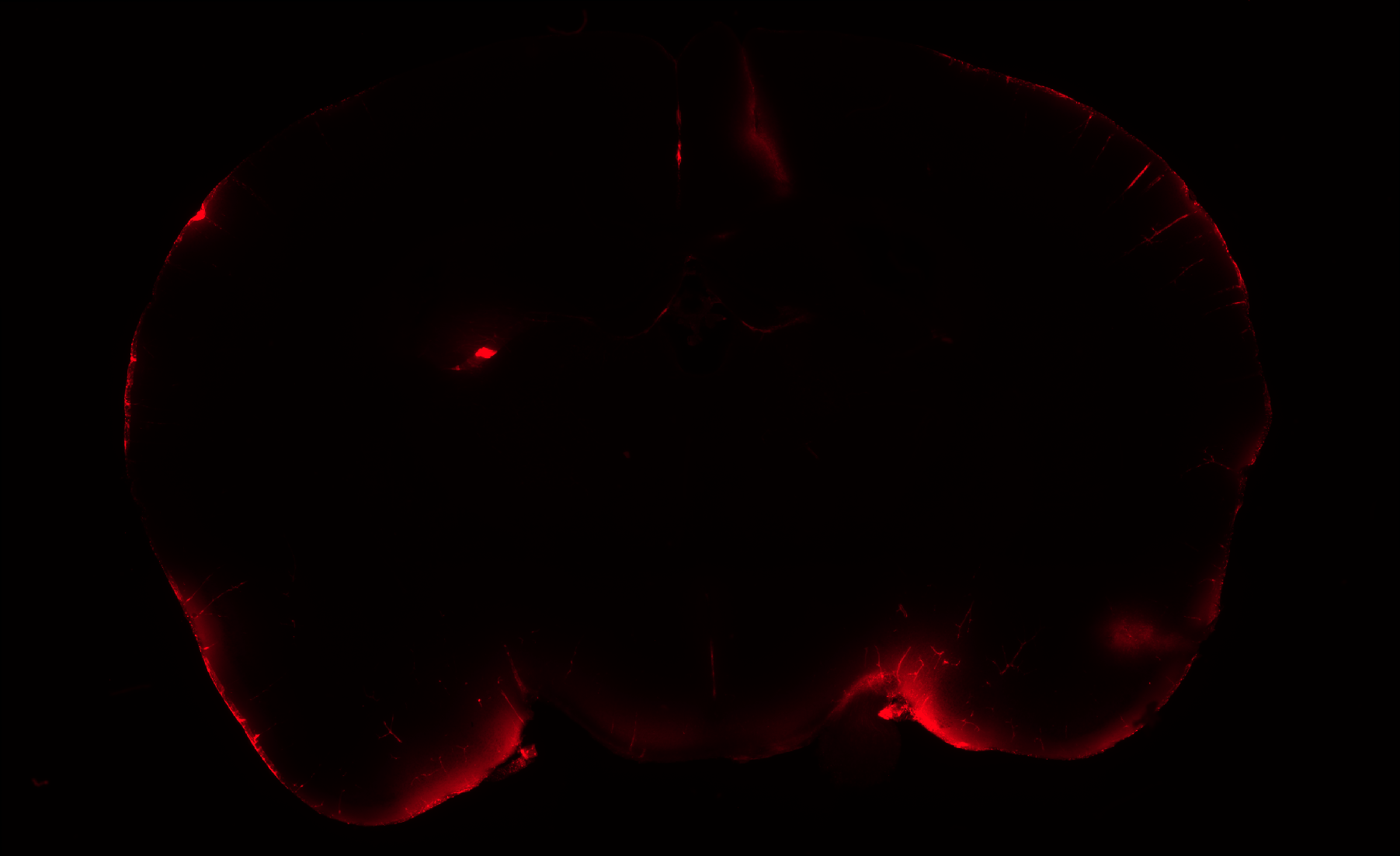

Supplement: Supplementary file 6 — Source data Fig. 4 [file 44321_2026_438_MOESM6_ESM.zip › Figure 4/4A/Enhanced TIF Control/1200_4_Enchanced_Red.tif]

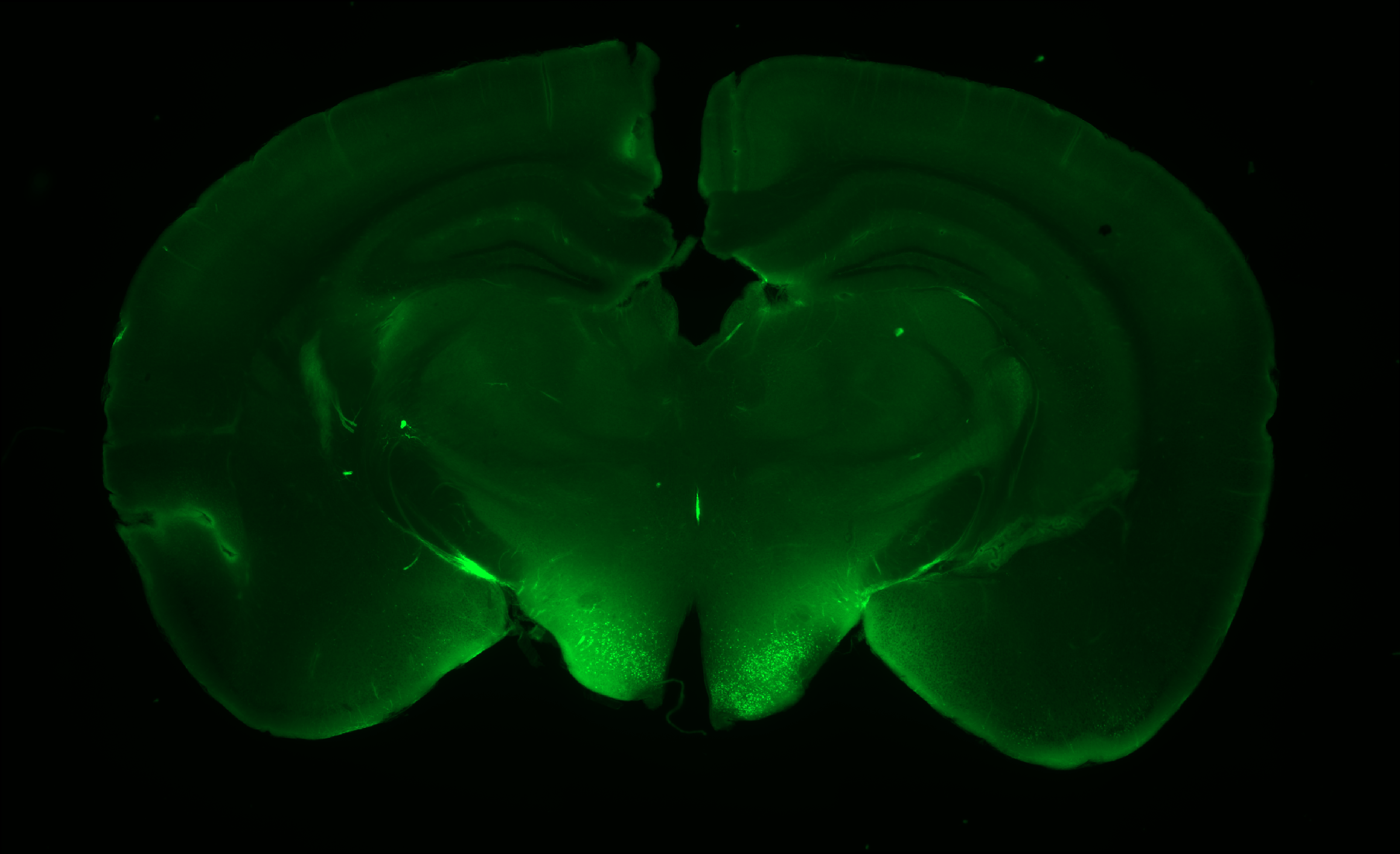

Supplement: Supplementary file 6 — Source data Fig. 4 [file 44321_2026_438_MOESM6_ESM.zip › Figure 4/4A/Enhanced TIF Control/1200_5_Enchanced_Green.tif]

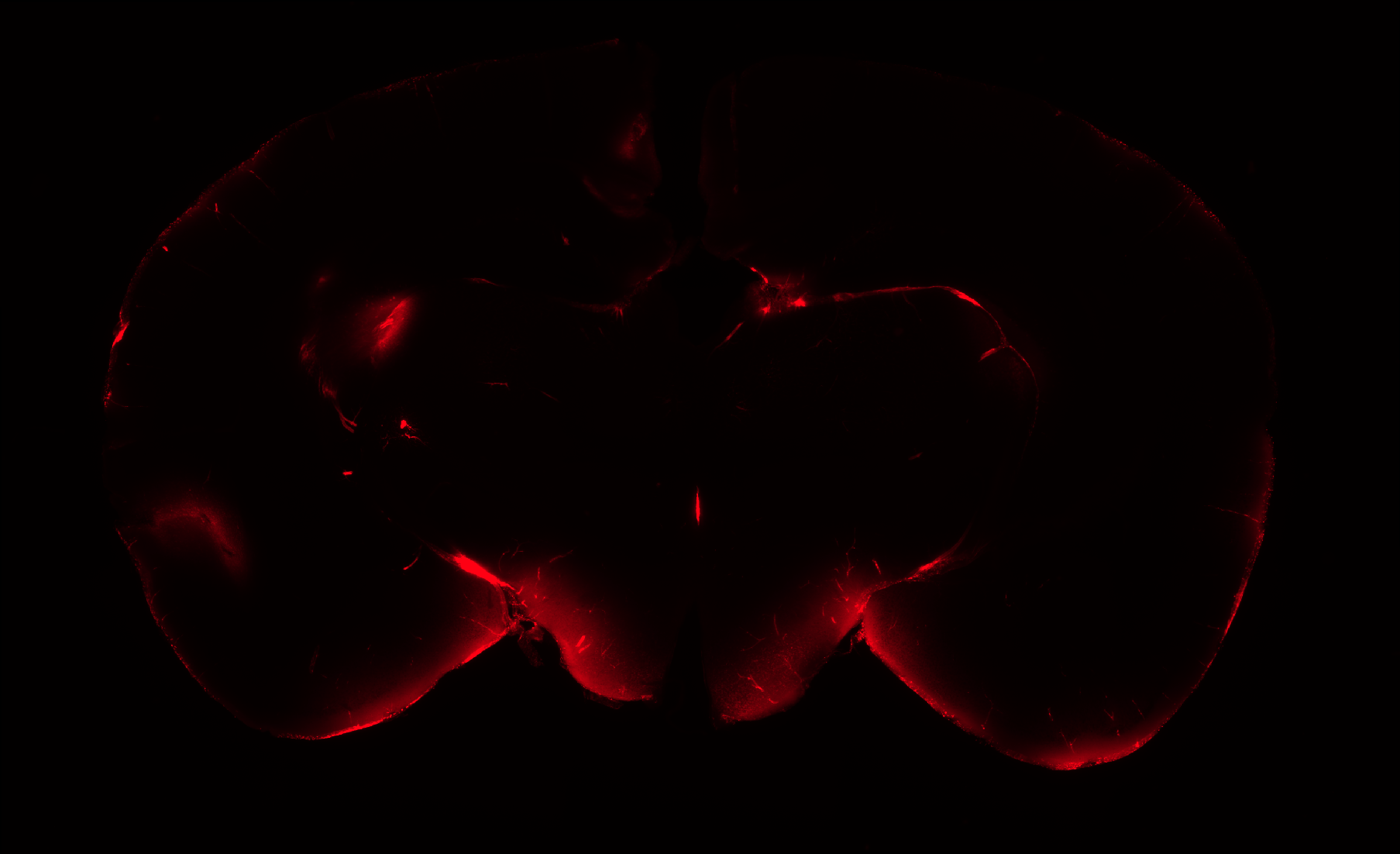

Supplement: Supplementary file 6 — Source data Fig. 4 [file 44321_2026_438_MOESM6_ESM.zip › Figure 4/4A/Enhanced TIF Control/1200_5_Enchanced_Red.tif]

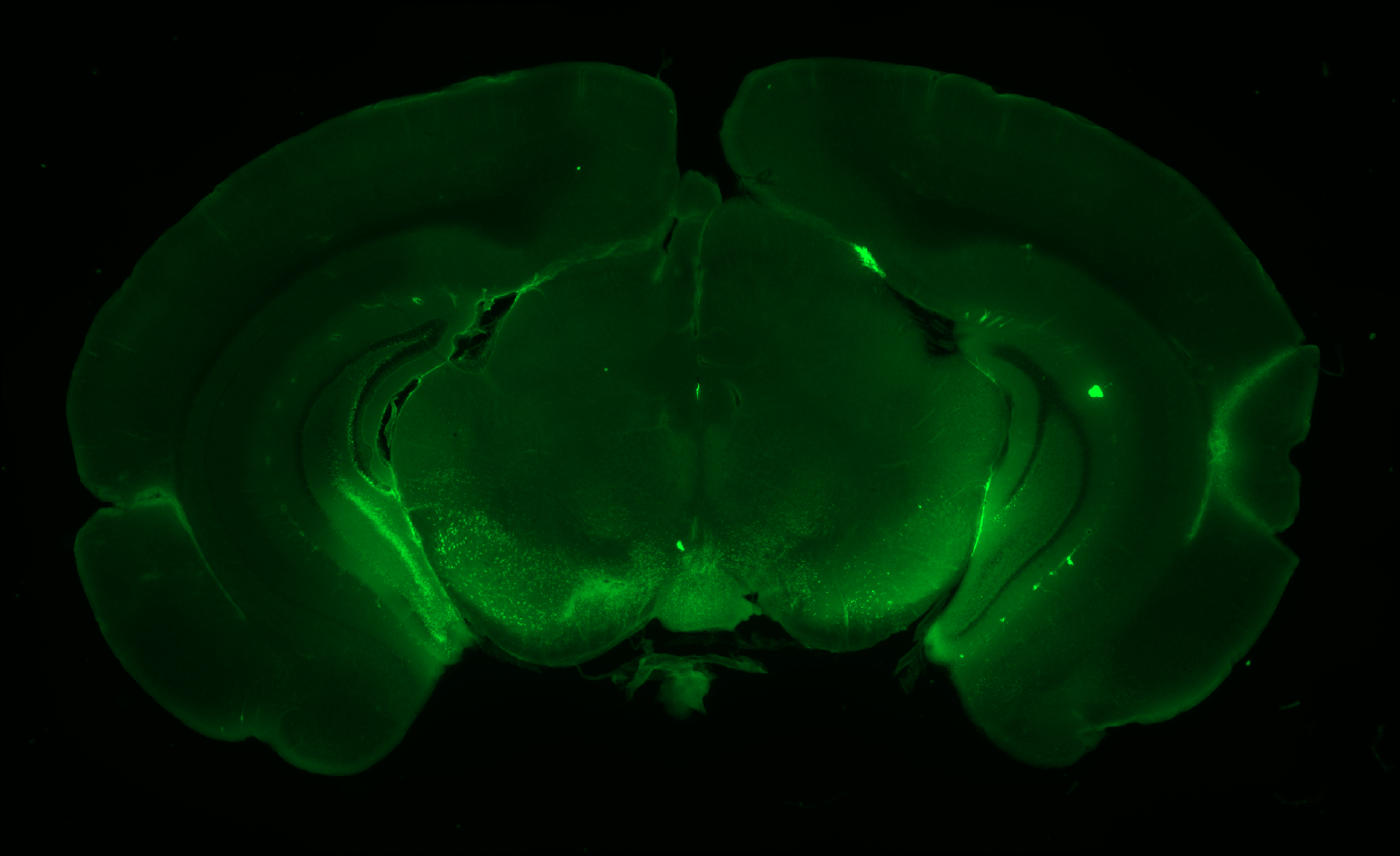

Supplement: Supplementary file 6 — Source data Fig. 4 [file 44321_2026_438_MOESM6_ESM.zip › Figure 4/4A/Enhanced TIF Control/1200_6_Enchanced_Green.tif]

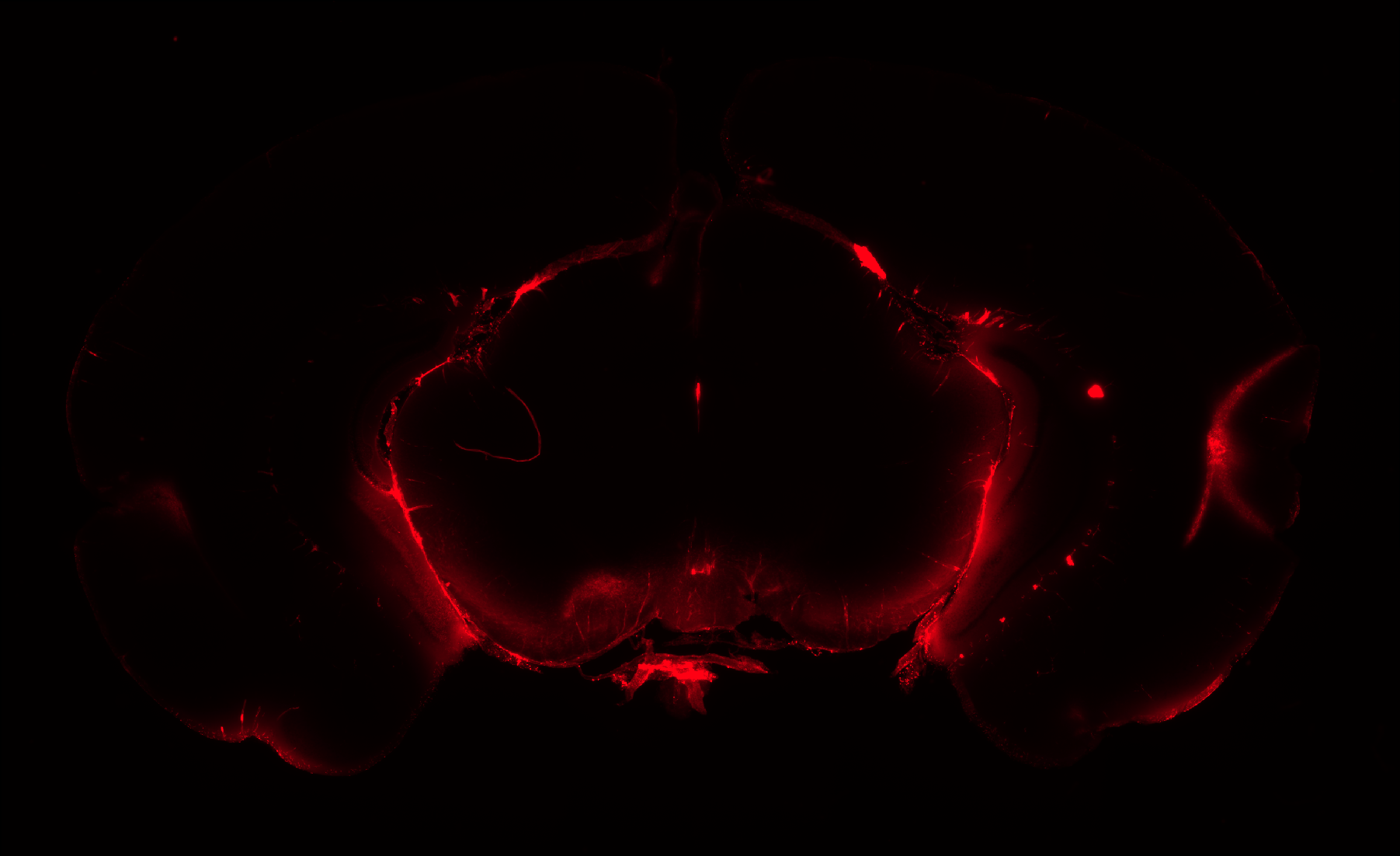

Supplement: Supplementary file 6 — Source data Fig. 4 [file 44321_2026_438_MOESM6_ESM.zip › Figure 4/4A/Enhanced TIF Control/1200_6_Enchanced_Red.tif]

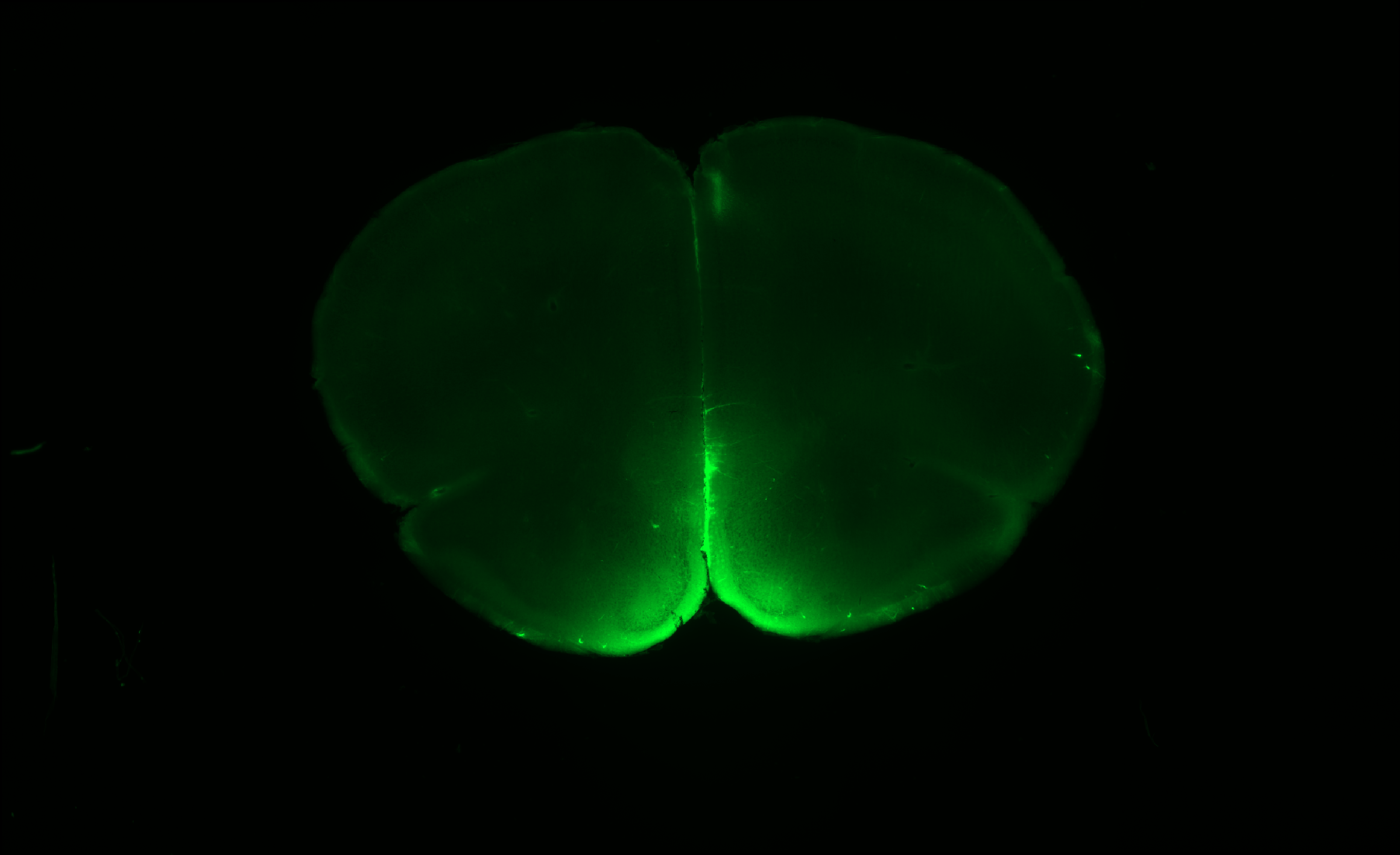

Supplement: Supplementary file 6 — Source data Fig. 4 [file 44321_2026_438_MOESM6_ESM.zip › Figure 4/4A/Enhanced TIF KO/1206_1_Enchanced_Green.tif]

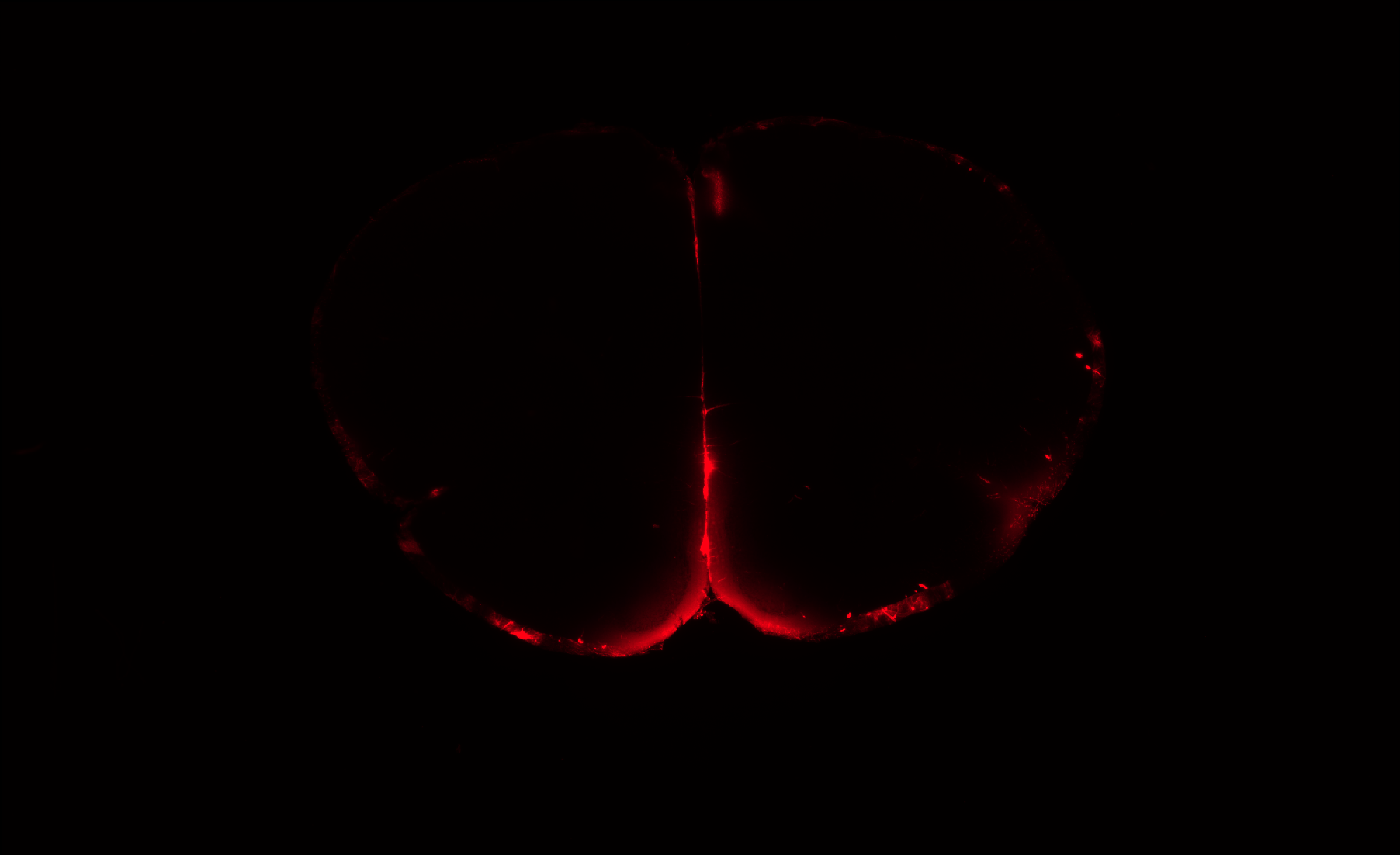

Supplement: Supplementary file 6 — Source data Fig. 4 [file 44321_2026_438_MOESM6_ESM.zip › Figure 4/4A/Enhanced TIF KO/1206_1_Enchanced_Red.tif]

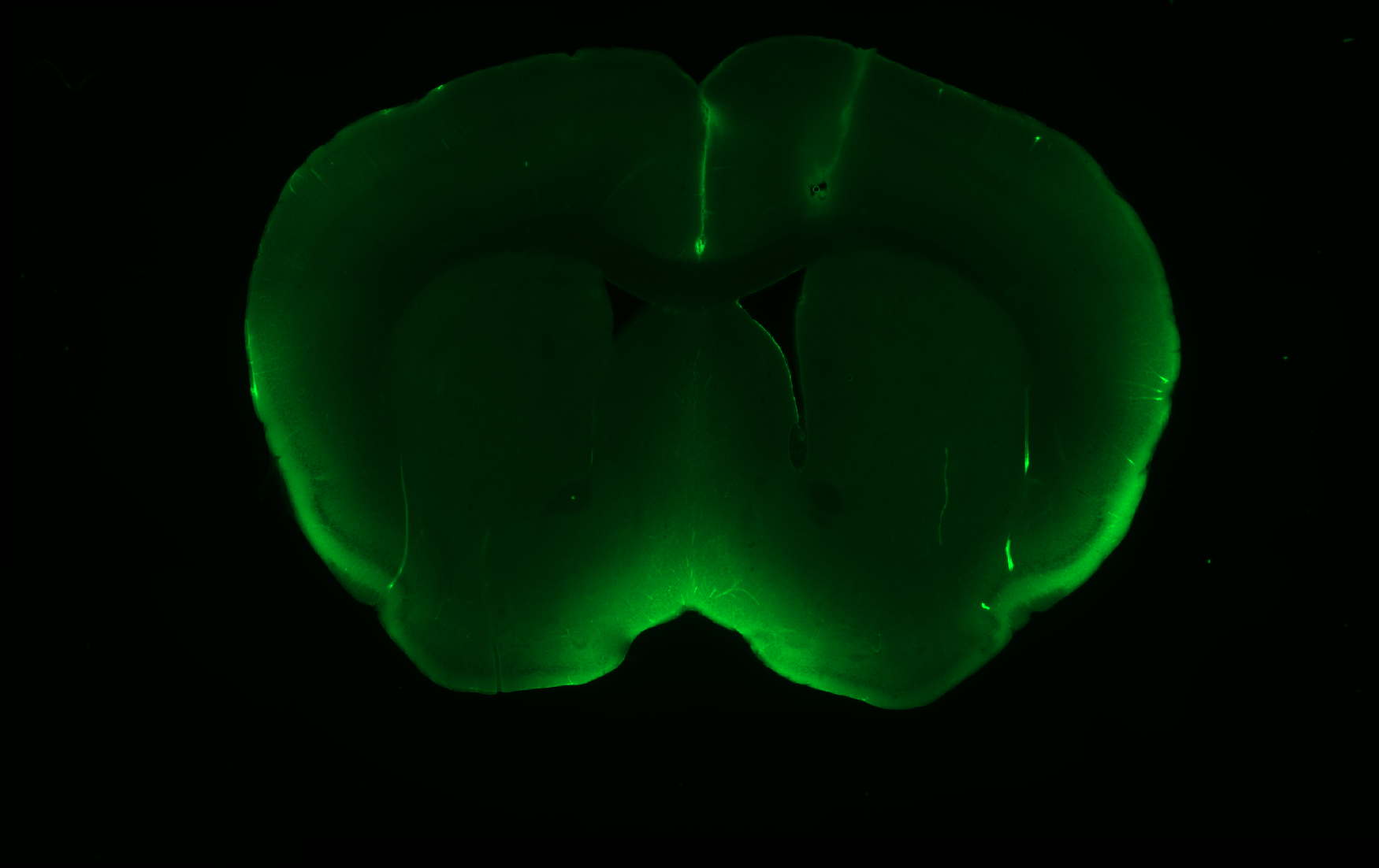

Supplement: Supplementary file 6 — Source data Fig. 4 [file 44321_2026_438_MOESM6_ESM.zip › Figure 4/4A/Enhanced TIF KO/1206_2_Enchanced_Green.tif]

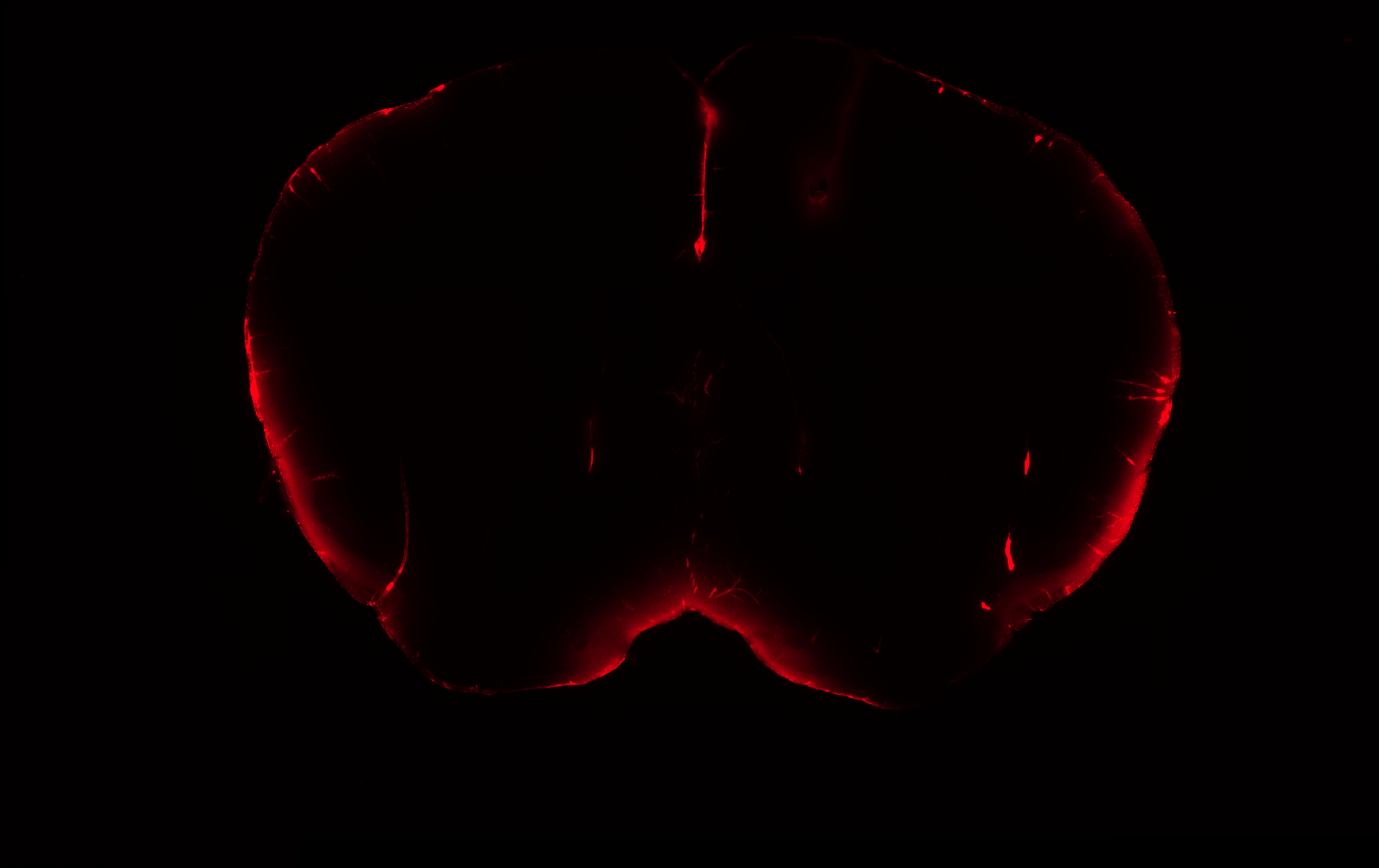

Supplement: Supplementary file 6 — Source data Fig. 4 [file 44321_2026_438_MOESM6_ESM.zip › Figure 4/4A/Enhanced TIF KO/1206_2_Enchanced_Red.tif]

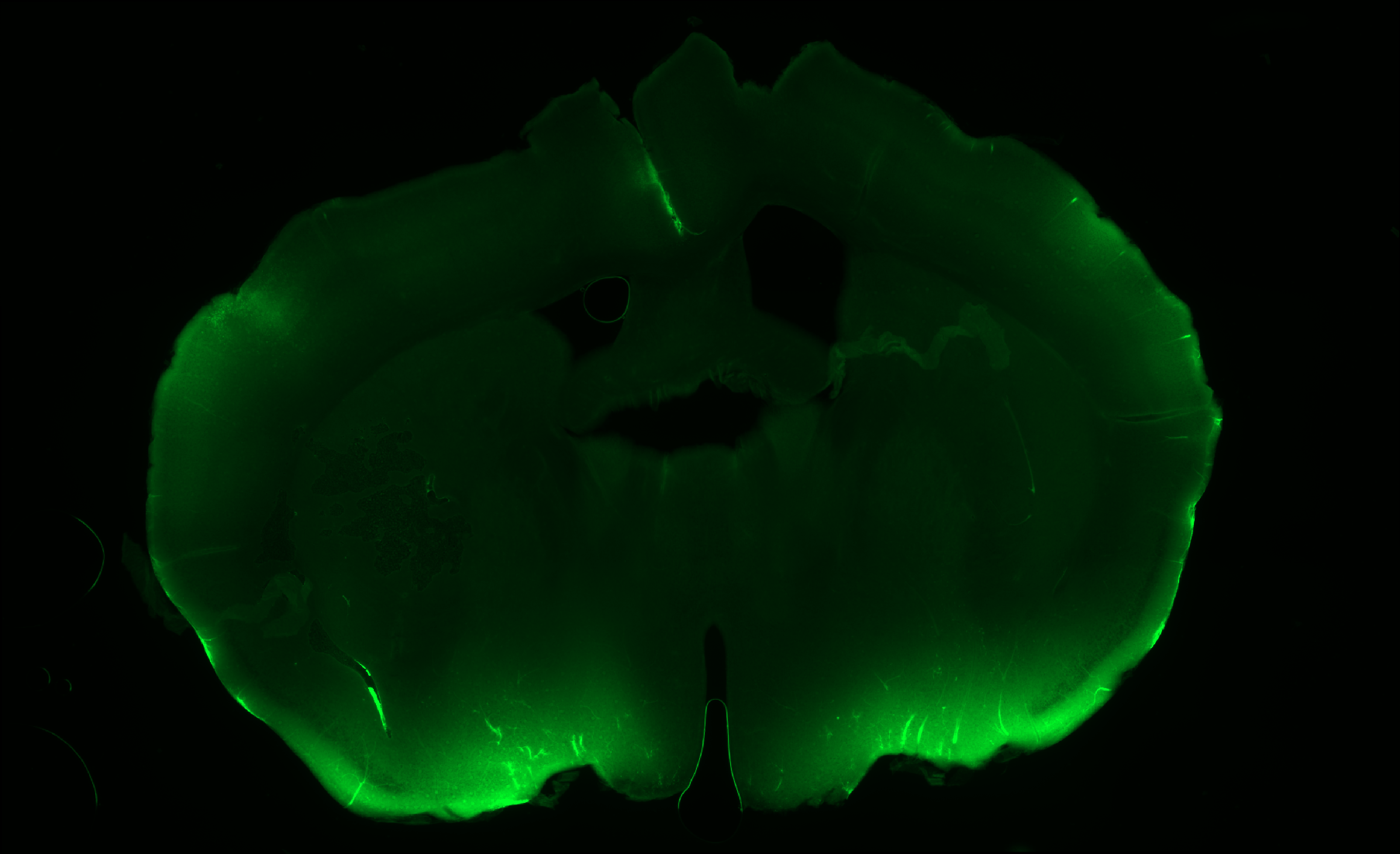

Supplement: Supplementary file 6 — Source data Fig. 4 [file 44321_2026_438_MOESM6_ESM.zip › Figure 4/4A/Enhanced TIF KO/1206_3_Enchanced_Green.tif]

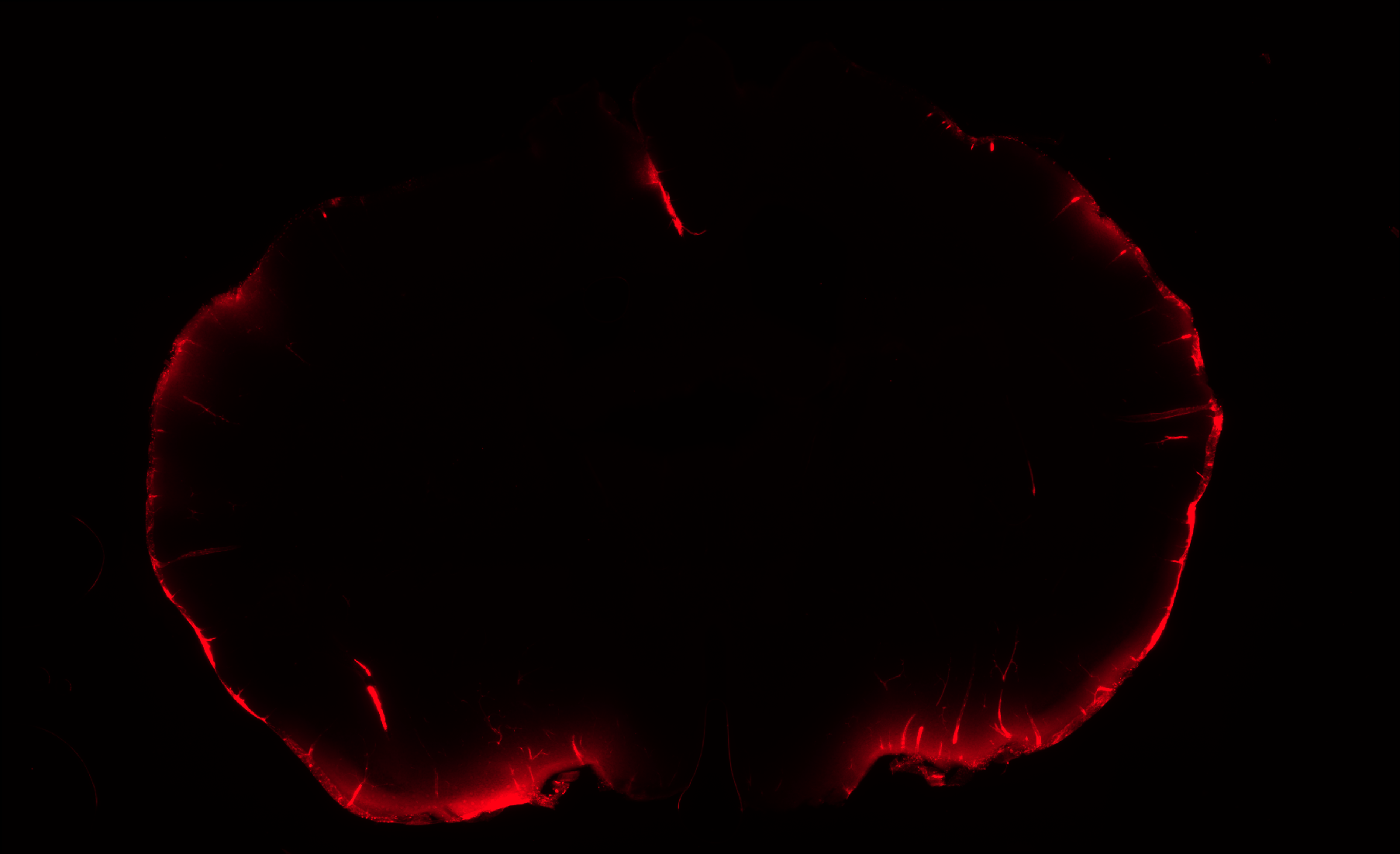

Supplement: Supplementary file 6 — Source data Fig. 4 [file 44321_2026_438_MOESM6_ESM.zip › Figure 4/4A/Enhanced TIF KO/1206_3_Enchanced_Red.tif]

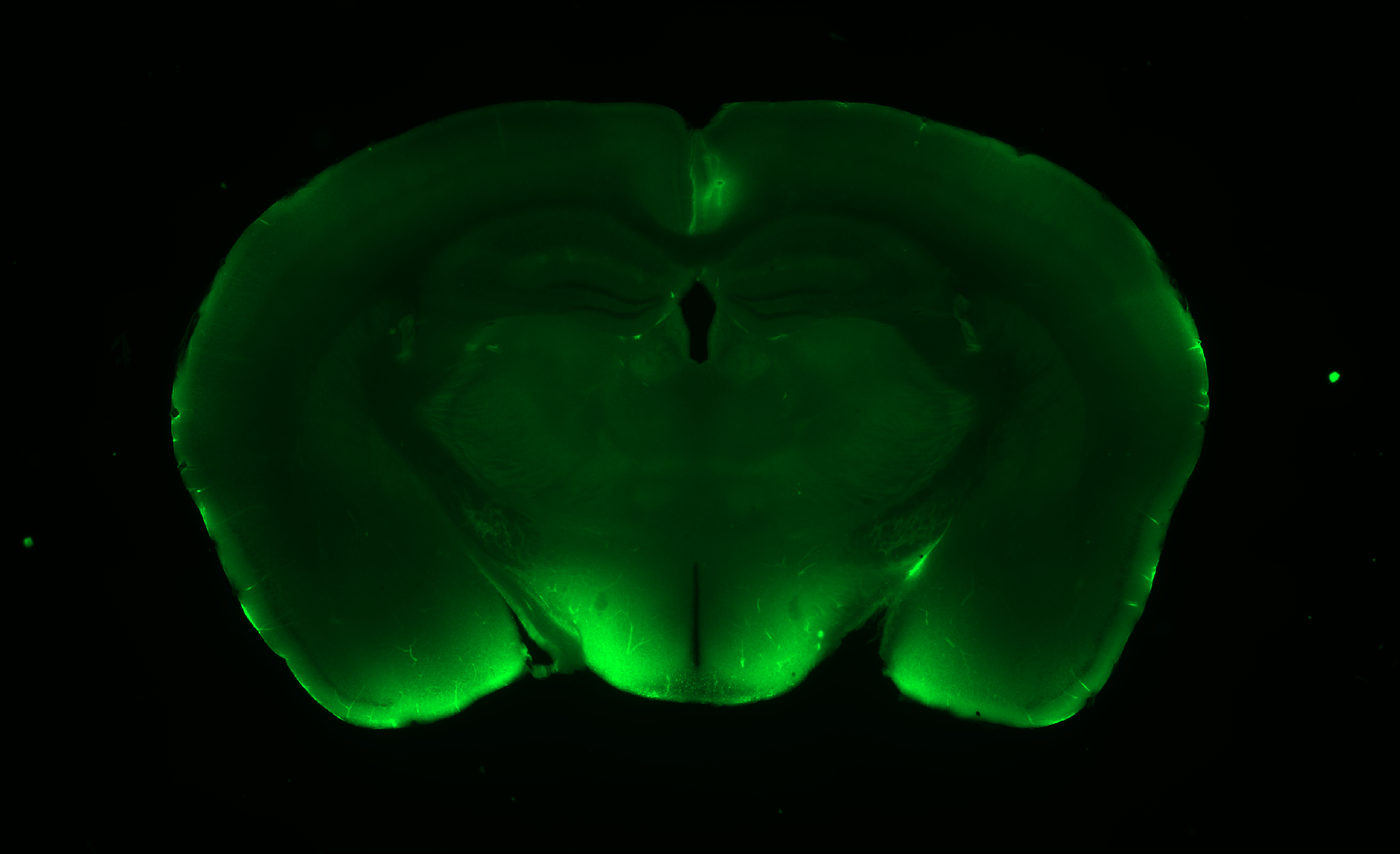

Supplement: Supplementary file 6 — Source data Fig. 4 [file 44321_2026_438_MOESM6_ESM.zip › Figure 4/4A/Enhanced TIF KO/1206_4_Enchanced_Green.tif]

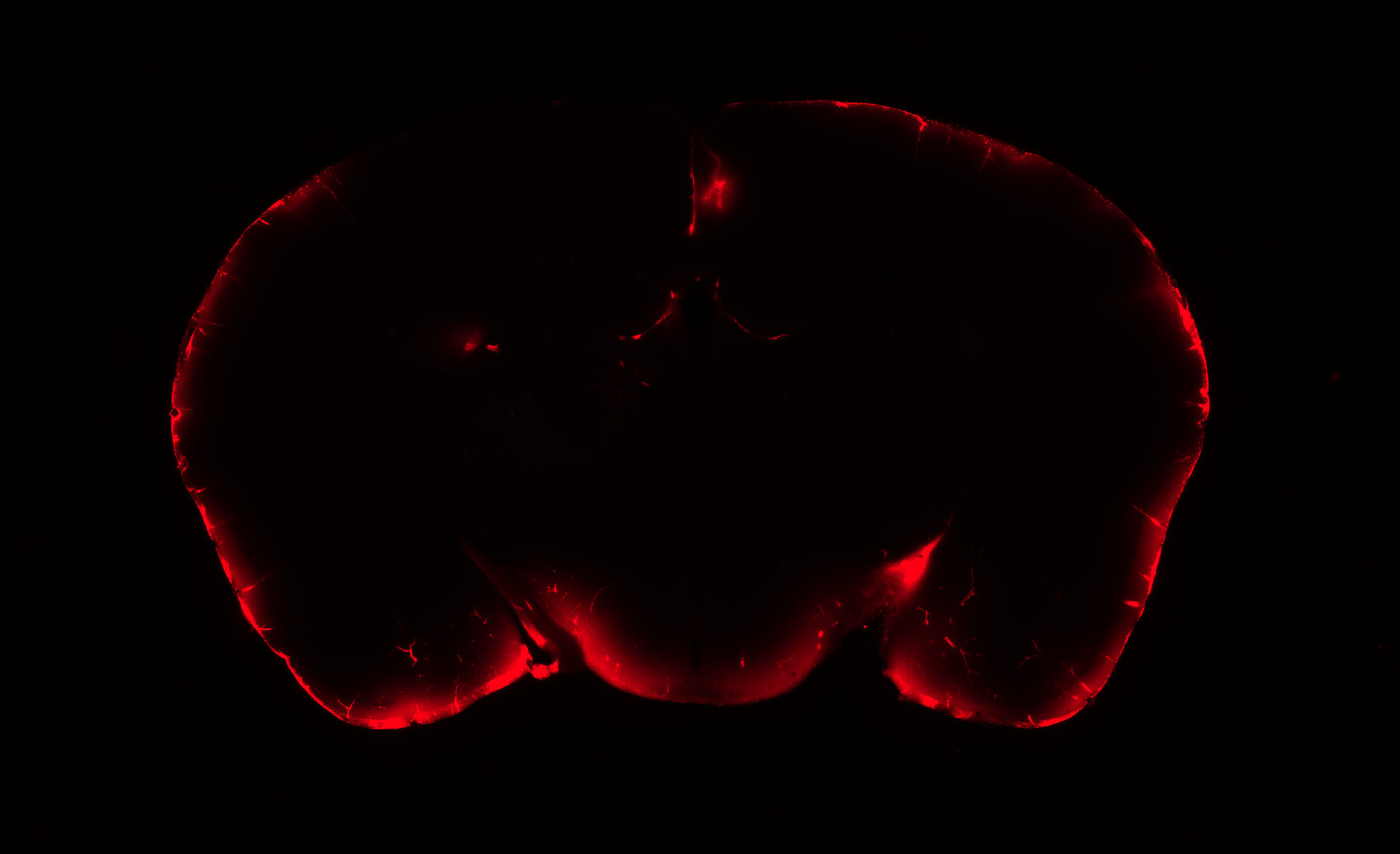

Supplement: Supplementary file 6 — Source data Fig. 4 [file 44321_2026_438_MOESM6_ESM.zip › Figure 4/4A/Enhanced TIF KO/1206_4_Enchanced_Red.tif]

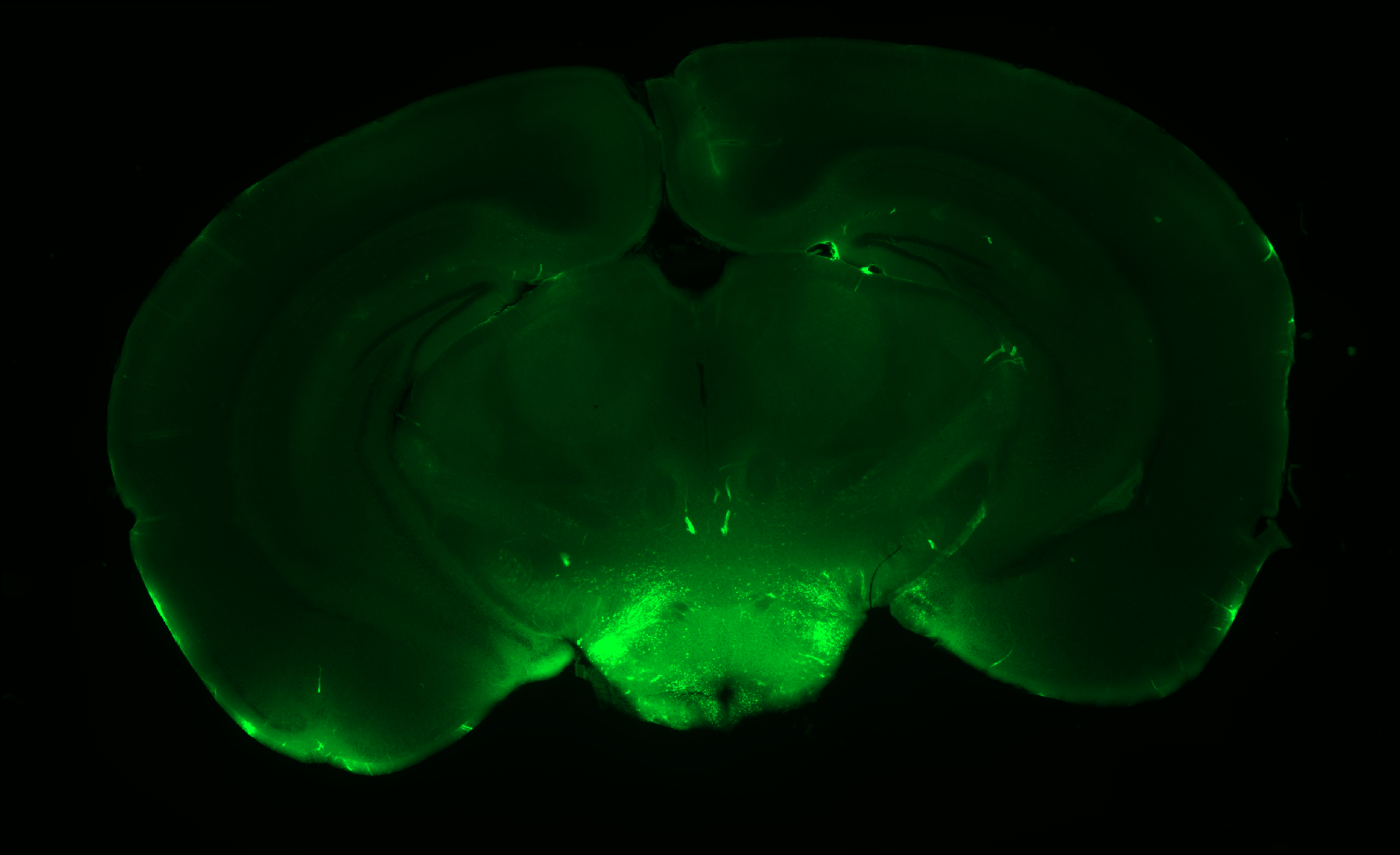

Supplement: Supplementary file 6 — Source data Fig. 4 [file 44321_2026_438_MOESM6_ESM.zip › Figure 4/4A/Enhanced TIF KO/1206_5_Enchanced_Green.tif]

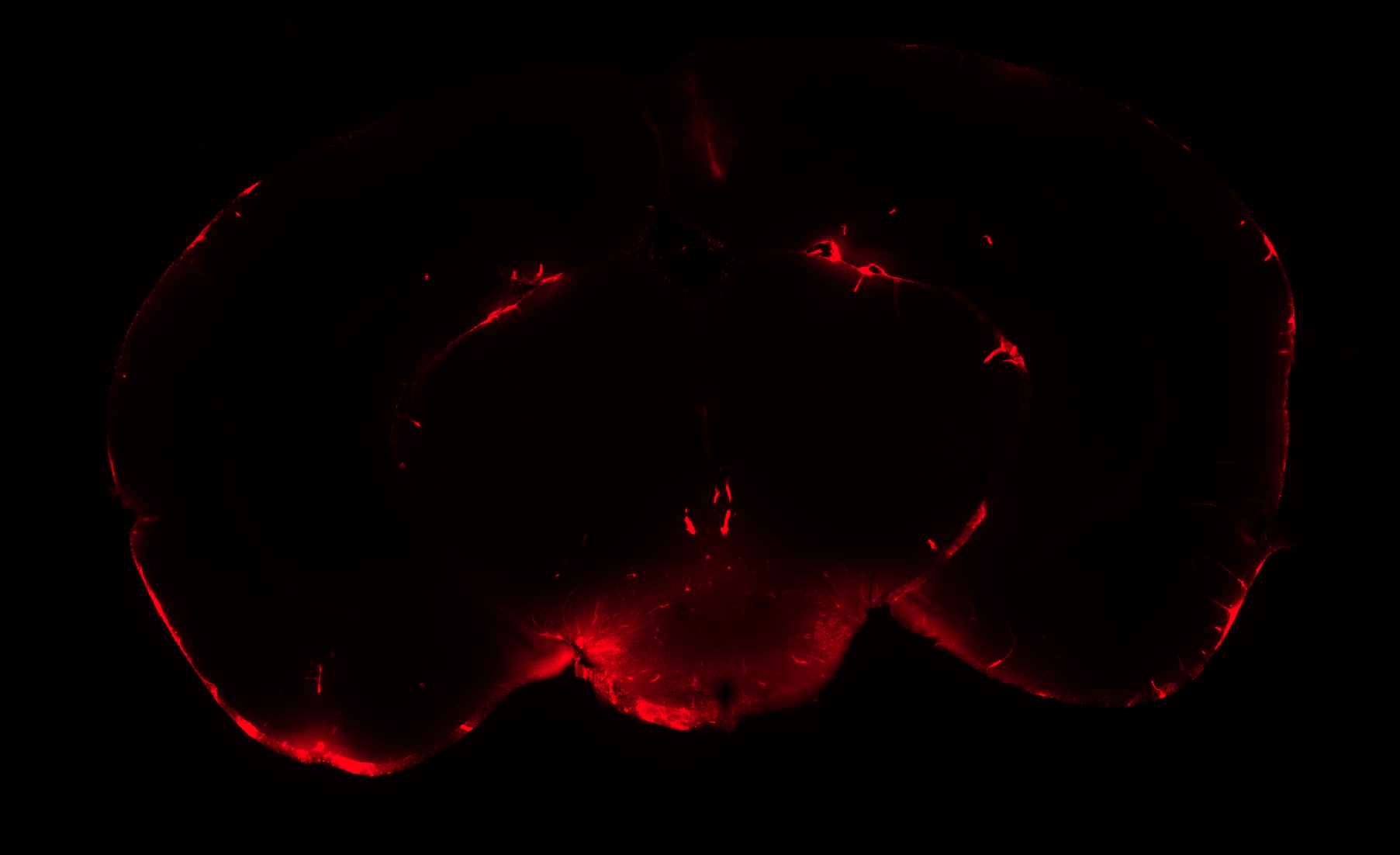

Supplement: Supplementary file 6 — Source data Fig. 4 [file 44321_2026_438_MOESM6_ESM.zip › Figure 4/4A/Enhanced TIF KO/1206_5_Enchanced_Red.tif]

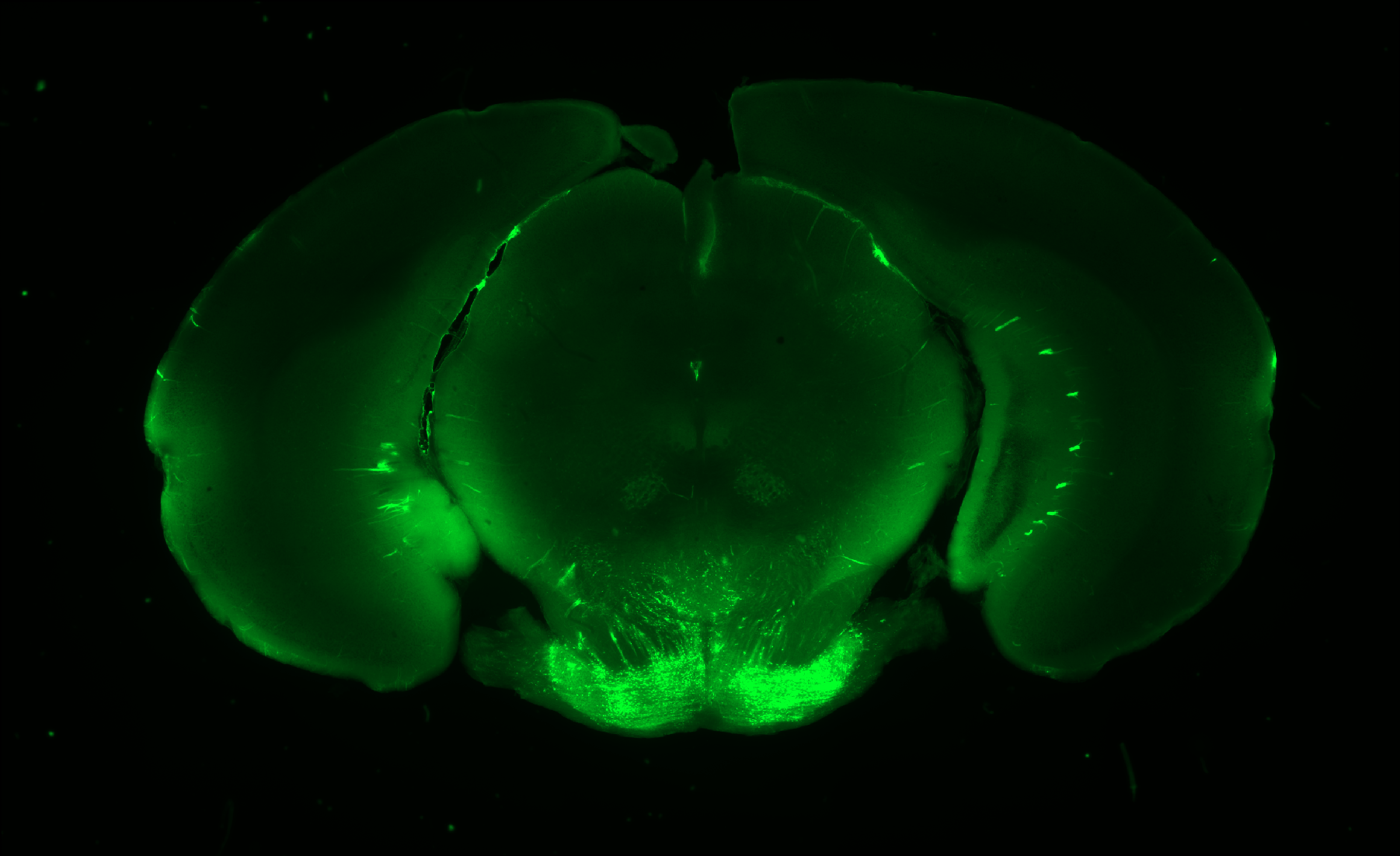

Supplement: Supplementary file 6 — Source data Fig. 4 [file 44321_2026_438_MOESM6_ESM.zip › Figure 4/4A/Enhanced TIF KO/1206_6_Enchanced_Green.tif]

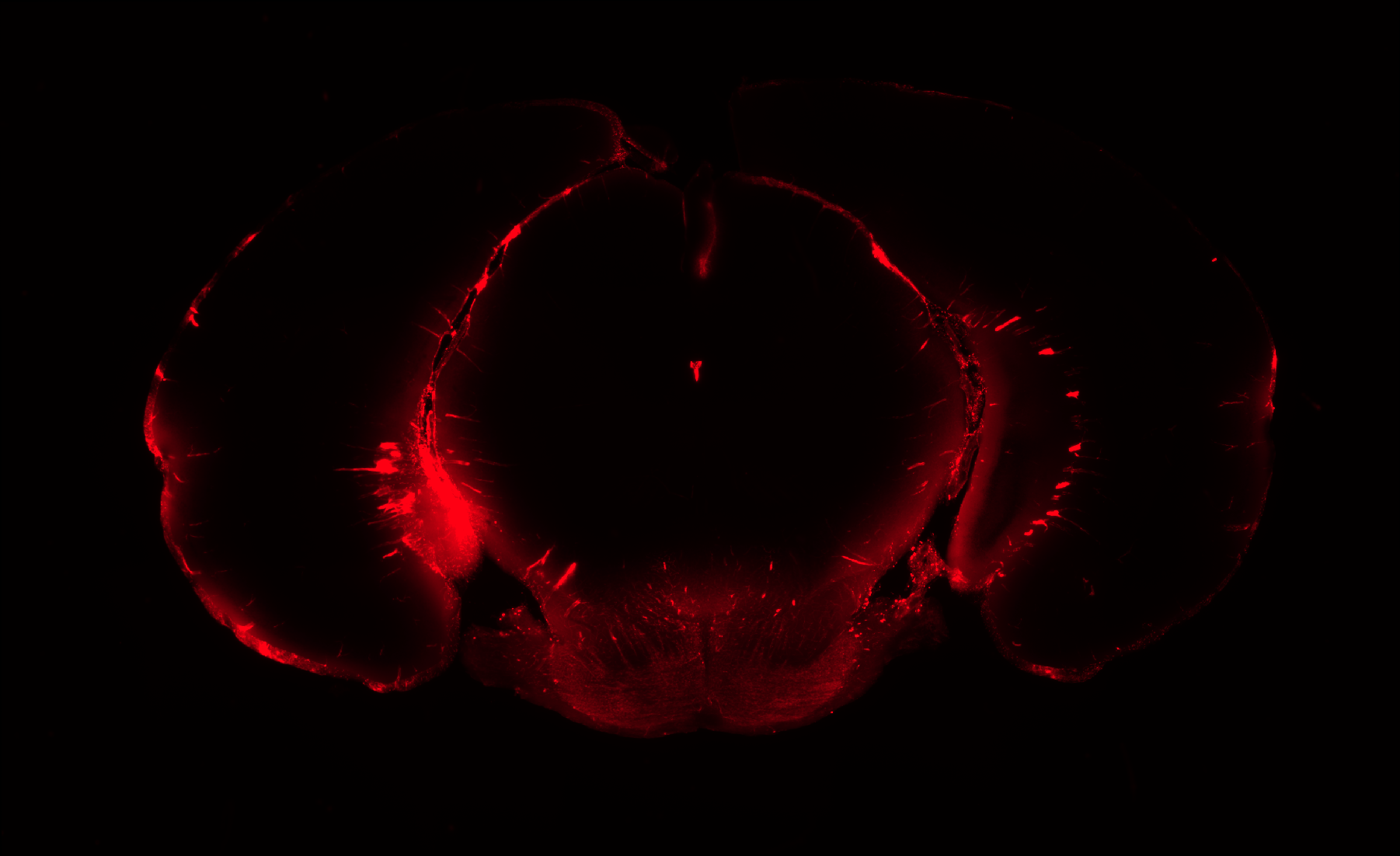

Supplement: Supplementary file 6 — Source data Fig. 4 [file 44321_2026_438_MOESM6_ESM.zip › Figure 4/4A/Enhanced TIF KO/1206_6_Enchanced_Red.tif]

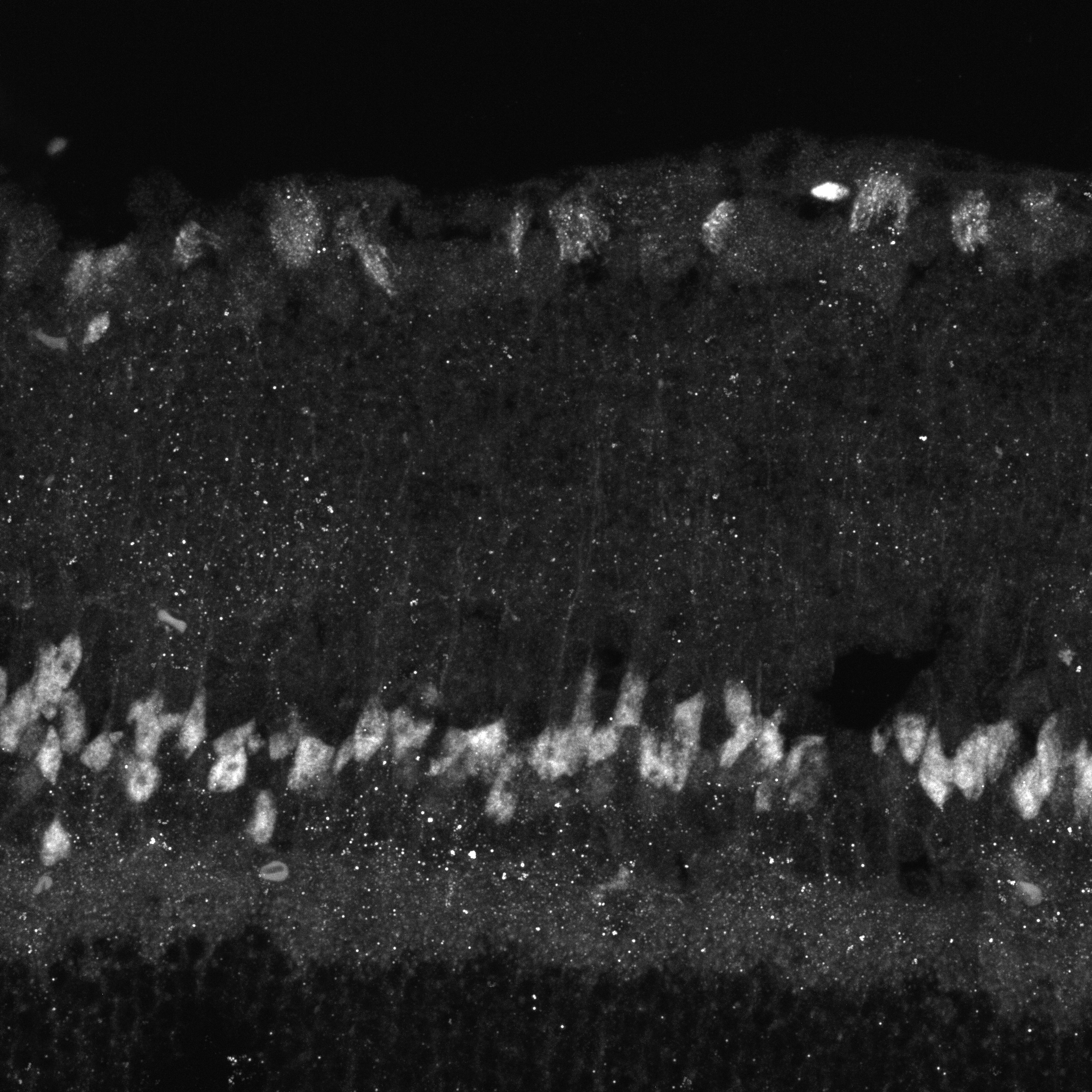

Supplement: Supplementary file 9 — Figure EV1 Source Data [file 44321_2026_438_MOESM9_ESM.zip › Figure EV1/EV1B/FS025_AW7672_Sox9only_8.png]

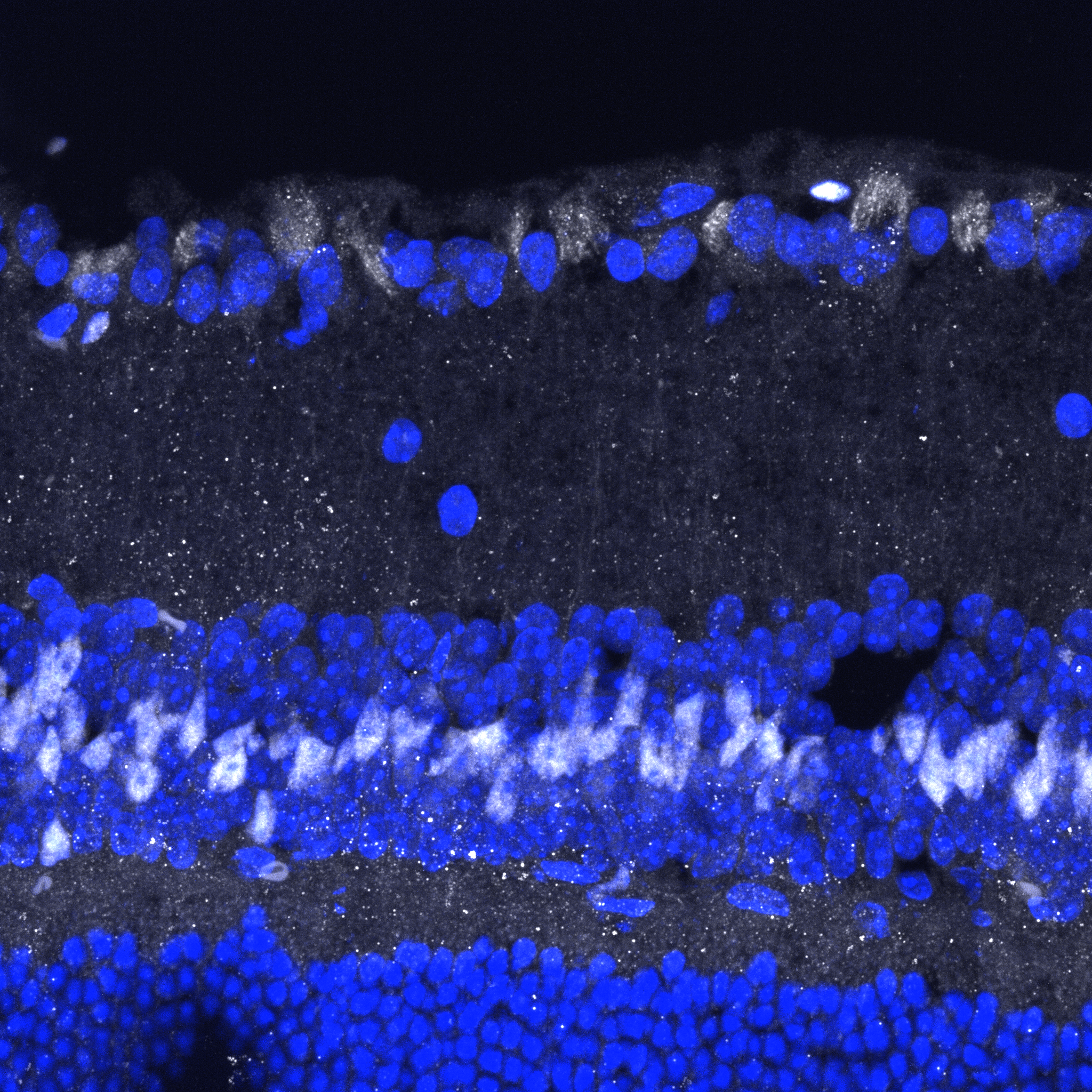

Supplement: Supplementary file 9 — Figure EV1 Source Data [file 44321_2026_438_MOESM9_ESM.zip › Figure EV1/EV1B/FS025_AW7672_Sox9_8-noTomato.png]

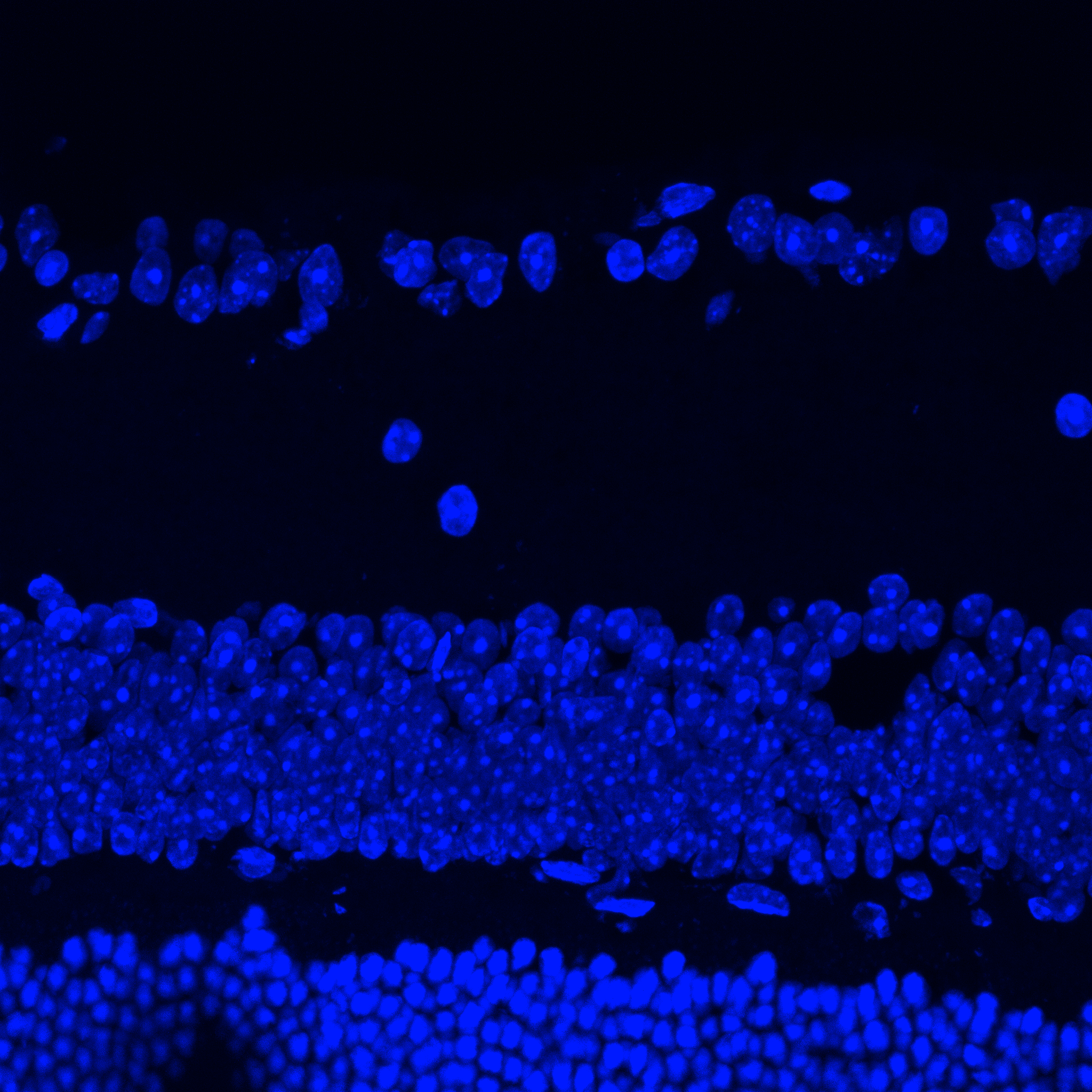

Supplement: Supplementary file 9 — Figure EV1 Source Data [file 44321_2026_438_MOESM9_ESM.zip › Figure EV1/EV1B/FS025_AW7672_Sox9_8.png]

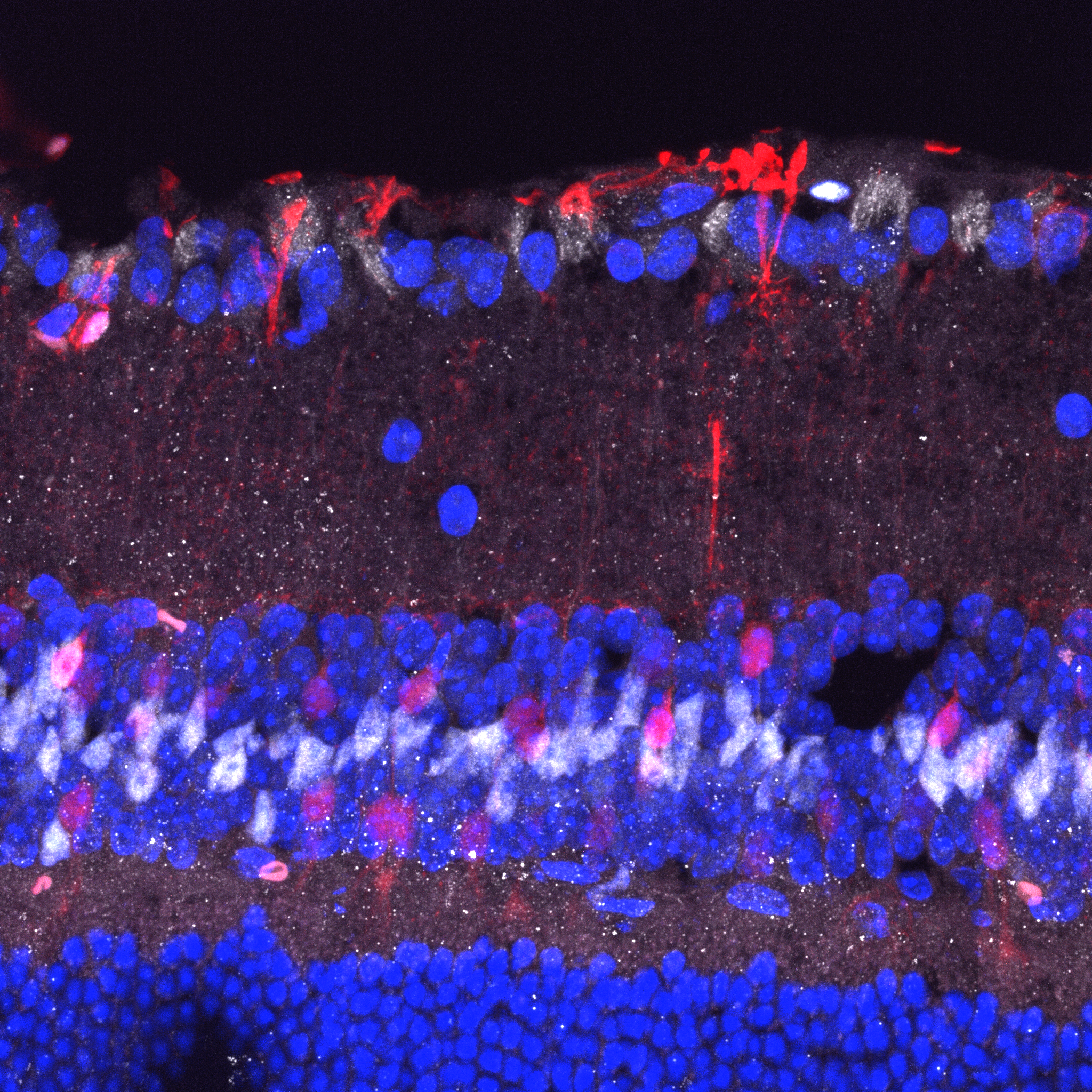

Supplement: Supplementary file 9 — Figure EV1 Source Data [file 44321_2026_438_MOESM9_ESM.zip › Figure EV1/EV1B/FS025_AW7672_Sox9_8_.png]

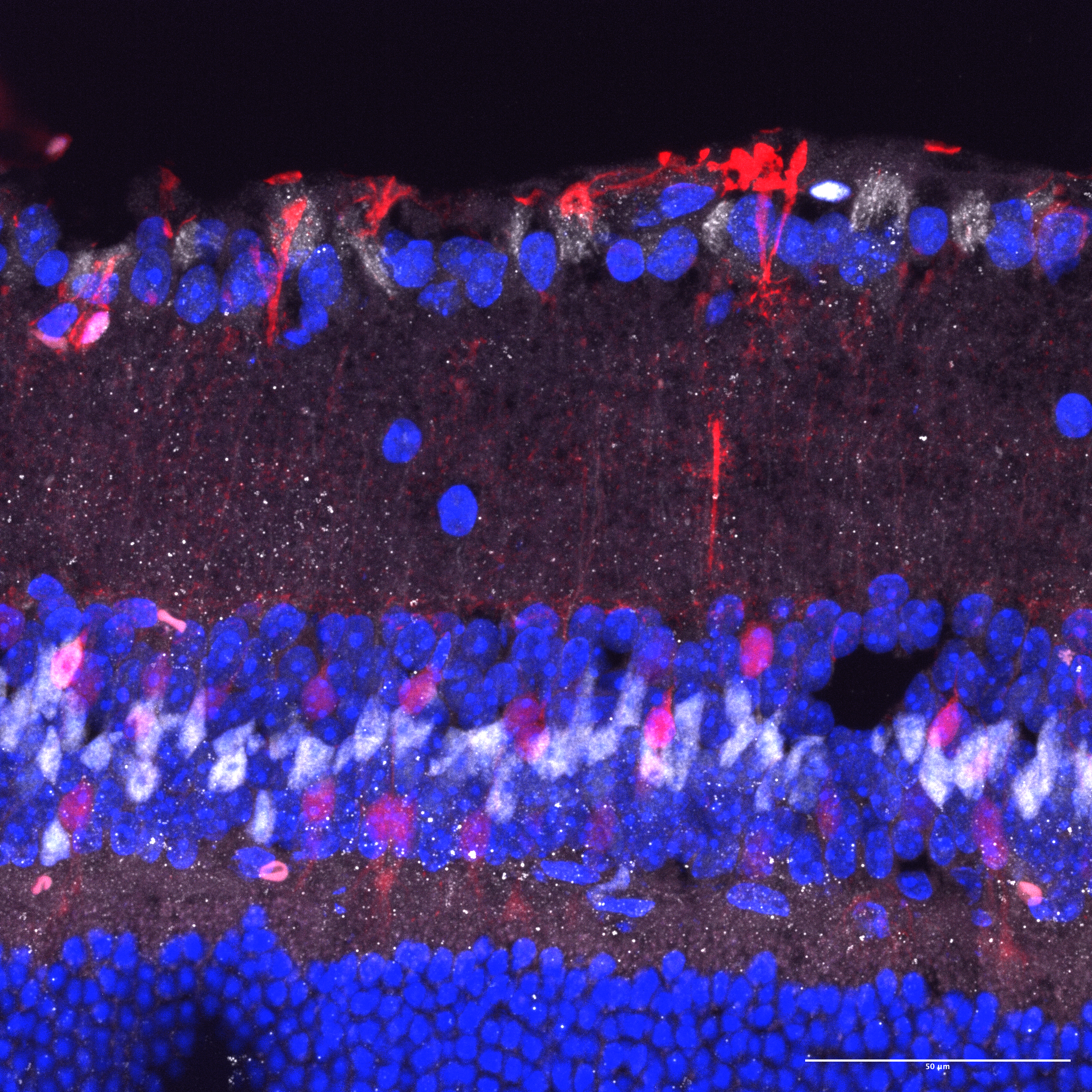

Supplement: Supplementary file 9 — Figure EV1 Source Data [file 44321_2026_438_MOESM9_ESM.zip › Figure EV1/EV1B/FS025_AW7672_Sox9_8_scale.png]

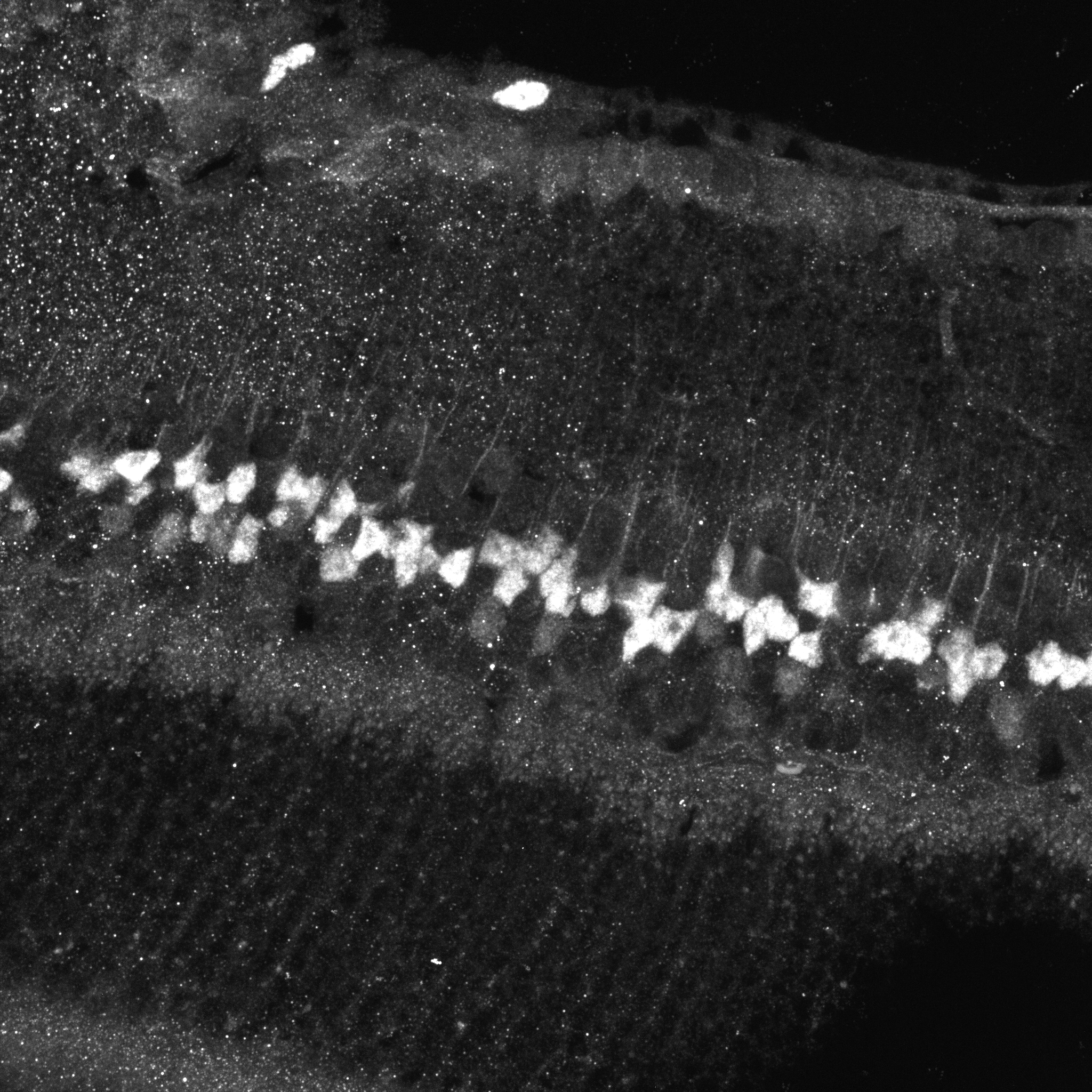

Supplement: Supplementary file 9 — Figure EV1 Source Data [file 44321_2026_438_MOESM9_ESM.zip › Figure EV1/EV1B/FS025_AW7675_Sox9only_4.png]

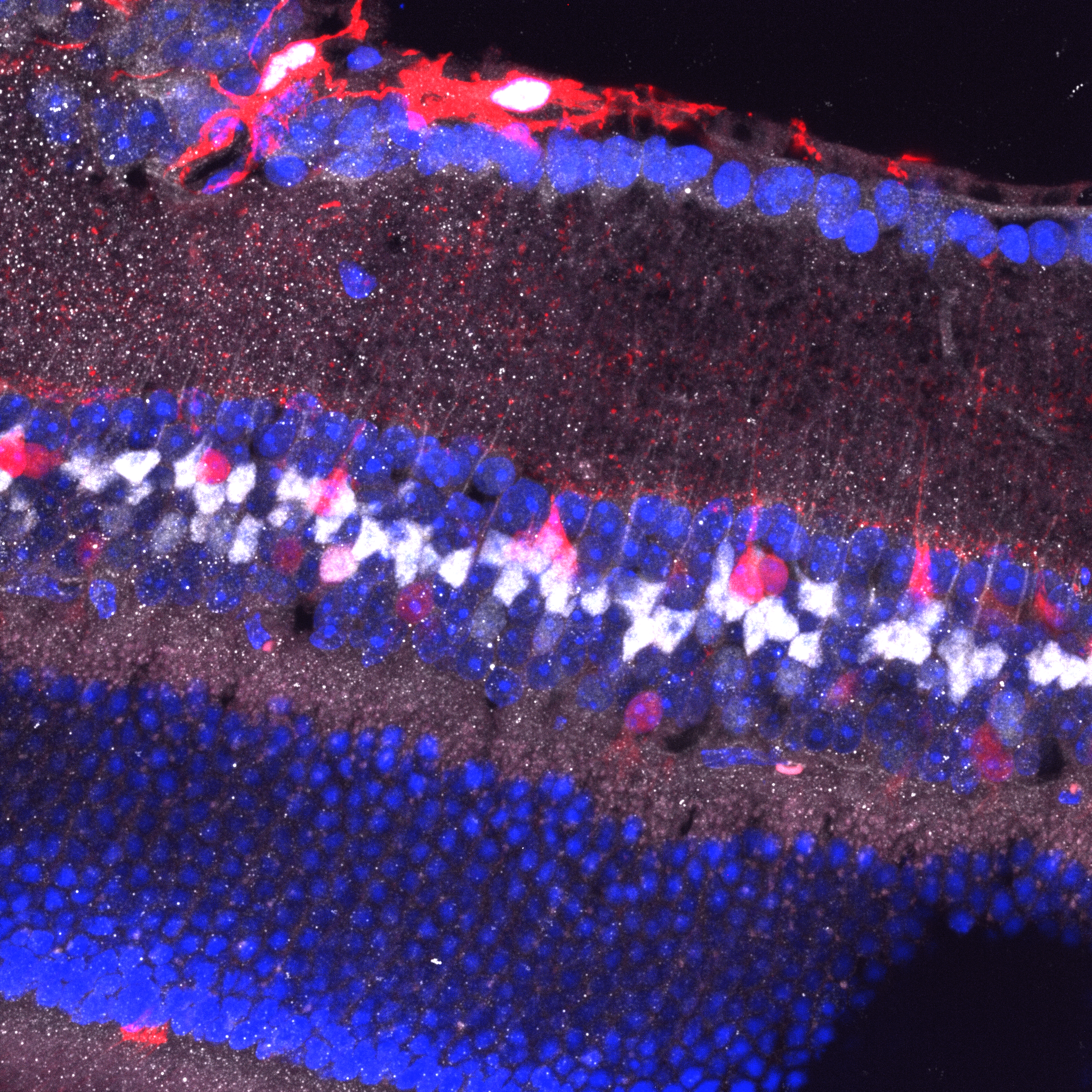

Supplement: Supplementary file 9 — Figure EV1 Source Data [file 44321_2026_438_MOESM9_ESM.zip › Figure EV1/EV1B/FS025_AW7675_Sox9_4.png]

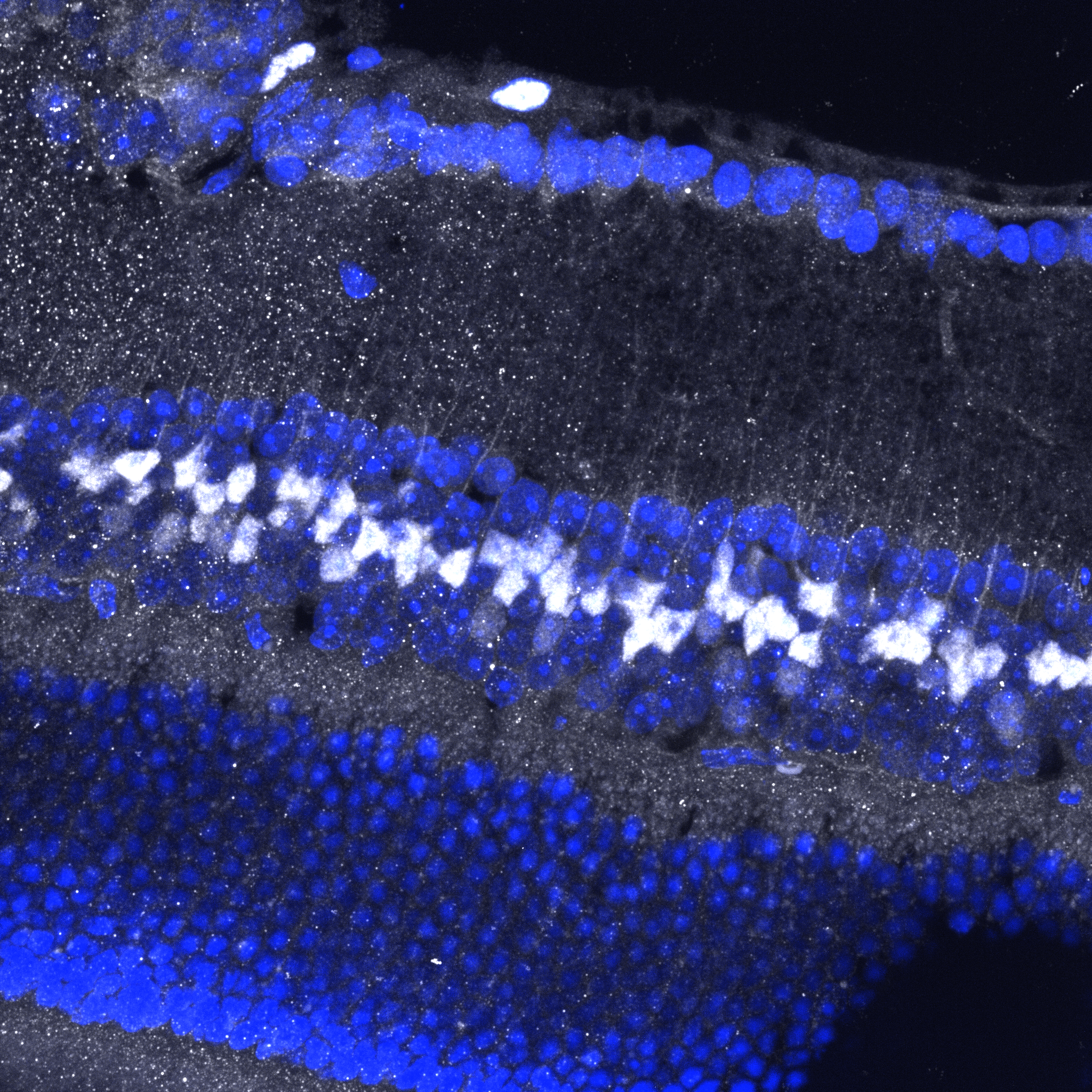

Supplement: Supplementary file 9 — Figure EV1 Source Data [file 44321_2026_438_MOESM9_ESM.zip › Figure EV1/EV1B/FS025_AW7675_Sox9_4_noTomato.png]

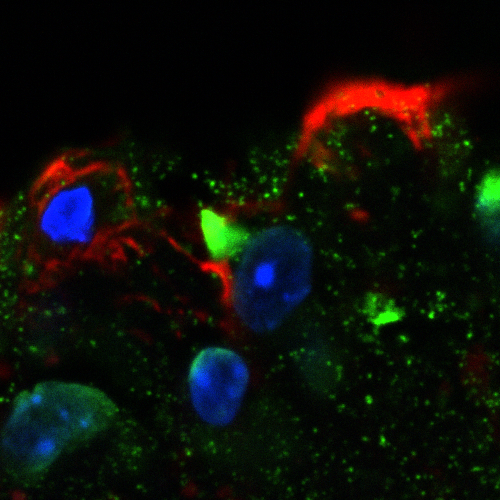

Supplement: Supplementary file 10 — Figure EV2 Source Data [file 44321_2026_438_MOESM10_ESM.zip › Figure EV2/EV2A/AW7672_crop.png]

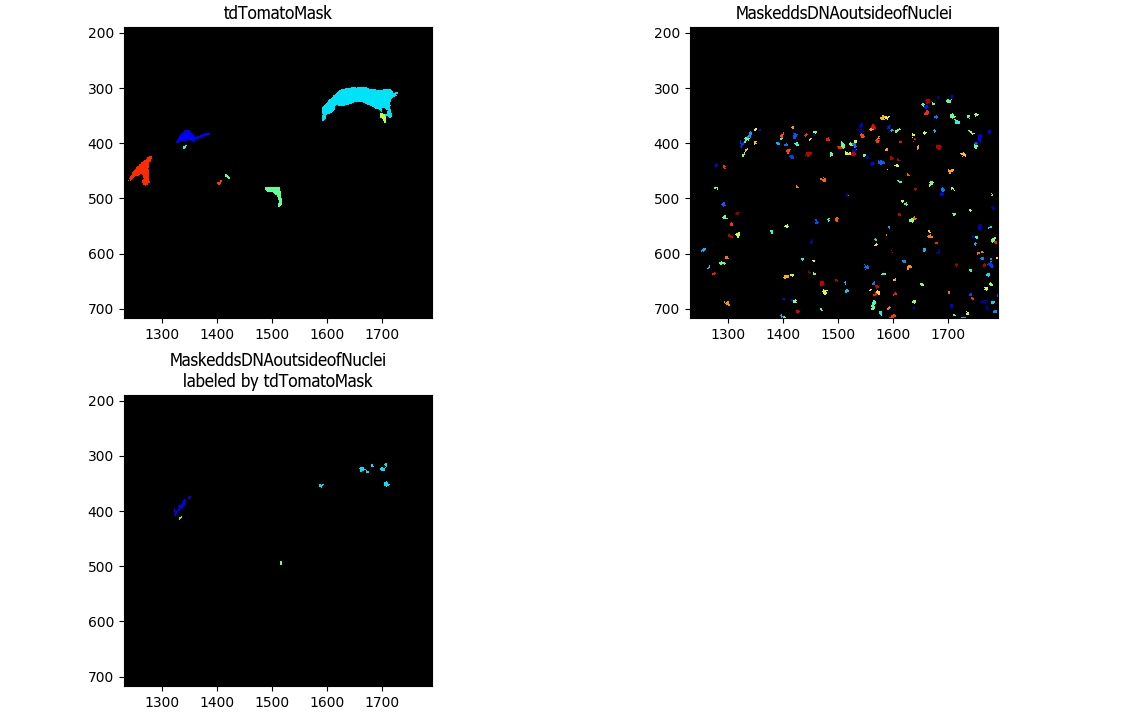

Supplement: Supplementary file 10 — Figure EV2 Source Data [file 44321_2026_438_MOESM10_ESM.zip › Figure EV2/EV2A/AW7672_crop_CP.png]

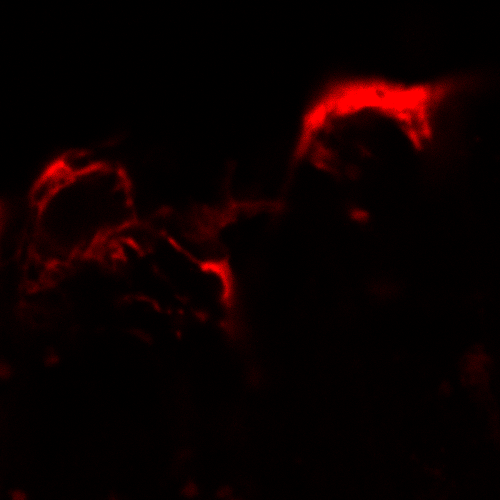

Supplement: Supplementary file 10 — Figure EV2 Source Data [file 44321_2026_438_MOESM10_ESM.zip › Figure EV2/EV2A/C1-AW7672_crop.png]

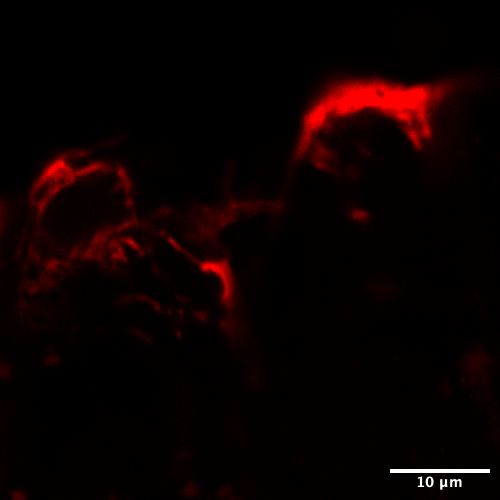

Supplement: Supplementary file 10 — Figure EV2 Source Data [file 44321_2026_438_MOESM10_ESM.zip › Figure EV2/EV2A/C1-AW7672_crop_scale.png]
