# Supplementary material for: Loss of mitochondrial DNA helicase in retinal macroglia drives neovascular retinopathy
Source: EMBO Mol Med. 2026 May 8;18(7):2573–98. doi: 10.1038/s44321-026-00438-0 (PMC13365537; doi:10.1038/s44321-026-00438-0)
Supplement: Supplementary file 4 — Source data Fig. 2 [file 44321_2026_438_MOESM4_ESM.zip › Figure 2/2C/Tracer_injection_flow.pdf]

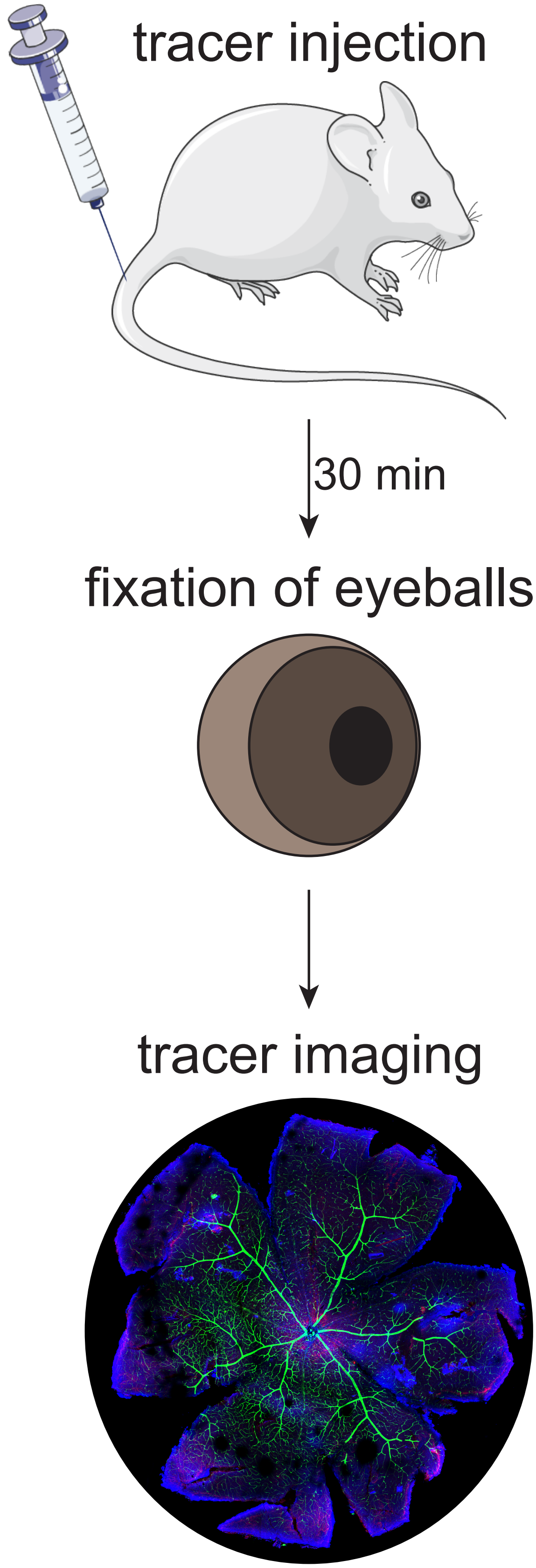
tracer injection
30 min
fixation of eyeballs
tracer imaging
