# Supplementary figures and images for: Loss of mitochondrial DNA helicase in retinal macroglia drives neovascular retinopathy (part 2 of 2)
Source: EMBO Mol Med. 2026 May 8;18(7):2573–98. doi: 10.1038/s44321-026-00438-0 (PMC13365537; doi:10.1038/s44321-026-00438-0)

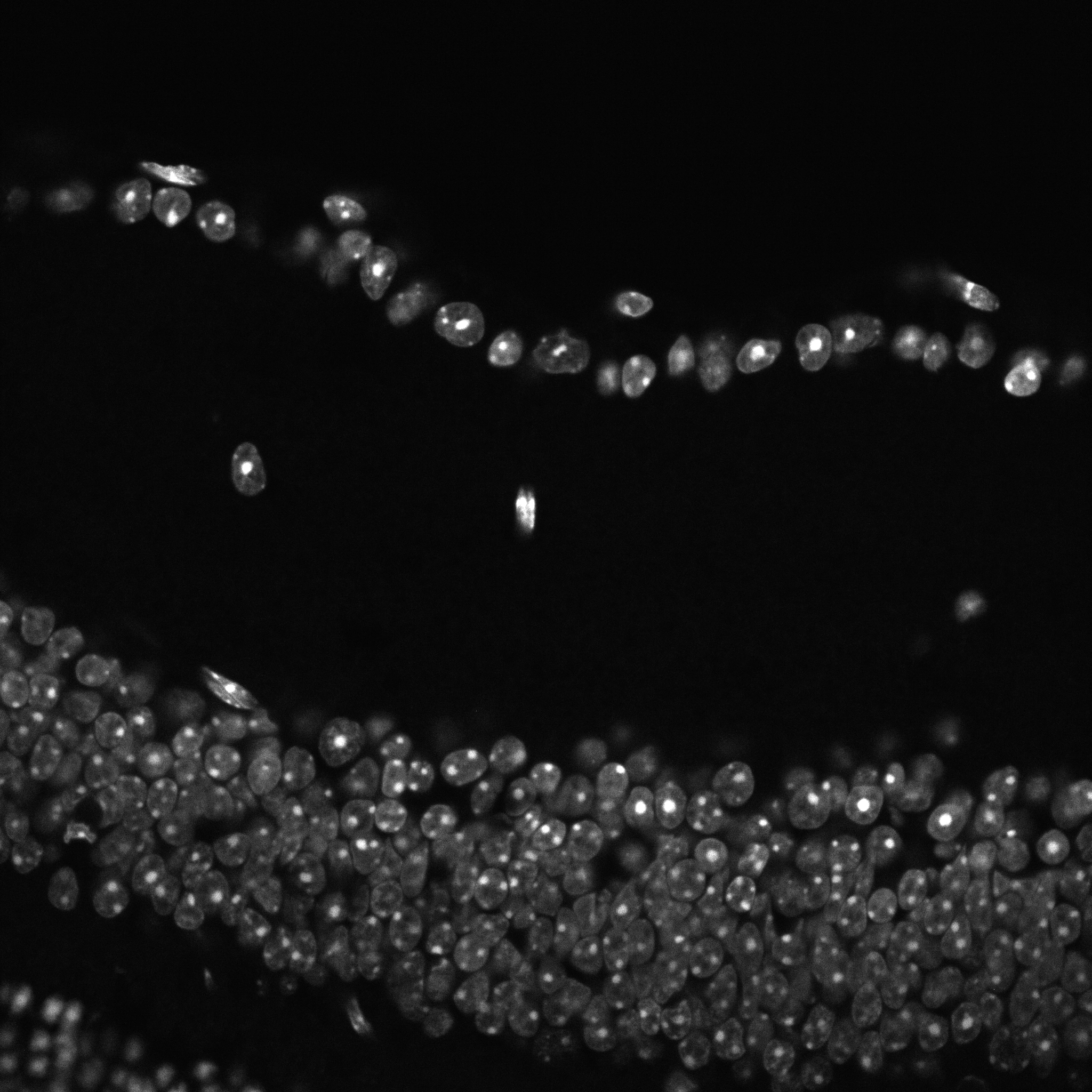

Supplement: Supplementary file 10 — Figure EV2 Source Data [file 44321_2026_438_MOESM10_ESM.zip › Figure EV2/EV2A/C1_AW7675_5u_405_DAPI_CF40_Zyla_488_GFP_CF40_Zyla_561_RFP_C..._RFP_CF40_Zyla_Retina60x_mtDNA_tomato_2.ims Resolution Level 1 Z=15 C=0.tif]

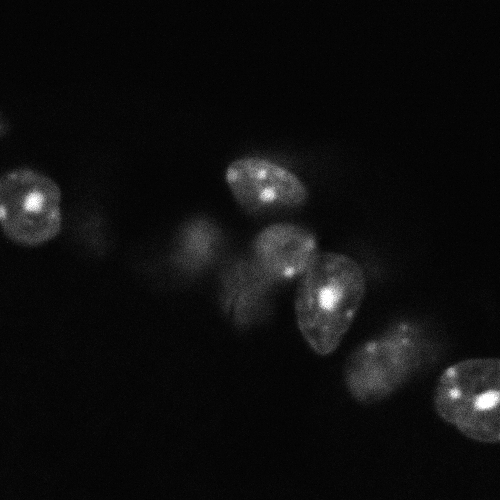

Supplement: Supplementary file 10 — Figure EV2 Source Data [file 44321_2026_438_MOESM10_ESM.zip › Figure EV2/EV2A/C1_AW7675_crop C=0.tif]

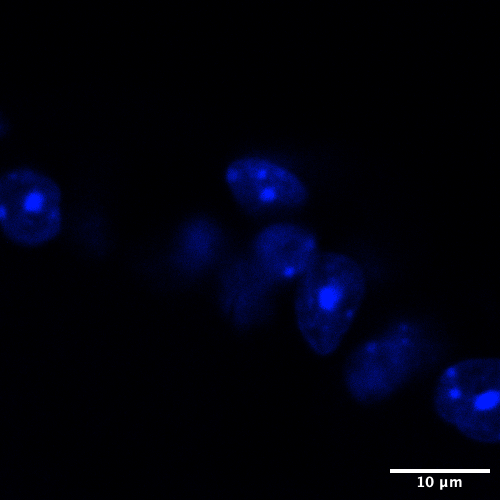

Supplement: Supplementary file 10 — Figure EV2 Source Data [file 44321_2026_438_MOESM10_ESM.zip › Figure EV2/EV2A/C1_AW7675_crop.png]

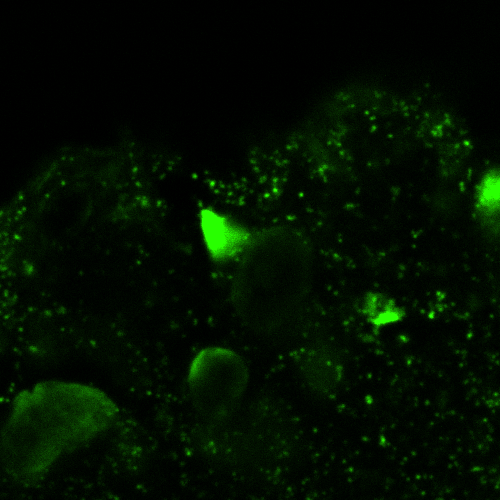

Supplement: Supplementary file 10 — Figure EV2 Source Data [file 44321_2026_438_MOESM10_ESM.zip › Figure EV2/EV2A/C2-AW7672_crop.png]

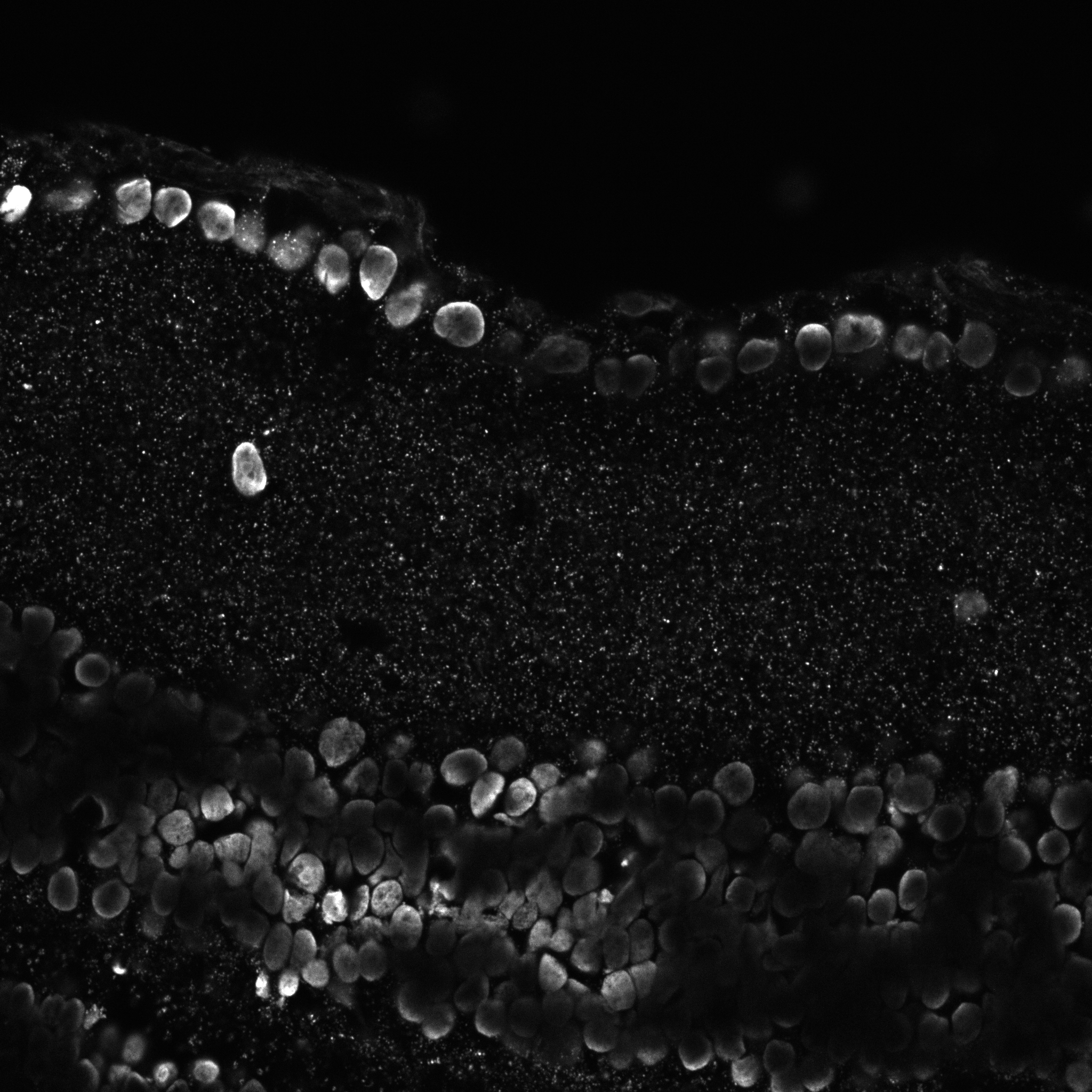

Supplement: Supplementary file 10 — Figure EV2 Source Data [file 44321_2026_438_MOESM10_ESM.zip › Figure EV2/EV2A/C2_AW7675_5u_405_DAPI_CF40_Zyla_488_GFP_CF40_Zyla_561_RFP_C..._RFP_CF40_Zyla_Retina60x_mtDNA_tomato_2.ims Resolution Level 1 Z=15 C=1.tif]

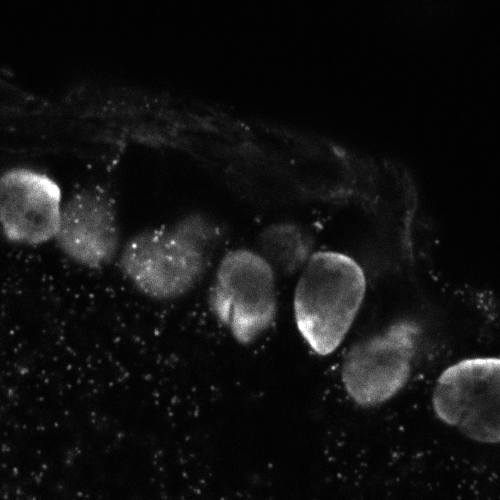

Supplement: Supplementary file 10 — Figure EV2 Source Data [file 44321_2026_438_MOESM10_ESM.zip › Figure EV2/EV2A/C2_AW7675_crop C=1.tif]

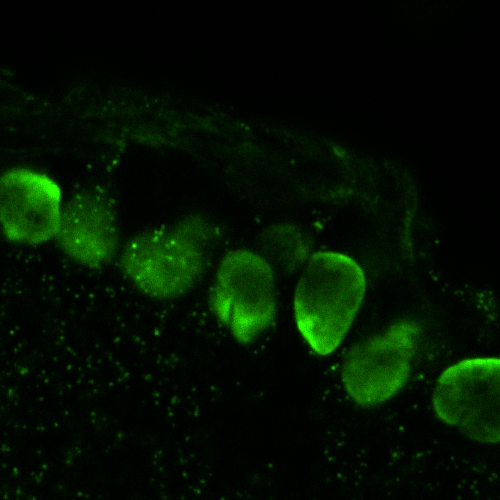

Supplement: Supplementary file 10 — Figure EV2 Source Data [file 44321_2026_438_MOESM10_ESM.zip › Figure EV2/EV2A/C2_AW7675_crop.png]

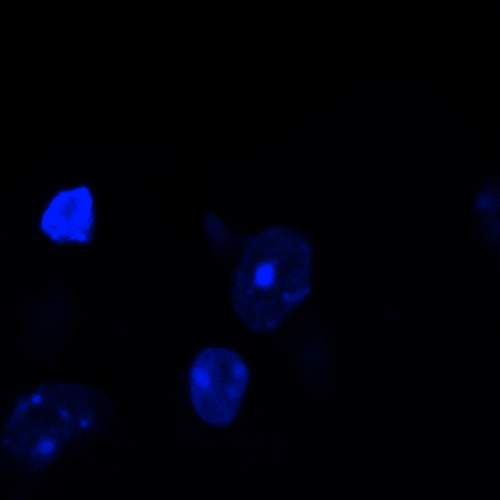

Supplement: Supplementary file 10 — Figure EV2 Source Data [file 44321_2026_438_MOESM10_ESM.zip › Figure EV2/EV2A/C3-AW7672_crop.png]

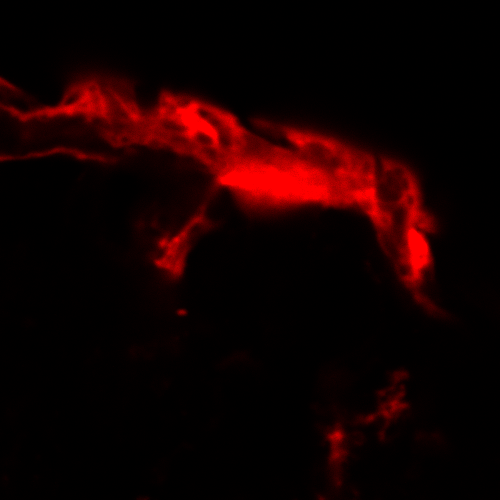

Supplement: Supplementary file 10 — Figure EV2 Source Data [file 44321_2026_438_MOESM10_ESM.zip › Figure EV2/EV2A/C3_AW7675_5crop.png]

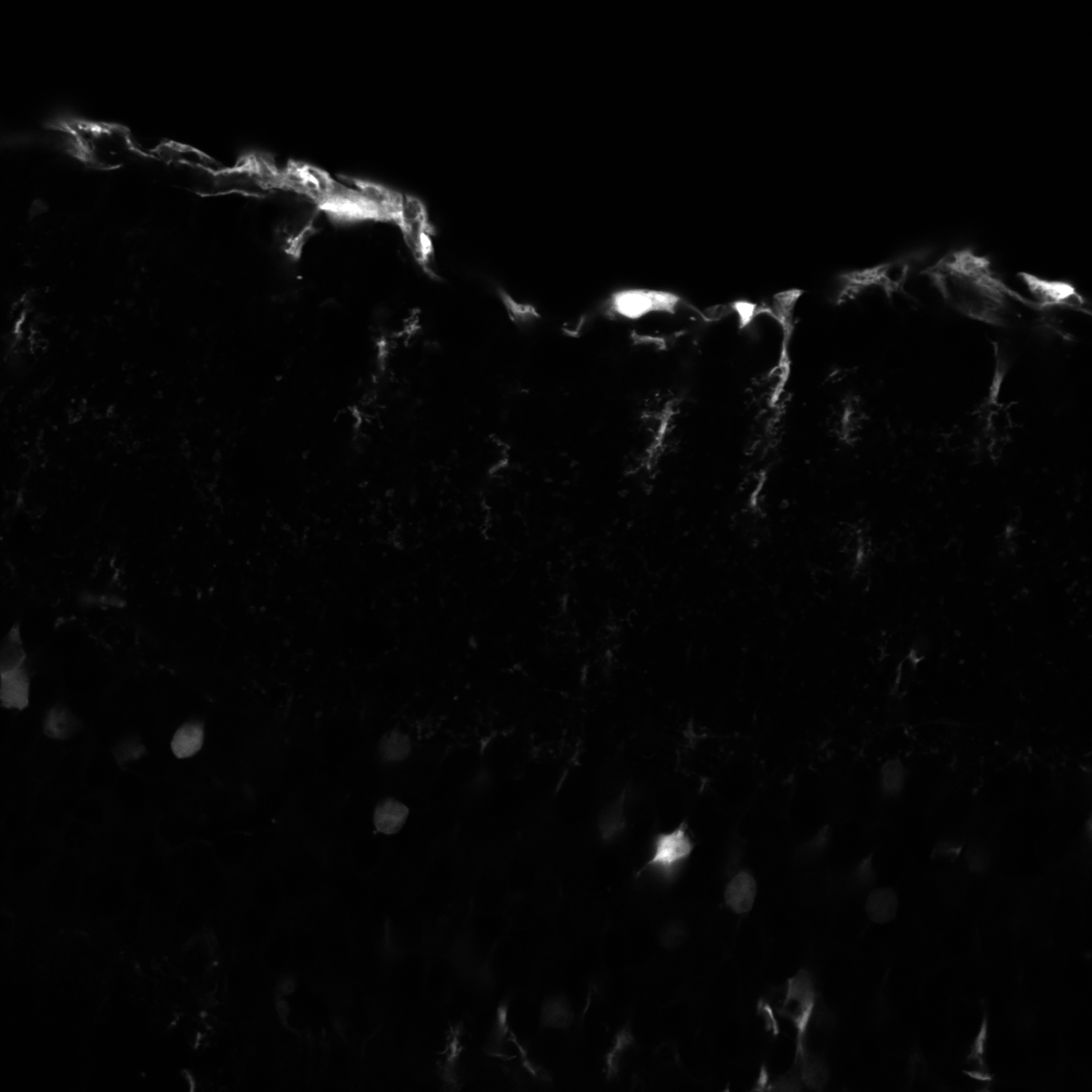

Supplement: Supplementary file 10 — Figure EV2 Source Data [file 44321_2026_438_MOESM10_ESM.zip › Figure EV2/EV2A/C3_AW7675_5u_405_DAPI_CF40_Zyla_488_GFP_CF40_Zyla_561_RFP_C..._RFP_CF40_Zyla_Retina60x_mtDNA_tomato_2.ims Resolution Level 1 Z=15 C=2.tif]

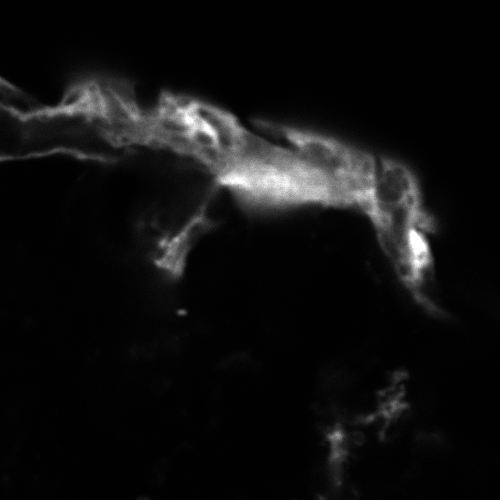

Supplement: Supplementary file 10 — Figure EV2 Source Data [file 44321_2026_438_MOESM10_ESM.zip › Figure EV2/EV2A/C3_AW7675_crop C=2.tif]

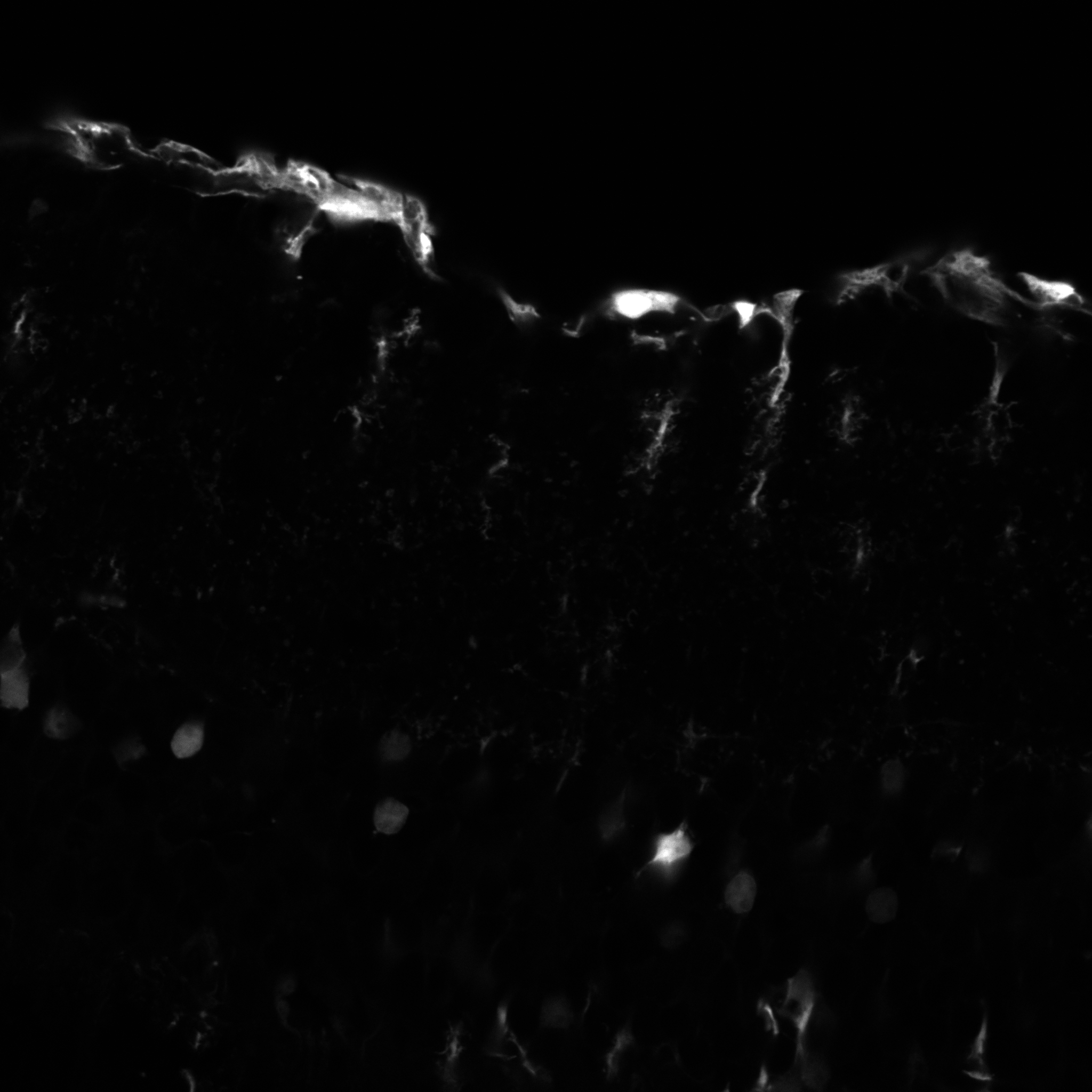

Supplement: Supplementary file 10 — Figure EV2 Source Data [file 44321_2026_438_MOESM10_ESM.zip › Figure EV2/EV2A/Composit_AW7675_5u_405_DAPI_CF40_Zyla_488_GFP_CF40_Zyla_561_RFP_C..._RFP_CF40_Zyla_Retina60x_mtDNA_tomato_2.ims Resolution Level 1 Z=15 C=2.tif]

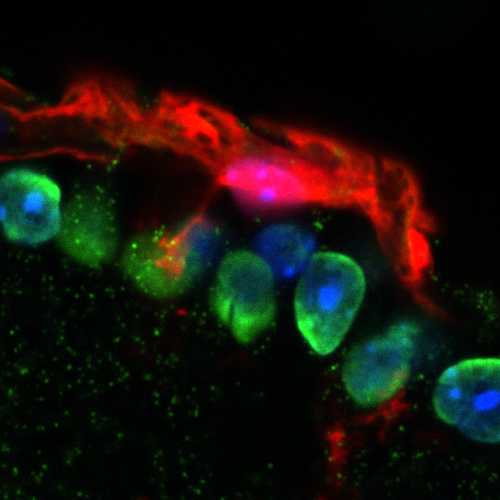

Supplement: Supplementary file 10 — Figure EV2 Source Data [file 44321_2026_438_MOESM10_ESM.zip › Figure EV2/EV2A/Composit_AW7675_crop.png]

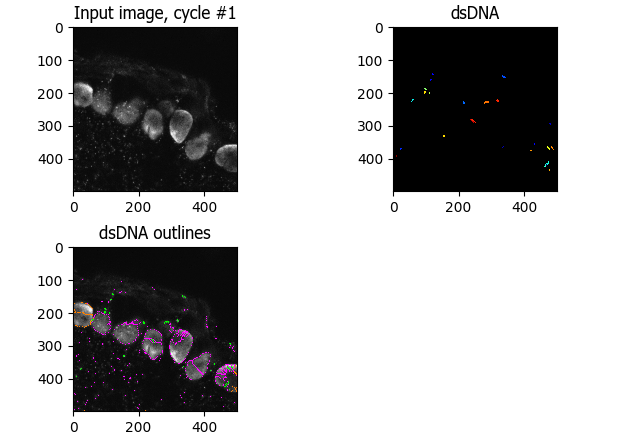

Supplement: Supplementary file 10 — Figure EV2 Source Data [file 44321_2026_438_MOESM10_ESM.zip › Figure EV2/EV2A/CP_dsDNA_AW7675_crop.png]

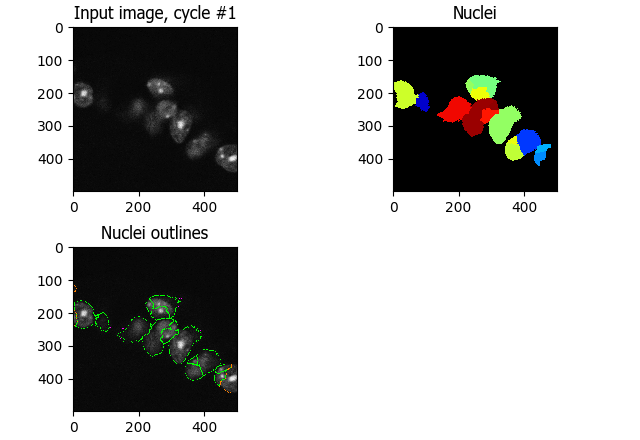

Supplement: Supplementary file 10 — Figure EV2 Source Data [file 44321_2026_438_MOESM10_ESM.zip › Figure EV2/EV2A/CP_Nuclei_AW7675_crop.png]

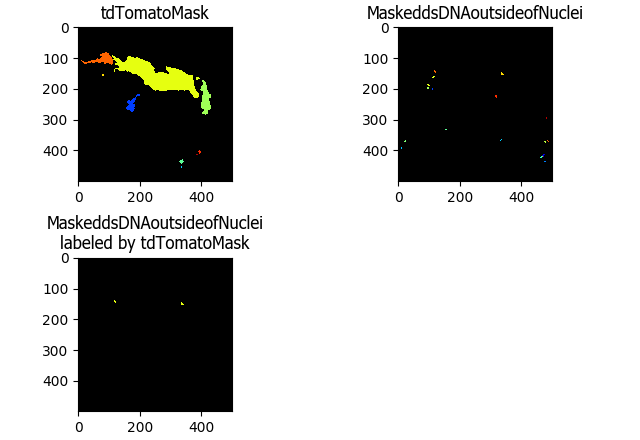

Supplement: Supplementary file 10 — Figure EV2 Source Data [file 44321_2026_438_MOESM10_ESM.zip › Figure EV2/EV2A/CP_Relate_AW7675_crop.png]

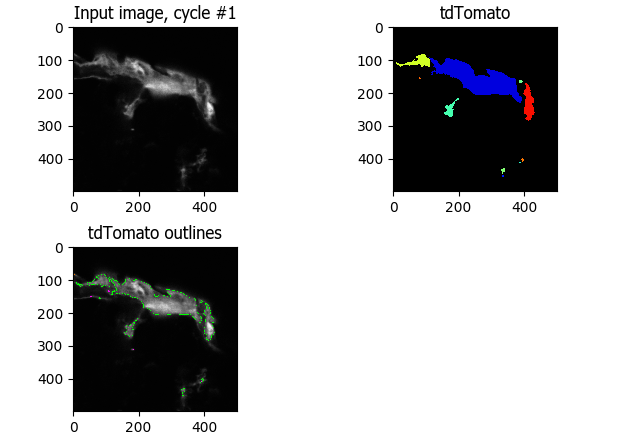

Supplement: Supplementary file 10 — Figure EV2 Source Data [file 44321_2026_438_MOESM10_ESM.zip › Figure EV2/EV2A/CP_tdTomato_AW7675_crop.png]

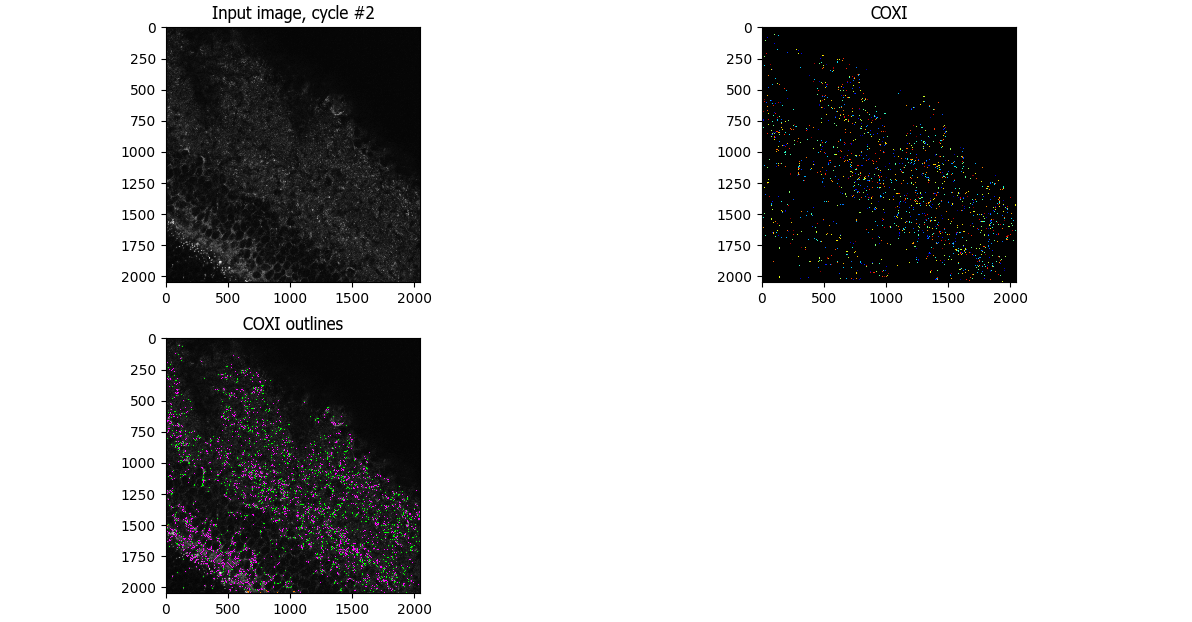

Supplement: Supplementary file 10 — Figure EV2 Source Data [file 44321_2026_438_MOESM10_ESM.zip › Figure EV2/EV2C/CP_AW7782_7_COXI.png]

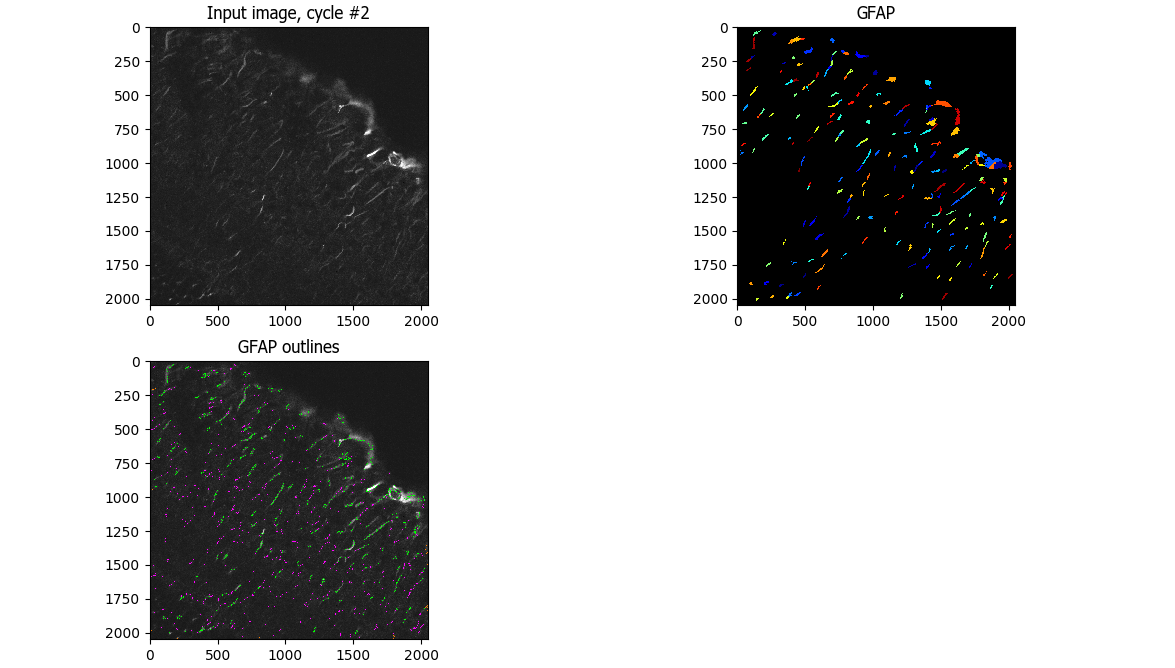

Supplement: Supplementary file 10 — Figure EV2 Source Data [file 44321_2026_438_MOESM10_ESM.zip › Figure EV2/EV2C/CP_AW7782_7_GFAP.png]

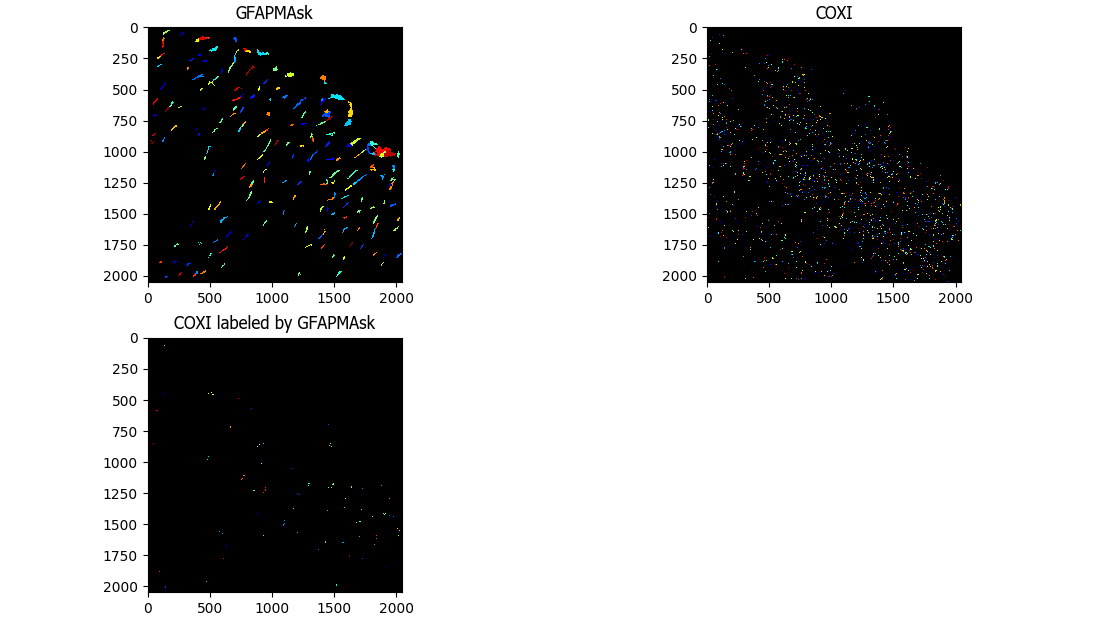

Supplement: Supplementary file 10 — Figure EV2 Source Data [file 44321_2026_438_MOESM10_ESM.zip › Figure EV2/EV2C/CP_AW7782_7_Relate.png]

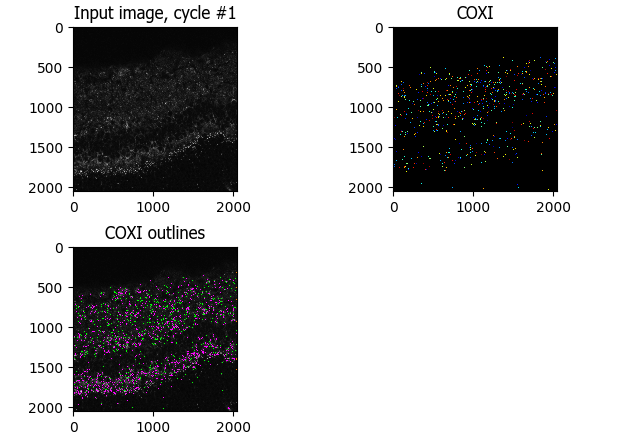

Supplement: Supplementary file 10 — Figure EV2 Source Data [file 44321_2026_438_MOESM10_ESM.zip › Figure EV2/EV2C/CP_AW7788_COXI.png]

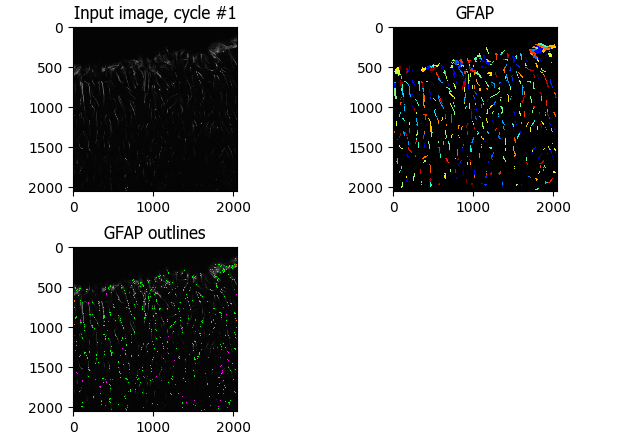

Supplement: Supplementary file 10 — Figure EV2 Source Data [file 44321_2026_438_MOESM10_ESM.zip › Figure EV2/EV2C/CP_AW7788_GFAP.png]

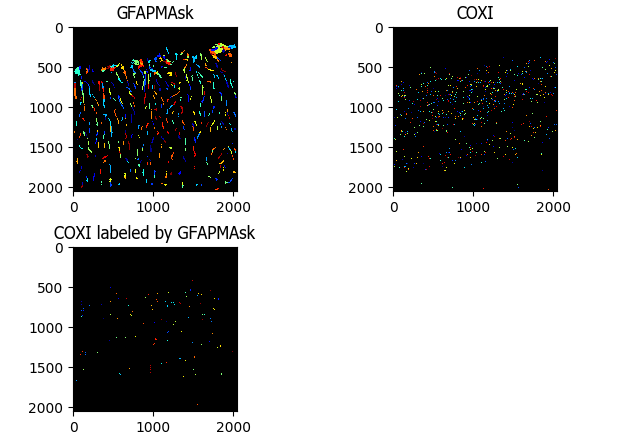

Supplement: Supplementary file 10 — Figure EV2 Source Data [file 44321_2026_438_MOESM10_ESM.zip › Figure EV2/EV2C/CP_AW7788_Relate.png]

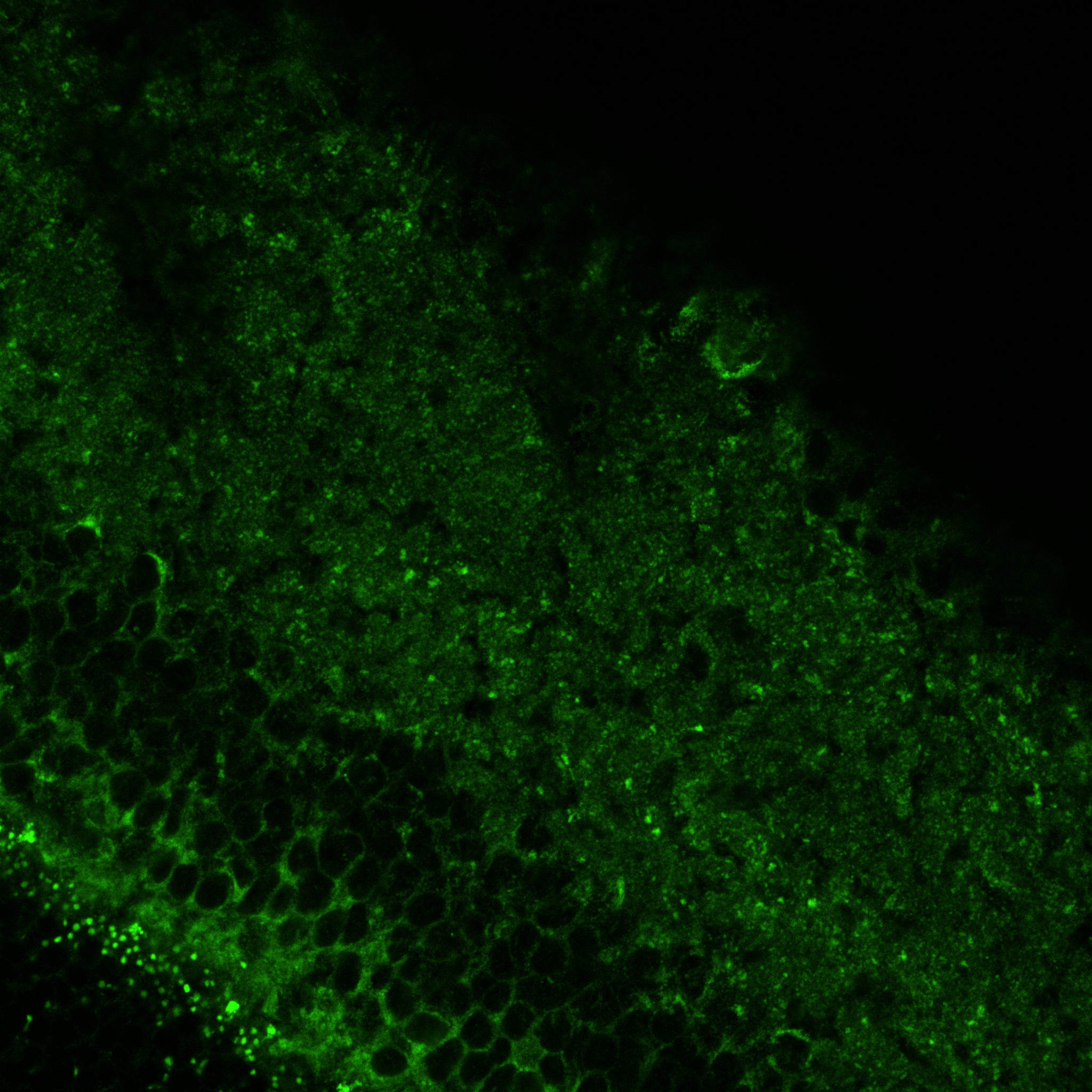

Supplement: Supplementary file 10 — Figure EV2 Source Data [file 44321_2026_438_MOESM10_ESM.zip › Figure EV2/EV2C/FS027_AW7782_7_dsDNA.png]

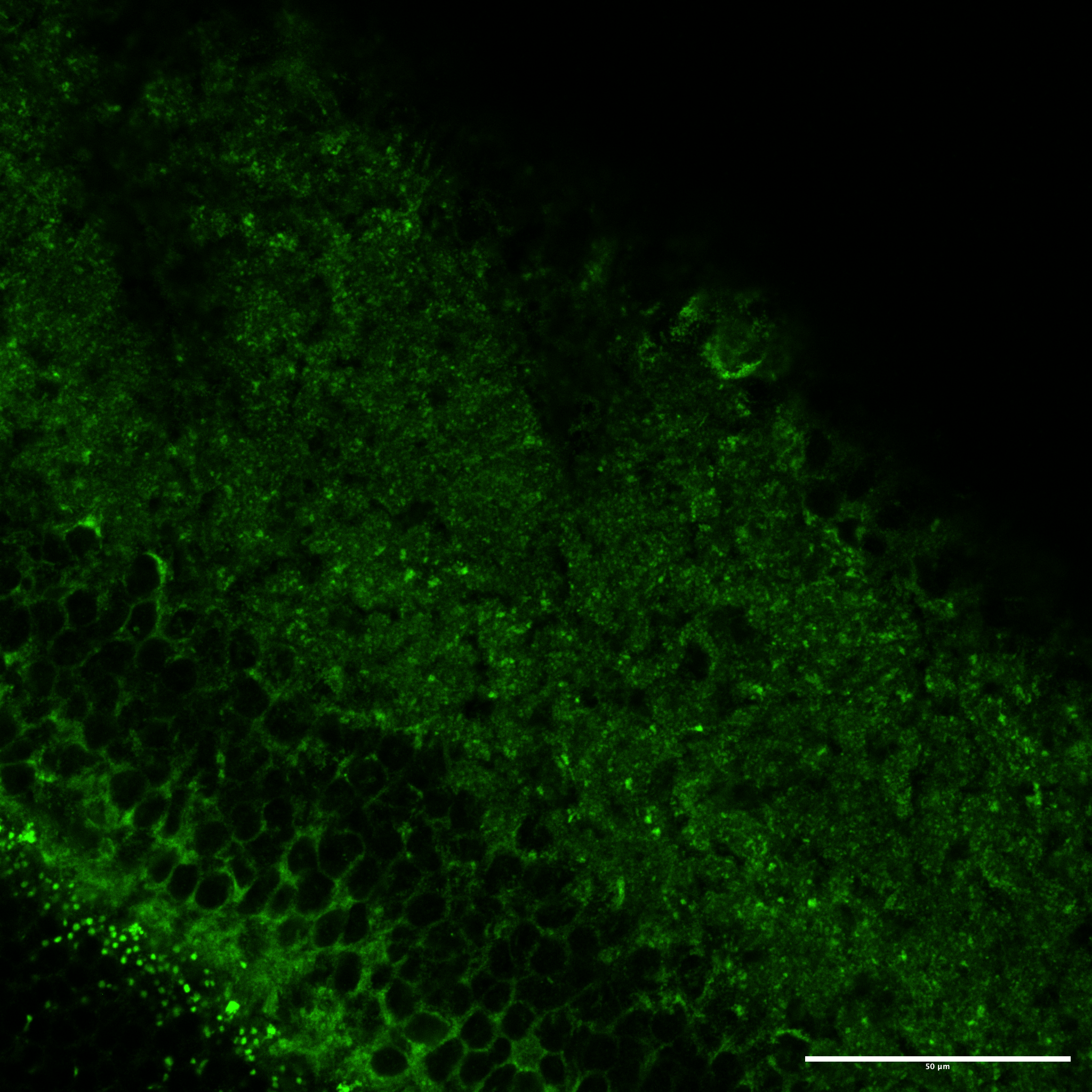

Supplement: Supplementary file 10 — Figure EV2 Source Data [file 44321_2026_438_MOESM10_ESM.zip › Figure EV2/EV2C/FS027_AW7782_7_dsDNA_scale.png]

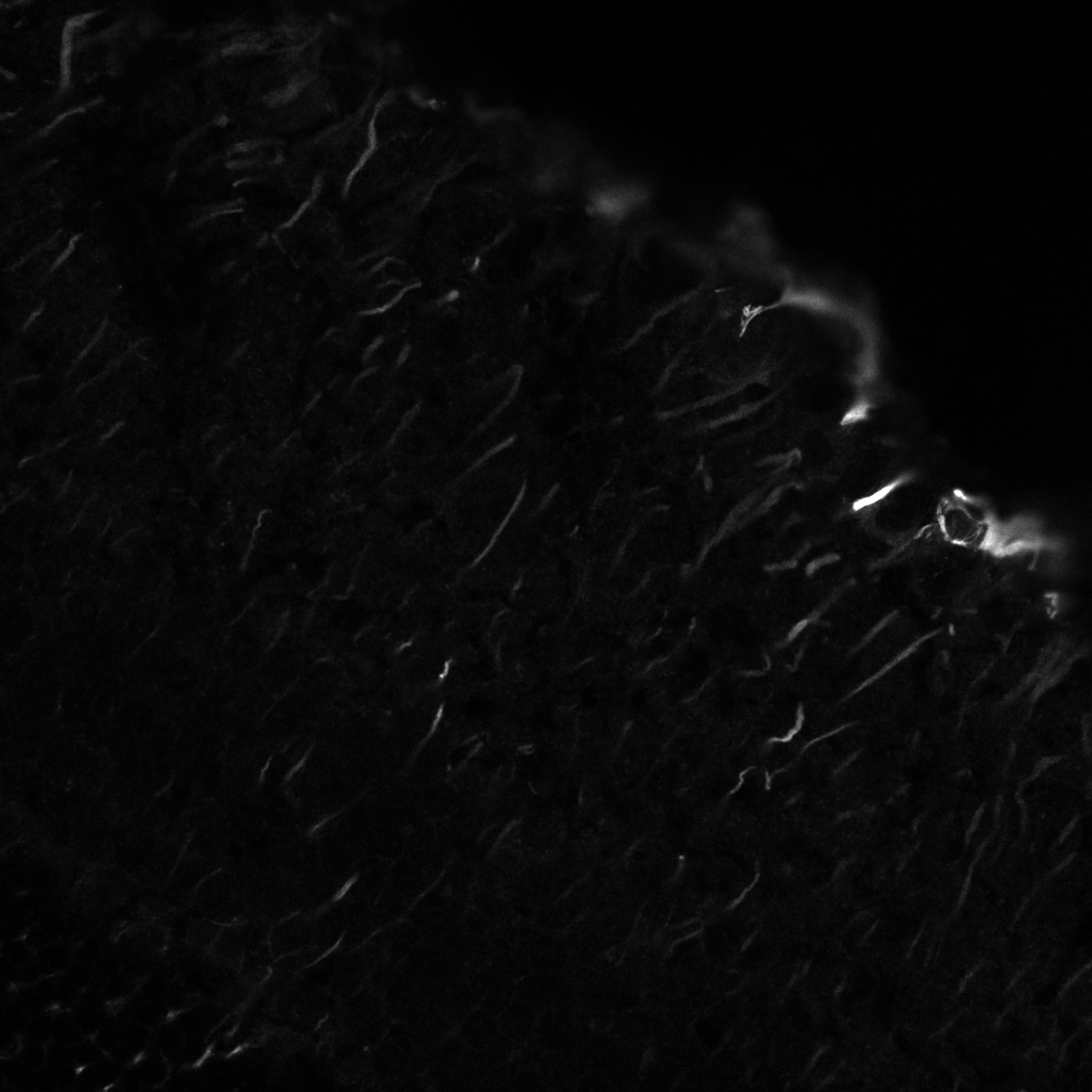

Supplement: Supplementary file 10 — Figure EV2 Source Data [file 44321_2026_438_MOESM10_ESM.zip › Figure EV2/EV2C/FS027_AW7782_7_GFAP.jpg]

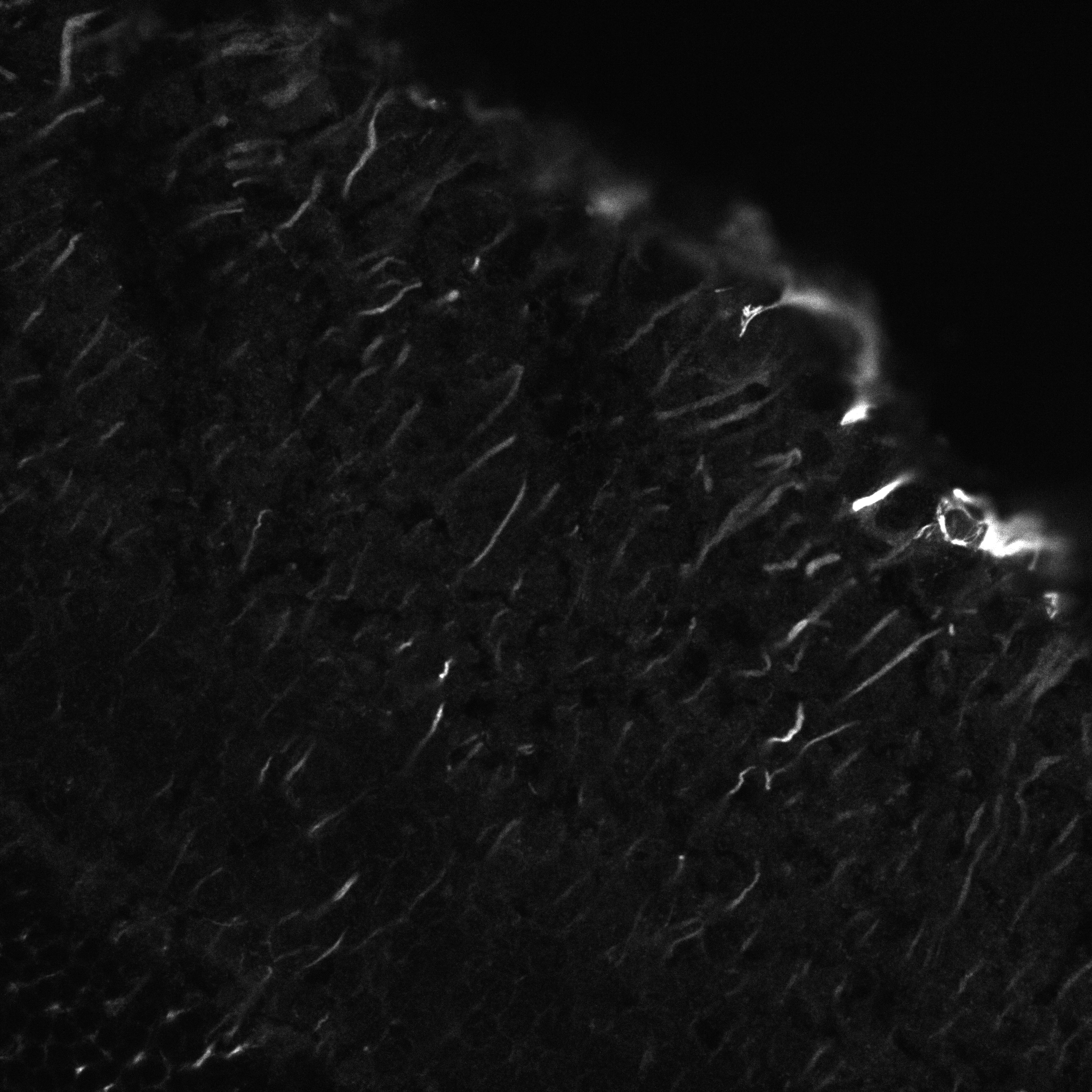

Supplement: Supplementary file 10 — Figure EV2 Source Data [file 44321_2026_438_MOESM10_ESM.zip › Figure EV2/EV2C/FS027_AW7782_7_GFAP.png]

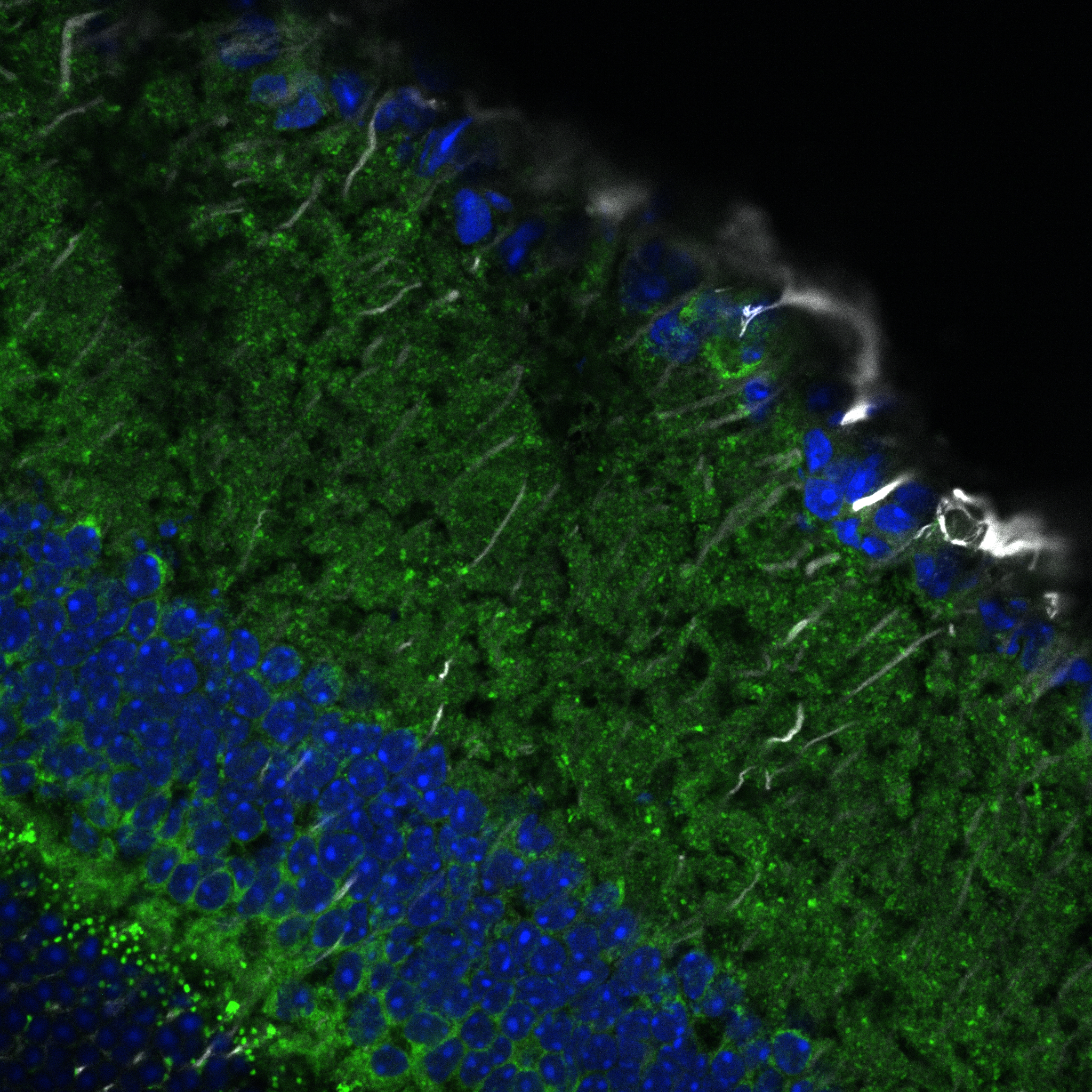

Supplement: Supplementary file 10 — Figure EV2 Source Data [file 44321_2026_438_MOESM10_ESM.zip › Figure EV2/EV2C/FS027_AW7782_7_merge.png]

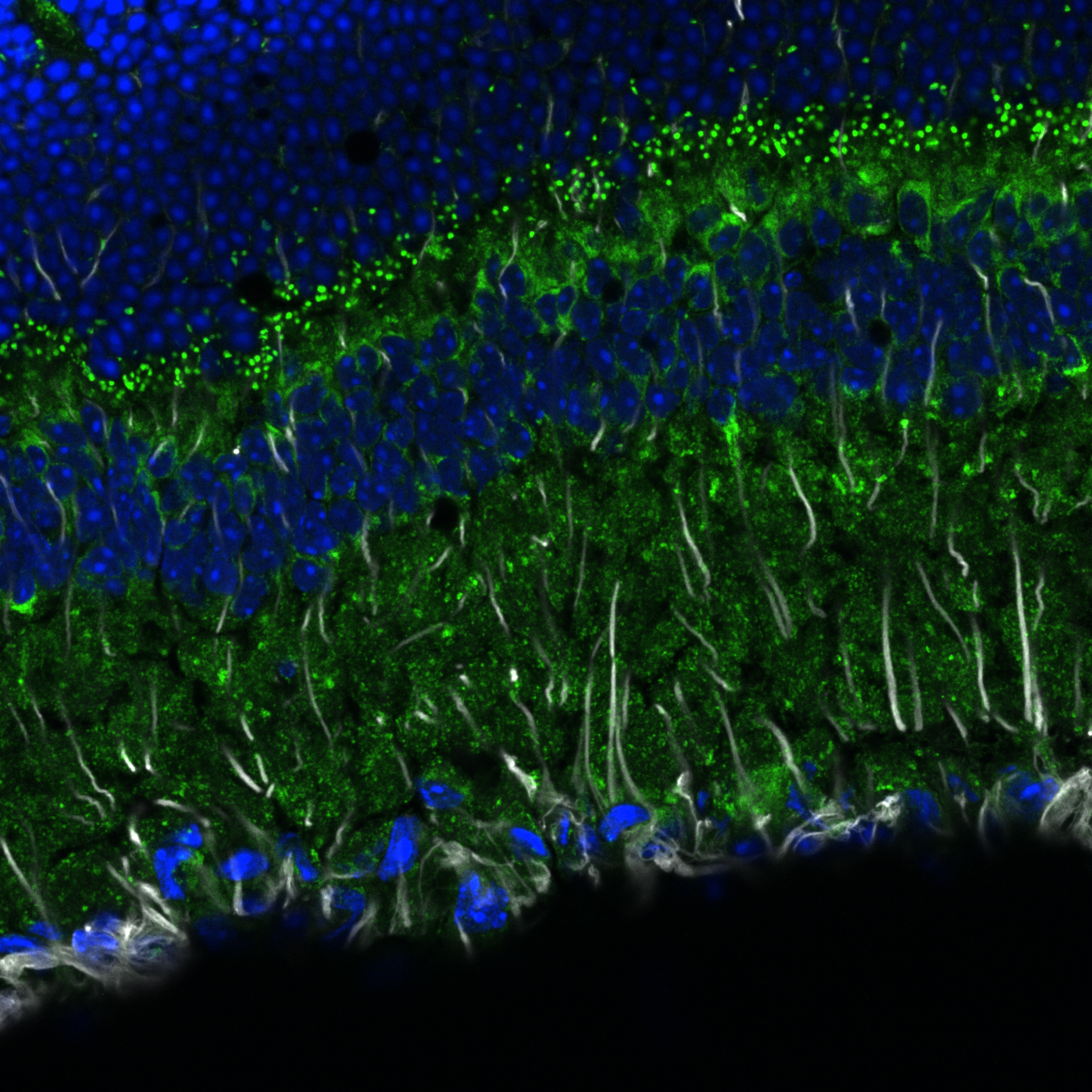

Supplement: Supplementary file 10 — Figure EV2 Source Data [file 44321_2026_438_MOESM10_ESM.zip › Figure EV2/EV2C/FS027_AW7788_composite.png]

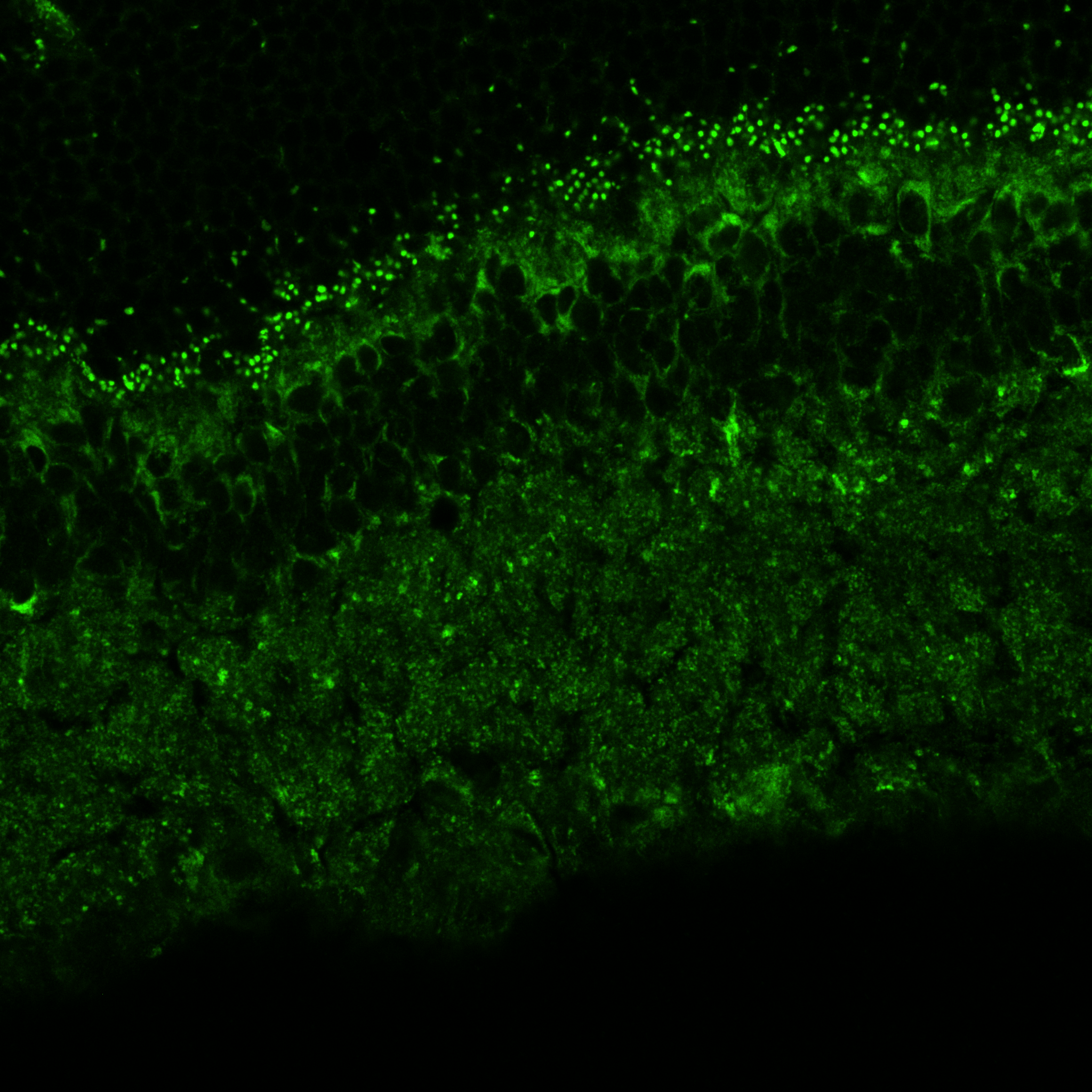

Supplement: Supplementary file 10 — Figure EV2 Source Data [file 44321_2026_438_MOESM10_ESM.zip › Figure EV2/EV2C/FS027_AW7788_COXI.png]

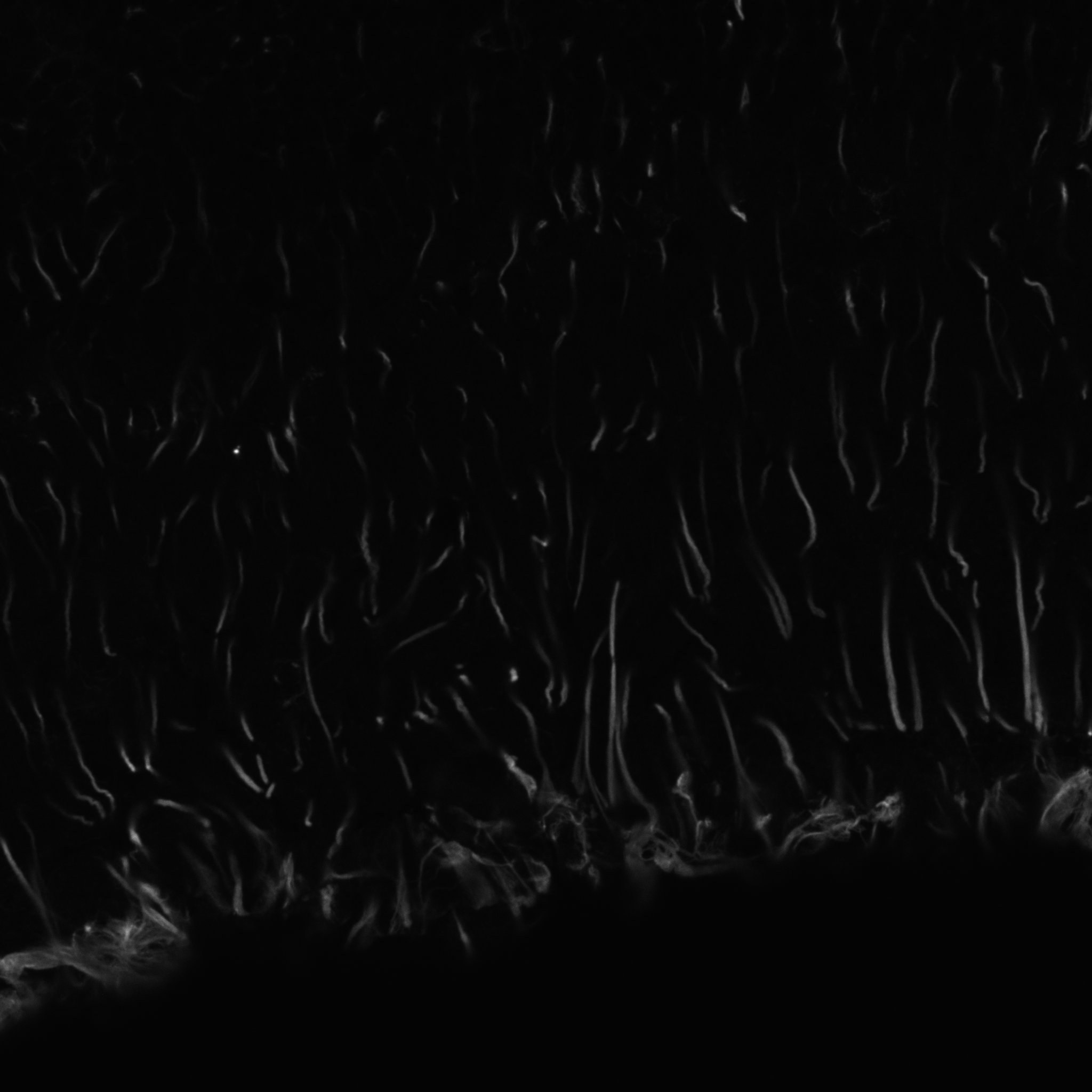

Supplement: Supplementary file 10 — Figure EV2 Source Data [file 44321_2026_438_MOESM10_ESM.zip › Figure EV2/EV2C/FS027_AW7788_GFAP.jpg]

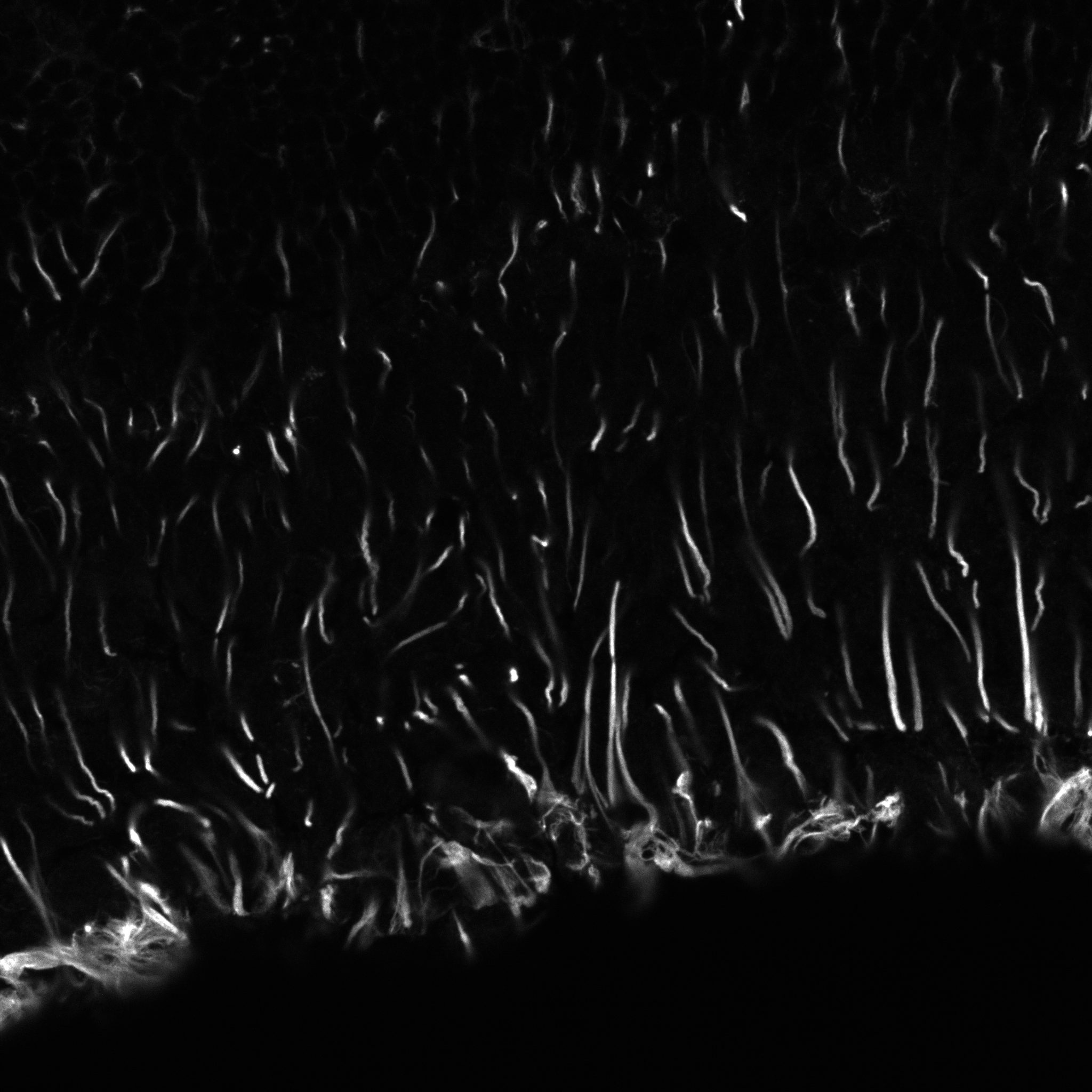

Supplement: Supplementary file 10 — Figure EV2 Source Data [file 44321_2026_438_MOESM10_ESM.zip › Figure EV2/EV2C/FS027_AW7788_GFAP.png]

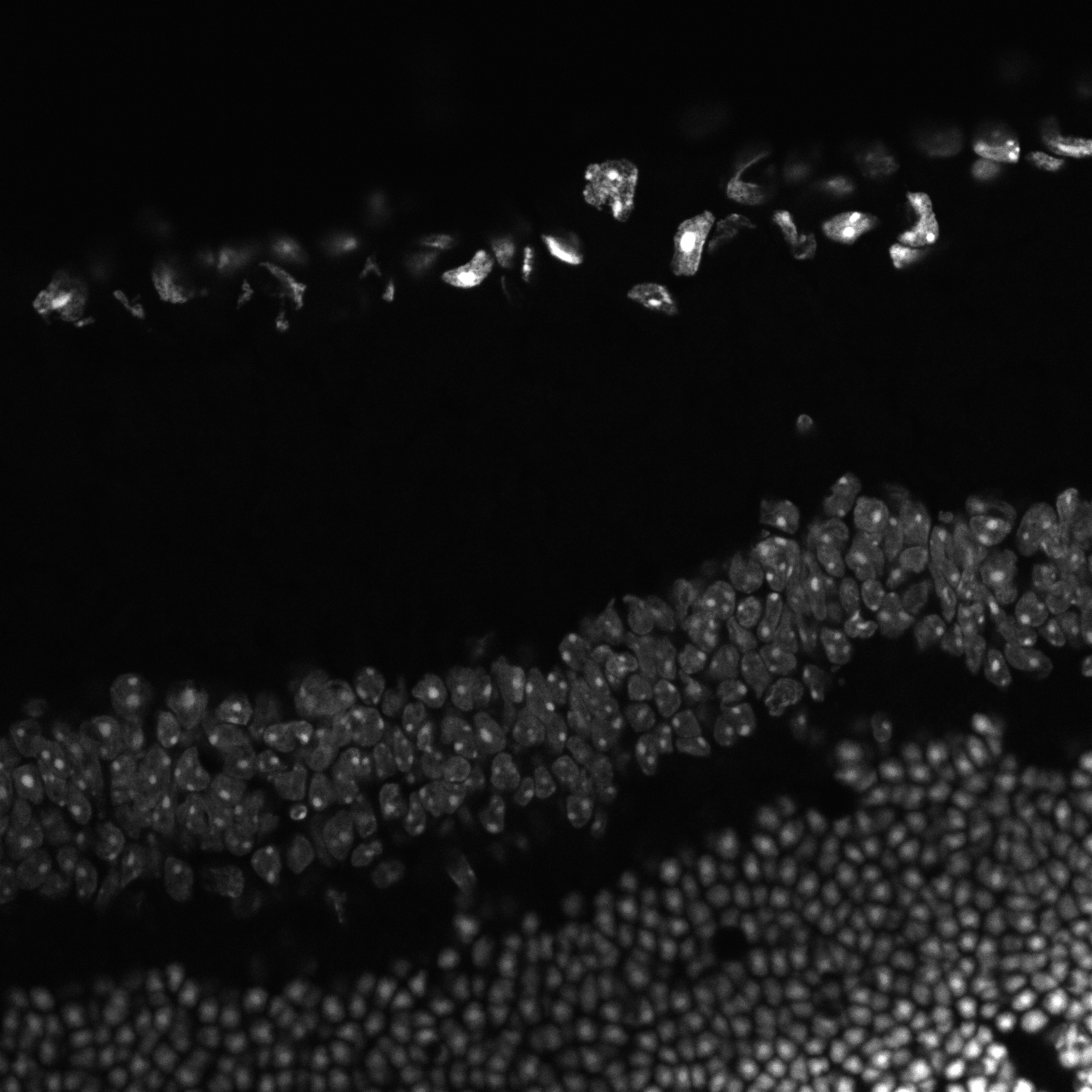

Supplement: Supplementary file 10 — Figure EV2 Source Data [file 44321_2026_438_MOESM10_ESM.zip › Figure EV2/EV2C/FS027_AW7788__405_DAPI_CF40_Zyla_488_GFP_CF40_Zyla_640_Cy5_CF4...640_Cy5_CF40_Zyla_Retina60x_COX1_GFAP_1.ims Resolution Level 1 Z=15 C=0.tif]

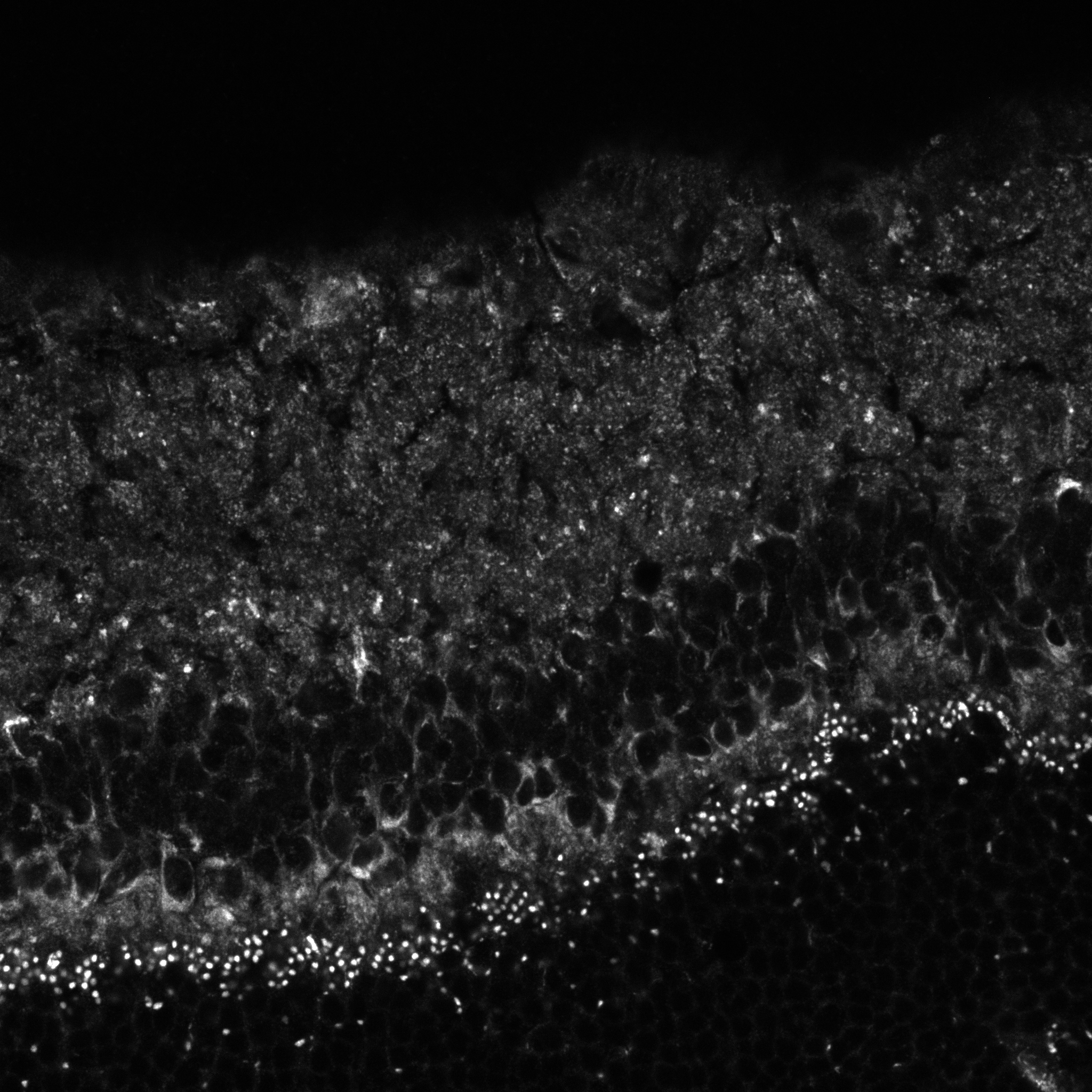

Supplement: Supplementary file 10 — Figure EV2 Source Data [file 44321_2026_438_MOESM10_ESM.zip › Figure EV2/EV2C/FS027_AW7788__405_DAPI_CF40_Zyla_488_GFP_CF40_Zyla_640_Cy5_CF4...640_Cy5_CF40_Zyla_Retina60x_COX1_GFAP_1.ims Resolution Level 1 Z=15 C=1.tif]

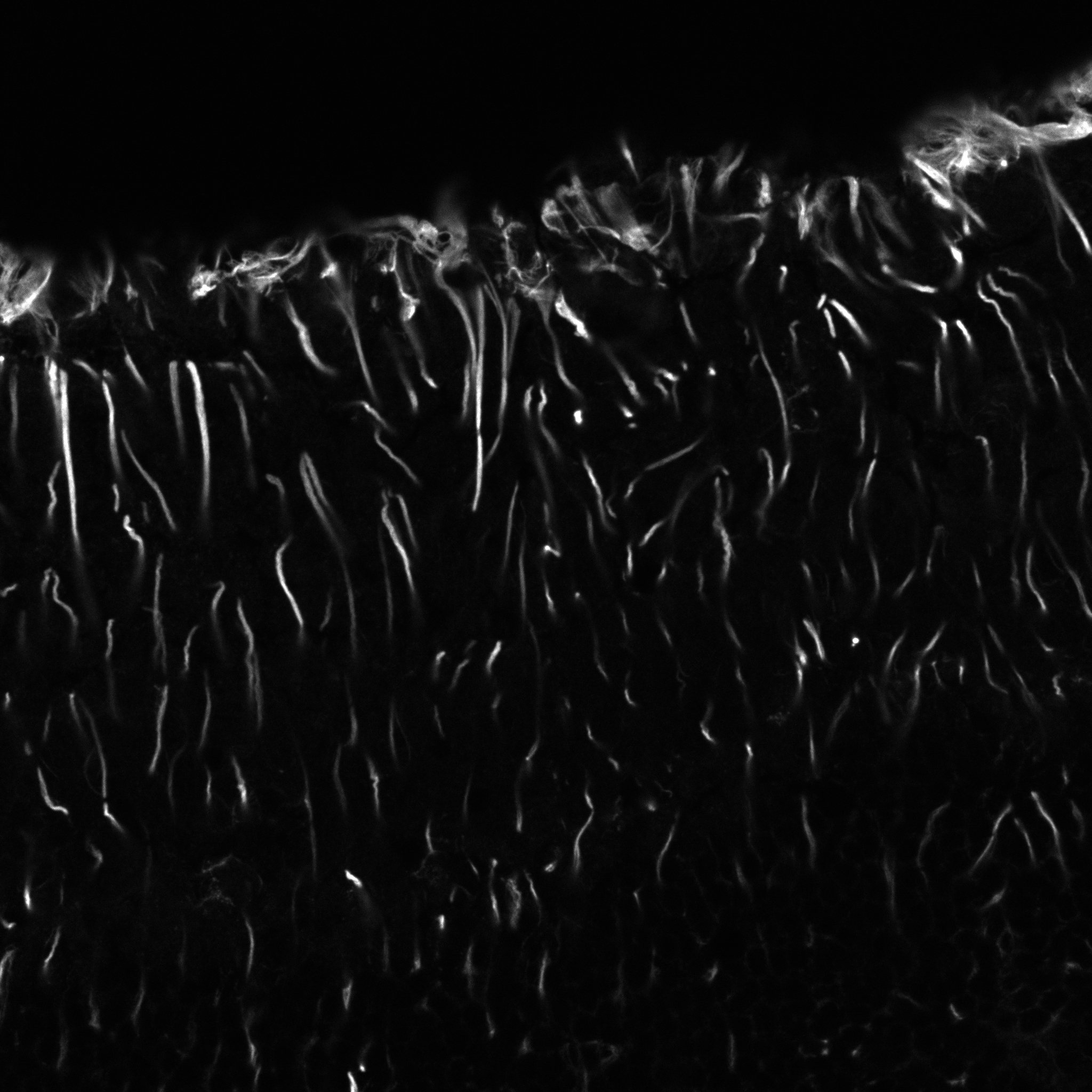

Supplement: Supplementary file 10 — Figure EV2 Source Data [file 44321_2026_438_MOESM10_ESM.zip › Figure EV2/EV2C/FS027_AW7788__405_DAPI_CF40_Zyla_488_GFP_CF40_Zyla_640_Cy5_CF4...640_Cy5_CF40_Zyla_Retina60x_COX1_GFAP_1.ims Resolution Level 1 Z=15 C=2.tif]

# dsDNA in astrocytes

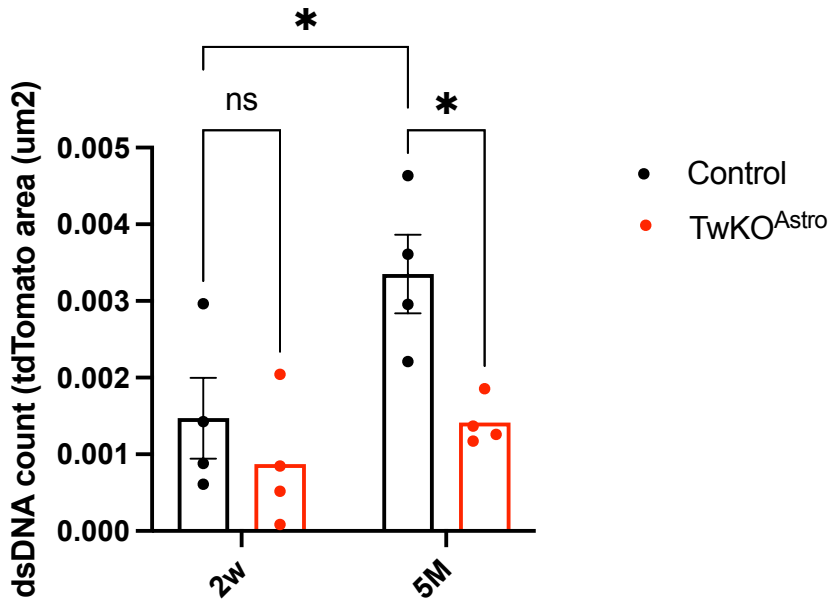

Supplement: Supplementary file 10 — Figure EV2 Source Data [file 44321_2026_438_MOESM10_ESM.zip › Figure EV2/EV2B/EV2B.pdf]

# COXI/GFAP

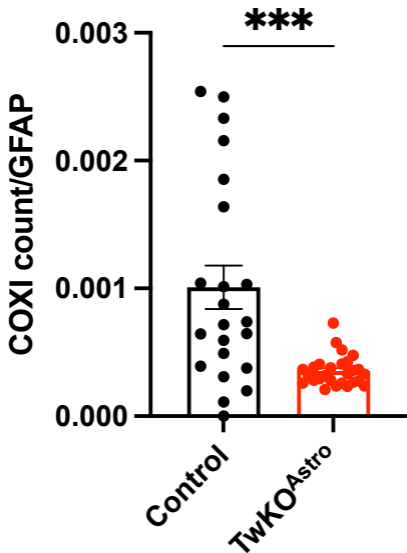

Supplement: Supplementary file 10 — Figure EV2 Source Data [file 44321_2026_438_MOESM10_ESM.zip › Figure EV2/EV2D/EV2D.pdf]

# GFAP area

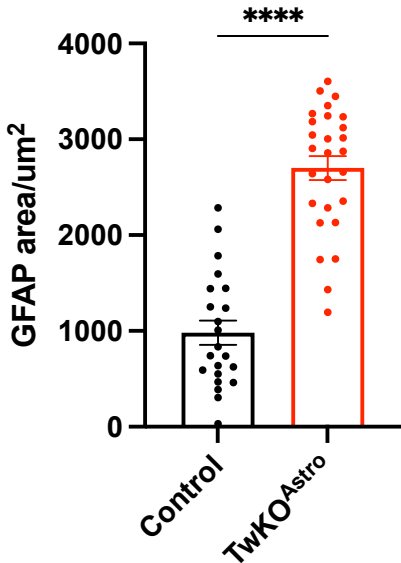

Supplement: Supplementary file 11 — Figure EV3 Source Data [file 44321_2026_438_MOESM11_ESM.zip › Figure EV3/EV3 C.pdf]

# FJC intensity in tdTomato

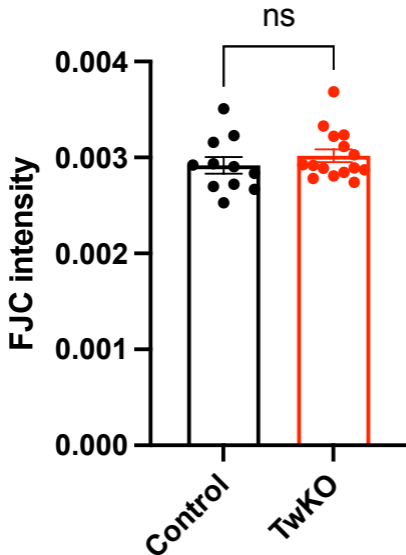

Supplement: Supplementary file 11 — Figure EV3 Source Data [file 44321_2026_438_MOESM11_ESM.zip › Figure EV3/EV3B.pdf]

Microglia/field of view

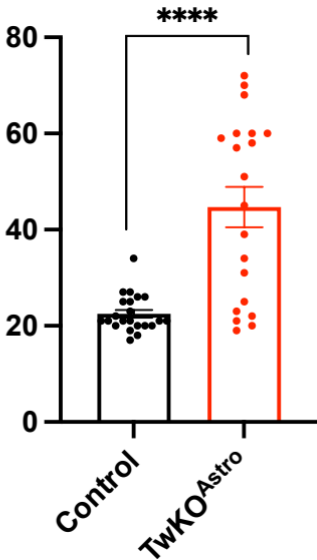

Supplement: Supplementary file 11 — Figure EV3 Source Data [file 44321_2026_438_MOESM11_ESM.zip › Figure EV3/EV3D.pdf]

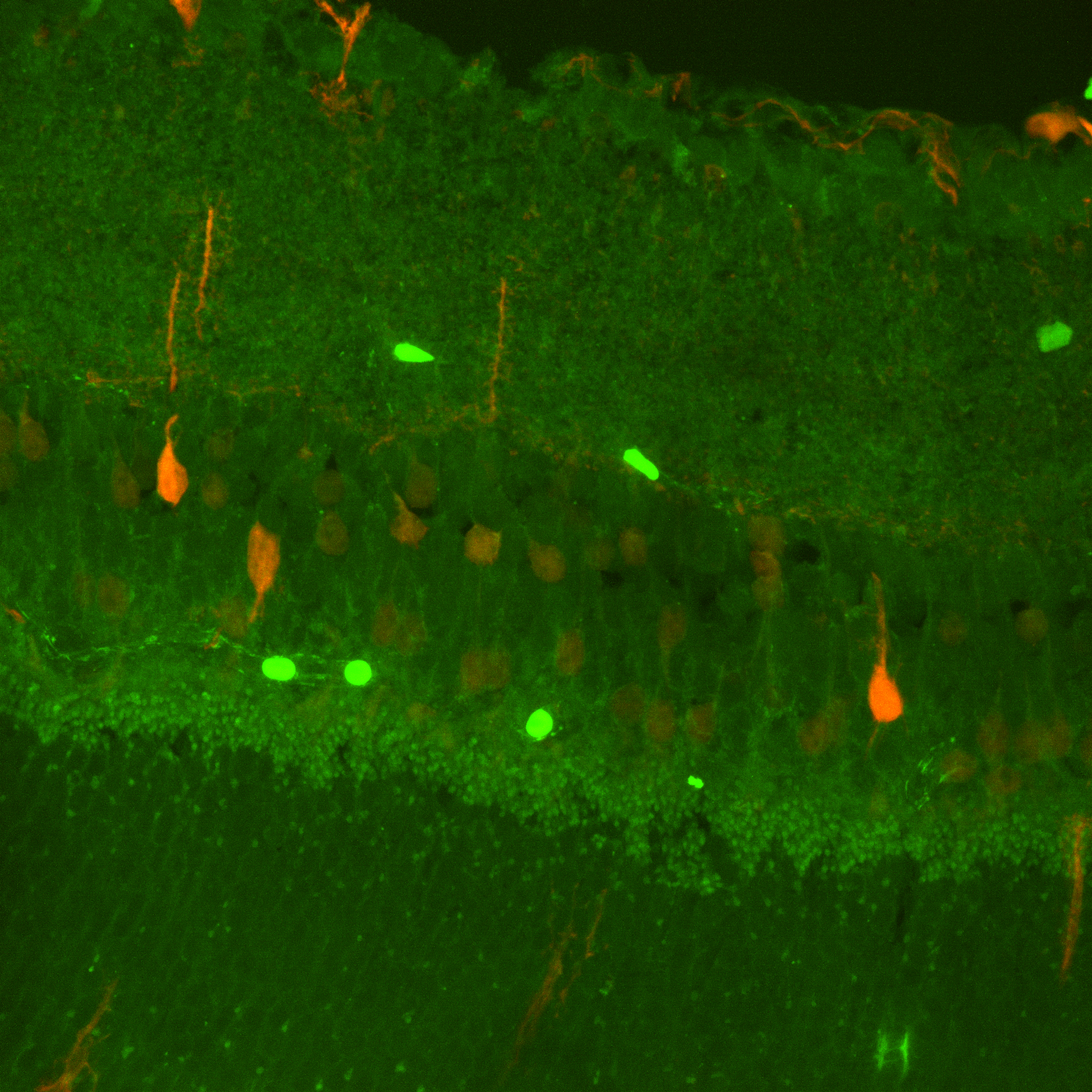

Supplement: Supplementary file 11 — Figure EV3 Source Data [file 44321_2026_438_MOESM11_ESM.zip › Figure EV3/EV3A/FS024_AW7672 Comp.tif]

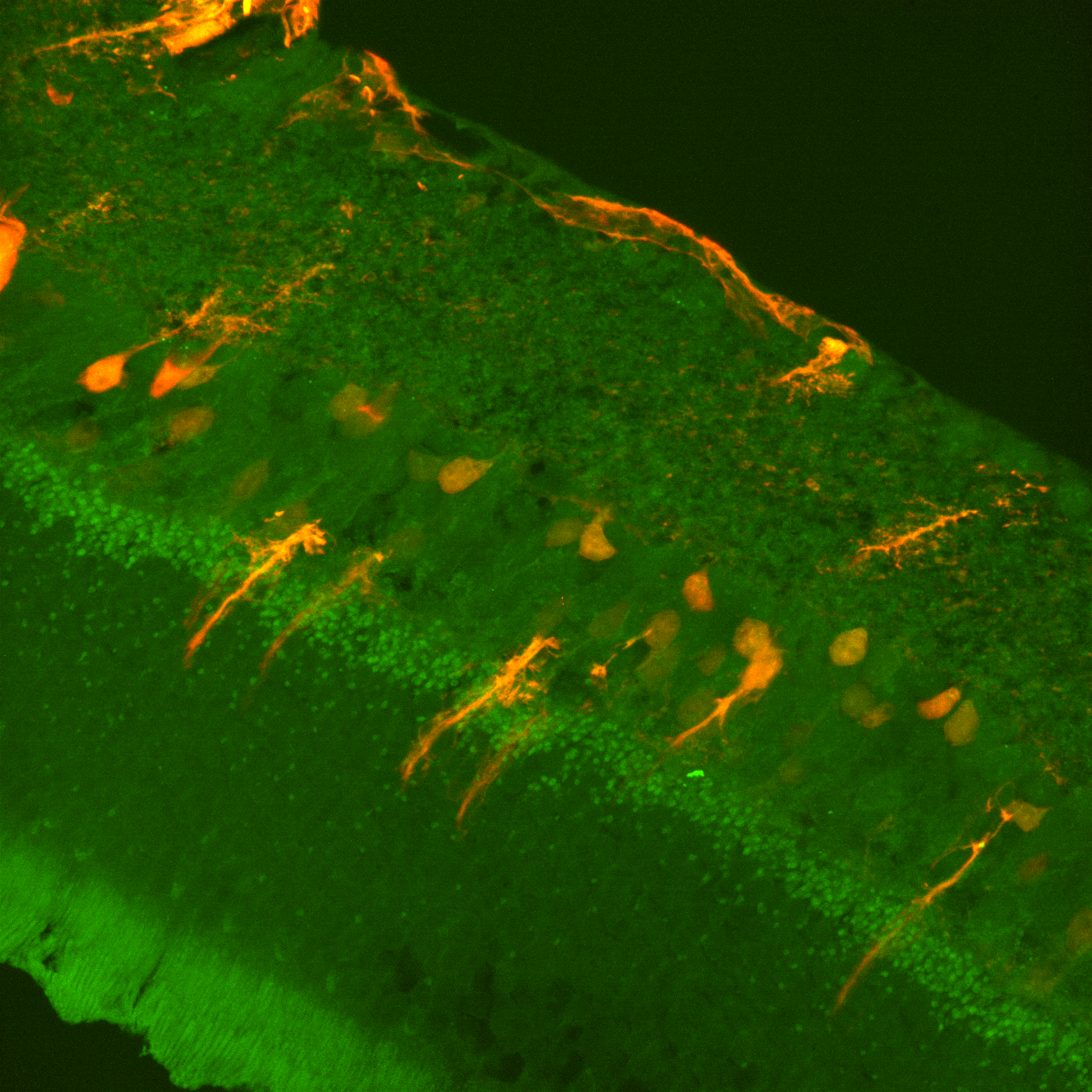

Supplement: Supplementary file 11 — Figure EV3 Source Data [file 44321_2026_438_MOESM11_ESM.zip › Figure EV3/EV3A/FS024_AW7676 Comp.tif]

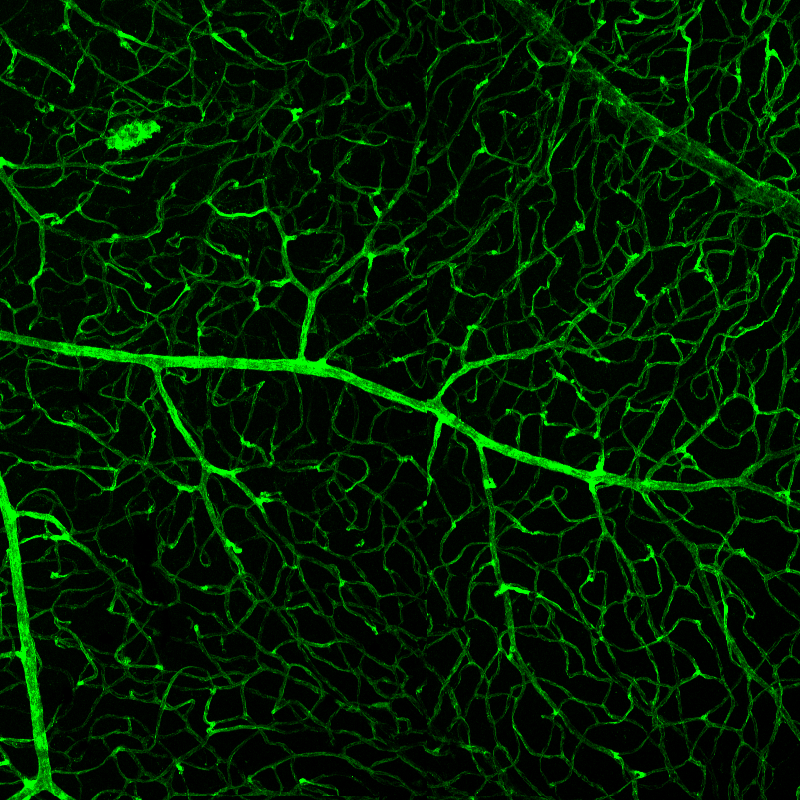

Supplement: Supplementary file 12 — Figure EV4 Source Data [file 44321_2026_438_MOESM12_ESM.zip › Figure EV4/EV4A/AW4229C_IB4_GFAP_10x_zstack_Maximum intensity projection-Create Image Subset-39.tif]

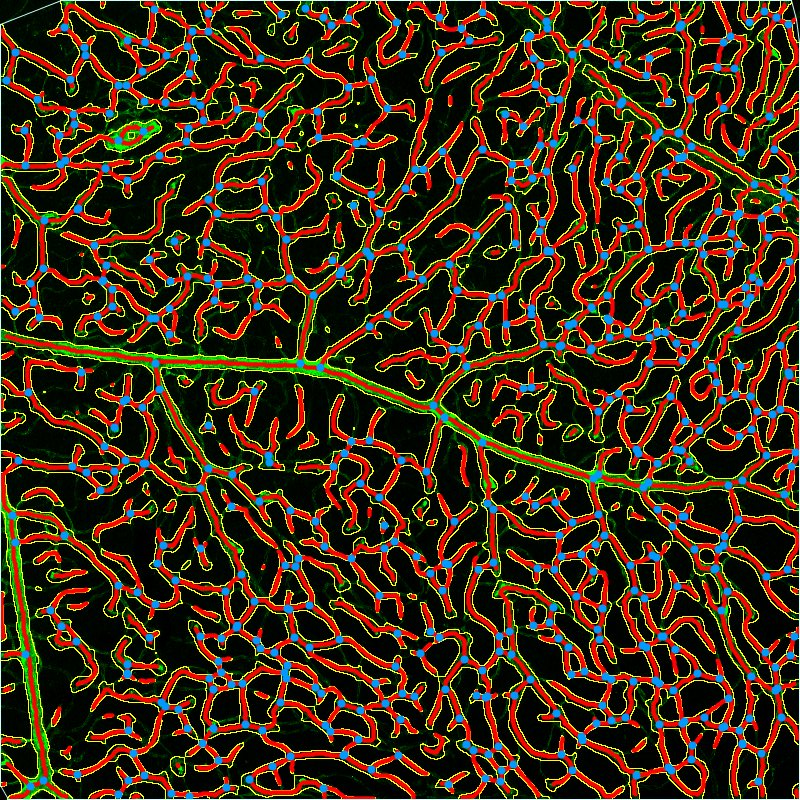

Supplement: Supplementary file 12 — Figure EV4 Source Data [file 44321_2026_438_MOESM12_ESM.zip › Figure EV4/EV4A/AW4229C_IB4_GFAP_10x_zstack_Maximum intensity projection-Create Image Subset-39.tif result.tif]

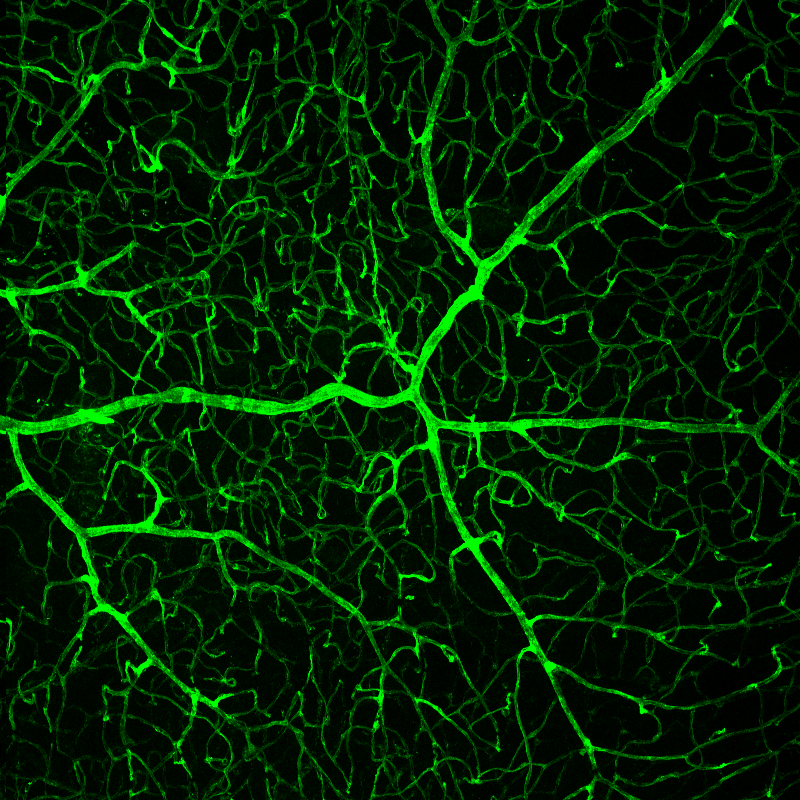

Supplement: Supplementary file 12 — Figure EV4 Source Data [file 44321_2026_438_MOESM12_ESM.zip › Figure EV4/EV4A/AW4233CT_IB4_GFAP_10x_zstack_Maximum intensity projection-Create Image Subset-31.tif]

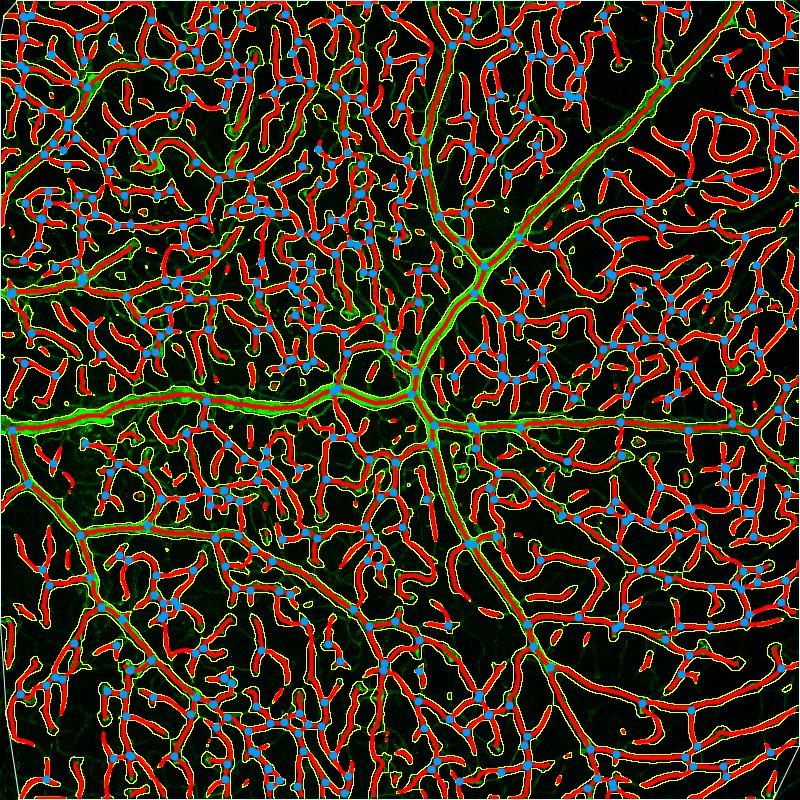

Supplement: Supplementary file 12 — Figure EV4 Source Data [file 44321_2026_438_MOESM12_ESM.zip › Figure EV4/EV4A/AW4233CT_IB4_GFAP_10x_zstack_Maximum intensity projection-Create Image Subset-31.tif result.tif]

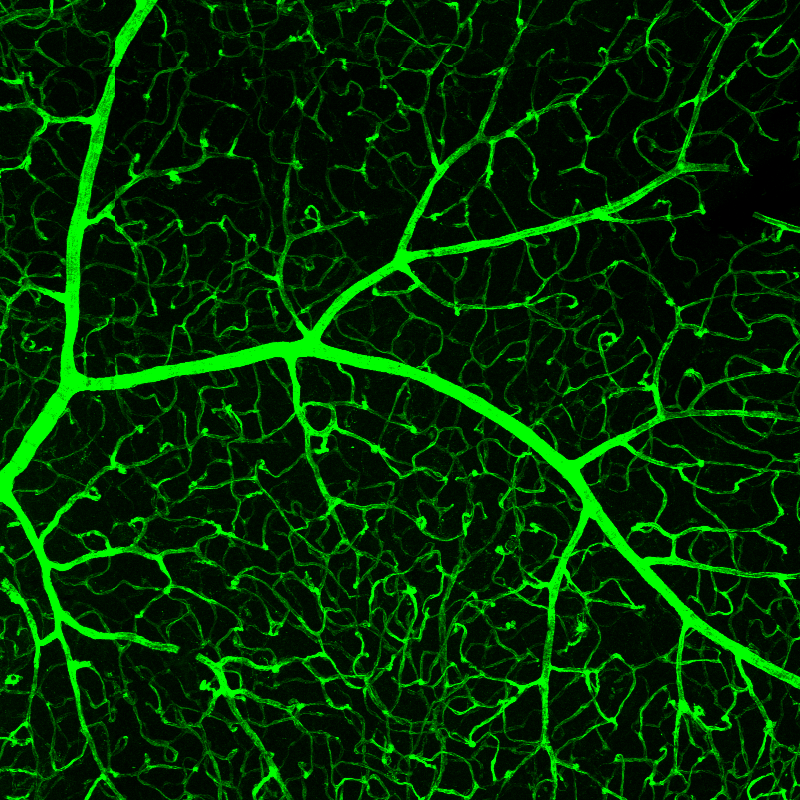

Supplement: Supplementary file 12 — Figure EV4 Source Data [file 44321_2026_438_MOESM12_ESM.zip › Figure EV4/EV4C/MUT3403_IB4_GFAP_10x_Z-stack_Tiles_Maximum intensity projection-Create Image Subset-44 copy.tif]

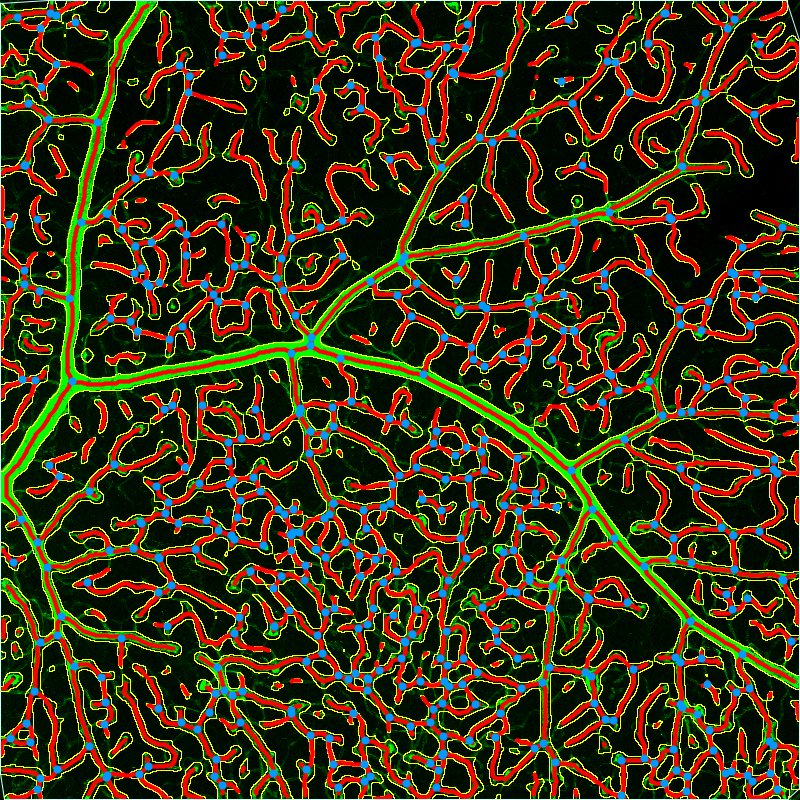

Supplement: Supplementary file 12 — Figure EV4 Source Data [file 44321_2026_438_MOESM12_ESM.zip › Figure EV4/EV4C/MUT3403_IB4_GFAP_10x_Z-stack_Tiles_Maximum intensity projection-Create Image Subset-44.tif result copy.jpg]

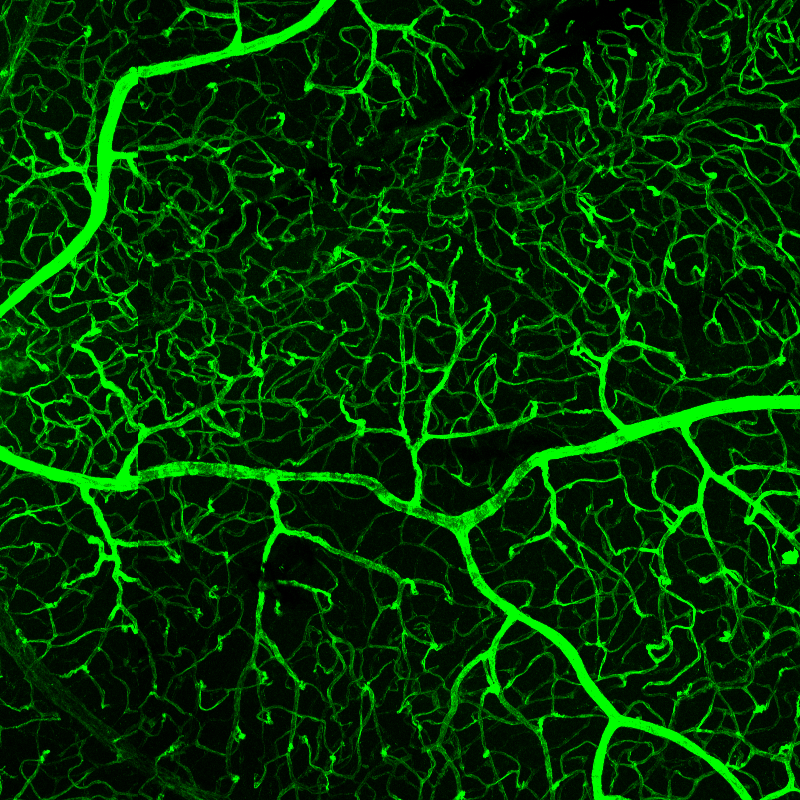

Supplement: Supplementary file 12 — Figure EV4 Source Data [file 44321_2026_438_MOESM12_ESM.zip › Figure EV4/EV4C/MUT3421_IB4_GFAP_10x_Z-stack_Tiles_Maximum intensity projection-Create Image Subset-34 copy.tif]

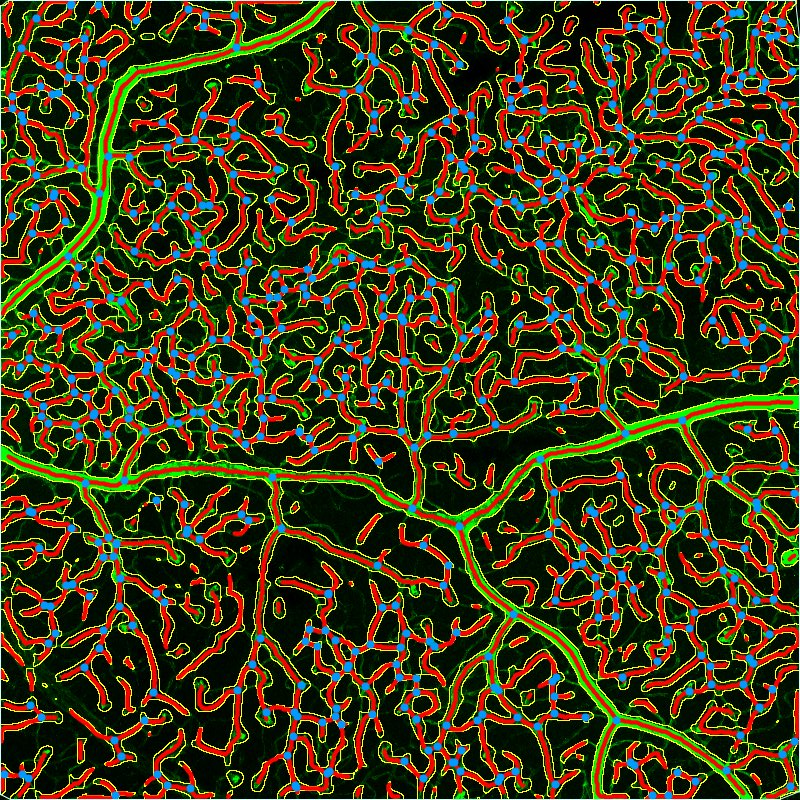

Supplement: Supplementary file 12 — Figure EV4 Source Data [file 44321_2026_438_MOESM12_ESM.zip › Figure EV4/EV4C/MUT3421_IB4_GFAP_10x_Z-stack_Tiles_Maximum intensity projection-Create Image Subset-34.tif result copy.tif]

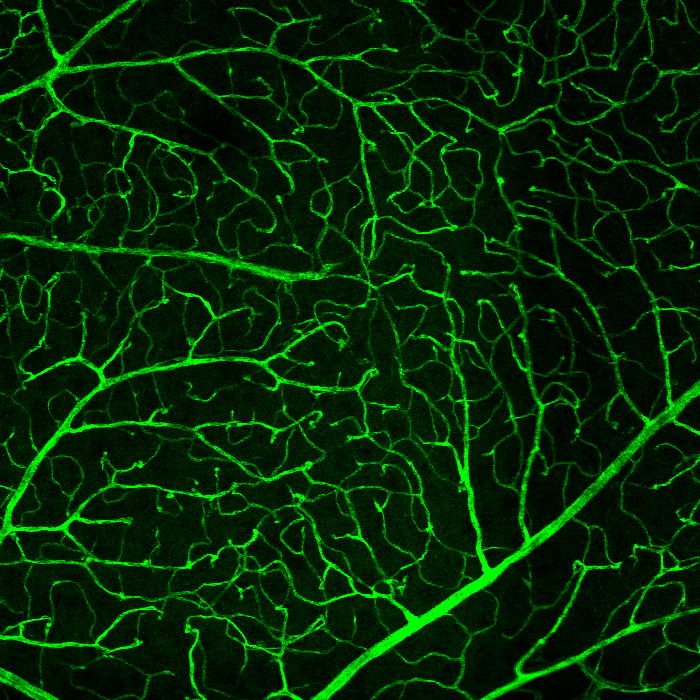

Supplement: Supplementary file 12 — Figure EV4 Source Data [file 44321_2026_438_MOESM12_ESM.zip › Figure EV4/EV4E/Ndufs4_3516(Ct)_IB4_10x_Maximum intensity projection.tif]

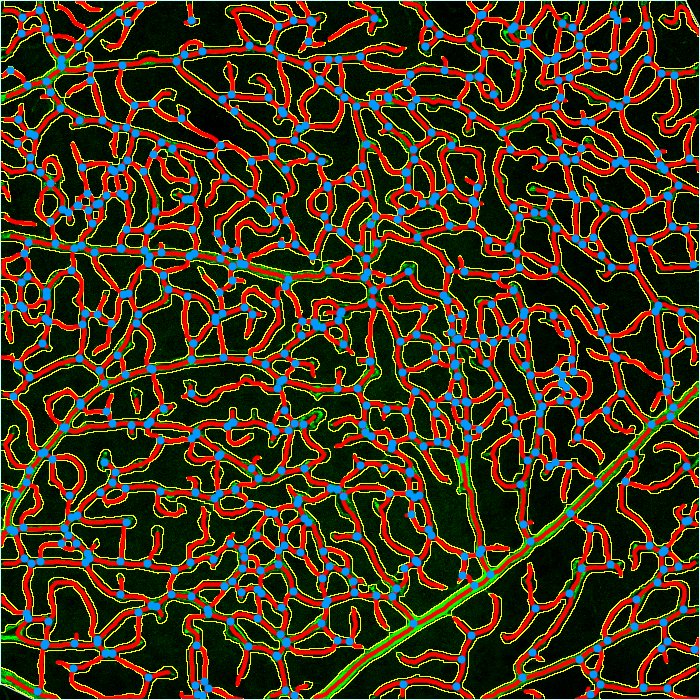

Supplement: Supplementary file 12 — Figure EV4 Source Data [file 44321_2026_438_MOESM12_ESM.zip › Figure EV4/EV4E/Ndufs4_3516(Ct)_IB4_10x_Maximum intensity projection_AngioTool.jpg]

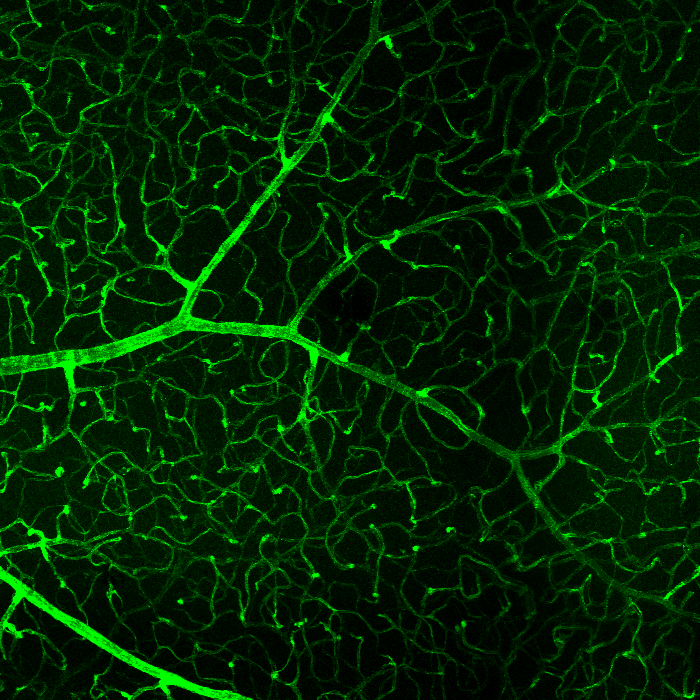

Supplement: Supplementary file 12 — Figure EV4 Source Data [file 44321_2026_438_MOESM12_ESM.zip › Figure EV4/EV4E/Ndufs4_3521(KO)_IB4_10x_Maximum intensity projection.tif]

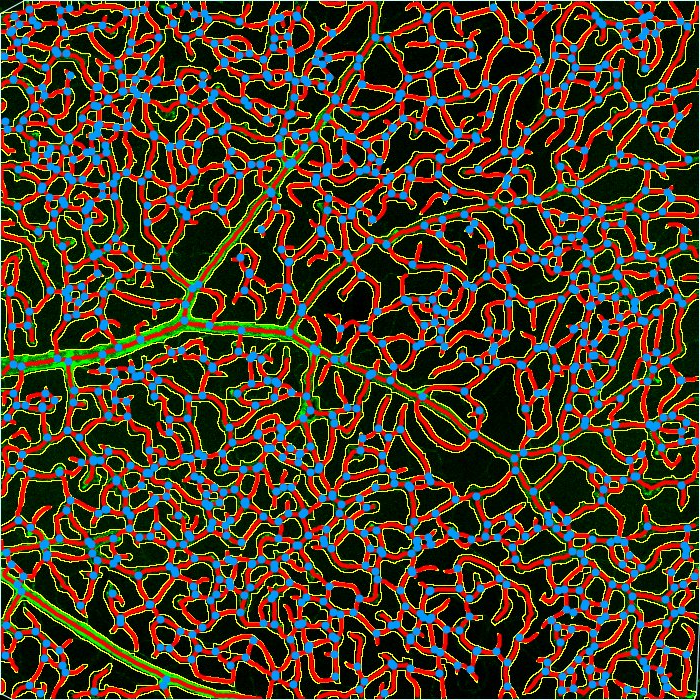

Supplement: Supplementary file 12 — Figure EV4 Source Data [file 44321_2026_438_MOESM12_ESM.zip › Figure EV4/EV4E/Ndufs4_3521(KO)_IB4_10x_Maximum intensity projection_AngioTool.tif]

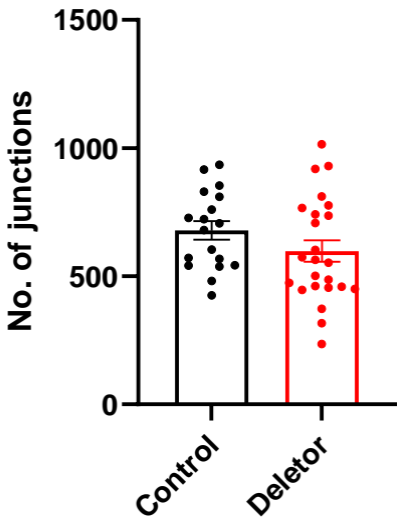

Supplement: Supplementary file 12 — Figure EV4 Source Data [file 44321_2026_438_MOESM12_ESM.zip › Figure EV4/EV4B/No of junctions (both, multiple values per mouse)_Deletor.pdf]

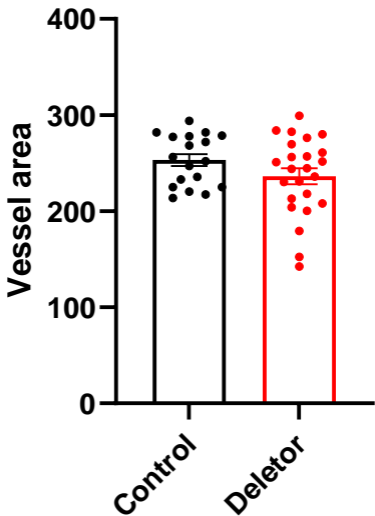

Supplement: Supplementary file 12 — Figure EV4 Source Data [file 44321_2026_438_MOESM12_ESM.zip › Figure EV4/EV4B/Vessel area (both, multiple values per mouse)_Deletor.pdf]

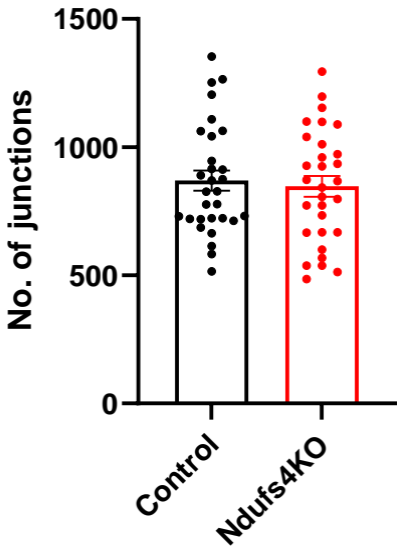

Supplement: Supplementary file 12 — Figure EV4 Source Data [file 44321_2026_438_MOESM12_ESM.zip › Figure EV4/EV4F/No of junctions (both, multiple values per mouse)_Ndufs4KO.pdf]

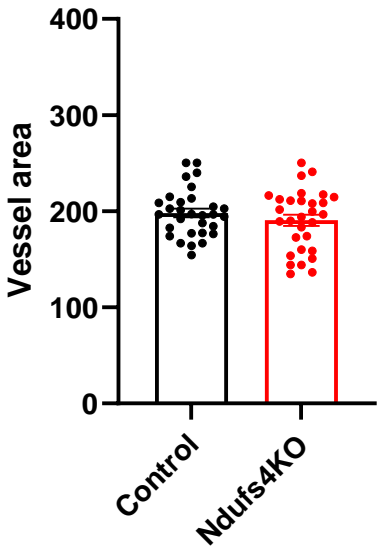

Supplement: Supplementary file 12 — Figure EV4 Source Data [file 44321_2026_438_MOESM12_ESM.zip › Figure EV4/EV4F/Vessel area (both, multiple values per mouse)_Ndufs4KO.pdf]

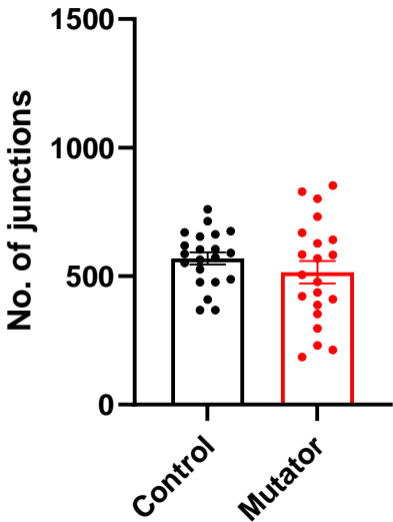

Supplement: Supplementary file 12 — Figure EV4 Source Data [file 44321_2026_438_MOESM12_ESM.zip › Figure EV4/EV4D/No of junctions (both, multiple values per mouse)_Mutator.pdf]

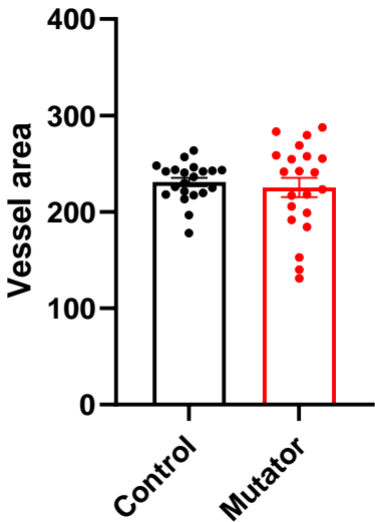

Supplement: Supplementary file 12 — Figure EV4 Source Data [file 44321_2026_438_MOESM12_ESM.zip › Figure EV4/EV4D/Vessel area (both, multiple values per mouse)_Mutator.pdf]

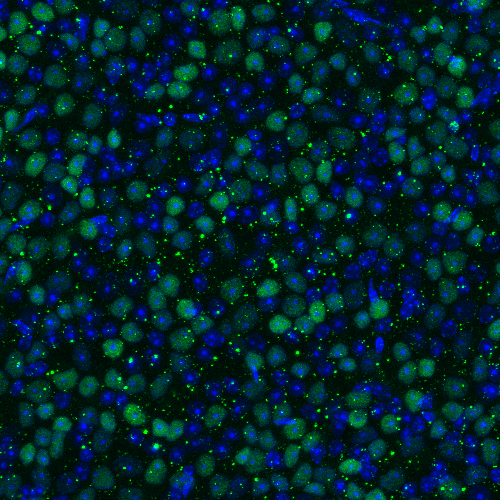

Supplement: Supplementary file 13 — Figure EV6 Source Data [file 44321_2026_438_MOESM13_ESM.zip › Figure EV6/AW6477_Brn3a_20x_3_Maximum intensity projection-Create Image Subset-42_c1+2.tif]

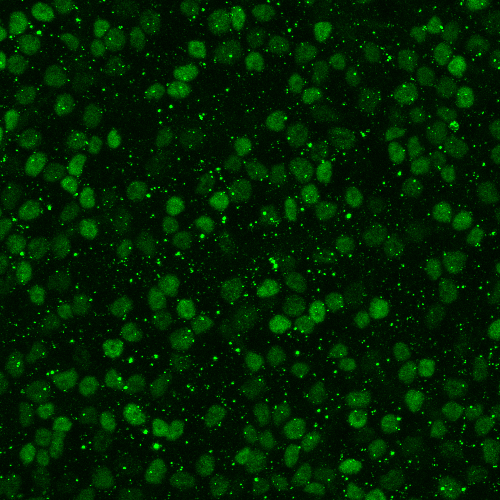

Supplement: Supplementary file 13 — Figure EV6 Source Data [file 44321_2026_438_MOESM13_ESM.zip › Figure EV6/AW6477_Brn3a_20x_3_Maximum intensity projection-Create Image Subset-42_c1.tif]

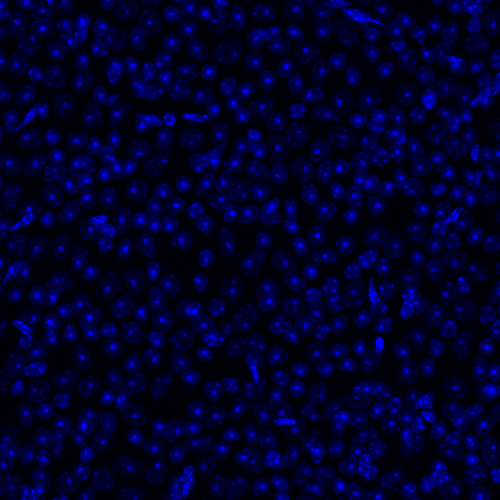

Supplement: Supplementary file 13 — Figure EV6 Source Data [file 44321_2026_438_MOESM13_ESM.zip › Figure EV6/AW6477_Brn3a_20x_3_Maximum intensity projection-Create Image Subset-42_c2.tif]

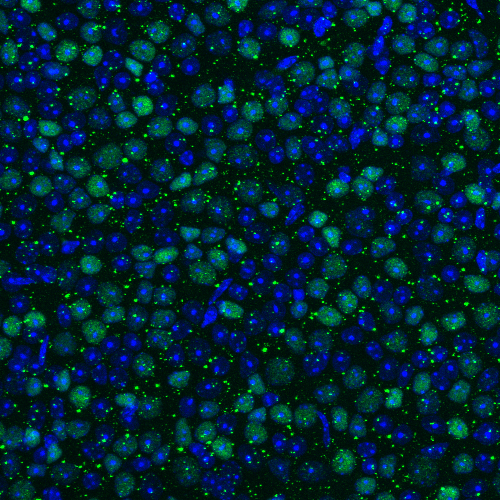

Supplement: Supplementary file 13 — Figure EV6 Source Data [file 44321_2026_438_MOESM13_ESM.zip › Figure EV6/AW6485_Brn3a_20x_3_Maximum intensity projection-Create Image Subset-85_c1+2.tif]

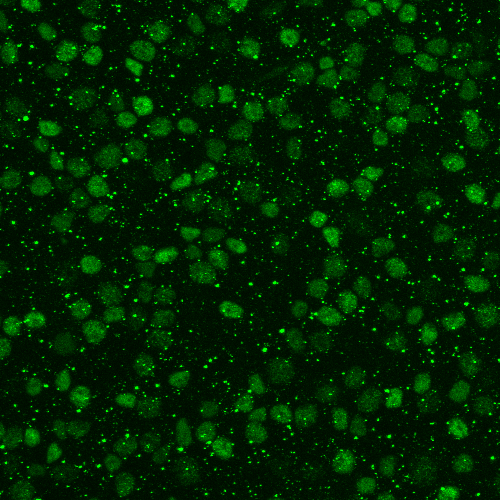

Supplement: Supplementary file 13 — Figure EV6 Source Data [file 44321_2026_438_MOESM13_ESM.zip › Figure EV6/AW6485_Brn3a_20x_3_Maximum intensity projection-Create Image Subset-85_c1.tif]

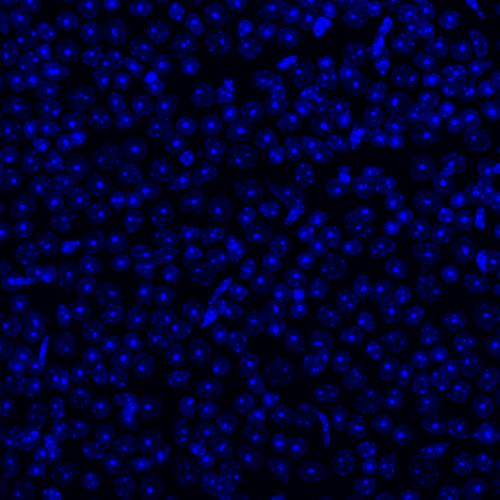

Supplement: Supplementary file 13 — Figure EV6 Source Data [file 44321_2026_438_MOESM13_ESM.zip › Figure EV6/AW6485_Brn3a_20x_3_Maximum intensity projection-Create Image Subset-85_c2.tif]

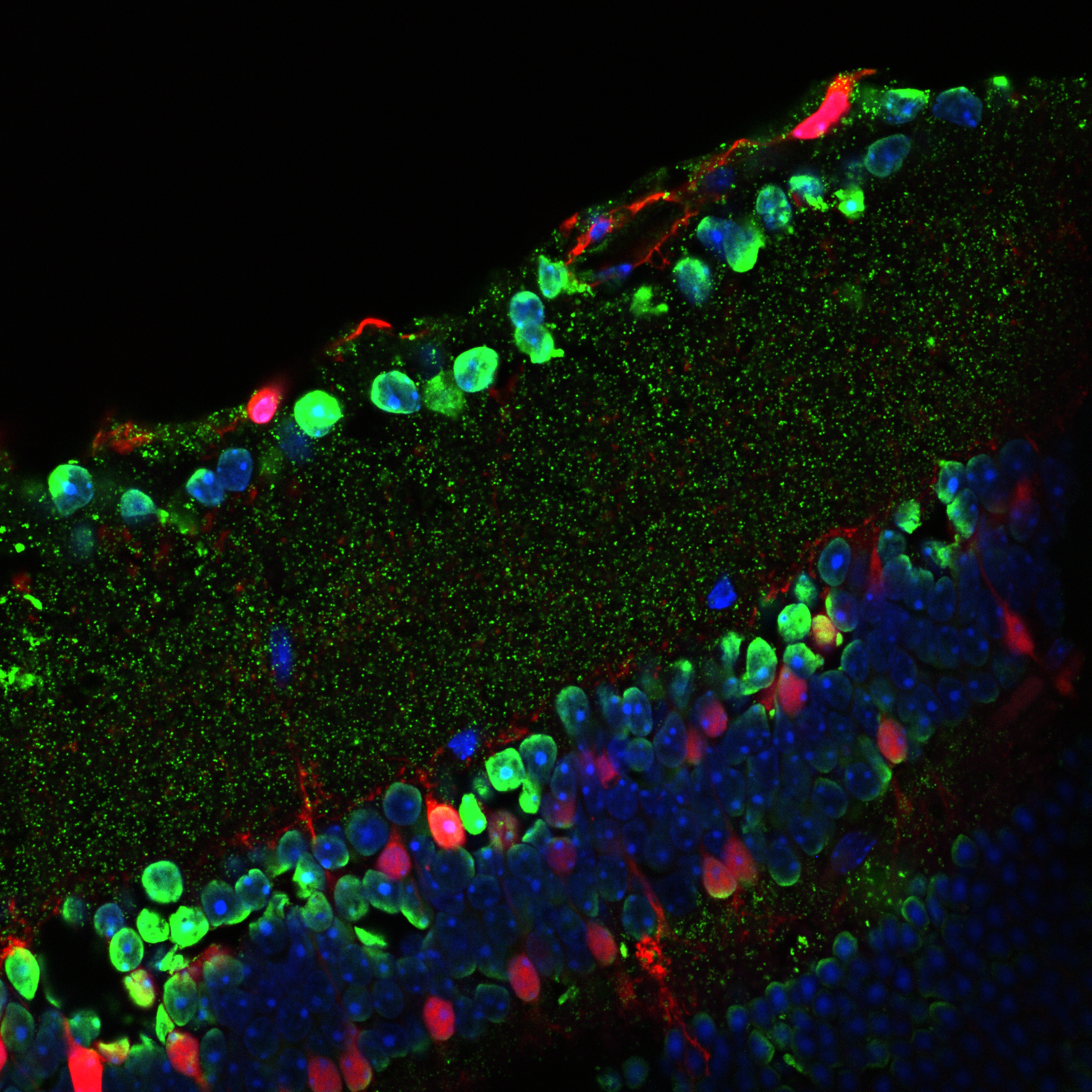

Supplement: Supplementary file 14 — Figure EV7 Source Data [file 44321_2026_438_MOESM14_ESM.zip › Figure EV7/Composite.png]

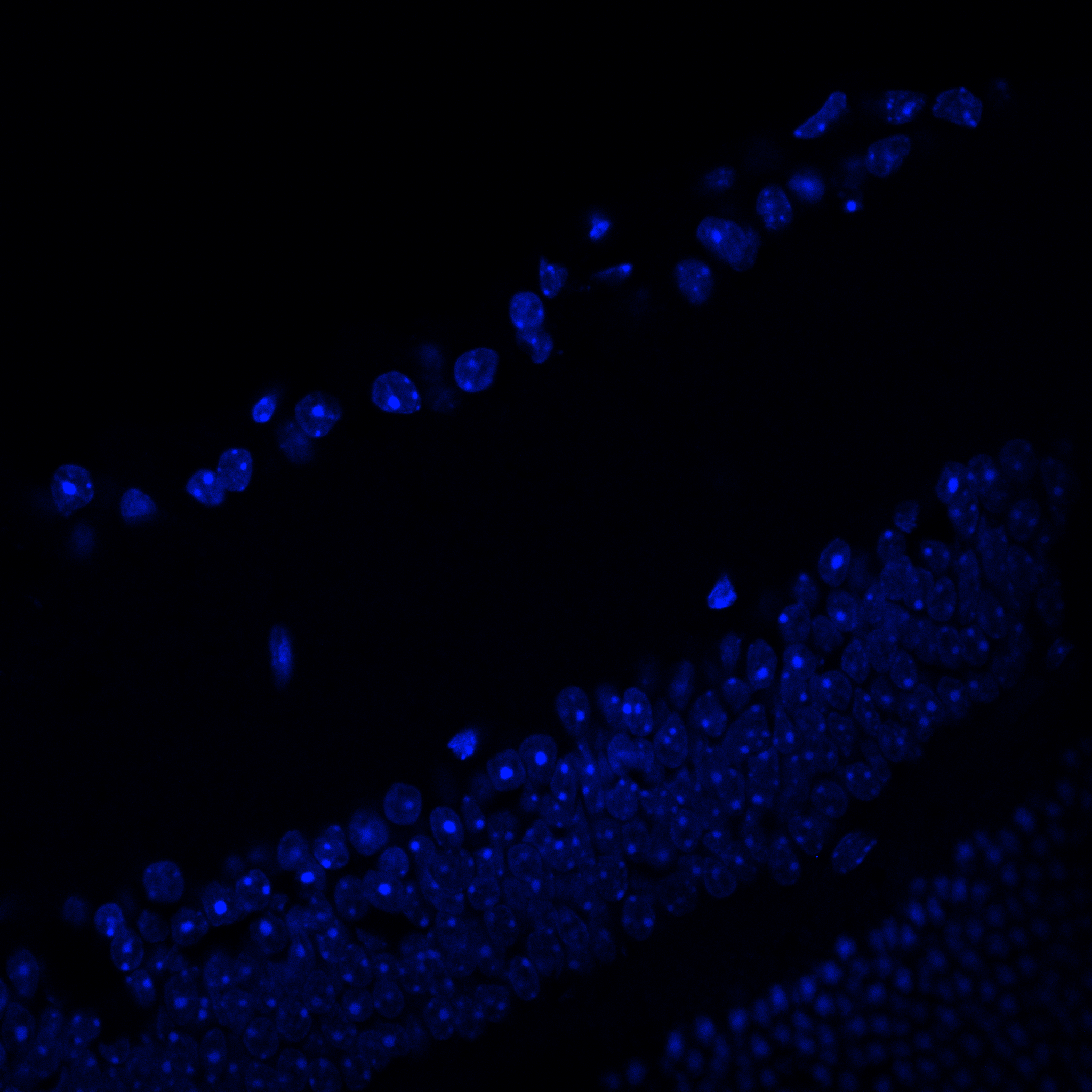

Supplement: Supplementary file 14 — Figure EV7 Source Data [file 44321_2026_438_MOESM14_ESM.zip › Figure EV7/FS023_AW7672_405_DAPI_CF40_Zyla_488_GFP_CF40_Zyla_561_RFP_CF40...61_RFP_CF40_Zyla_Retina60x_mtDNA_tomato.ims Resolution Level 1 Z=15 C=0.png]

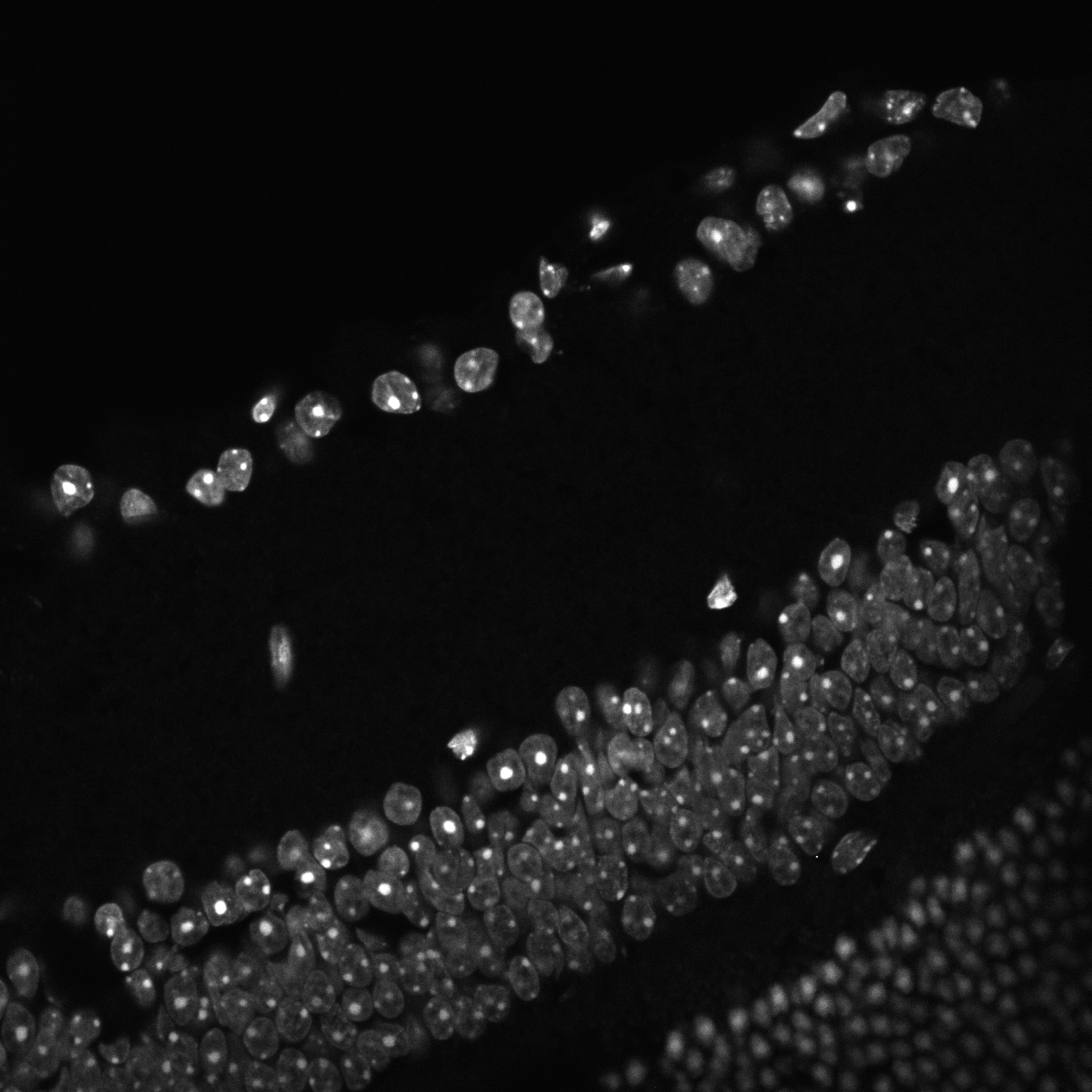

Supplement: Supplementary file 14 — Figure EV7 Source Data [file 44321_2026_438_MOESM14_ESM.zip › Figure EV7/FS023_AW7672_405_DAPI_CF40_Zyla_488_GFP_CF40_Zyla_561_RFP_CF40...61_RFP_CF40_Zyla_Retina60x_mtDNA_tomato.ims Resolution Level 1 Z=15 C=0.tif]

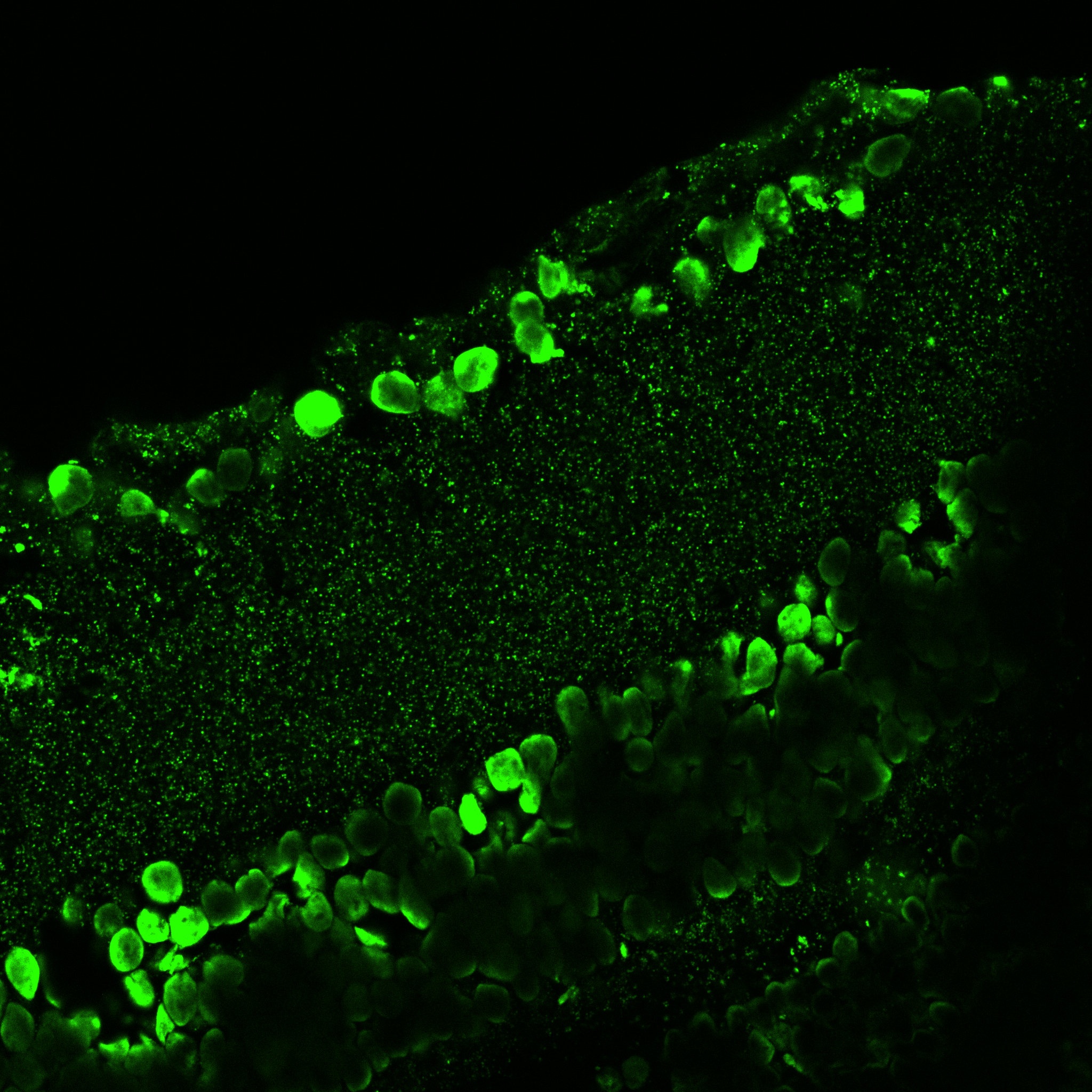

Supplement: Supplementary file 14 — Figure EV7 Source Data [file 44321_2026_438_MOESM14_ESM.zip › Figure EV7/FS023_AW7672_405_DAPI_CF40_Zyla_488_GFP_CF40_Zyla_561_RFP_CF40...61_RFP_CF40_Zyla_Retina60x_mtDNA_tomato.ims Resolution Level 1 Z=15 C=1.png]

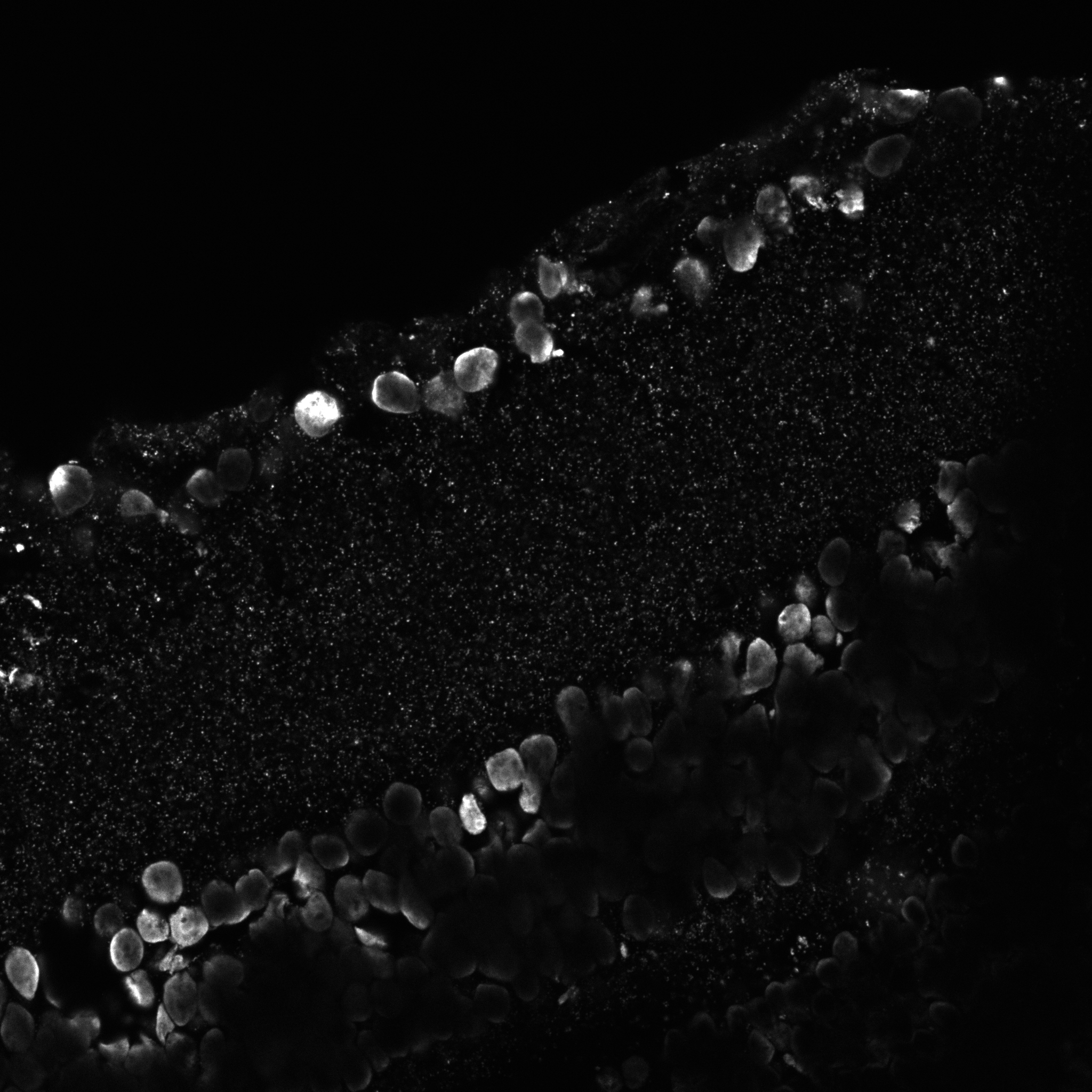

Supplement: Supplementary file 14 — Figure EV7 Source Data [file 44321_2026_438_MOESM14_ESM.zip › Figure EV7/FS023_AW7672_405_DAPI_CF40_Zyla_488_GFP_CF40_Zyla_561_RFP_CF40...61_RFP_CF40_Zyla_Retina60x_mtDNA_tomato.ims Resolution Level 1 Z=15 C=1.tif]

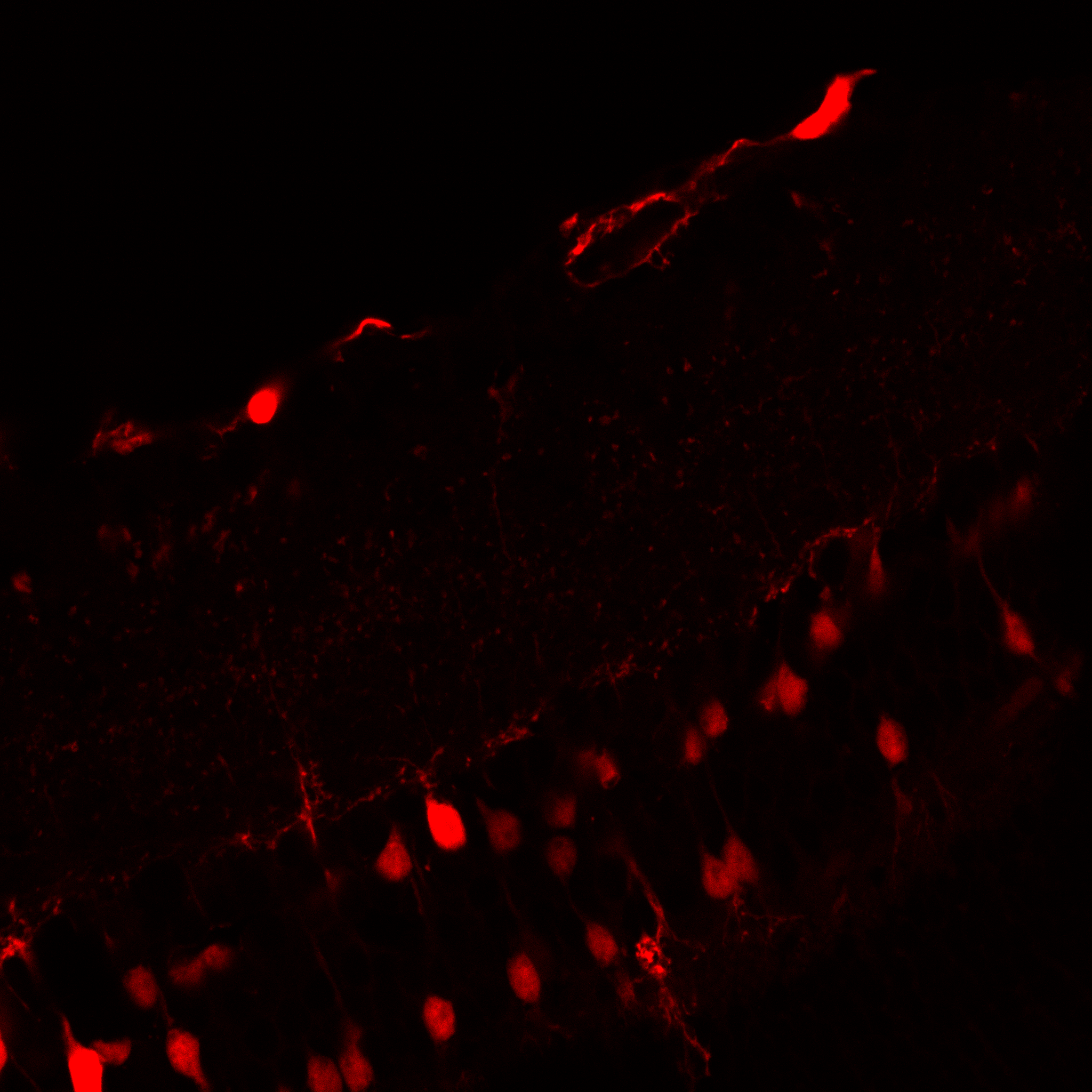

Supplement: Supplementary file 14 — Figure EV7 Source Data [file 44321_2026_438_MOESM14_ESM.zip › Figure EV7/FS023_AW7672_405_DAPI_CF40_Zyla_488_GFP_CF40_Zyla_561_RFP_CF40...61_RFP_CF40_Zyla_Retina60x_mtDNA_tomato.ims Resolution Level 1 Z=15 C=2.png]

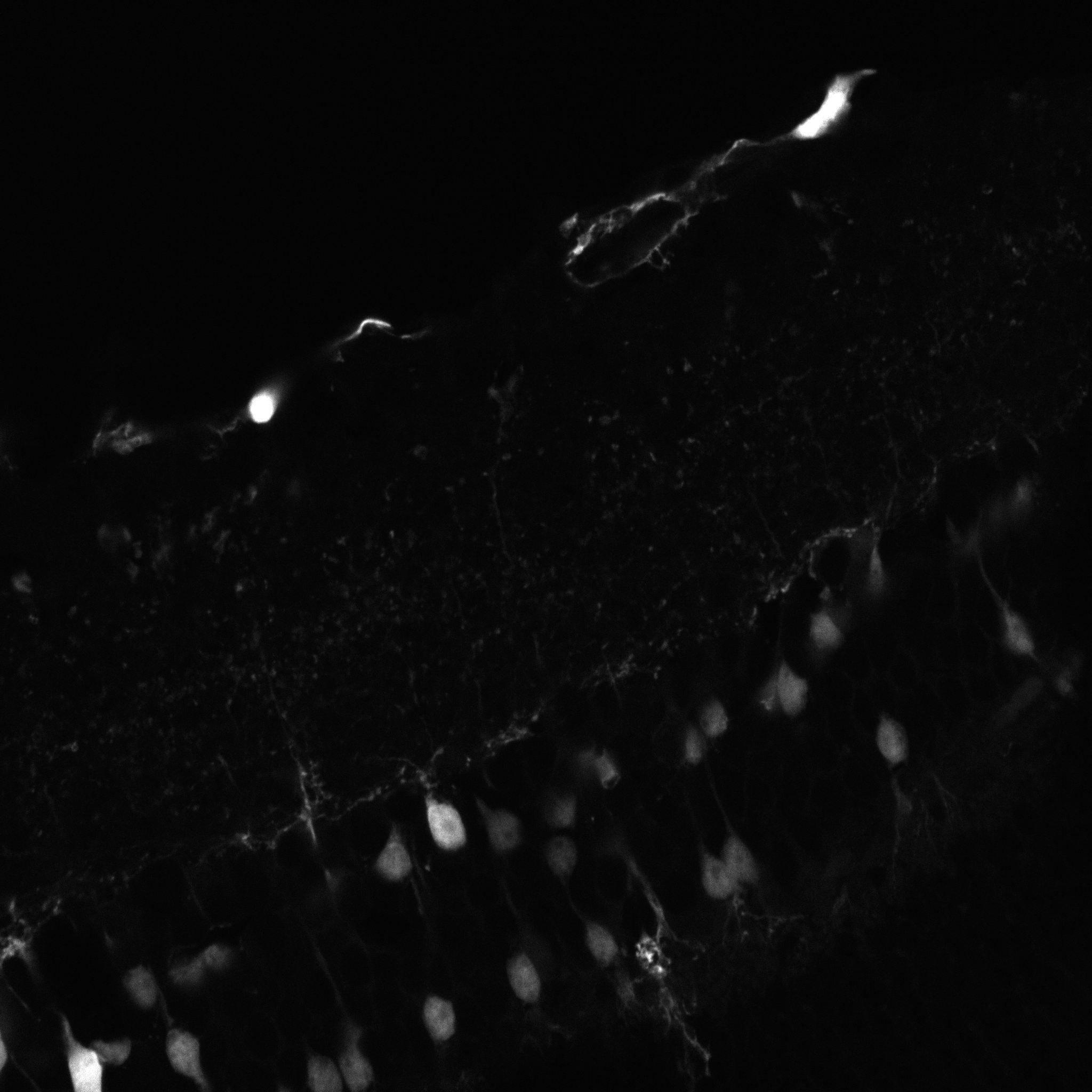

Supplement: Supplementary file 14 — Figure EV7 Source Data [file 44321_2026_438_MOESM14_ESM.zip › Figure EV7/FS023_AW7672_405_DAPI_CF40_Zyla_488_GFP_CF40_Zyla_561_RFP_CF40...61_RFP_CF40_Zyla_Retina60x_mtDNA_tomato.ims Resolution Level 1 Z=15 C=2.tif]

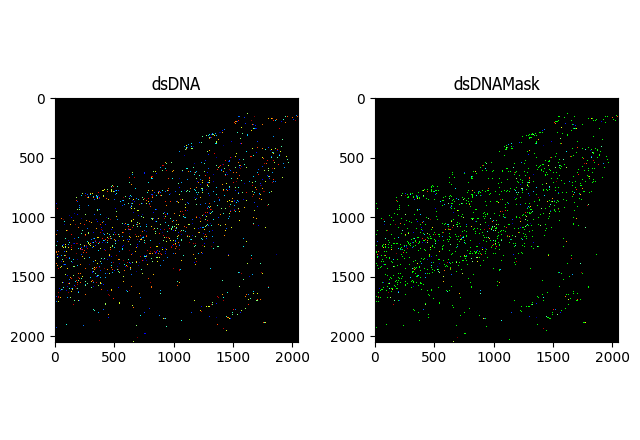

Supplement: Supplementary file 14 — Figure EV7 Source Data [file 44321_2026_438_MOESM14_ESM.zip › Figure EV7/MaskdsDNA.png]

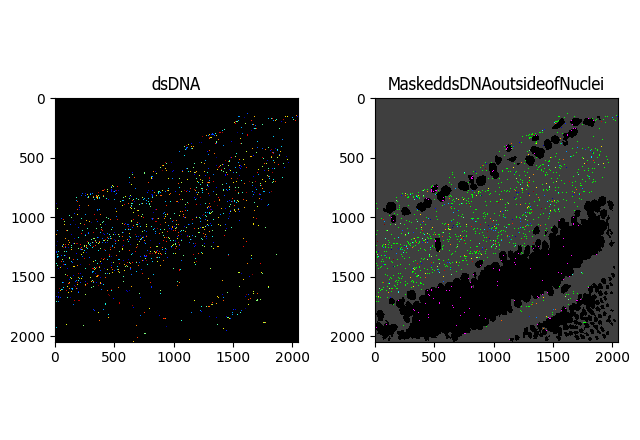

Supplement: Supplementary file 14 — Figure EV7 Source Data [file 44321_2026_438_MOESM14_ESM.zip › Figure EV7/MaskdsDNAoutNuclei.png]

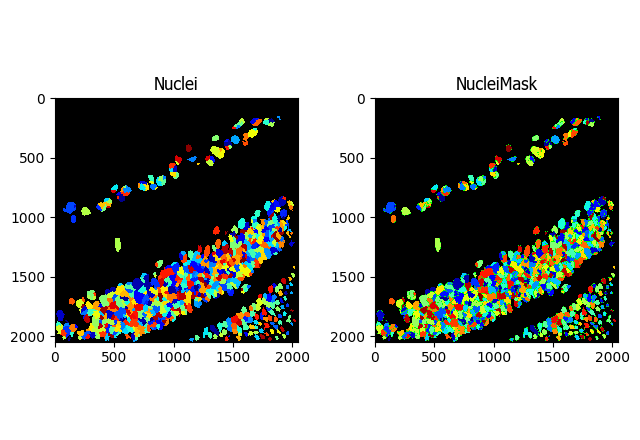

Supplement: Supplementary file 14 — Figure EV7 Source Data [file 44321_2026_438_MOESM14_ESM.zip › Figure EV7/MaskNuclei.png]

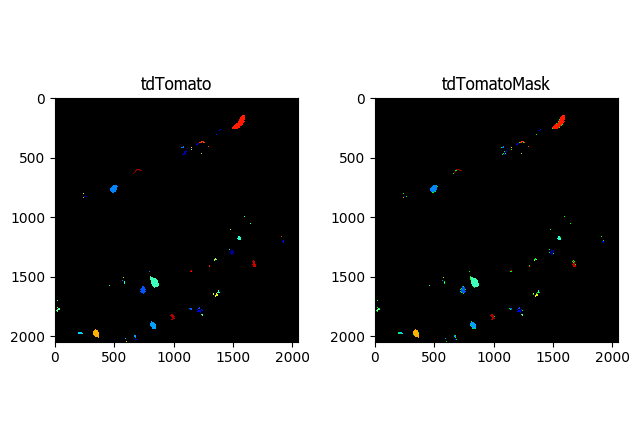

Supplement: Supplementary file 14 — Figure EV7 Source Data [file 44321_2026_438_MOESM14_ESM.zip › Figure EV7/MasktdTomato.png]

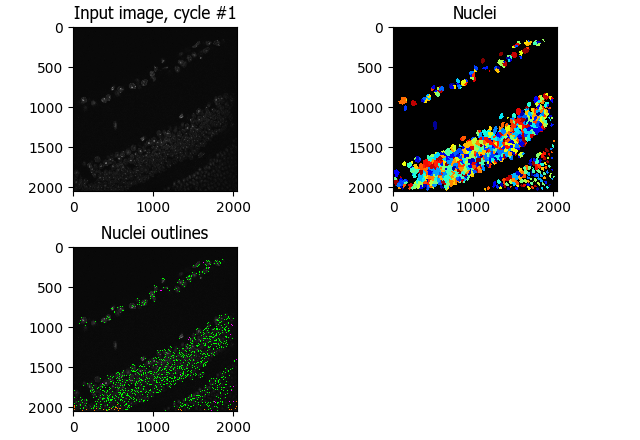

Supplement: Supplementary file 14 — Figure EV7 Source Data [file 44321_2026_438_MOESM14_ESM.zip › Figure EV7/PrimaryDNA.png]

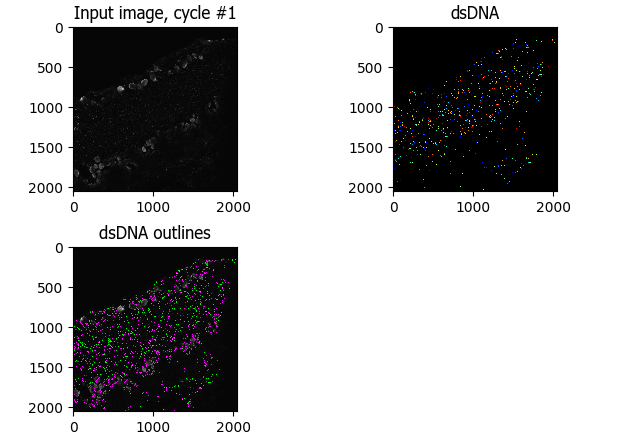

Supplement: Supplementary file 14 — Figure EV7 Source Data [file 44321_2026_438_MOESM14_ESM.zip › Figure EV7/PrimarydsDNA.png]

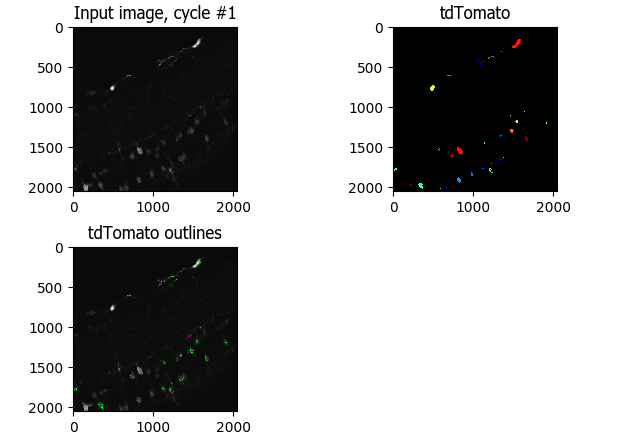

Supplement: Supplementary file 14 — Figure EV7 Source Data [file 44321_2026_438_MOESM14_ESM.zip › Figure EV7/PrimarytdTomato.png]

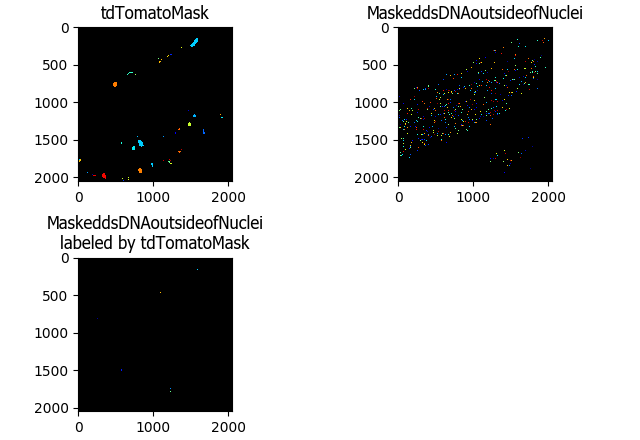

Supplement: Supplementary file 14 — Figure EV7 Source Data [file 44321_2026_438_MOESM14_ESM.zip › Figure EV7/RelateObjects.png]
