# Supplementary material for: Loss of mitochondrial DNA helicase in retinal macroglia drives neovascular retinopathy
Source: EMBO Mol Med. 2026 May 8;18(7):2573–98. doi: 10.1038/s44321-026-00438-0 (PMC13365537; doi:10.1038/s44321-026-00438-0)
Supplement: Supplementary file 15 — Expanded View Figures [file 44321_2026_438_MOESM15_ESM.pdf]

# Expanded View Figures

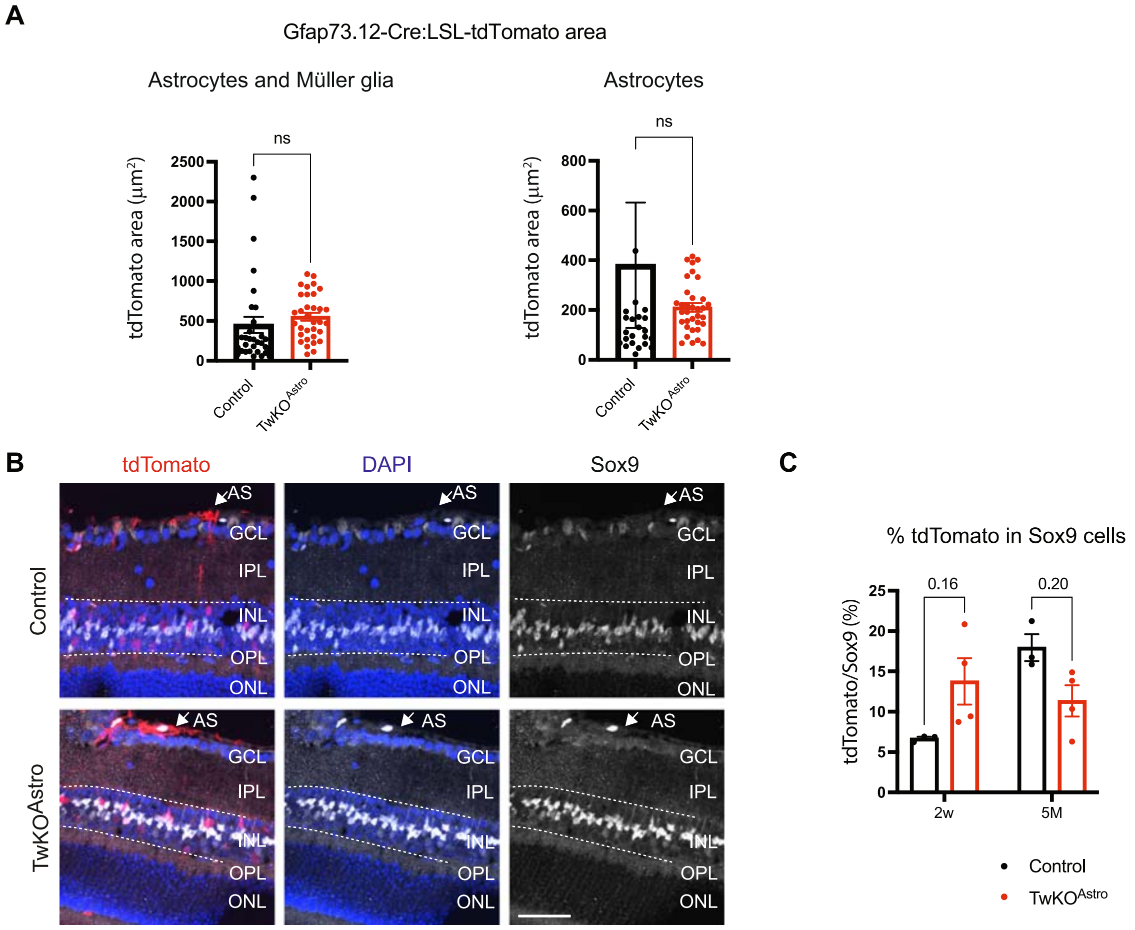


**Figure EV1. Validation of Twinkle inactivation in retinal macroglia of TwKO$^{Astro}$ mice.**

**(A)** Quantification of cells expressing tdTomato under GFAP-promoter in 5-month-old TwKO$^{Astro}$ and control mice (Gfap73.12-Cre:LSL-tdTomato). Left panel: quantification of retina section of 205.8 × 205.8 µm$^2$ image area including AS, GCL, IPL, INL, OPL, and ONL. Right panel: quantification of cropped astrocyte layer 100 × 100 µm$^2$. Six to nine locations in each retina section were imaged. Each dot represents an image. No difference in recombination activity between genotypes ($P = 0.2301$ for the left panel and $P = 0.1692$ for the right panel). Data represent mean ± SEM. *P* values were calculated using two-tailed unpaired *t* test. Statistical significance: $P < 0.05$. Control mice, $n = 3$; TwKO$^{Astro}$ mice, $n = 3$. **(B)** Müller glia, representative image. immunofluorescence with Sox9 antibody, retinal cross-section from 5-month-old mice. The white dotted line indicates the inner nuclear layer (INL) with Sox9-positive Müller glia cells and tdTomato-positive cells. Scale bar: 50 µm. **(C)** Quantification of GFAP-promoter activity in Müller glia; tdTomato/Sox9 ratio per image (2 weeks and 5 M old control $n = 3$, TwKO$^{Astro}$ $n = 4$). Eight to 14 different locations in each retina section were imaged; ×60 objective. Data represent mean ± SEM. *P* values were calculated using two-way ANOVA multiple comparisons. Statistical significance: $P < 0.05$. AS astrocytes, GCL ganglion cell layer, IPL inner plexiform layer, INL inner nuclear layer, OPL outer plexiform layer, ONL outer nuclear layer. Source data are available online for this figure.

 © The Author(s)

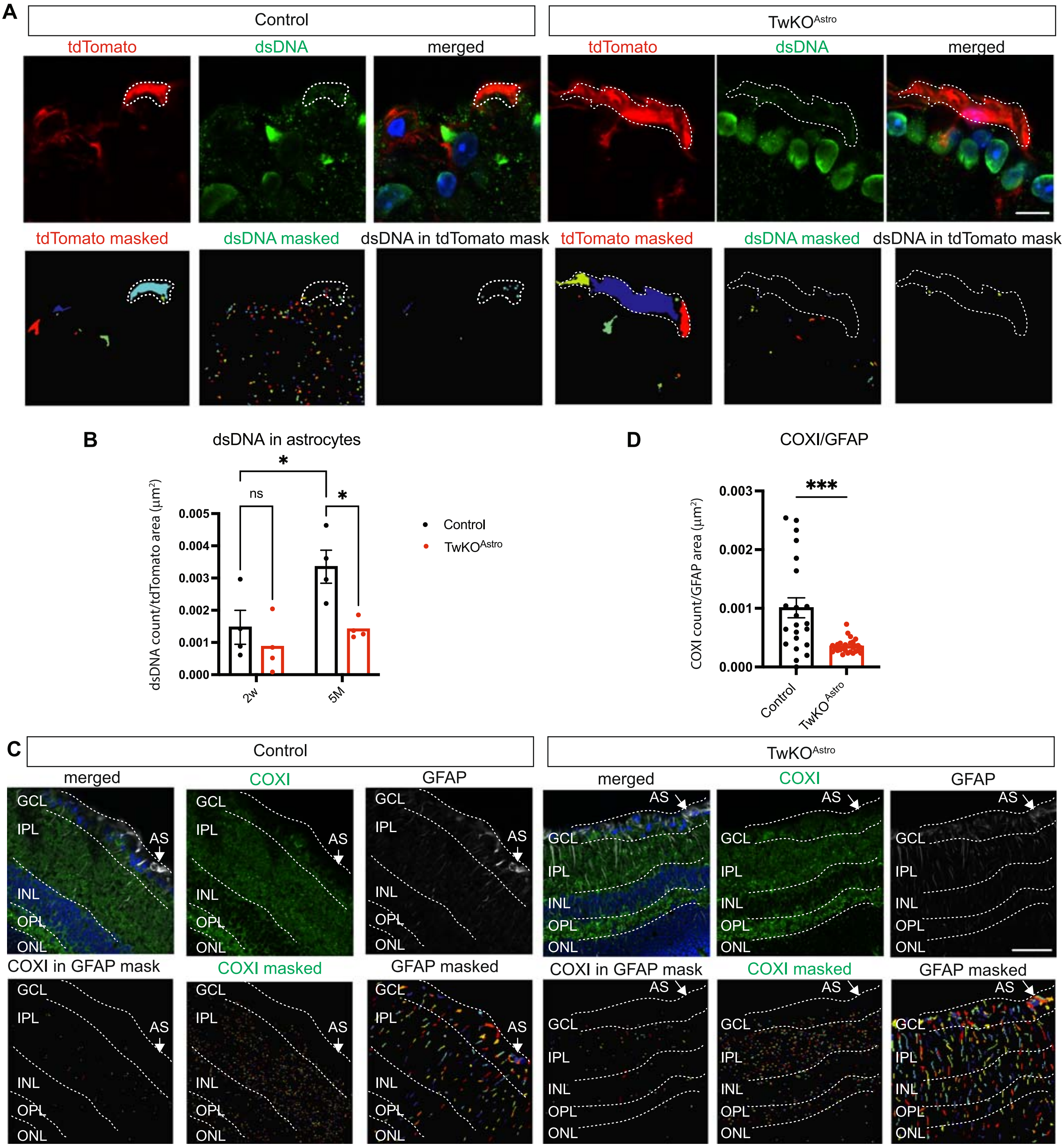
A
Control
TwKOAstro
tdTomato
dsDNA
merged
tdTomato masked
dsDNA masked
dsDNA in tdTomato mask
B
dsDNA in astrocytes
dsDNA count/tdTomato area (µm²)
0.005
0.004
0.003
0.002
0.001
0.000
ns
*
2w
5M
Control
TwKOAstro
D
COXI/GFAP
COXI count/GFAP area (µm²)
0.003
0.002
0.001
0.000
***
Control
TwKOAstro
C
merged
COXI
GFAP
COXI in GFAP mask
COXI masked
GFAP masked
GCL
IPL
INL
OPL
ONL
AS

◀ **Figure EV2. Quantification of mtDNA and its translation product cytochrome c oxidase subunit I (COX-I) in retina.**

(**A**) mtDNA in TwKO$^{Astro}$. Upper panel: Double-stranded DNA (dsDNA) immunofluorescence (green) in retinal cross-section with tdTomato (red). Lower panel: Cell Profiler masked images. Outlined with a white dotted line, examples of tdTomato-positive astrocytes in the vicinity of the ganglion cell layer (green nuclei). Scale bar: 10 μm. (**B**) Quantification of dsDNA signal in tdTomato-positive astrocytes (count/μm$^2$). Control $n = 4$, TwKO$^{Astro}$ $n = 4$. Six to nine locations in each retina section were imaged using a ×60 objective. The Cell Profiler pipeline was designed to quantify dsDNA within the tdTomato-positive cells in each image. Overlapping dsDNA signals within the nuclear (DAPI) signal were omitted. dsDNA count is normalized to the area of tdTomato positive signal area. Each dot in the graph represents an animal. Data represent mean ± SEM. *P* values were calculated using two-way ANOVA multiple comparisons. Statistical significance: Control 2 M vs 5 M; $^*P = 0.041$. 5 M Control vs TwKO$^{Astro}$; $^*P = 0.034$. (**C**) Mitochondrial DNA encoded protein expression: cytochrome c oxidase subunit I (COX-I). Upper panel: COX-I and GFAP immunofluorescence; retina cross-section; Lower panel: Cell Profiler masked images. Control $n = 3$, TwKO$^{Astro}$ $n = 4$. Six to nineteen locations in each retina section were imaged using a ×60 objective. The white dotted line delineates the boundary between retinal layers. Scale bar: 50 μm. (**D**) Quantification of COX-I in GFAP-positive astrocytes. Control $n = 4$, TwKO$^{Astro}$ $n = 4$. Six to nine locations in each retina section were imaged using a ×60 objective. Each dot in the graph represents an image from which all positive COXI/GFAP-positive signals were calculated. Data represent mean ± SEM. *P* values were calculated using two-tailed unpaired *t* test. Statistical significance: $^{***}P = 0.0001$. AS astrocytes (pointed with a white arrow), GCL ganglion cell layer, IPL inner plexiform layer, INL inner nuclear layer, OPL outer plexiform layer, ONL outer nuclear layer. Source data are available online for this figure.

 © The Author(s)

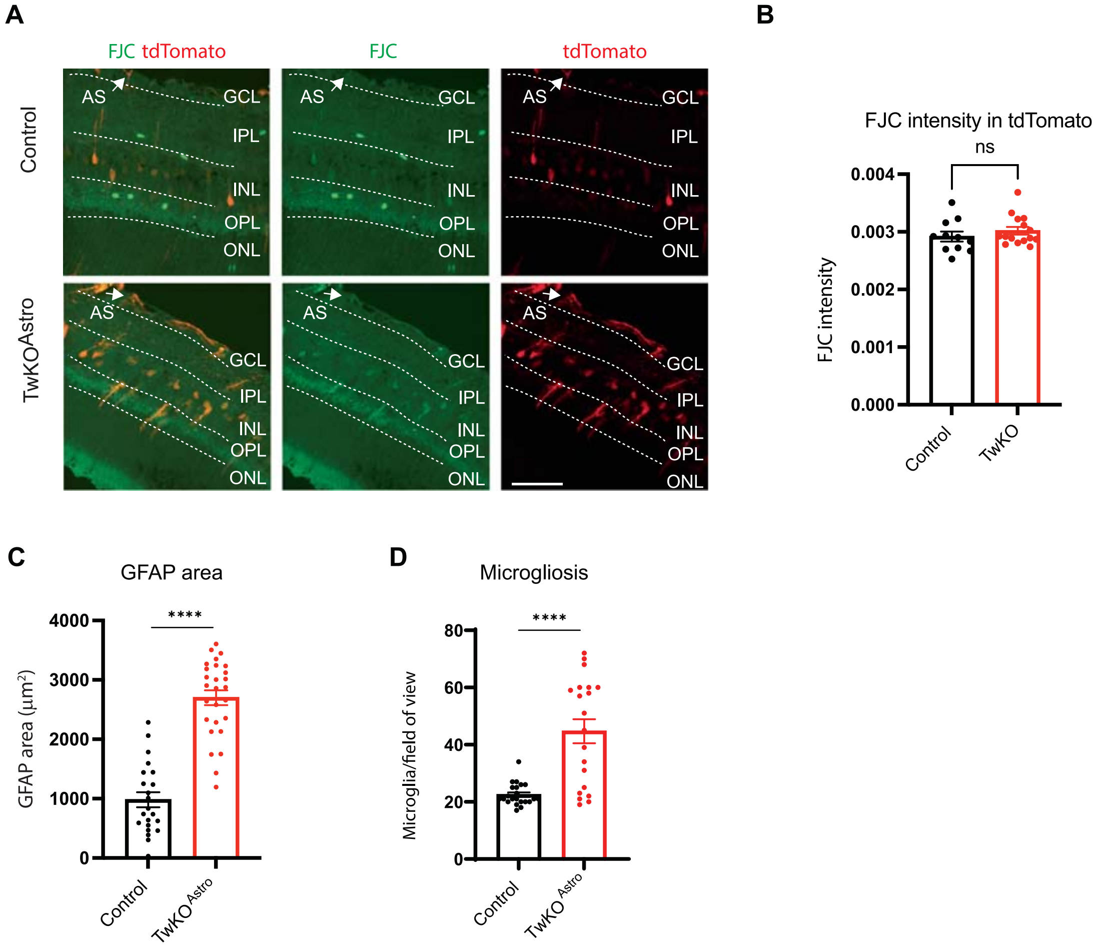


**Figure EV3. Quantification of microglial and GFAP-expressing cells.**

(**A**) Cell death in retina of $TwKO^{Astro}$ and control mice, Fluoro-Jade C (FJC) staining (green); tdTomato (red). White dotted line delineates the boundary between retinal layers. Scale bar: 50 μm. AS astrocytes, GCL ganglion cell layer, IPL inner plexiform layer, INL inner nuclear layer, OPL outer plexiform layer, ONL outer nuclear layer. (**B**) Quantification of (**D**); FJC intensity in cells expressing Gfap73.12-Cre:LS-tdTomato. Data represent mean ± SEM. Four to six locations in each retina section were imaged. Each dot represents an image. Control mice, $n = 3$; TwKOAstro mice, $n = 3$. $P$ values were calculated using two-tailed unpaired $t$ test. Statistical significance: $^*P < 0.05$. (**C**) Macroglial (GFAP area) quantification on retina sections. Control n = 4, $TwKO^{Astro}$ $n = 4$. Six to nine locations in each retina section were imaged for 10 μm z-stack using a ×60 objective. Cell Profiler pipeline was designed to quantify GFAP area for the maximum intensity projection of z-stack in each image. Data represent mean ± SEM. $P$ values were calculated using two-tailed unpaired $t$ test. Statistical significance: $^{****}P = 1.341 \times 10^{-8}$. Each dot in the graph represents each image. (**D**) Microglia in whole-mount retinas; IBA1 staining. Six locations in each retina were imaged using a ×20 objective. The number of cell bodies in each image ("field of view") was counted, with each dot in the graph representing the cell count per field of view. Control $n = 4$. $TwKO^{Astro}$ $n = 4$. Statistical significance: $^{****}P = 3 \times 10^{-6}$. Source data are available online for this figure.

© The Author(s) 

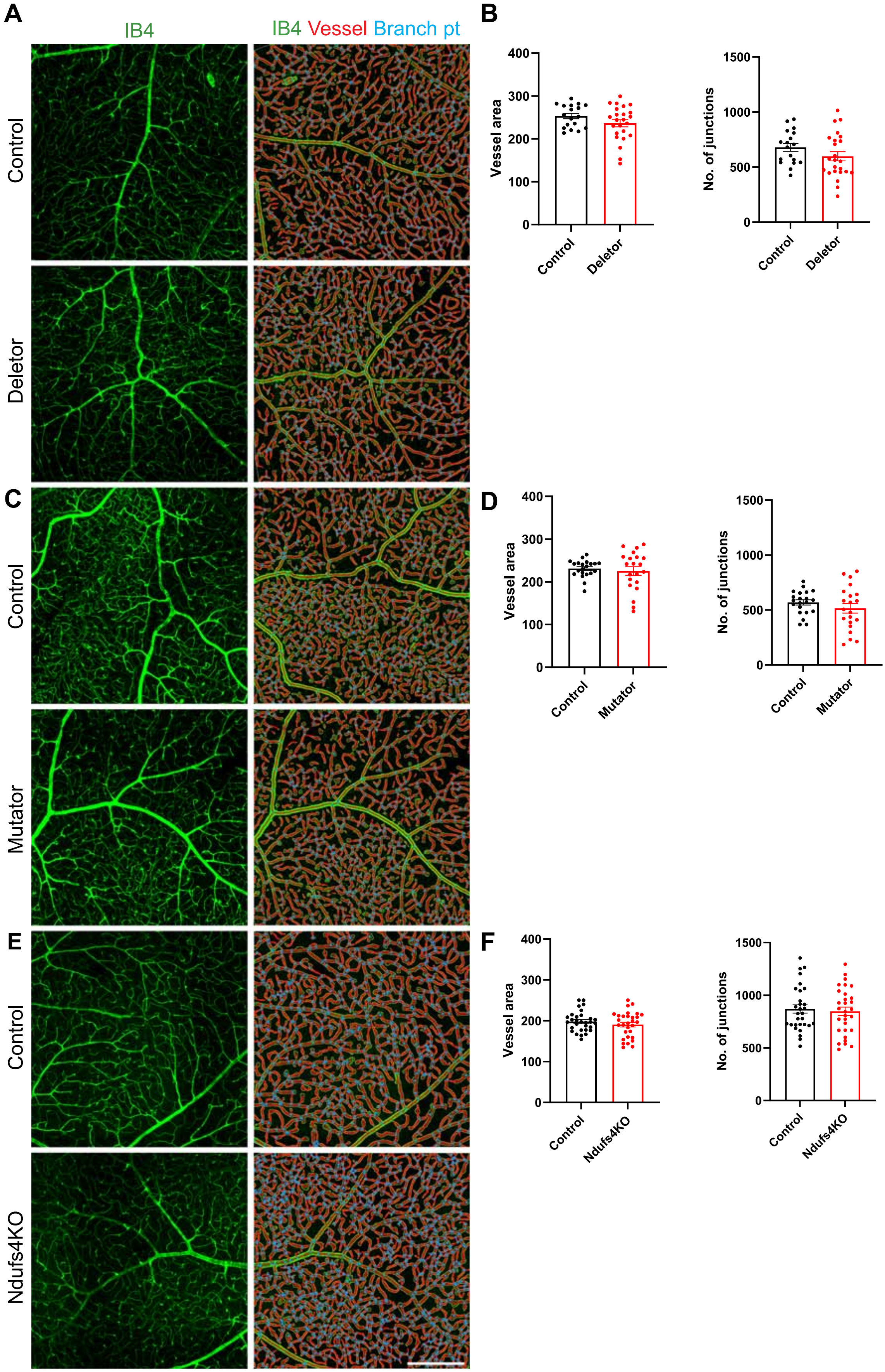
A
IB4
IB4 Vessel Branch pt
Control
Deletor
B
Vessel area
400
300
200
100
0
Control
Deletor
No. of junctions
1500
1000
500
0
Control
Deletor
C
Control
Mutator
D
Vessel area
400
300
200
100
0
Control
Mutator
No. of junctions
1500
1000
500
0
Control
Mutator
E
Control
Ndufs4KO
F
Vessel area
400
300
200
100
0
Control
Ndufs4KO
No. of junctions
1500
1000
500
0
Control
Ndufs4KO

 © The Author(s)

**Figure EV4. Retinal vasculature in different mitochondrial disease models.**

IB4 staining of retinal whole mounts and their corresponding AngioTool quantification. (**A**, **B**) Deletor mice ($n = 8$) and littermate controls ($n = 6$). (**C**, **D**) Mutator mice ($n = 7$) and their littermate controls ($n = 7$). (**E**, **F**) Ndufs4KO ($n = 6$) and their littermate controls ($n = 6$). Data represent mean ± SEM. Each dot represents an individual measurement. *P* values were calculated using unpaired two-tailed parametric *t* test. Scale bar, 200 µm. Source data are available online for this figure.

© The Author(s) 

**A**

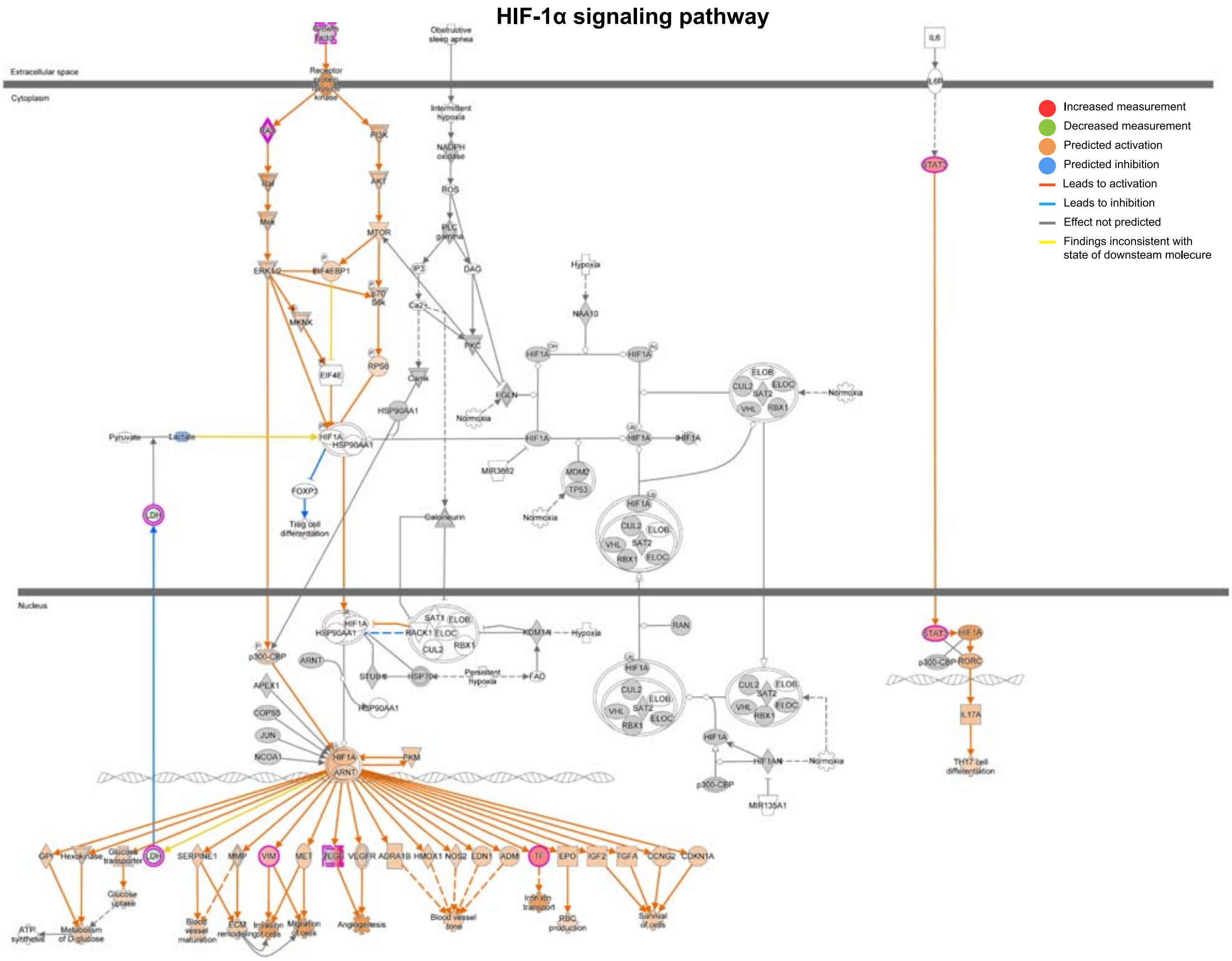


**Figure EV5. HIF-1α pathway.**

(**A**) HIF-1α signaling pathway. Highlighting differentially expressed genes in TwKO$^{Astro}$ retina; Ingenuity Pathway Analysis of RNA sequencing data.


© The Author(s)

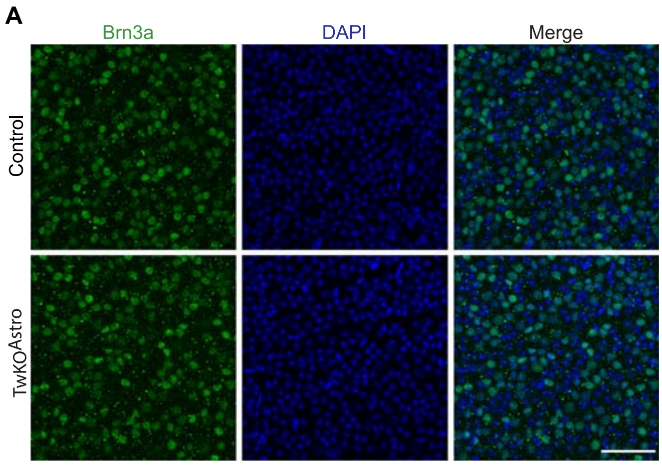


**Figure EV6. Retinal ganglion cells.**

(**A**) Brn3a immunofluorescent staining of whole-mount retinas of $TwKO^{Astro}$ and control mice. Scale bar, 50 µm. Source data are available online for this figure.

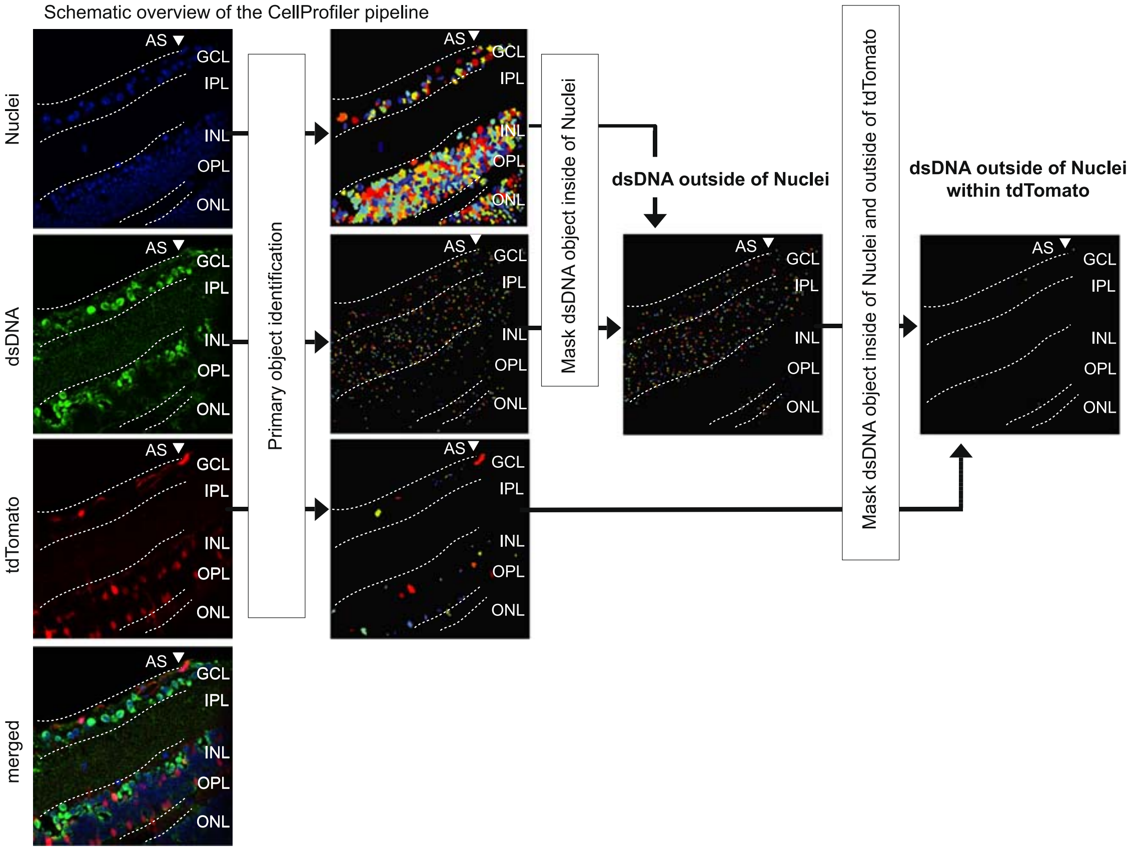


**Figure EV7. Schematic overview of the CellProfiler pipeline for mtDNA quantification within tdTomato-positive retinal astrocytes.**

Each stained object was identified and outlined (nuclei, DAPI, blue; dsDNA, green, both in nucleus and cytoplasm (mtDNA); Tomato indicating cells with cre-activity), the dsDNA signal within the nucleus was masked and removed, and the remaining cytoplasmic dsDNA signal within the tdTomato positive region was quantified. The white dotted line delineates the boundary between retinal layers. Source data are available online for this figure.

 © The Author(s)
