# Supplementary material for: Loss of mitochondrial DNA helicase in retinal macroglia drives neovascular retinopathy
Source: EMBO Mol Med. 2026 May 8;18(7):2573–98. doi: 10.1038/s44321-026-00438-0 (PMC13365537; doi:10.1038/s44321-026-00438-0)
Supplement: Supplementary file 2 — Peer Review File [file 44321_2026_438_MOESM2_ESM.pdf]

EMBO Molecular Medicine

Loss of mitochondrial DNA helicase in retinal macroglia drives neovascular retinopathy

Sofiia Olander, Sinem Karaman, Fumi Suomi, Kevin Aguilar, Aleksandra Zhaivoron, Maiken Nedergaard, Lina Smeds, Jussi Tiihonen, Albert Quintana, Juan Hidalgo, Kari Alitalo, Petri Ala-Laurila, Gulayse Ince-Dunn, and Anu Suomalainen-Wartiovaara

Corresponding authors: Anu Suomalainen-Wartiovaara (anu.wartiovaara@helsinki.fi) , Gulayse Ince-Dunn (gulayse.ince@gmail.com)

Review Timeline:

Submission Date:	7th Jan 25
Editorial Decision:	4th Feb 25
Revision Received:	9th Nov 25
Editorial Decision:	19th Dec 25
Revision Received:	31st Mar 26
Editorial Decision:	1st Apr 26
Revision Received:	15th Apr 26
Accepted:	21st Apr 26

Editor: Lise Roth

Transaction Report:

(Note: With the exception of the correction of typographical or spelling errors that could be a source of ambiguity, letters and reports are not edited. Depending on transfer agreements, referee reports obtained elsewhere may or may not be included in this compilation. Referee reports are anonymous unless the Referee chooses to sign their reports.)

4th Feb 2025

Dear Prof. Suomalainen-Wartiovaara,

Thank you for the submission of your manuscript to EMBO Molecular Medicine. We have now received feedback from the three reviewers who agreed to evaluate your manuscript.

As you will see from the enclosed reports, the referees all acknowledge the interest of the findings, however they also raise partially overlapping major concerns, related - but not limited to - lack of validation of the ko, inadequate image quality and sampling, and overinterpretations.

Based on the nature of the concerns and the amount of time and work that would be needed to address them, considering also that at EMBO Press we encourage a single round of revisions in a limited time frame, we prefer to return the manuscript to you at this point with the decision that we cannot offer to publish it.

However, given the potential interest of the findings, we would welcome a new manuscript on the same topic if you obtained data that would satisfactorily address the referees' concerns.

To be completely clear, however, I would like to stress that if you were to send a new manuscript, and depending on the timeline, it might be treated as a new submission rather than a revision and would be reviewed afresh. If you decide to follow this route, please make sure you nevertheless upload a letter of response to the referees' comments.

I am sorry that I could not bring better news this time and hope that the referee comments are helpful in your continued work in this area.

With kind regards,

Lise Roth

Lise Roth, Ph.D
Senior Editor
EMBO Molecular Medicine

***** Reviewer's comments *****

Referee #1 (Comments on Novelty/Model System for Author):

This is covered in the longer review.

Referee #1 (Remarks for Author):

This manuscript by Olander, et al. examines the consequences of a glial specific deletion of a mitochondrial DNA replicative helicase. They report that Twinkle deletion from glia leads to neovascularization, gliosis, blood retinal barrier breakdown and complement activation. However, no loss of neurons or differences in the coding fidelity of RGCs were found. This is markedly different from brain glia deletion of Twinkle suggesting different mechanism are responsible for maintaining glia homeostasis between the brain and retina.

(1) In Figure 1A, The recombination of GFAP-CRE Tdtomato should be better quantified as this is important. The image provided is of low quality and has some confusing labeling (i.e. Tdtomato even outside the ONL where Muller glia processes do not span). Also, this is only one section presented but there are many more MG than this in the retina, how many recombine? Quantifications should be made of a MG marker like Sox9 or Sox2 in the INL with Tdtomato, if only a small percentage recombine as it looks to be in this picture it could explain some of the variability or penetrance of the phenotype.

(2) Imaging quality and sampling size is also a concern with Fig 1E, strange diagonal orientation with only 1 microglia per field of view is shown. Typically, the standard is to present all retinal images the same, straight, with GCL face down. Wholemounts with microglia morphology and numbers would be a more proper representation, the counts in Fig 1D say they are from wholemounts but the quantification says "per field of view" and if there is only 1 per field of view this is a very small sampling. Both of the

microglia presented here in Fig 1E look ramified and like they do in the homeostatic retina versus the ameboid shape they take in response to retinal pathology.

(3) Is it validated that Twinkle is actually deleted from glia with this approach? The authors show RNA-seq data and it is not part of their downregulated genes. Has this been shown with western blot or other techniques that Twinkle is actually deleted effectively?

(4) Referring to this phenotype as pathological may be a stretch. Taken together, there is a slight increase in vessel area, one more junction per average, and a "gliosis" response which is under quantified but displayed as one more microglia per field of view and one small picture of GFAP induction. What is the consequence of this phenotype on the retina? The structure is fine (Fig 1B), the retina shows no cell death and the physiology seems not impacted. The narrative of the story regarding neovascular retinopathy does not seem to match the results, there is often reference to "wide-spread reactive gliosis", "major structural changes", etc. I think this model is convincingly showing that retinal glia mitochondria are involved with keeping glia in a state that helps maintain vessel homeostasis, beyond that may be overstated.

(5) Would the phenotype of Twinkle knockout not be seen until later ages? It was mentioned by 7 months animal well-being is significantly compromised. I'm confused about the age comparisons made here. The methods say the TwkO astro line was used at 4 and 4.5 months of age versus the control GFAp CRE Td Tomato at 1-2 weeks old? This is not an appropriate comparison for gliosis and neuroprotection experiments if this is the two age groups being compared? The control animals need to be age matched to the knockout especially because Muller glia are not mature at 1-2 weeks.

(6) In the differentially expressed transcripts from the RNA-seq data cone arrestin (arr3) was significantly downregulated. Is there loss of these photoreceptors? The authors look at rod-coding and RGC numbers but are they missing a phenotype? The neuroprotection counts also only using Pou4f1 staining which is not in all RGCs, there may be other vulnerable neuron populations in this model that were not assayed.

Referee #2 (Comments on Novelty/Model System for Author):

The manuscript investigates the effects of a macroglia-specific knockout (KO) of the mitochondrial helicase TWINKLE, revealing microgliosis, macrogliosis, and vascular anomalies. While the topic is compelling and merits further exploration, the study has significant shortcomings. These include insufficient validation of the TWINKLE KO in retinal macroglia, a lack of detailed experimental descriptions, limited integration of state-of-the-art omics approaches, and overinterpretation of the presented data in this specific study. Due to these issues, I cannot support the publication of the manuscript in its current form.
Comments/concerns:
1) In the present study, genetic targeting of the mitochondrial helicase TWINKLE leads to vascular abnormalities. The authors speculate that similar changes also lead to vascular defects observed in diabetic retinopathy (DR), which is a cause of systemic metabolic dysregulation and thus most likely leads to mitochondrial dysfunction, but for quite different reasons than genetic modulation as done in the present study. Please discuss how these two settings can really be compared? Is downregulation of TWINKLE observed in DR?
2) Introduction, page 3: The numbers on cell types seem to refer to mouse - please specify this, since numbers for other species (pig, human) are different (the percentage of glia in the tissue is higher).
3) Introduction - page 3: The description of the retinal cytoarchitecture is quite wrong in many aspects. (i) Retinal astrocytes are more likely to be found in the nerve fiber layer, where the superficial retinal plexus is located, than in the GCL. Surely the typical retinal astrocyte does not interact with the optic nerve (in the optic nerve, of course, there is a population of astrocytes, but it is certainly distinct from those in the retina). Please correct. (ii) The sentences "Apart from the outer photoreceptor layer, Muller glia's extensive cell bodies span the rest of all the retinal layers, interacting with all cell types." is wrong. The outer stem process of Müller cells reaches the outer limiting membrane (in fact, they form it), and Müller cell velate sheaths heavily envelop photoreceptor somata in the ONL. Müller cell microvilli even extend into the subretinal space and interact with photoreceptor segments. Please correct.
4) The authors missed to cite literature in which the role of Müller cells in the context of for example diabetic retinopathy was studied (e.g. work done by the Reichenbach & Bringmann labs - for instance see Krügel et al., Involvement of oxidative stress and mitochondrial dysfunction in the osmotic swelling of retinal glial cells from diabetic rats. Exp Eye Res. 2011 Jan;92(1):87-93. doi: 10.1016/j.exer.2010.11.007. or Pfaller et al., The glucocorticoid receptor as a master regulator of the Müller cell response to diabetic conditions in mice. J Neuroinflammation. 2024 Jan 25;21(1):33. doi: 10.1186/s12974-024-03021-x.) Please go back to the literature and add relevant work.
5) "The diseases can manifest as extraocular muscle weakness, optic atrophy or retinopathy, which typically is pigmentary [11]" - what is meant with pigmentary? This is quite a strange term. Do the authors mean that symptoms are similar to what is observed in retinitis pigmentosa? Please clarify. Moreover - what is a liscencing DNA helicase?
6) Results - Validation of KO in the retina (Fig.1). Typically, Müller cells in the healthy retina express very low levels of GFAP. Consistent with this, it appears (Fig. 1A) that by far not all Müller cells are dtTomato positive (looks like less than 50% of them!).

This in turn means that there is a mosaic of some recombined and many more non-recombined Müller cells. I lack data to show actual numbers for the proportion of the latter. I assume that depending on the recombination efficacy of the GFAP driver in different parts of the CNS, different consequences will be observed, not necessarily due to different mechanisms, but primarily due to the fact that different proportions of macroglia are affected by the KO. This aspect should be much better addressed in order to draw solid conclusions. In this regard, a co-staining with established astrocyte and Müller cell markers are highly recommended.

7) It should be stated also at least once in the results part at which developmental time point the authors think the KO hits the macroglia. Would that before or after vessel ingrowth into the retina? Or the other way around - the neovascularization mention in the first section of the results is indeed neurovascularization or rather a disruption of angiogenesis during development? I miss a systematic approach to address this key question in this manuscript as it seems as only adult mice were studied and no earlier timepoints of retinal development. Generally, please state the age of the mice studied throughout this manuscript accurately (at least in figure legends). Searching for that information, I found the puzzeling info that the Gfap73.12-Cre:LSL-tdTomato used to demonstrate the cell type-specific recombination (Fig. 1A) were 1-2 weeks old (this is before Müller cells actually are fully mature or even formed (1 week old)!) while subsequent analysis on the TWINKLE-Astro mice were performed in 4.5 months old mice. This is very strange. Please provide age-matched data.

8) Fig. 1D,E - why present microglia in a retinal section when they were actually quantified in flatmount preparations. This should be streamlined. Did the authors discriminate between in specific retinal layers (e.g. OPL versus inner retina) to see a trend as to where the cells would accumulated due to the tissue stress?

9) Does KO of TWINKLE lead to loss of macroglia? Would be important to check this. This would explain some of the observed tissue responses also observed when Müller cells were ablated (Shen et al., Conditional Müllercell ablation causes independent neuronal and vascular pathologies in a novel transgenic model. J Neurosci. 2012 Nov 7;32(45):15715-27. doi: 10.1523/JNEUROSCI.2841-12.2012; Shen et al., Effect of glucocorticoids on neuronal and vascular pathology in a transgenic model of selective Müller cell ablation. Glia. 2014 Jul;62(7):1110-24. doi: 10.1002/glia.22666.)

10) Besides - even though this mouse model is published, the authors should demonstrate a direct proof that TWINKLE is indeed knocked out in Müller cells or retinal astrocytes. I miss such kind of data.

11) Page 5 - the statement about GFAP is wrong or at least misleading. Retinal astrocytes of the healthy retina express high levels of GFAP, while Müller cells don't (see above). Please rephrase.

12) Microgliosis is stated on page 5 without specifying the readout parameter to which this statement refers (I assume simple cell counts?). Staining for microglial activation markers (e.g. TSPO or CD68) would be good additional indications.

13) Figure 2. There is no explanation of what the dots in the bar graph represent. Does one represent the result of a single mouse? So the number of biological replicates is <20?

14) It is unclear how the data about the function of the paravascular clearance system in the brain fit into this paper as no comparable data for the retina - the main focus of the present work - are provided. They can be removed for the sake of streamling this work.

15) The additional mouse models (Deletor, Mutator, Ndufs4 knockout mice) used for comparison are not well described - please provide more details here - the reader does not want to do a literature search to get this information. E.g. if they are cell type specific, if they are conditional - when and in which cell type effects can be expected... Otherwise it is very difficult to draw conclusions about the results of the respective experiments.

16) It is stated that in the Deletor mouse "transgenic overexpression of a dominant mutation in Twinkle, leading to mtDNA deletion accumulation" - what does it mean? That mtDNA is deleted or accumulated?

17) It is stated that "Importantly, all the tested models, including TwKOAstro, share the downstream consequence of respiratory chain deficiency." This is a very bold statement not supported by data in the manuscript (and potentially also not by existing literature). Whether this is indeed the case needs to be demonstrated for the retina and the specific cell types therein. Extrapolating this from the results of other cell types/tissues that may have been studied in these different mouse models is not a good practice, as the effects of the genetic manipulations may certainly be quite different depending on the cell type/tissue and their need/expression profile of interactors of TWINKLE.

18) Performing bulk RNA-seq is not state of the art if the interest is on a cell population that is a clear minority in the tissue (as also stated by the authors as limitation of the study). The authors tried to overcome this limitation by checking top DEGs for cell type-specific expression in a published scRNA-seq data set, but for this analysis, even though top DEGs clearly indicate a key role of microglia (interferon, interleukins, C1q) where not included in the analysis. This is a major flaw. Please add this key cell type if it comes to understand tissue inflammation.

19) GSE60361 refers to a study that states "Single-cell RNA-seq of mouse cerebral cortex" and not retina. Please double check and correct. The cited reference refers to the old Makosco study from 2015 - this was run on P15 retina which again is not mature yet. There much better mouse retinal scRNA-seq studies out (even from diabetes models; Van Hove et al., Single-cell transcriptome analysis of the Akimba mouse retina reveals cell-type-specific insights into the pathobiology of diabetic retinopathy. Diabetologia. 2020 Oct;63(10):2235-2248. doi: 10.1007/s00125-020-05218-0; Niu et al., Pathogenesis Study Based on High-Throughput Single-Cell Sequencing Analysis Reveals Novel Transcriptional Landscape and Heterogeneity of Retinal Cells in Type 2 Diabetic Mice. Diabetes. 2021 May;70(5):1185-1197. doi: 10.2337/db20-0839) that would allow better conclusions on expression profiles of retinal cell types in adult retina and retinae with potential neovascularization. For complement expression in retinal cell types, you may also want to check on Pauly et al., 2019 (Cell-Type-Specific Complement Expression in the Healthy and Diseased Retina. Cell Rep. 2019 Nov 26;29(9):2835-2848.e4. doi: 10.1016/j.celrep.2019.10.084.)

20) The authors did not try to validate any of the candidates they pulled out from their somewhat outdated bulkRNA-seq approach - at least this should have been done.

21) Fig. 6G - please provide an exemplary Brn3 image for both genotypes. The figure legend should provide the information

whether cells were counted in flatmounts or sections. Generally, the level of neuronal loss should be better characterized also checking on cells of the inner nuclear layer.
22) Given that the hypothesis is that mitochondria are effected and dysfunctional I am supprised that the manuscript does not provide any data on the mitochondrial fitness of Müller cells/astrocytes in their model (as they also do not prove the actual knockout of TWINKLE - see my comment above). This is worrisome and could/should have been done.
Given the many issues I do have with the results part of the manuscript, I keep my feedback very short about the discussion.
23) The author spent quite a big part of their discussion on DR which brings me back to my concern why they took the P15 scRNA-seq data set from Makosco et al to validate their bulk RNA-seq data and not one of the multiple scRNA-seq data sets from DR mouse models (see above). In its current form, to elaborate that much on DR in the discussion does not make to much sense.
24) Similar effects as described in the TWINKLE Astro model were observed in a model of Müller cell ablation, but the latter is not even mentioned (Shen et al., Conditional Müllercell ablation causes independent neuronal and vascular pathologies in a novel transgenic model. J Neurosci. 2012 Nov 7;32(45):15715-27. doi: 10.1523/JNEUROSCI.2841-12.2012; Shen et al., Effect of glucocorticoids on neuronal and vascular pathology in a transgenic model of selective Müller cell ablation. Glia. 2014 Jul;62(7):1110-24. doi: 10.1002/glia.22666.) Please add and properly discuss.
25) Closing my review with highlighting why I have issues with the interpretation of the data in this study citing the first sentence of the final conclusion of the manuscript "In conclusion, our data highlight the role of mitochondrial metabolism and macroglia in regulating retinal vascular homeostasis and their intriguing potential roles in neovascular retinopathy of diabetic type". There is any data in the manuscript that gives insight into the mitochondrial metabolism of macroglia!

Minor comments:
1) It would be nice if the Müller cells were really written with an "ü" and not spelled "Muller cells".
2) Labeling of the retinal layers in all figures is highly recommended.

Referee #3 (Comments on Novelty/Model System for Author):

The technical quality of the retinal sections and microglia analyses are limited. As detailed in my reviewer comments below, up to date histology and quantitative morphometry should be used in a revised version.

Referee #3 (Remarks for Author):

This paper reports that cell specific deletion of Twinkle in retinal Müller cells and astrocytes causes (aberrant) retinal neovascularization. These findings are interesting and provide a new mechanism and target pathway (mitochondria) for retinal neo-angiogenesis. The authors compared Twinkle-deficient mice with several other mitochondrial mutant mice (Deletor, Mutator, NDUFS4) and showed specific effects only for this gene knockout.

I find the paper really interesting, but my enthusiasm is very limited because the manuscript has several text problems and the technical quality of the retinal histology analyses are partially of poor quality.

Major points of criticism:

1. In general, I would recommend to change the figure subpanel labelling from Figures 1 and 2 as it is confusing. Here subpanels should be labelled from left to right in the appropriate order. For example, in Figure 1 B is followed by E and in Figure 2 A is followed by G.

2. The authors should realign the images from subpanel A, C and E that the retina is correctly positioned with GCL facing down (or up). Also, the retinal layers should be labelled accordingly.

3. Figure 1C: Please indicate the layer and the area from which these images were taken. I assume its GCL. In addition, the authors should do a combination of glutamine synthetase (GS) and GFAP staining to assess Müller cell reactivity. A quantification of these GFAP stress fibers would also be helpful.

4. Figure 1D: Please define what field of view means. Precise information must be provided here. Also here, the authors cannot conclude from these Iba1-stained cryosections that microglia are activated. This is an overstatement. Here, only numbers of microglia per field of view were counted. It is also not clear if microglia were counted in all layers or just IPL or OPL? Cryosections are normally used to analyze the localization of microglia cells. The authors need to complement this analysis with retinal whole mounts stained with Iba1 in order to assess retinal morphology at least in OPL and IPL and also conduct morphometric analysis such as analysis of ramification index, total and spanned area, number of branches, junctions and endpoints.

5. The authors conclude that there are no differences in retinal structure and tissue organization of TwKOAstro and control mice based on retinal cryosections. Here a panorama view from retinas of both mouse lines would be helpful to show the overall

retinal structure. Also, OCT scans and analysis of retinal thickness would be helpful and document the findings.

6. Methods: The authors should indicate for the animal experiments whether mice from both sexes were used and include a statement about potential sex differences.

7. Figure 2 B and D: Here the authors normalized the values to the control mice. However, it would be more helpful if the absolute values would be provided.

8. Analysis of fibrinogen is based hon fluorescent intensity. I would recommend to perform Western Blots or ELISAs to validate the strong reduction of fibrinogen in the retina.

9. Classical complement activation as well as the pro-angiogenic factors identified in transcriptomics should be verified also via ELISAs.

10. The authors should show the wholemount images that were used to analyze Brn3a-positive cells (e.g. in suppl. figures)

Minor points of criticism:
1. The title is completely misleading as the cell-specific deletion was not done in microglia but macroglia.
2. Most figures and figure legends lack n-numbers and specification of analysed sample (e.g. eye. resction, cell etc.)
3. Figure 2 subpanel E: Please make the appearance of statistically significant differences consistent throughout the manuscript.
4. Figure 4: Information about the scale bar is missing.
5. Muller glia' needs to be 'Müller glia'.

As a service to authors, EMBO provides authors with the possibility to transfer a manuscript that one journal cannot offer to publish to another EMBO publication. The full manuscript and if applicable, reviewers reports are automatically sent to the receiving journal to allow for fast handling and a prompt decision on your manuscript. For more details of this service, and to transfer your manuscript to another EMBO title please click on Link Not Available

Please do not share this URL as it will give anyone who clicks it access to your account.

THE AUTHORS' RESPONSES TO THE REVIEWER COMMENTS:

We would like to take the opportunity to thank all the three Reviewers for their favorable and constructive comments, which improved the paper. Here we have addressed all their concerns.

Reviewer Comments:

Reviewer #1: This manuscript by Olander, et al. examines the consequences of a glial specific deletion of a mitochondrial DNA replicative helicase. They report that Twinkle deletion from glia leads to neovascularization, gliosis, blood retinal barrier breakdown and complement activation. However, no loss of neurons or differences in the coding fidelity of RGCs were found. This is markedly different from brain glia deletion of Twinkle suggesting different mechanism are responsible for maintaining glia homeostasis between the brain and retina.

1) In Figure 1A, The recombination of GFAP-CRE Tdtomato should be better quantified as this is important. The image provided is of low quality and has some confusing labeling (i.e. Tdtomato even outside the ONL where Muller glia processes do not span). Also, this is only one section presented but there are many more MG than this in the retina, how many recombine? Quantifications should be made of a MG marker like Sox9 or Sox2 in the INL with Tdtomato, if only a small percentage recombine as it looks to be in this picture it could explain some of the variability or penetrance of the phenotype.

Au: Thank you. As suggested by the Reviewer, we quantified the fraction of Müller glia which had undergone CRE-mediated recombination by crossing TwKOAstro mice to ROSA26-CAG-TdTomato reporter line and quantifying TdTomato signal gated on Sox9+ cells. Our analysis indicated that TdTomato reporter was active in approximately 6-18% of Müller glia. ***Representative images and quantification are now added to Fig. 1EV.*** *These findings are incorporated into the main text p. 5 (…recombination efficiency of less than 20%...). We also acknowledge that there is some TdTomato-positive staining outside of the ONL, in layers not spanned by MG. This extra-ONL signal that faintly is found outside the ONL, is in layers not spanned by Müller glia, mostly within the photoreceptor layer and does not overlap with DAPI-positive cell bodies. While we do not know the exact source of this signal, given that it is faint and distant to the three vascular plexi, we did not focus on its characterization.*

2) Imaging quality and sampling size is also a concern with Fig 1E, strange diagonal orientation with only 1 microglia per field of view is shown. Typically, the standard is to present all retinal images the same, straight, with GCL face down. Wholemounts with microglia morphology and numbers would be a more proper representation, the counts in Fig 1D say they are from wholemounts but the quantification says "per field of view" and if there is only 1 per field of view this is a very small sampling. Both of the microglia presented here in Fig 1E look ramified and like they do in the homeostatic retina versus the ameboid shape they take in response to retinal pathology.

Au: The figures were submitted alongside the manuscript text in a Word file, which may account for the lower image quality observed by the reviewers. All immunofluorescence images included in the

manuscript were acquired using a confocal microscope (LSM780 and ANDOR Dragonfly 505) at a resolution of 1028×1028 or higher. ***We changed the orientation of the retinal images in Fig 1, as requested by the reviewer and marked the retinal layers in all the figures.*** *We quantified the microglia from wholemount images. Six random locations in each retina were imaged, using a 20x objective on the confocal microscope, with IBA1 staining to visualize the cells. The number of cell bodies in each image was counted, which is referred to as "per field of view." The average cell count per retina was then calculated and used for statistical analysis (Figure EV4 A). To assess the morphology of the microglia cells, retinas stained with* ***CD68 and IBA1 antibodies were imaged using a 63x oil objective (NA 1.4 for Figure1 D) and 40x water objective (NA 1.15 for Figure1 E) on the confocal microscope from section (Figure1 D) and whole mount tissue (Figure1 E) and representative images and quantification are shown in Fig. 1D-F****. We have now updated the figure legend. Our findings of increased IBA1 area and perimeter, as well as CD68 intensity in IBA1-gated regions are consistent with a hyper-ramified state.* ***We have updated the wording of this section accordingly in the Results p.5 and updated the figure 1 and figure legend.***

3) Is it validated that Twinkle is actually deleted from glia with this approach? The authors show RNA-seq data and it is not part of their downregulated genes. Has this been shown with western blot or other techniques that Twinkle is actually deleted effectively?The RNAseq data was bulk-sequencing from full retina, and because of the high cellularity of the tissue and low amount of targeted cells (0.5% astrocytes; <5% Mueller cells, of the latter only a fraction was targeted), it is no wonder that Twinkle, typically expressed in low amount compared to other mtDNA replisome proteins, is not detected.

Au: Thank you. While detailed validation of the TwKO^{Astro} *mice was previously done for cerebral cortex astrocytes of these mice using e.g. TdTomato expression as a marker for cre-recombination, OXPHOS enzyme presence and mtDNA copy number (Ignatenko et al. 2018), we agree with the Reviewer that we had not verified whether TdTomato would in the same mice mark for successful cre-recombination in the retina. To answer the question of the Reviewer, we tested whether mtDNA and its encoded protein cytochrome c oxidase (COX-I encoded by MT-CO1) are decreased in amount, because the mtDNA depletion is the direct consequence of depletion of Twinkle, the replicative helicase of mtDNA. Twinkle regulates mtDNA copy number, but not feasible to quantify in reliable manner in the cell dense retina, because of its low copy protein amount (Tyynismaa et al. 2004; Milenkovic et al. 2013; Ignatenko et al. 2018). We used an antibody against double-stranded DNA (a clone detecting mtDNA) on GFAP-CRE tdTomato and COX-I staining co-stained with GFAP antibody on retina sections.* ***Both dsDNA and COX-I were decreased in the astrocyte layer, strongly supporting the conclusion that Twinkle is deleted. The data are now added in Figure EV3, including example images of the quantification and the quantification strategy by CellProfiler (Fig EV2).***

4) Referring to this phenotype as pathological may be a stretch. Taken together, there is a slight increase in vessel area, one more junction per average, and a "gliosis" response which is under quantified but displayed as one more microglia per field of view and one small picture of GFAP induction. What is the consequence of this phenotype on the retina? The structure is fine (Fig 1B), the retina shows no cell death and the physiology seems not impacted. The narrative of the story regarding neovascular retinopathy does not seem to match the results, there is often reference to "wide-spread reactive gliosis", "major structural changes", etc. I think this model is convincingly showing that retinal glia mitochondria are involved with keeping glia in a state that helps maintain vessel homeostasis, beyond that may be overstated.

Au: Thank you for the important points. ***We have revised the quantification of the microglia and vasculature to display absolute numbers, for improved clarity****. The reviewer may have misunderstood our method: the microglia were quantified in wholemount images, not cross-sections. Our analysis reveals that TwKO^{Astro} mice have on the average more than twice as much microglial area and perimeter and exhibit several hundreds more numerous vascular branch points per image (Fig 2A).* ***To enhance clarity, we have added an inset to Figure 2A that highlights only the branch points detected by AngioTool,*** *illustrating how the software traces them. We note now that the images of reactive astrocytes stained for GFAP were unfortunately previously affected by compression, resulting in non-optimal quality. They are now available in their original high resolution.*

Our data demonstrate that mitochondrial dysfunction in retinal astrocytes and Müller glia leads to widespread reactive astrogliosis, remarkable pathological neovascularization, retinal blood vessel leakage, and secondary microglial activation, together indicating severe pathology.

Additionally, we show that the expression of angiogenic factors Fgf2 (Fibroblast Growth Factor 2), Pgf (Placental Growth Factor) and the retinal microvasculopathy inducer Lcn2 (Lipocalin-2) is highly upregulated in the retina, along with neuroinflammatory cytokines and components of the complement cascade. Notably, similar vascular pathology was not observed in the cortex of the same mouse model, which shows instead spongiotic degeneration as a consequence of vast astrocyte reactivation because of TwKOastro. Also, other mitochondrial disease models of ours lack the neovascularization. Our findings highlight region-specific roles of astrocytes in disease pathology and underscore the ***essential role*** *of mitochondrial signaling in macroglia of retina for maintaining vascular homeostasis. Astrocyte/Müller glia reactivation in our model is the primary pathology driver, then followed by microgliosis and vasculopathy.*

5) Would the phenotype of Twinkle knockout not be seen until later ages? It was mentioned by 7 months animal well-being is significantly compromised. I'm confused about the age comparisons made here. The methods say the TwkO astro line was used at 4 and 4.5 months of age versus the control GFAP CRE Td Tomato at 1-2 weeks old? This is not an appropriate comparison for gliosis and neuroprotection experiments if this is the two age groups being compared? The control animals need to be age matched to the knockout especially because Muller glia are not mature at 1-2 weeks.

Au: Thank you for pointing out potentially unclear explanation. In all our experiments, littermates are used as controls, as was mentioned in the methods of the original manuscript, in the current ms p. 14: "Littermates were used as controls", ***and now also in the text. P. 6.*** *The majority of the experiments involved 4-4,5 months old TwKO^{Astro} mice and their littermates as we were interested to capture the vasculopathy in its early stages and because of animal welfare and ethical reasons concerning mice with a severe progressive disease. Gfap-Cre tdTomato mice, aged 1-2 weeks as well as 4-5 weeks were used specifically to validate Cre recombination in retinal astrocytes and Müller glia. Please note that Twinkle protein, mtDNA, its translation products (proteins of oxidative phosphorylation complexes) have long half-lives, and the phenotype progresses over months before it is detected. After that the wellbeing of the mice is fast worsening.* ***We have revised the manuscript to clarify this for the reader (p.13 Limitations of study).***

6) In the differentially expressed transcripts from the RNA-seq data cone arrestin (arr3) was significantly downregulated. Is there loss of these photoreceptors? The authors look at rod-coding and RGC numbers but are they missing a phenotype? The neuroprotection counts also only using

Pou4f1 staining which is not in all RGCs, there may be other vulnerable neuron populations in this model that were not assayed.

Au: Yes, Arr3 expression is significantly down-regulated in our data (FC=-0.74, padj<0.001). However, we assume that the reviewer meant Brn3a staining instead of Pou4f1, as we did not carry out the latter. While the possibility of other cellular types secondarily contributing to the phenotypes is likely, the initial cause of the phenotype is the TwKO in astrocyte mitochondria. Our point is that macroglial mitochondrial metabolism affects retinal metabolic homeostasis, and the consequences can trigger vasculopathy. ***We have now added the point that secondary effects in other cell types, triggered by reactivated macroglia with TwKO, can affect the pathogenesis, but still the primary cause is macroglial TwKO.*** *(Limitations of Study, p.13).*

Reviewer #2:

The manuscript investigates the effects of a macroglia-specific knockout (KO) of the mitochondrial helicase TWINKLE, revealing microgliosis, macrogliosis, and vascular anomalies. While the topic is compelling and merits further exploration, the study has significant shortcomings. These include insufficient validation of the TWINKLE KO in retinal macroglia, a lack of detailed experimental descriptions, limited integration of state-of-the-art omics approaches, and overinterpretation of the presented data in this specific study. Due to these issues, I cannot support the publication of the manuscript in its current form.

1) In the present study, genetic targeting of the mitochondrial helicase TWINKLE leads to vascular abnormalities. The authors speculate that similar changes also lead to vascular defects observed in diabetic retinopathy (DR), which is a cause of systemic metabolic dysregulation and thus most likely leads to mitochondrial dysfunction, but for quite different reasons than genetic modulation as done in the present study. Please discuss how these two settings can really be compared? Is downregulation of TWINKLE observed in DR?

Au: Thank you for the question. Our model is one for severe energetic defect in astrocytes, caused by progressive depletion of machinery for oxidative energy metabolism. Twinkle inactivation has its effects gradually: the protein itself has a long half-life. The depletion of Twinkle leads to depletion of mitochondrial DNA, the replication of which is dependent on Twinkle. MtDNA also has a long half-life in non-dividing tissues. MtDNA encodes for specific proteins of oxidative phosphorylation, which also have a long half-life. Therefore, by inactivating Twinkle, we cause a progressive gradual decrease of oxidative ATP production in macroglia, a minor cell population in retina. The loss of oxphos activity makes the cells totally dependent of glucose as a fuel, by fermentative glycolysis, which requires glucose uptake. glycolytic energy metabolism increases glucose uptake; we have not shown it, but otherwise the cells would not be alive.

Therefore, we are not claiming that Twinkle itself is crucial for DR, but we show that mitochondrial metabolic defect in a minor cell population in retina, the astrocytes and some Müller glia, manifests with key features associated with diabetic retinopathy: pathological retinal neovascularization, microaneurysm-like abnormalities, vascular leakage, microglia activation, complement factor response and inflammation. We show that the same animals, having vast reactivation of astrocytes in cerebral cortex and hippocampus, show no vascular abnormality in these cerebral regions, indicating that ***glycolytic, reactivated astrocytes/Müller glia in retina can trigger a response that leads to all findings typical for diabetic retinopathy.*** *Our future question is whether mechanisms of*

retinopathy in glucose-dependent astrocytes, which form vascular walls, and high blood glucose in diabetics that affect vascular walls are overlapping.

To conclude, we do not suggest that the causes and mechanisms are identical. Rather, we emphasize that the retinal changes are similar which raises interest in the minor macroglial cellular population and mitochondrial metabolism in retina as a factor in diabetic retinopathy. We argue that disturbances of unique aspects of glial mitochondrial homeostasis should be considered as a contributor to the pathology of DR, even though in the DR state the root cause of glial mitochondrial stress is not a loss of Twinkle itself but rather systemic metabolic dysregulation. This model has the potential to provide valuable insights into the intrinsic mechanisms of neovascular retinopathy, and of mitochondrial metabolism and macroglia in maintaining retinal vascular homeostasis.

We have now tuned down the discussion somewhat of the similarities with DR.

2) Introduction, page 3: The numbers on cell types seem to refer to mouse - please specify this, since numbers for other species (pig, human) are different (the percentage of glia in the tissue is higher).

Au: Thank you for noticing this inaccuracy. We have now amended this in the introduction.

3) Introduction - page 3: The description of the retinal cytoarchitecture is quite wrong in many aspects.
(i) Retinal astrocytes are more likely to be found in the nerve fiber layer, where the superficial retinal plexus is located, than in the GCL. Surely the typical retinal astrocyte does not interact with the optic nerve (in the optic nerve, of course, there is a population of astrocytes, but it is certainly distinct from those in the retina). Please correct.

(ii) The sentences "Apart from the outer photoreceptor layer, Muller glia's extensive cell bodies span the rest of all the retinal layers, interacting with all cell types." is wrong. The outer stem process of Müller cells reaches the outer limiting membrane (in fact, they form it), and Müller cell velate sheaths heavily envelop photoreceptor somata in the ONL. Müller cell microvilli even extend into the subretinal space and interact with photoreceptor segments. Please correct.

Au: We agree with the Reviewer and have now made the description of the retinal structure accurate, as suggested.

4) The authors missed to cite literature in which the role of Müller cells in the context of for example diabetic retinopathy was studied (e.g. work done by the Reichenbach & Bringmann labs - for instance see Krügel et al., Involvement of oxidative stress and mitochondrial dysfunction in the osmotic swelling of retinal glial cells from diabetic rats. Exp Eye Res. 2011 Jan;92(1):87-93. doi: 10.1016/j.exer.2010.11.007. or Pfaller et al., The glucocorticoid receptor as a master regulator of the Müller cell response to diabetic conditions in mice. J Neuroinflammation. 2024 Jan 25;21(1):33. doi: 10.1186/s12974-024-03021-x.) Please go back to the literature and add relevant work.

Au: We have incorporated those relevant studies into the references 34 and 35. Here we wish to point out that in our quantification of TdTomato-positive Müller glia, only a minority of them are positive (see our response to point 6), whereas the astrocyte lining shows remarkable reactivity, focusing the interests especially in retinal astrocytes.

5) "The diseases can manifest as extraocular muscle weakness, optic atrophy or retinopathy, which typically is pigmentary [11]" - what is meant with pigmentary? This is quite a strange term. Do the authors mean that symptoms are similar to what is observed in retinitis pigmentosa? Please clarify. Moreover - what is a licencing DNA helicase?

Au: Thank you for your accuracy in terminology. We indeed were meaning retinitis pigmentosa being the most common type of mitochondrial retinopathy. ***We have now corrected the term as implied by the reviewer. (p. 4)***

A licensing DNA helicase is a helicase that plays a role in initiating DNA replication. In this context, Twinkle as a licensing helicase initiates mtDNA replication, and by doing this, Twinkle regulates mtDNA copy number (for example, Tyynismaa et al. 2004). ***For clarity, we have now removed the word "licencing" as it is not crucial for the message of this paper and explained the role in a more clear manner, p. 4. "Essential replicative helicase of mtDNA, initiating mtDNA replication and being the main regulator…"***

6) Results - Validation of KO in the retina (Fig.1). Typically, Müller cells in the healthy retina express very low levels of GFAP. Consistent with this, it appears (Fig. 1A) that by far not all Müller cells are dtTomato positive (looks like less than 50% of them!). This in turn means that there is a mosaic of some recombined and many more non-recombined Müller cells. I lack data to show actual numbers for the proportion of the latter. I assume that depending on the recombination efficacy of the GFAP driver in different parts of the CNS, different consequences will be observed, not necessarily due to different mechanisms, but primarily due to the fact that different proportions of macroglia are affected by the KO. This aspect should be much better addressed in order to draw solid conclusions. In this regard, a co-staining with established astrocyte and Müller cell markers are highly recommended.

Au. We quantified the fraction of Muller glia, which had undergone CRE-mediated recombination by crossing TwKOAstro mice to ROSA26-CAG-TdTomato reporter line and quantifying TdTomato signal gated on Sox9+ cells. The amount is about 20% that underwent the excision. ***Representative images and description of quantification are now added to Fig. EV1*** *and the main text.*

7) It should be stated also at least once in the results part at which developmental time point the authors think the KO hits the macroglia. Would that before or after vessel ingrowth into the retina? Or the other way around - the neovascularization mention in the first section of the results is indeed neurovascularization or rather a disruption of angiogenesis during development? I miss a systematic approach to address this key question in this manuscript as it seems as only adult mice were studied and no earlier timepoints of retinal development. Generally, please state the age of the mice studied throughout this manuscript accurately (at least in figure legends). Searching for that information, I found the puzzling info that the Gfap73.12-Cre:LSL-tdTomato used to demonstrate the cell type-specific recombination (Fig. 1A) were 1-2 weeks old (this is before Müller cells actually are fully mature or even formed (1 week old)!) while subsequent analysis on the TWINKLE-Astro mice were performed in 4.5 months old mice. This is very strange. Please provide age-matched data.

Au: We used the Gfap73.12-Cre:LSL-tdTomato model to show that Cre recombination begins at 1-2 weeks of postnatal age, as we had reported in Ignatenko et al. Nat Comm 2018. After Twinkle gene inactivation, as explained in detail in our response nr 1. to this reviewer, Twinkle depletion leads to

mtDNA depletion, and the mtDNA-encoded proteins of oxidative phosphorylation become gradually deficient. All these components (Twinkle, mtDNA, oxphos proteins) have long half-lives, and therefore the physiological effect of Twinkle loss is gradual and takes months. Given that the retinal angiogenesis in mice is typically completed by postnatal day 21, the vessels are fully formed by the time mitochondrial dysfunction in retinal microglia begins to manifest.

We have added now the data from two-week and five-month-old tdTomato retinas, co-stained with Sox9, along with quantifications in the Figure EV1 B, C.

8) Fig. 1D,E - why present microglia in a retinal section when they were actually quantified in flatmount preparations. This should be streamlined. Did the authors discriminate between in specific retinal layers (e.g. OPL versus inner retina) to see a trend as to where the cells would accumulate due to the tissue stress?

Au: Thank you for the comment. ***To address the comment, we have now microglia images from both retinal cross sections (Fig. 1D) and whole mount (Fig. E-F).*** *Our observations indicate no noticeable difference in microglia distribution across the layers. We performed the quantification of microglia-related parameters in whole mount images since we were able to capture the whole microglial cytoarchitecture much better in this format.*

9) Does KO of TWINKLE lead to loss of macroglia? Would be important to check this. This would explain some of the observed tissue responses also observed when Müller cells were ablated (Shen et al., Conditional Müllercell ablation causes independent neuronal and vascular pathologies in a novel transgenic model. J Neurosci. 2012 Nov 7;32(45):15715-27. doi: 10.1523/JNEUROSCI.2841-12.2012; Shen et al., Effect of glucocorticoids on neuronal and vascular pathology in a transgenic model of selective Müller cell ablation. Glia. 2014 Jul;62(7):1110-24. doi: 10.1002/glia.22666.)

Au: To address the question of cell death as a consequence of the primary astrocyte/Müller cell reactivation, ***we have now stained retinal sections with Fluoro-Jade C (FJC), a marker of degenerating/dying cells.*** *We quantified FJC signal as gated on TdTomato-positive cells as shown below. We did not observe any notable difference between controls and TwAstro KO cells.* ***These results of the quantification are now added in page 5, figure EV1 D-E.***

10) Besides - even though this mouse model is published, the authors should demonstrate a direct proof that TWINKLE is indeed knocked out in Müller cells or retinal astrocytes. I miss such kind of data.

Au: Hereby we provide the requested data. We measured mtDNA and COXI levels from TdTomato and GFAP-positive gated areas, mostly astrocyte layer. It is well established that loss of Twinkle leads to progressive mtDNA depletion (e.g. Tyynismaa et al. 2004; Milenkovic et al. 2013; Ignatenko et al. 2018) and consequent progressive depletion of proteins encoded by mtDNA such as COX-I. Twinkle shows low abundance, and its amount reflects the mtDNA amount. Because the signal of this low-abundance protein would be "loss of signal", reliable data from the mosaically targeted Müller cells in the cell-dense retina was not feasible. ***Our new data show that mtDNA and COX-I are reduced in TwKO mouse retina astrocytes, lining the retina-vitreal body interface, showing that Twinkle is knocked out from retinal astrocytes. Data are presented in Fig. EV3 and the strategy of quantification in Figure EV2.***

11) Page 5 - the statement about GFAP is wrong or at least misleading. Retinal astrocytes of the healthy retina express high levels of GFAP, while Müller cells don't (see above). Please rephrase.

Au: The resting Müller cells express minimal levels of GFAP, while resting retinal astrocytes exhibit higher levels of GFAP expression. ***We have rephrased the tex****t to indicate that the expression of GFAP in Müller cells and retinal astrocytes in the TwKO^{Astro} model was higher compared to the expression of GFAP in these cell types in the control group.* ***Page 5.***

12) Microgliosis is stated on page 5 without specifying the readout parameter to which this statement refers (I assume simple cell counts?). Staining for microglial activation markers (e.g. TSPO or CD68) would be good additional indications.

Au: The Reviewer's interpretation is correct. In our original submission microglia analysis was limited to the count of IBA1 positive cells. ***Here, we provide new data with CD68 staining in addition to IBA1 staining. The results are presented in Fig 1E-F.*** *Briefly, we find that IBA1 perimeter and area are increased in KO retina and CD68 count in IBA1-gated areas is significantly increased as well.*

13) Figure 2. There is no explanation of what the dots in the bar graph represent. Does one represent the result of a single mouse? So the number of biological replicates is <20?

Au: Thank you for your careful reading. Each dot represents an individual measurement, with three measurements per mouse. ***We have now added this information, along with the n-numbers for each genotype, to the figure legend.***

14) It is unclear how the data about the function of the paravascular clearance system in the brain fit into this paper as no comparable data for the retina - the main focus of the present work - are provided. They can be removed for the sake of streamlining this work.

Au: Upon discovery of the retinal vasculopathy, we carefully also explored different regions of the brain. We were surprised that we did not find any structural differences in vascular organization in cortex or hippocampus, even though astrocyte reactivation was remarkable. Therefore, we opted for a thorough analysis of the nervous system vascular function, including paravascular clearance. We find this negative finding important and ***prefer to keep the data in the manuscript as they add additional perspective to the unique vulnerability of retinal macroglia as opposed to cortical astrocytes.***

15) The additional mouse models (Deletor, Mutator, Ndufs4 knockout mice) used for comparison are not well described - please provide more details here - the reader does not want to do a literature search to get this information. E.g. if they are cell type specific, if they are conditional - when and in which cell type effects can be expected... Otherwise it is very difficult to draw conclusions about the results of the respective experiments.

Au: Thank you. ***We have expanded the details on the mouse models to enhance clarity and improve the understanding of the respective experiments (p.6).*** *The other models were constitutively, ubiquitously expressing models (Deletor; Twinkle patient mutation), knock-in (Mutator) or knockout (NDUFS4KO), and all affect downstream the mitochondrial respiratory chain, as also TwKO-astro.*

We cannot therefore fully exclude the possibility that the metabolic homeostasis as a consequence of every cell carrying the genetic defect could compensate for vascular signaling caused by the astrocytic KO of Twinkle being specific for the cells (the current TwKO-astro mice) but find that unlikely. ***We have also included this aspect into limitations of the study, p. 13-14.***

16) It is stated that in the Deletor mouse "transgenic overexpression of a dominant mutation in Twinkle, leading to mtDNA deletion accumulation" - what does it mean? That mtDNA is deleted or accumulated?

Au: The more accurate term is "leading to accumulation of multiple large mtDNA deletions. These deletions accumulate over time. ***We have edited this expression now for the sake of clarity, p. 6****.*

17) It is stated that "Importantly, all the tested models, including TwKOAstro, share the downstream consequence of respiratory chain deficiency." This is a very bold statement not supported by data in the manuscript (and potentially also not by existing literature). Whether this is indeed the case needs to be demonstrated for the retina and the specific cell types therein. Extrapolating this from the results of other cell types/tissues that may have been studied in these different mouse models is not a good practice, as the effects of the genetic manipulations may certainly be quite different depending on the cell type/tissue and their need/expression profile of interactors of TWINKLE.

Au: Thank you. We would like to remind the reviewer that all the genes targeted in these mouse models are crucial for the function of the respiratory chain. The field of mitochondrial medicine started on the finding from 1988 (Holt et al. Nature) that mtDNA deletions cause respiratory chain enzyme deficiency, because mtDNA encodes the core subunits of the mitochondrial respiratory chain. The literature on the subject is vast, from 1988 onwards until today, with recent example reviews e.g. Gorman et al. 2017 Nat Rev Dis Primer; Suomalainen, Nunnari; Cell 2024; Battersby & Suomalainen Nat Rev Mol Cell Biol 2017. Depletion of mtDNA is the downstream consequence of Twinkle KO, because Twinkle is the only replicative helicase of mtDNA, and without Twinkle, mtDNA cannot replicate. Depletion of mtDNA is the most severe cause of respiratory chain deficiency, as it affects all mtDNA encoded subunits of oxidative phosphorylation complexes I, III, IV and V. In NDUFS4KO, the genetic manipulation of the nuclear gene removes a subunit of Complex I of respiratory chain (largest of oxidative phosphorylation complexes), thereby removing the activity of the whole megacomplex of Complex I and this is needed in every cell. Further, random mutagenesis is a consequence of POLG exonuclease deficiency of mutator. Every cell that has mtDNA (except for mature red blood cells) has to replicate, and without exonuclease activity of POLG the point mutations accumulate and cause respiratory chain deficiency. Deletor mouse expresses a Twinkle mutation homologous to that in patients, who develop multiple large partial deletions of mtDNA in postmitotic tissues, with especially high amount in the nervous system. ***We have now added in p. 6 basic knowledge on the genetic change and respiratory chain / oxidative phosphorylation defects.***

For TwKOastro, we have added immunofluorescent stainings of mtDNA (dsDNA, clone with mtDNA as antigen) and cytochrome c oxidase subunit I (core mtDNA encoded subunit of respiratory chain complex IV) staining compared to TdTomato expression or GFAP-positivity, respectively. These stainings show that in the astrocyte lining of retina, with GFAP-promoter-driven cre-expression, mtDNA is depleted and consequently COX-I, mtDNA-encoded protein, is depleted. The data is in Fig EV3 and the used strategy in EV2.

18) Performing bulk RNA-seq is not state of the art if the interest is on a cell population that is a clear minority in the tissue (as also stated by the authors as limitation of the study). The authors tried to overcome this limitation by checking top DEGs for cell type-specific expression in a published scRNA-seq data set, but for this analysis, even though top DEGs clearly indicate a key role of microglia (interferon, interleukins, C1q) where not included in the analysis. This is a major flaw. Please add this key cell type if it comes to understand tissue inflammation.

Au: ***We have now updated Table 1 to include information on microglia, and the text on page 9 has been revised to reflect the new conclusions drawn from this analysis.*** *Our analysis revealed that roughly half of the upregulated genes originated from retinal macroglia, while around a quarter were mainly expressed by retinal microglia.*

19) GSE60361 refers to a study that states "Single-cell RNA-seq of mouse cerebral cortex" and not retina. Please double check and correct. The cited reference refers to the old Makosco study from 2015 - this was run on P15 retina which again is not mature yet. There much better mouse retinal scRNA-seq studies out (even from diabetes models; Van Hove et al., Single-cell transcriptome analysis of the Akimba mouse retina reveals cell-type-specific insights into the pathobiology of diabetic retinopathy. Diabetologia. 2020 Oct;63(10):2235-2248. doi: 10.1007/s00125-020-05218-0; Niu et al., Pathogenesis Study Based on High-Throughput Single-Cell Sequencing Analysis Reveals Novel Transcriptional Landscape and Heterogeneity of Retinal Cells in Type 2 Diabetic Mice. Diabetes. 2021 May;70(5):1185-1197. doi: 10.2337/db20-0839) that would allow better conclusions on expression profiles of retinal cell types in adult retina and retinae with potential neovascularization. For complement expression in retinal cell types, you may also want to check on Pauly et al., 2019 (Cell-Type-Specific Complement Expression in the Healthy and Diseased Retina. Cell Rep. 2019 Nov 26;29(9):2835-2848.e4. doi: 10.1016/j.celrep.2019.10.084.)

Au: Thank you for careful reading and noticing the mistake in the accession number, which has now been corrected.

As asked by the reviewer, we reanalyzed the dataset of Akimba mice with the goal to identify the retinal cell types of interest (Müller cells, rods, bipolar cells, astrocytes). In line with our study, astrocytes could not be distinguished with certainty in Akimba mice either, most likely due to their very low representation in the cellularity of retina (below 0.5%). In particular, no Gfap-positive cells were detected in the control sample, as expression of GFAP in non-reactive astrocytes and Müller cells is low. We were able to annotate Müller cells, rods, and bipolar cells.

Because only two biological samples were available in the suggested dataset (control and Akimba), the results are reported as log2 fold changes without statistical testing, as variance could not be estimated.

*Our comparison between the TwKOAstro retina dataset and the Akimba retina single-cell data show concordance. The majority of key inflammatory and glial activation genes (including C4b, Serpina3n, Ccl12, Pgf, Gfap, A2m, Cebpd, Lcn2, C1qa, Calca, Ly86, and Chil1**)** are upregulated in both datasets (Table 1), consistent with their role as an important responder to retinal stress. Rod photoreceptors and bipolar cells also display upregulation of several inflammatory mediators, although in some cases the magnitude is lower. Overall, the low n-number of Akimba samples leaves conclusions thin. Also, the reanalysis indicates that astrocytes cannot be identified from single-cell or bulk-RNAseq as*

an independent population in retina. ***The data are included in the manuscript but the point of low ability to conclusions is mentioned. p.9.***

20) The authors did not try to validate any of the candidates they pulled out from their somewhat outdated bulkRNA-seq approach - at least this should have been done.

Au: This is unfortunately true that in protein level we could not detect the signals. We did several attempts with different antibodies to detect pro-angiogenic factors FGF2 or PGF, but as secreted factors, these were not successfully detected by Western blot or by immunofluorescent staining. Also the staining with various complement factors was not reliably interpretablel. The follow-up study may include using inhibitors against these factors, but this is not in the scope of this study.

21) Fig. 6G - please provide an exemplary Brn3 image for both genotypes. The figure legend should provide the information whether cells were counted in flatmounts or sections. Generally, the level of neuronal loss should be better characterized also checking on cells of the inner nuclear layer.

We have revised the figure legend of Fig6G to clarify that the Brna3a-positive cells were quantified in wholemount retinal images. Additionally, ***we have included the images of wholemount retinas immunostained against Brn3a (Suppl. Fig. 3),*** *which were used for ganglion cell quantification. We have* ***stained retinal sections with Fluoro Jade C (FJC) as a marker of degenerating cells****. We quantified FJC signal as gated on TdTomato-positive cells as shown below. We did not observe any notable difference between controls and TwAstro KO cells, suggesting that there is no cell death.* ***These results are included in the Fig EV1.***

22) Given that the hypothesis is that mitochondria are affected and dysfunctional I am supprised that the manuscript does not provide any data on the mitochondrial fitness of Müller cells/astrocytes in their model (as they also do not prove the actual knockout of TWINKLE - see my comment above). This is worrisome and could/should have been done.

Thank you. Twinkle is very low in expression in all tissues, and its absence vs presence is not possible to analyze by immunofluorescence (not shown). Instead, we measured the direct target of Twinkle: mtDNA, and mtDNA-encoded protein cytochrome c oxidase subunit I (COXI) amounts from TdTomato and GFAP-positive gated areas. It is well established that loss of Twinkle leads to progressive mtDNA depletion (e.g Tyynismaa et al. 2004; Milenkovic et al. 2013; Ignatenko et al. 2018) and depletion of proteins encoded by mtDNA such as COX-I. We show that both mtDNA and COX-I arereduced in Twinkle-Astro KOs, consistent with loss of Twinkle function in astrocytes and Müller Glia. Data are presented in Fig. EV3 and the quantification strategy in Fig EV2..

Given the many issues I do have with the results part of the manuscript, I keep my feedback very short about the discussion.

Au: thank you for your constructive comments that improved the manuscript, while not changing the original conclusions.

23) The author spent quite a big part of their discussion on DR which brings me back to my concern why they took the P15 scRNA-seq data set from Makosco et al to validate their bulk RNA-seq data

and not one of the multiple scRNA-seq data sets from DR mouse models (see above). In its current form, to elaborate that much on DR in the discussion does not make too much sense.

Au. Please see our response to point 19 with the same question by this reviewer.

24) Similar effects as described in the TWINKLE Astro model were observed in a model of Müller cell ablation, but the latter is not even mentioned (Shen et al., Conditional Müllercell ablation causes independent neuronal and vascular pathologies in a novel transgenic model. J Neurosci. 2012 Nov 7;32(45):15715-27. doi: 10.1523/JNEUROSCI.2841-12.2012; Shen et al., Effect of glucocorticoids on neuronal and vascular pathology in a transgenic model of selective Müller cell ablation. Glia. 2014 Jul;62(7):1110-24. doi: 10.1002/glia.22666.) Please add and properly discuss.

Au: Thank you for the suggestion. ***We have now included the reference into the paper, ref 48.*** *. In our case, VEGF-A was not induced, which was a surprise to us. Instead, two angiogenic factors, PGF and FGF2, were increased.*

25) Closing my review with highlighting why I have issues with the interpretation of the data in this study citing the first sentence of the final conclusion of the manuscript "In conclusion, our data highlight the role of mitochondrial metabolism and macroglia in regulating retinal vascular homeostasis and their intriguing potential roles in neovascular retinopathy of diabetic type". There is any data in the manuscript that gives insight into the mitochondrial metabolism of macroglia!

We acknowledge the reviewer's feedback and ***have accordingly revised the manuscript's conclusion, p. 13.***

Minor comments:

1) It would be nice if the Müller cells were really written with an "ü" and not spelled "Muller cells".

Thank you, corrected.

2) Labelling of the retinal layers in all figures is highly recommended.

Yes, absolutely. ***We have labelled the layers in all the retinal cross-section images.***

Referee #3 (Comments on Novelty/Model System for Author):

The technical quality of the retinal sections and microglia analyses are limited. As detailed in my

reviewer comments below, up to date histology and quantitative morphometry should be used in a revised version.

Au: Unfortunately, the figures were submitted alongside the manuscript text in a Word file, which account for the lower image quality observed by the reviewers, when actually all the immunofluorescence images included in the manuscript were acquired using a confocal microscope (LSM780 and ANDOR Dragonfly 505) at a resolution of 1028×1028 or higher. ***We have now confirmed the delivery of high-resolution images.***

Referee #3 (Remarks for Author):

This paper reports that cell specific deletion of Twinkle in retinal Müller cells and astrocytes causes (aberrant) retinal neovascularization. These findings are interesting and provide a new mechanism and target pathway (mitochondria) for retinal neo-angiogenesis. The authors compared Twinkle-deficient mice with several other mitochondrial mutant mice (Deletor, Mutator, NDUFS4) and showed specific effects only for this gene knockout.

I find the paper really interesting, but my enthusiasm is very limited because the manuscript has several text problems, and the technical quality of the retinal histology analyses are partially of poor quality.

Major points of criticism:

1) In general, I would recommend to change the figure subpanel labelling from Figures 1 and 2 as it is confusing. Here subpanels should be labelled from left to right in the appropriate order. For example, in Figure 1 B is followed by E and in Figure 2 A is followed by G.

Au: We have now replaced the panels in order, from left to right, top to bottom.

2) The authors should realign the images from subpanel A, C and E that the retina is correctly positioned with GCL facing down (or up). Also, the retinal layers should be labelled accordingly.

Au: We have now changed the orientation of the retinal images as requested, and marked the retinal layers in all the figures.

3) Figure 1C: Please indicate the layer and the area from which these images were taken. I assume its GCL. In addition, the authors should do a combination of glutamine synthetase (GS) and GFAP staining to assess Müller cell reactivity. A quantification of these GFAP stress fibers would also be helpful.

Au: The whole mount GFAP staining is a z-stack collapse and represents all the layers. ***This information is now added to the figure legend.*** We *performed the GS staining on tdTomato sections but overlap was not observed in our hands(not included). However, our Sox9 staining on tdTomato sections (new data) indicates that only less than 20% of Müller glia underwent TwKO.* (Figure EV1 B, C). GFAP area quantitation on retina sections shows the significant increase in TwKO^{astro} retina ***(´Now added to Figure EV4 B)***

4) Figure 1D: Please define what field of view means.

Au: The number of microglia cells was quantified from wholemount images. Six random locations in each retina were imaged, using a 20x objective on the confocal microscope, with IBA1 staining to visualize the cells. The number of cell bodies in each image was counted, which is referred to as "per field of view." The average cell count per retina was then calculated and used for statistical analysis. ***We have now included clarification in the figure 1F legend.***

Precise information must be provided here. Also here, the authors cannot conclude from these Iba1-stained cryosections that microglia are activated. This is an overstatement. Here, only numbers of microglia per field of view were counted. It is also not clear if microglia were counted in all layers or just IPL or OPL? Cryosections are normally used to analyze the localization of microglia cells. The authors need to complement this analysis with retinal whole mounts stained with Iba1 in order to assess retinal morphology at least in OPL and IPL and also conduct morphometric analysis such as analysis of ramification index, total and spanned area, number of branches, junctions and endpoints.

Au: Thank you. To assess the morphology of the microglia cells, IBA1 and CD68 stainings were carried out on retinal whole mount tissue and representative images are shown in Fig. 1D-F. Quantification is presented in Fig. 1F. We observed that the morphology of the microglia are not typical of a fully reactivated state. Our findings of increased IBA1 area and perimeter, as well as CD68 intensity in IBA1-gated regions is consistent with a hyper-ramified state. ***We have updated the wording of this section accordingly in Results section, p. 5.***

5) The authors conclude that there are no differences in retinal structure and tissue organization of TwKOAstro and control mice based on retinal cryosections. Here a panorama view from retinas of both mouse lines would be helpful to show the overall retinal structure. Also, OCT scans and analysis of retinal thickness would be helpful and document the findings.

Au; We wish that our current images in EV1 and figure 1 indicating the layers, as well the added data of Fluoro-J, suggesting no dying or degenerative cells would respond sufficiently to this question. We do not have OCT available for mouse studies.

6) Methods: The authors should indicate for the animal experiments whether mice from both sexes were used and include a statement about potential sex differences.

Au: Thank you for this comment. ***We have now included details on the genders used for each mouse model in the Materials and Methods section p.14.***

7) Figure 2 B and D: Here the authors normalized the values to the control mice. However, it would be more helpful if the absolute values would be provided.

Au: ***We have changed all figures from normalized values to absolute numbers for clarity.***

8) Analysis of fibrinogen is based on fluorescent intensity. I would recommend to perform Western Blots or ELISAs to validate the strong reduction of fibrinogen in the retina.

Au: Actually, the goal of this experiment is not to quantify overall fibrinogen levels in retina, but to assess its leakage from vessels to surrounding tissues. Under normal conditions, an intact blood-retinal barrier prevents fibrinogen from entering the retinal tissue. When the barrier is compromised, fibrinogen extravasates, which we have demonstrated by measuring fluorescence intensity in blood vessels and adjacent tissues. In control mice, fibrinogen fluorescence was confined to blood vessels. In contrast, in TwKO^{Astro}, there was no significant difference in fluorescence intensity between vessels and tissues, and the fibrinogen signal in the tissue was significantly higher than in control mice. These findings highlight the breakdown of the barrier.

9) Classical complement activation as well as the pro-angiogenic factors identified in transcriptomics should be verified also via ELISAs.

Au: Thank you. We did several attempts to assess complement factor western blots that however did not give a consistent reproducible result. We also tried complement factor IF staining in retinal section, again did not work well. Here, the robust complement pathway activation in RNAseq indicates pathway induction, to our opinion.

10) The authors should show the wholemount images that were used to analyze Brn3a-positive cells (e.g. in suppl. figures)

Thank you. ***We have now included the images of wholemount retinas immunostained against Brn3a (Suppl. Fig. 3)****, which were used for ganglion cell quantification.*

Minor points of criticism:

1) The title is completely misleading as the cell-specific deletion was not done in microglia but macroglia.

Au: Maybe the reviewer misread the title, as it exactly mentions macroglia, not microglia. "Loss of mitochondrial DNA helicase in retinal macroglia drives neovascular retinopathy".

2) Most figures and figure legends lack n-numbers and specification of analysed sample (e.g. eye. resction, cell etc.)

Au: We have ensured that ***all figure legends include the type of analyzed samples and corresponding n-numbers.***

3) Figure 2 subpanel E: Please make the appearance of statistically significant differences consistent throughout the manuscript.

Au: We have adjusted the appearance of statistically significant differences in Figure 2E to be consistent with the rest of the manuscript. However, we have reported exact p-values for two comparisons (p = 0.0502 and p = 0.0506) to highlight their proximity to significance.

4) Figure 4: Information about the scale bar is missing.

Au: Thank you for careful reading. We have added the scalebar information to the figure legend.

5) Muller glia' needs to be 'Müller glia'.

Au: We have corrected "Muller glia' to 'Müller glia' in the manuscript.

19th Dec 2025

Dear Dr. Ince-Dunn,

Thank you for submitting your revised manuscript to EMBO Molecular Medicine, and please accept my apologies for the delay in getting back to you as one referee needed more time to complete their report. We have now received feedback from the three initial reviewers. As you will see from the reports below, while referee #3 is satisfied with the revisions, referees #1 and #2 remain critical and find that several major concerns have not been addressed properly (in particular related to TWINKEL deletion in retinal cells and recombination in Muller cells).
We have further discussed with the referees, and with an expert advisor, who mentioned that "the findings of a relatively mild phenotype are biologically meaningful" and that "the paper has a high relevance for the field and a novel aspect of linking glial metabolism with a vascular phenotype" and overall supported publication of your work. Further taking into consideration the fact that the model system is not new to the revisions, and that all referees mentioned the quality of the study, we decided to invite revisions of the manuscript, in which we would like you to clearly state the limitations of the study and carefully articulate the conclusions of your work.

If you would like to discuss further the points raised by the referees, I am available to do so via email or video. Let me know if you are interested in this option.

We are expecting your revised manuscript within three months, if you anticipate any delay, please contact us.

When preparing your revised manuscript, please refer to our guidelines: https://link.springer.com/journal/44321/submission-guidelines#cms-Revised-submissions. We perform an initial quality control of all revised manuscripts before re-review; failure to include requested items will delay the evaluation of your revision.

We require:

1) A .docx formatted version of the manuscript text (including legends for main figures, EV figures and tables). Please make sure that the changes are highlighted to be clearly visible.

2) Individual production quality figure files as .eps, .tif, .jpg (one file per figure). For guidance, download the 'Figure Guide PDF': https://media.springernature.com/original/springer-cms/rest/v1/content/27825798/data/v1.

3) A .docx formatted letter INCLUDING the reviewers' reports and your detailed point-by-point responses to their comments. As part of the EMBO Press transparent editorial process, the point-by-point response is part of the Review Process File (RPF), which will be published alongside your paper.

4) A complete author checklist, which you can download from our author guidelines. Please insert information in the checklist that is also reflected in the manuscript. The completed author checklist will also be part of the RPF.

5) Please note that all corresponding authors are required to supply an ORCID ID for their name upon submission of a revised manuscript. Please also provide an institutional email address for the corresponding author.

6) It is mandatory to include a 'Data Availability' section after the Materials and Methods. Before submitting your revision, primary datasets produced in this study need to be deposited in an appropriate public database, and the accession numbers and database listed under 'Data Availability'. Please remember to provide a reviewer password if the datasets are not yet public.

In case you have no data that requires deposition in a public database, please state so in this section. Note that the Data Availability Section is restricted to new primary data that are part of this study.

7) For data quantification: please specify the name of the statistical test used to generate error bars and P values, the number (n) of independent experiments (specify technical or biological replicates) underlying each data point and the test used to calculate p-values in each figure legend. The figure legends should contain a basic description of n, P and the test applied. Graphs must include a description of the bars and the error bars (s.d., s.e.m.).

8) At EMBO Press we ask authors to provide source data for the main manuscript figures. You will receive a separate email with instructions for providing source data with your revised manuscript, including how to upload and organize the files.

9) Our journal encourages inclusion of *data citations in the reference list* to directly cite datasets that were re-used and obtained from public databases. Data citations in the article text are distinct from normal bibliographical citations and should directly link to the database records from which the data can be accessed. In the main text, data citations are formatted as

follows: "Data ref: Smith et al, 2001" or "Data ref: NCBI Sequence Read Archive PRJNA342805, 2017". In the Reference list, data citations must be labeled with "[DATASET]". A data reference must provide the database name, accession number/identifiers and a resolvable link to the landing page from which the data can be accessed at the end of the reference.

10) We replaced Supplementary Information with Expanded View (EV) Figures and Tables that are collapsible/expandable online. A maximum of 5 EV Figures can be typeset. EV Figures should be cited as 'Figure EV1, Figure EV2" etc... in the text and their respective legends should be included in the main text after the legends of regular figures.

- For the figures that you do NOT wish to display as Expanded View figures, they should be bundled together with their legends in a single PDF file called *Appendix*, which should start with a short Table of Content. Appendix figures should be referred to in the main text as: "Appendix Figure S1, Appendix Figure S2" etc.

- Additional Tables/Datasets should be labeled and referred to as Table EV1, Dataset EV1, etc. Legends have to be provided in a separate tab in case of .xls files. Alternatively, the legend can be supplied as a separate text file (README) and zipped together with the Table/Dataset file.

11) The paper explained: EMBO Molecular Medicine articles are accompanied by a summary of the articles to emphasize the major findings in the paper and their medical implications for the non-specialist reader. Please provide a draft summary of your article highlighting
- the medical issue you are addressing,
- the results obtained and
- their clinical impact.

This may be edited to ensure that readers understand the significance and context of the research. Please refer to any of our published articles for an example.

12) Author contributions: You will be asked to provide CRediT (Contributor Role Taxonomy) terms in the submission system. These replace a narrative author contribution section in the manuscript.

13) A Conflict of Interest statement should be provided in the main text.

14) Every published paper includes a 'Synopsis' to further enhance discoverability. Synopses are displayed on the journal webpage and are freely accessible to all readers. They include a short stand first (maximum of 300 characters, including space) as well as 2-5 one-sentences bullet points that summarizes the paper. Please write the bullet points to summarize the key NEW findings. They should be designed to be complementary to the abstract - i.e. not repeat the same text. We encourage inclusion of key acronyms and quantitative information (maximum of 30 words / bullet point). Please use the passive voice. Please attach these in a separate file or send them by email, we will incorporate them accordingly.

Please also suggest a striking image or visual abstract to illustrate your article as a PNG file 550 px wide x 300-600 px high.

15) Include a Reagents and Tools Table as part of the Methods section, which can be downloaded from our author guidelines.
16) As part of the EMBO Publications transparent editorial process initiative (see our Editorial at http://embomolmed.embopress.org/content/2/9/329), EMBO Molecular Medicine will publish online a Review Process File (RPF) to accompany accepted manuscripts.
In the event of acceptance, this file will be published in conjunction with your paper and will include the anonymous referee reports, your point-by-point response and all pertinent correspondence relating to the manuscript. Let us know whether you agree with the publication of the RPF and as here, if you want to remove or not any figures from it prior to publication.
Please note that the Authors checklist will be published at the end of the RPF.

EMBO Molecular Medicine has a "scooping protection" policy, whereby similar findings that are published by others during review or revision are not a criterion for rejection. Should you decide to submit a revised version, I do ask that you get in touch after three months if you have not completed it, to update us on the status.
Molecular Medicine.

I look forward to receiving your revised manuscript.

Yours sincerely,

Lise Roth

Lise Roth, PhD
Senior Editor
EMBO Molecular Medicine

***** Reviewer's comments *****

Referee #1 (Remarks for Author):

The reviewers have adequately addressed the majority of my previous technical concerns. This is an improved version from the last but I still feel there are overinterpretations. The mosaic recombination rate of GFAP-CRE being around 6-18% of Muller glia is significant limitation of this approach and potentially masking larger effects. I also do not find the data robust regarding Twinkle deletion in retinal glia. The argument for deletion is indirect showing mtDNA and Cox1 at IHC levels.

A minor point for clarification of my previous comment is that Pou4f1 is Brn3a, and I still maintain that showing no differences in neuronal survival undermines the argument for retinal pathology. There may be differences in neuronal populations which is why it was suggested to look at cones or other RGC-markers. Not that it is the "primary cause" of pathology but it may speak to the biological relevance of the phenotype.

Referee #2 (Comments on Novelty/Model System for Author):

I still have my major issues with the validation of the astrocyte-specific KO of TWINKEL in the retina for multiple reasons: (i) The Fig. EV 1C legend misses the information whether the quantification of SOX9+/dTtomato+ cells was confined to the INL or whether astrocytes were included as well. Less than 20% of recombination efficacy in Müller cells is extremely low. No recombination effic)acy is stated for astrocytes at all - could have easily performed in flatmounts using the SOX9 staining. Fig. EV2 - not clear why the analysis was performed on sections and not flatmounts that would be much more appropriate to study astrocytes. (ii) Additionally, adding to this issue with the mouse model - in Fig. EV 1B and Fig. EV2 it looks like a considerable number of neurons of the inner nuclear layer also recombined (potentially amacrine and horizontal cells)...Most likely this also drives the phenotype in this line. A co-staining with some amacrine and horizontal markers would help clarify this key issue of a potentially leaking GFAP promotor it has already been described by others (see Hu et al., Expression Patterns of Inducible Cre Recombinase Driven by Differential Astrocyte-Specific Promoters in Transgenic Mouse Lines. Neurosci Bull. 2020, PMID: 31828740 and in support of my hypothesis a study on retina demonstrating leaking expression in amacrine cells - Rao et al., Exp Eye Res. 2023, PMID: 37659708). (iii) Moreover, the effectiveness of a Cre-mediated deletion of a target gene also largely depends on the fl/fl construct. Therefore reading out dTtomato signals can only be a very rough approximation for what actually happens to TWINKEL. Given that neurons clearly rely on functional mitochondria, the findings of this study could result form primary degeneration of amacrine and horizontal cells followed by macro- and microgliosis. This, unfortunately, stays a major flaw of this study as all the work hypothesis and interpretation base on the assumption that TWINKEL is exclusively knocked out in macroglia - which does not seem to be the case here.

All in all, these are major flaws of the study. It kind of shows that even after all reviewers asked for a proper validation of the macroglia-specific KO data remain bits and pieces and are not convincing - at least not to me.

Referee #2 (Remarks for Author):

The authors have made a substantial effort to address the reviewers' concerns. However, the specificity of the mouse model for the macroglia-specific deletion of TWINKEL remains highly questionable. Importantly, the additional data provided during the revision process, including the new validation experiments in the retina, do not clarify this issue; instead, they further strengthen my concerns regarding the suitability of the GFAP driver line for addressing the question in a cell-type-specific manner (also see my detailed concerns below):
(i) The GFAP driver exhibits low recombination efficiency in Müller cells; (ii) information on recombination efficacy in astrocytes is limited and not assessed using the same readout as for Müller cells, preventing a direct comparison; and, most critically, (iii) the line appears to be leaky, driving reporter expression and potentially Cre recombinase activity in a substantial number of neurons within the inner nuclear layer. This neuronal recombination in a considerable number of cells (see Fig. EV3) could plausibly explain the observed Müller cell gliosis, microglial activation, and remodeling of the intermediate and deep retinal plexi as secondary consequences of neuronal dysfunction or degeneration and not, as concluded by the authors, by first hitting astrocytes/macroglia.
For these reasons, I cannot recommend this study for publication. As the principal limitation lies in the mouse model itself, I do not see how this issue could be adequately resolved through a classical revision. I regret that I cannot provide a more favorable

recommendation.
Comments/concerns:
1) The following sentence is still incorrect: "Apart from the outer photoreceptor layer, Muller glia's extensive cell bodies span the rest of all the retinal layers, interacting with all cell types." The outer stem process of Müller cells reaches the outer limiting membrane (in fact, they form it), and Müller cell velate sheaths heavily envelop photoreceptor somata in the ONL. Maybe you meant the outer photoreceptor segments instead of a layer. Then please rephrase to avoid misunderstandings!
2) A scheme illustrating the wealth of mouse lines used would be helpful.
3) Results - I still have my major issues with the validation of the astrocyte-specific KO of TWINKEL in the retina for multiple reasons: (i) The Fig. EV 1C legend misses the information whether the quantification of SOX9+/dTtomato+ cells was confined to the INL or whether astrocytes were included as well. Less than 20% of recombination efficacy in Müller cells is extremely low. No recombination effic)acy is stated for astrocytes at all - could have easily performed in flatmounts using the SOX9 staining. Fig. EV2 - not clear why the analysis was performed on sections and not flatmounts that would be much more appropriate to study astrocytes. (ii) Additionally, adding to this issue with the mouse model - in Fig. EV 1B and Fig. EV2 it looks like a considerable number of neurons of the inner nuclear layer also recombined (potentially amacrine and horizontal cells)...Most likely this also drives the phenotype in this line. A co-staining with some amacrine and horizontal markers would help clarify this key issue of a potentially leaking GFAP promotor it has already been described by others (see Hu et al., Expression Patterns of Inducible Cre Recombinase Driven by Differential Astrocyte-Specific Promoters in Transgenic Mouse Lines. Neurosci Bull. 2020, PMID: 31828740 and in support of my hypothesis a study on retina demonstrating leaking expression in amacrine cells - Rao et al., Exp Eye Res. 2023, PMID: 37659708). (iii) Moreover, the effectiveness of a Cre-mediated deletion of a target gene also largely depends on the fl/fl construct. Therefore reading out dTtomato signals can only be a very rough approximation for what actually happens to TWINKEL. Given that neurons clearly rely on functional mitochondria, the findings of this study could result form primary degeneration of amacrine and horizontal cells followed by macro- and microgliosis. This, unfortunately, stays a major flaw of this study as all the work hypothesis and interpretation base on the assumption that TWINKEL is exclusively knocked out in macroglia - which does not seem to be the case here.

All in all, these are major flaws of the study. It kind of shows that even after all reviewers asked for a proper validation of the macroglia-specific KO data remain bits and pieces and are not convincing - at least not to me.

4) Results - Validation of KO in the retina (Fig. EV 1D). Seems like almost all dTtomato+ cells also are positive for FJC in both control and Twinkle KO. To me this rather looks like a bleed through from the dTtomato channel than a real indicator of cell death. Would be extremely careful interpreting such kind of data. A TUNEL assay or caspase staining could/should give much more relevant information.
5) If only 20% of Müller cells are targeted, why do the authors observe changes in all three retinal vascular plexi - only the superficial one is stabilized by astrocytes, while the other two (intermediate & deep) rely on Müller cells only.
6) The authors state that state that validation of candidates was challenging since many of them were secreted factors hard to detect by stainings. True - but for many of the very sensitive ELISA assays exist to measure changes e.g. in retinal samples.
7) I asked the authors to better assess neurodegeneration especially in the INL. They respond that they - on top of Brn3 quantification - added the FJC analysis in dTtomato positive cells. Well, this does not answer my question, since ideally dTtomato+ cells should not be neurons. Besides - coming back to my comment No. 3, it seems like there is leakiness of the GFAP promotor driven Cre expression in neurons... Which makes the whole thing even worse.

Referee #3 (Remarks for Author):

The authors have addressed all my previous concerns in a very thorough revision including several high quality microscopic images.

THE AUTHORS' RESPONSES TO THE REVIEWER COMMENTS:

We would like to thank the Reviewer 3 for appreciation of our extensive revisions and requiring no further revisions and the Reviewer 1 for supportive and constructive comments. For each of the comment of Reviewer 2 we also have responded to. Please note that the EV-figure numbers have been changed to follow the editorial instructions.

Reviewer Comments:

Referee #1:

1. The authors have adequately addressed the majority of my previous technical concerns. This is an improved version from the last but I still feel there are overinterpretations. The mosaic recombination rate of GFAP-CRE being around 6-18% of Muller glia is significant limitation of this approach and potentially masking larger effects. I also do not find the data robust regarding Twinkle deletion in retinal glia. The argument for deletion is indirect showing mtDNA and Cox1 at IHC levels.

Au: *We are happy to hear that the majority of the concerns have been solved. Please note that this model system has already been characterized and published (PMID: 36383135). The main target of the genetic modification is the astrocytes, and these show widespread reactivation in as quantified from whole-mount stainings* ***(New data: Fig 1C quantification of GFAP staining, pseudocoloured in red). The*** *phenotype on retina is significant even with the low efficiency of Müller cell recombination.*

Then of mitochondrial biology. Twinkle helicase is the only replicative helicase of mtDNA. Twinkle is essential for mtDNA replication, and regulates mtDNA copy number. Because of the regulatory role, Twinkle itself is of low copy number protein. Therefore, the most specific and reliable test for Twinkle depletion is measurement of decrease of mtDNA, which leads to progressive depletion of its encoded proteins of the respiratory chain. One of these is cytochrome c oxidase (COX). Therefore, we assessed Twinkle depletion by measuring its functional effects: mtDNA and its product COX-I (Figure EV6). The COX-! Is a core subunit of cytochrome c oxidase, and the enzyme is not assembled without it. ***We have not further revised the paper for this aspect, because the results are clear and strong for Twinkle defect, and further studies would not change the interpretation or conclusions of the data.***

2. A minor point for clarification of my previous comment is that Pou4f1 is Brn3a, and I still maintain that showing no differences in neuronal survival undermines the argument for retinal

pathology. There may be differences in neuronal populations which is why it was suggested to look at cones or other RGC-markers. Not that it is the "primary cause" of pathology but it may speak to the biological relevance of the phenotype.

*Au: The point of the paper is that affecting macroglial mitochondrial DNA replication causes neovascular retinopathy. That is the primary defect and all the downstream effects are consequences of that specific manipulation. **We did not further modify this point, also because no changes were proposed by the reviewer.***

Referee #2 (Comments on Novelty/Model System for Author):

Rev 1. I still have my major issues with the validation of the astrocyte-specific KO of TWINKEL in the retina for multiple reasons: (i) The Fig. EV 1C legend misses the information whether the quantification of SOX9+/dTtomato+ cells was confined to the INL or whether astrocytes were included as well. Less than 20% of recombination efficacy in Müller cells is extremely low. No recombination effic)acy is stated for astrocytes at all - could have easily performed in flatmounts using the SOX9 staining.

Au: The quantification was done from the whole image, and not restricted to INL. We have now added the information to the figure legend and the methods.

Rev 2. Fig. EV2 - not clear why the analysis was performed on sections and not flatmounts that would be much more appropriate to study astrocytes. (ii) Additionally, adding to this issue with the mouse model - in Fig. EV 1B and Fig. EV2 it looks like a considerable number of neurons of the inner nuclear layer also recombined (potentially amacrine and horizontal cells)...Most likely this also drives the phenotype in this line. A co-staining with some amacrine and horizontal markers would help clarify this key issue of a potentially leaking GFAP promotor it has already been described by others (see Hu et al., Expression Patterns of Inducible Cre Recombinase Driven by Differential Astrocyte-Specific Promoters in Transgenic Mouse Lines. Neurosci Bull. 2020, PMID: 31828740 and in support of my hypothesis a study on retina demonstrating leaking expression in amacrine cells - Rao et al., Exp Eye Res. 2023, PMID: 37659708).
re(iii) Moreover, the effectiveness of a Cre-mediated deletion of a target gene also largely depends on the fl/fl construct.

Au: The reviewer is absolutely correct in referring to the basics of making a genetic modification in mice, and that the specificity of the promoter that drives cre and the floxed allele design are crucial. The reviewer may not have noticed that this model has been extensively characterized in previous publications. For example, the Ignatenko et al. Nat Comm 2018, included the detailed description of the flox/flox construct and detailed characterization of the GFAP-cre that expresses only postnatally and targets astrocytes in the cerebrum, and also extensively characterized in our previous paper mentioned above and used in followup papers. This postnatal GFAP-promoter minimally affects retinal neurons and the main target are astrocytes and a small proportion of Müller cells. The issue of many

other GFAP promoters that the reviewer refers to is the developmental expression of GFAP that can lead to partial neuronal targeting. Also please note that the tdTomato is expressed under the exactly the same GFAP-promotor as the cre is, therefore indicating the cells in which the GFAP-promotor drives cre. We refer accordingly to Ignatenko et al 2018 and see little justification to include the full characterization of the mouse generation again, which the editor also acknowledged.

Rev 3: Therefore reading out dTtomato signals can only be a very rough approximation for what actually happens to TWINKEL.

Au: We agree totally with the Reviewer. TdTomato indicates in which cells the GFAP-cre is active and does not test the presence of Twinkle. That is the reason why we test the functional consequences of Twinkle loss by quantifying mtDNA amount and mtDNA product COX-I, the cytochrome c oxidase. Twinkle helicase is the only replicative helicase of mtDNA. Its loss causes mtDNA depletion. Twinkle is essential for mtDNA replication, and regulates mtDNA copy number, which is why Twinkle itself is a low-copy protein. Therefore, the most specific and reliable test for successful Twinkle depletion is measurement of decrease of mtDNA, which leads to progressive depletion of its encoded proteins of the respiratory chain. One of these is cytochrome c oxidase (COX). Therefore, we assessed Twinkle depletion by measuring its functional effects: mtDNA and its product COX-I (Figure EV6). The COX-I Is a core subunit of cytochrome c oxidase, and the enzyme is not assembled without it. ***We have not further revised the paper for this aspect, because the results are clear and strong for Twinkle defect judged by its functional consequences to mtDNA and its product COX-I. Further studies would not change the interpretation or conclusions of the data.***

Rev 4: Given that neurons clearly rely on functional mitochondria, the findings of this study could result form primary degeneration of amacrine and horizontal cells followed by macro- and microgliosis.

Au: We cordially disagree. There is no basis of this claim that postnatally-expressed GFAP driver, that typically targets astrocytes and some Müller cells, with major reactivation of astrocytes, would actually cause mainly a neuronal phenotype. We find major reactivation of the astrocyte layer (Fig 1C wholemount). We find no phenotype in any other cell type or indication of degeneration or death of cell layers, including neuronal layers. <u>The astrocytes were targeted, and the phenotype is astrocytic.</u> Maybe the extent of astrocyte layer reactivation in Figure 1C remained unnoticed by the reviewer, as the pseudo-colour used in Fig 1C was red, indicating GFAP-antibody stained cells (now also quantified); it was not td-Tomato.

Rev 5: This, unfortunately, stays a major flaw of this study as all the work hypothesis and interpretation base on the assumption that TWINKEL is exclusively knocked out in macroglia - which does not seem to be the case here. All in all, these are major flaws of the study. It kind of shows that even after all reviewers asked for a proper validation of the macroglia-specific KO data remain bits and pieces and are not convincing - at least not to me.

Au: As we indicate above in our responses, the issues of this Reviewer are responded to in full.

Referee #2 (Remarks for Author):

Rev 6. The authors have made a substantial effort to address the reviewers' concerns. However, the specificity of the mouse model for the macroglia-specific deletion of TWINKEL remains highly questionable.

Au: Again, the mouse model and the specificity of GFAP promoter on macroglia has been reported in Ignatenko et al. 2018 in detail. Please see our responses for this reviewer, points 2. and 3. Above.

Rev 7. Importantly, the additional data provided during the revision process, including the new validation experiments in the retina, do not clarify this issue; instead, they further strengthen my concerns regarding the suitability of the GFAP driver line for addressing the question in a celltype-specific manner (also see my detailed concerns below):
(i) The GFAP driver exhibits low recombination efficiency in Müller cells;

Au: Our main finding is exactly in astrocytes, because their reactivation is remarkable (Fig 1C, for example). Muller cells were not the main target, but we noted that they are also lowly targeted. The reactivated astrocytes wrap often themselves around the vasculature in a manner that vessels can be recognized in the GFAP-astrocyte staining (Figure 1C). Because of the major structural changes that astrocytes undergo in their reactivation, the separation of astrocyte from another is challenging. To our strong opinion, the whole-mount staining that we did for astrocytes indicate that without doubt, and we can show that the whole-mount shows two-fold increase in astrocyte area because of their reactivation. The cross-sections were quantified especially for Müller cell activation, as requested by the reviewers.

Rev 8. (ii) information on recombination efficacy in astrocytes is limited and not assessed using the same readout as for Müller cells, preventing a direct comparison; and, most critically, (iii) the line appears to be leaky, driving reporter expression and potentially Cre recombinase activity in a substantial number of neurons within the inner nuclear layer. This neuronal recombination in a considerable number of cells (see Fig. EV3) could plausibly explain the observed Müller cell gliosis, microglial activation, and remodeling of the intermediate and deep retinal plexi as secondary consequences of neuronal dysfunction or degeneration and not, as concluded by the authors, by first hitting astrocytes/macroglia.

Au: We cordially disagree. Also please see our response to this Reviewer’s point 4. Firstly, the primary Twinkle-KO is driven to macroglia, mostly astrocytes. Twinkle-KO in the targeted cells is the primary reason of the consequences, as explained in length above. The secondary

consequence is the vasculopathy and microgliosis. We have meticulously validated the presence of Twinkle KO consequences - mtDNA and COX-I depletion – that are depleted especially in the astrocyte layer. The reactivation of the astrocytes can affect neurons secondarily as well but we found no major dropout of neurons, and our retinal electrophysiology did not support that either. *Therefore, we find no evidence of primary neuronal contribution to the vasculopathy, Our previous and current data indicate that Twinkle is deleted, especially prominent in astrocytes, with this driver.*

Rev. 9. For these reasons, I cannot recommend this study for publication. As the principal limitation lies in the mouse model itself, I do not see how this issue could be adequately resolved through a classical revision. I regret that I cannot provide a more favorable recommendation.

Au: As we indicate above in our responses, the issues of this Reviewer were either solved or were caused by Reviewer misinterpretation.

Comments/concerns:

Rev 10. The following sentence is still incorrect: "Apart from the outer photoreceptor layer, Muller glia's extensive cell bodies span the rest of all the retinal layers, interacting with all cell types." The outer stem process of Müller cells reaches the outer limiting membrane (in fact, they form it), and Müller cell velate sheaths heavily envelop photoreceptor somata in the ONL. Maybe you meant the outer photoreceptor segments instead of a layer. Then please rephrase to avoid misunderstandings!

Au: Thank you for the clarification, now it is rephrased.

Rev 11. A scheme illustrating the wealth of mouse lines used would be helpful.

Au: The cre-line and the floxed Twinkle line as well as TdTomato-line have been described in detail previously in Ignatenko et al. 2018. ***We have now added a graphical abstract as a visual summary to help the readers.***

Rev. 12. Results - I still have my major issues with the validation of the astrocyte-specific KO of TWINKEL in the retina for multiple reasons: (i) The Fig. EV 1C legend misses the information whether the quantification of SOX9+/dTtomato+ cells was confined to the INL or whether astrocytes were included as well. Less than 20% of recombination efficacy in Müller cells is extremely low.

Au: We agree that Müller cells are targeted in low amount, but this is not true for astrocytes. The full astrocyte layer, as shown in whole-mount staining with GFAP-ab in Fig 1C, is remarkably reactivated.

Rev 13. No recombination effic)acy is stated for astrocytes at all - could have easily performed in flatmounts using the SOX9 staining. Fig. EV2 - not clear why the analysis was performed on sections and not flatmounts that would be much more appropriate to study astrocytes.

Au: Figure 1c shows the whole-mount retina with GFAP staining, showing the remarkable astrocyte reactivation, now also quantified as explained above. As mentioned above, maybe the Reviewer misinterpreted the GFAP staining (red-pseudocolour) staining to be tdTomato (red). We added to method a note that quantification of SOX9 /TdTomato was done in the whole image without separating different layers in the Figure EV 4 C (Previously EV1). The analysis in Figure EV5 (previously EV2) was performed on section images to avoid overlap of the layers and present the signal within the target precisely. We agree the possible minor signals in the amacrine and horizontal cells presented on Figure EV4B (previously EV1) which however show no differences for neuronal death by FJC, and the whole retina shows no defects in neuronal function in the retinogram,

Rev 14. (ii) Additionally, adding to this issue with the mouse model - in Fig. EV 1B and Fig. EV2 it looks like a considerable number of neurons of the inner nuclear layer also recombined (potentially amacrine and horizontal cells)...Most likely this also drives the phenotype in this line. A co-staining with some amacrine and horizontal markers would help clarify this key issue of a potentially leaking GFAP promotor it has already been described by others (see Hu et al., Expression Patterns of Inducible Cre Recombinase Driven by Differential Astrocyte-Specific Promoters in Transgenic Mouse Lines. Neurosci Bull. 2020, PMID: 31828740 and in support of my hypothesis a study on retina demonstrating leaking expression in amacrine cells - Rao et al., Exp Eye Res. 2023, PMID: 37659708). (iii) Moreover, the effectiveness of a Cre-mediated deletion of a target gene also largely depends on the fl/fl construct. Therefore reading out dTtomato signals can only be a very rough approximation for what actually happens to TWINKEL. Given that neurons clearly rely on functional mitochondria, the findings of this study could result form primary degeneration of amacrine and horizontal cells followed by macro- and microgliosis. This, unfortunately, stays a major flaw of this study as all the work hypothesis and interpretation base on the assumption that TWINKEL is exclusively knocked out in macroglia - which does not seem to be the case here.

Au: Please see our comments to this repeated text above, to points 2-4 of this Reviewer.

Rev. 15. All in all, these are major flaws of the study. It kind of shows that even after all reviewers asked for a proper validation of the macroglia-specific KO data remain bits and pieces and are not convincing - at least not to me.

4) Results - Validation of KO in the retina (Fig. EV 1D). Seems like almost all dTtomato+ cells also are positive for FJC in both control and Twinkle KO. To me this rather looks like a bleed through from the dTtomato channel than a real indicator of cell death. Would be extremely

careful interpreting such kind of data. A TUNEL assay or caspase staining could/should give much more relevant information.

Au: First of all, there is no significant difference between the controls and KO-mice, indicating no significant cell death occurring in the KO. The FJC gives some signal in occasional cells, and these dots are specific for FJC, indicating that the staining does work. Even if there was some mild bleed-through, the conclusion is that there is no change in cell death between the two genotypes.
TUNEL is not compatible with tdTomato signal, because the latter gets lost during the staining process. FJC is a widely used marker for neurodegeneration (again please see e.g. Ignatenko et al. 2018)

5) If only 20% of Müller cells are targeted, why do the authors observe changes in all three retinal vascular plexi - only the superficial one is stabilized by astrocytes, while the other two (intermediate & deep) rely on Müller cells only.

Au: The neovascularization indeed occurs in all the three layers, which we find highly interesting and indicative of a major metabolic signaling induced by macroglia. As the primary mitochondrial defect is in the astrocytes and some Mueller glia, the neovascularization is a consequence of the pathological signaling, that we propose to involve Pgf and cytokines.

6) The authors state that state that validation of candidates was challenging since many of them were secreted factors hard to detect by stainings. True - but for many of the very sensitive ELISA assays exist to measure changes e.g. in retinal samples.

Au: The inflammation pattern is strong and wide in the RNAseq experiment, and secondary microgliosis well aligns with this finding. We do not consider the ELISA assay to improve considerably the information content of the study to justify production of new mouse cohorts for this purpose.

7) I asked the authors to better assess neurodegeneration especially in the INL. They respond that they - on top of Brn3 quantification - added the FJC analysis in dTtomato positive cells. Well, this does not answer my question, since ideally dTtomato+ cells should not be neurons. Besides - coming back to my comment No. 3, it seems like there is leakiness of the GFAP promotor driven
Cre expression in neurons... Which makes the whole thing even worse.

To address the reviewer's comment, we tested FJC staining to search for dying neurons – or any cells. The FJC signal is not observed in the INL (Fig EV1 D), and no difference exists between Twinkle-KO and the control, strongly supporting the conclusion that the primary macroglial KO of Twinkle does not cause neurodegeneration in the INL or any other parts of the retina. We find no evidence for retinal neurodegeneration by histology or by electrophysiology.

Referee #3 (Remarks for Author):

The authors have addressed all my previous concerns in a very thorough revision including several high quality microscopic images.

Au: Thank you very much for your supportive comment.

1st Apr 2026

Dear Prof. Suomalainen-Wartiovaara, Dear Anu,

Thank you for submitting your revised study. I have now checked the revised files, and I will be able to accept your manuscript once the following minor editorial matters are addressed:

1/ Manuscript text:
- Please indicate any new modification in track changes mode.
- Correct the order and headings of the sections in the manuscript text to: Abstract / The Paper Explained / Introduction / Results / Discussion / Methods / Data Availability / Acknowledgements / Disclosure and Competing Interests Statement / References / Figure Legends / Expanded View Figure Legends.
- Methods:
o Please indicate the origin of the mice.
o Statistics: Please provide a statement on inclusion/exclusion criteria and sample size.
o Add a Graphic section, stating that the visual abstract has been created with BioRender.
- Data availability section: please provide a URL link to the deposited data.
- Author contributions: remove from the manuscript text, and provide instead CRediT (Contributor Role Taxonomy) terms in the submission system.
- Disclosure and Competing Interests statement: please add 'K.A. and A. S.-W are editorial advisory board members'.
- References: please correct to alphabetical order, with 10 author names listed before et al.

2/ Figures:
- A callout is missing for Fig 1D; please include panel callouts for Fig EV2, and correct the sequence for the citations of Fig EV1D-E, which are currently after Fig EV2 and EV3.
- Please provide Table 1 in an editable format, as it will be typeset, or consider making it an expanded view table if you prefer to keep the current format. Remove the legend from the manuscript text and add it to the table file.
- During our standard image analysis, we detected potential duplication in the figure set. We kindly invite you to check and correct the composition of Figure 1D.
- Please address the queries from our data editors in the figure legends:
1. Please indicate the statistical test used for data analysis in the legends of figures EV3 B
2. Please note that information related to n is missing in the legends of figures 5B, EV1 E, EV2 D
3. Please note that the error bars are msising/not defined in the legend of figure EV1 B, EV7.
4. Please define the white dotted lines for all figure panels, as appropriate.

3/ Please note that all corresponding authors are required to supply an ORCID ID for their name upon submission of a revised manuscript. An ORCID identifier is missing for G. Ince-Dunn.

4/ Source Data:
- Please zip the numerical data (currently in file 'Data Set)' to the corresponding figures' remaining source data file.
- Please provide numerical data for Figure 1C (excel).
- Please check and correct the labelling for Fig. 2 source data files.

5/ Please provide The paper explained: EMBO Molecular Medicine articles are accompanied by a summary of the articles to emphasize the major findings in the paper and their medical implications for the non-specialist reader. Please provide a draft summary of your article highlighting
- the medical issue you are addressing,
- the results obtained and
- their clinical impact.
This may be edited to ensure that readers understand the significance and context of the research. Please refer to any of our published articles for an example.

6/ Synopsis:
- please upload the text individually.
- please upload the visual abstract without the synopsis text in a jpg, png or tiff file, 550 px wide x 300 px high.

7/ Reagents and Tools Table:
Please complete the table and remove the comments.

8/ As part of the EMBO Publications transparent editorial process initiative (see our Editorial at http://embomolmed.embopress.org/content/2/9/329), EMBO Molecular Medicine will publish online a Review Process File (RPF)

to accompany accepted manuscripts.
This file will be published in conjunction with your paper and will include the anonymous referee reports, your point-by-point response and all pertinent correspondence relating to the manuscript. Let us know whether you agree with the publication of the RPF.
Please note that the Authors checklist will be published at the end of the RPF.

I look forward to receiving your revised manuscript.

Sincerely,

Lise

Lise Roth, PhD
Senior Editor
EMBO Molecular Medicine

The authors addressed the remaining editorial issues.

21st Apr 2026

Dear Prof. Suomalainen-Wartiovaara, Dear Anu,

We are pleased to inform you that your manuscript is accepted for publication and is now being sent to our publisher to be included in the next available issue of EMBO Molecular Medicine.

Your manuscript will be processed for publication by EMBO Press. It will be copy edited and you will receive page proofs prior to publication. Please note that you will be contacted by Springer Nature Author Services to complete licensing and payment information.

You may qualify for financial assistance for your publication charges - either via a Springer Nature fully open access agreement or an EMBO initiative. Check your eligibility: https://link.springer.com/journal/44321/how-to-publish-with-us

Should you be planning a Press Release on your article, please get in contact with embo_production@springernature.com as early as possible in order to coordinate publication and release dates.

If you have any questions, please do not hesitate to contact the Editorial Office. Thank you for your contribution to EMBO Molecular Medicine.

Yours sincerely,

Lise

Lise Roth, Ph.D
Senior Editor
EMBO Molecular Medicine

>>> Please note that it is EMBO Molecular Medicine policy for the transcript of the editorial process (containing referee reports and your response letter) to be published as an online supplement to each paper. If you do NOT want this, you will need to inform the Editorial Office via email immediately. More information is available here: https://link.springer.com/partners/embo-press/editorial-policies#Peer%20review

EMBO Press Author Checklist

Corresponding Author Name: Anu Suomalainen Wartiovaara, Gulayse Ince-Dunn	
Journal Submitted to: EMBO Molecular Medicine	
Manuscript Number: EMM-2025-21211-V2	

USEFUL LINKS FOR COMPLETING THIS FORM
The EMBO Journal - Author Guidelines
EMBO Reports - Author Guidelines
Molecular Systems Biology - Author Guidelines
EMBO Molecular Medicine - Author Guidelines

Reporting Checklist for Life Science Articles (updated January
This checklist is adapted from Materials Design Analysis Reporting (MDAR) Checklist for Authors. MDAR establishes a minimum set of requirements in transparent reporting in the life sciences (see Statement of Task: 10.31222/osf.io/9sm4x). Please follow the journal's guidelines in preparing your
Please note that a copy of this checklist will be published alongside your article.

Abridged guidelines for figures
1. Data
The data shown in figures should satisfy the following conditions:
- the data were obtained and processed according to the field's best practice and are presented to reflect the results of the experiments in an accurate and unbiased manner.
- ideally, figure panels should include only measurements that are directly comparable to each other and obtained with the same assay.
- plots include clearly labeled error bars for independent experiments and sample sizes. Unless justified, error bars should not be shown for technical
- if n<5, the individual data points from each experiment should be plotted. Any statistical test employed should be justified.
- Source Data should be included to report the data underlying figures according to the guidelines set out in the authorship guidelines on Data

2. Captions
Each figure caption should contain the following information, for each panel where they are relevant:
- a specification of the experimental system investigated (eg cell line, species name).
- the assay(s) and method(s) used to carry out the reported observations and measurements.
- an explicit mention of the biological and chemical entity(ies) that are being measured.
- an explicit mention of the biological and chemical entity(ies) that are altered/varied/perturbed in a controlled manner.
- the exact sample size (n) for each experimental group/condition, given as a number, not a range;
- a description of the sample collection allowing the reader to understand whether the samples represent technical or biological replicates (including how many animals, litters, cultures, etc.).
- a statement of how many times the experiment shown was independently replicated in the laboratory.
- definitions of statistical methods and measures:
  - common tests, such as t-test (please specify whether paired vs. unpaired), simple χ2 tests, Wilcoxon and Mann-Whitney tests, can be unambiguously identified by name only, but more complex techniques should be described in the methods section;
  - are tests one-sided or two-sided?
  - are there adjustments for multiple comparisons?
  - exact statistical test results, e.g., P values = x but not P values < x;
  - definition of 'center values' as median or average;
  - definition of error bars as s.d. or s.e.m.

Please complete ALL of the questions below.
Select "Not Applicable" only when the requested information is not relevant for your study.

Materials

Newly Created Materials	Information included in the manuscript?	In which section is the information available? (Reagents and Tools Table, Materials and Methods, Figures, Data Availability Section)
New materials and reagents need to be available; do any restrictions apply?	Not Applicable	

Antibodies	Information included in the manuscript?	In which section is the information available? (Reagents and Tools Table, Materials and Methods, Figures, Data Availability Section)
For **antibodies** provide the following information:<br>- Commercial antibodies: RRID (if possible) or supplier name, catalogue number and or/clone number<br>- Non-commercial: RRID or citation	Yes	Materials and methods, Immunohistochemistry

DNA and RNA sequences	Information included in the manuscript?	In which section is the information available? (Reagents and Tools Table, Materials and Methods, Figures, Data Availability Section)
**Short novel DNA or RNA including primers, probes**: provide the sequences.	Not Applicable	

Cell materials	Information included in the manuscript?	In which section is the information available? (Reagents and Tools Table, Materials and Methods, Figures, Data Availability Section)
**Cell lines:** Provide species information, strain. Provide accession number in repository **OR** supplier name, catalog number, clone number, and/**OR** RRID.	Not Applicable	
**Primary cultures:** Provide species, strain, sex of origin, genetic modification status.	Not Applicable	
Report if the cell lines were recently **authenticated** (e.g., by STR profiling) and tested for mycoplasma contamination.	Not Applicable	

Experimental animals	Information included in the manuscript?	In which section is the information available? (Reagents and Tools Table, Materials and Methods, Figures, Data Availability Section)
**Laboratory animals or Model organisms:** Provide species, strain, sex, age, genetic modification status. Provide accession number in repository **OR** supplier name, catalog number, clone number, **OR** RRID.	Yes	Materials and methods, Mouse Models
**Animal observed in or captured from the field:** Provide species, sex, and age where possible.	Not Applicable	
Please detail **housing and husbandry conditions**.	Yes	Materials and methods, Mouse Models

Plants and microbes	Information included in the manuscript?	In which section is the information available? (Reagents and Tools Table, Materials and Methods, Figures, Data Availability Section)
**Plants:** provide species and strain, ecotype and cultivar where relevant, unique accession number if available, and source (including location for collected wild specimens).	Not Applicable	
**Microbes:** provide species and strain, unique accession number if available, and source.	Not Applicable	

Human research participants	Information included in the manuscript?	In which section is the information available? (Reagents and Tools Table, Materials and Methods, Figures, Data Availability Section)
If collected and within the bounds of privacy constraints report on age, sex and gender or ethnicity for all study participants.	Not Applicable	

Core facilities	Information included in the manuscript?	In which section is the information available? (Reagents and Tools Table, Materials and Methods, Figures, Data Availability Section)
If your work benefited from core facilities, was their service mentioned in the acknowledgments section?	Yes	Acknowledgements

Design

Study protocol	Information included in the manuscript?	In which section is the information available? (Reagents and Tools Table, Materials and Methods, Figures, Data Availability Section)
If study protocol has been **pre-registered, provide DOI in the manuscript**. For clinical trials, provide the trial registration number **OR** cite DOI.	Not Applicable	
Report the **clinical trial registration number** (at ClinicalTrials.gov or equivalent), where applicable.	Not Applicable	

Laboratory protocol	Information included in the manuscript?	In which section is the information available? (Reagents and Tools Table, Materials and Methods, Figures, Data Availability Section)
Provide DOI OR other citation details if **external detailed step-by-step protocols** are available.	Yes	Materials and methods

Experimental study design and statistics	Information included in the manuscript?	In which section is the information available? (Reagents and Tools Table, Materials and Methods, Figures, Data Availability Section)
Include a statement about **sample size** estimate even if no statistical methods were used.	Yes	Materials and methods, Figure legends
Were any steps taken to minimize the effects of subjective bias when allocating animals/samples to treatment (e.g. **randomization procedure**)? If yes, have they been described?	Yes	Materials and methods
Include a statement about **blinding** even if no blinding was done.	Yes	Materials and methods
Describe **inclusion/exclusion criteria** if samples or animals were excluded from the analysis. Were the criteria pre-established?<br><br>If sample or data points were omitted from analysis, report if this was due to attrition or intentional exclusion and provide justification.	Yes	Materials and methods
For every figure, are **statistical tests** justified as appropriate? Do the data meet the assumptions of the tests (e.g., normal distribution)? Describe any methods used to assess it. Is there an estimate of variation within each group of data? Is the variance similar between the groups that are being statistically compared?	Yes	Materials and methods

Sample definition and in-laboratory replication	Information included in the manuscript?	In which section is the information available? (Reagents and Tools Table, Materials and Methods, Figures, Data Availability Section)
In the figure legends: state number of times the experiment was **replicated** in laboratory.	Yes	Figure legends
In the figure legends: define whether data describe **technical or biological replicates**.	Yes	Figure legends

Ethics

Ethics	Information included in the manuscript?	In which section is the information available? (Reagents and Tools Table, Materials and Methods, Figures, Data Availability Section)
Studies involving **human participants**: State details of **authority granting ethics approval** (IRB or equivalent committee(s), provide reference number for approval.	Not Applicable	
Studies involving **human participants**: Include a statement confirming that **informed consent** was obtained from all subjects and that the experiments conformed to the principles set out in the WMA Declaration of Helsinki and the Department of Health and Human Services Belmont Report.	Not Applicable	
Studies involving **human participants: For** publication of **patient photos**, include a statement confirming that consent to publish was obtained.	Not Applicable	
Studies involving experimental **animals**: State details of **authority granting ethics approval** (IRB or equivalent committee(s), provide reference number for approval. Include a statement of compliance with ethical regulations.	Yes	Materials and methods, Mouse Models
Studies involving **specimen and field samples:** State if relevant **permits** obtained, provide details of authority approving study; if none were required, explain why.	Not Applicable	

Dual Use Research of Concern (DURC)	Information included in the manuscript?	In which section is the information available? (Reagents and Tools Table, Materials and Methods, Figures, Data Availability Section)
Could your study fall under dual use research restrictions? Please check biosecurity documents and list of **select agents and toxins** (CDC): https://www.selectagents.gov/sat/list.htm	Not Applicable	
If you used a select agent, is the security level of the lab appropriate and reported in the manuscript?	Not Applicable	
If a study is subject to dual use research of concern regulations, is the name of the **authority granting approval and reference number** for the regulatory approval provided in the manuscript?	Not Applicable	

Reporting

The MDAR framework recommends adoption of discipline-specific guidelines, established and endorsed through community initiatives. Journals have their own policy about requiring specific guidelines and recommendations to complement MDAR.

Adherence to community standards	Information included in the manuscript?	In which section is the information available? (Reagents and Tools Table, Materials and Methods, Figures, Data Availability Section)
State if relevant guidelines or checklists (e.g., **ICMJE, MIBBI, ARRIVE, PRISMA**) have been followed or provided.	Not Applicable	
For **tumor marker prognostic studies,** we recommend that you follow the **REMARK** reporting guidelines (see link list at top right). See author guidelines, under 'Reporting Guidelines'. Please confirm you have followed these guidelines.	Not Applicable	
For **phase II and III randomized controlled trials**, please refer to the **CONSORT** flow diagram (see link list at top right) and submit the CONSORT checklist (see link list at top right) with your submission. See author guidelines, under 'Reporting Guidelines'. Please confirm you have submitted this list.	Not Applicable	

Data Availability

Data availability	Information included in the manuscript?	In which section is the information available? (Reagents and Tools Table, Materials and Methods, Figures, Data Availability Section)
Have **primary datasets** been deposited according to the journal's guidelines (see 'Data Deposition' section) and the respective accession numbers provided in the Data Availability Section?	Yes	Materials and methods
Were **human clinical and genomic datasets** deposited in a public access-controlled repository in accordance to ethical obligations to the patients and to the applicable consent agreement?	Not Applicable	
Are **computational models** that are central and integral to a study available without restrictions in a machine-readable form? Were the relevant accession numbers or links provided?	Not Applicable	
If publicly available data were reused, provide the respective **data citations in the reference list**.	Yes	Materials and methods
